# Oxonium-Embedded Benzo[*b*]perylene: A Redox-Switchable Closed-Shell π‑Cation, and Its Paramagnetic Charge-Transfer Salt with TCNE^•–^

**DOI:** 10.1021/acscentsci.6c01018

**Published:** 2026-08-31

**Authors:** Chao Duan, Yuan Xue, Daniel B. Straus, Alexander Mariscal, Justin L. Ratkovec, Navamoney Arulsamy, Jianping Zhao, Mei Wang, Obadiah G. Reid, Lukasz Wojtas, Xuesong Li, Wenqi Liu, Penghao Li

**Affiliations:** † Department of Chemistry & Biochemistry, 8083 The University of Mississippi, University, Mississippi 38677, United States; ‡ Department of Chemistry, 5783 Tulane University, New Orleans, Louisiana 70118, United States; § Department of Chemistry, 7831 University of South Florida, Tampa, Florida 33620, United States; ∥ Department of Chemistry, 1877 University of Colorado Boulder, Boulder Colorado 80309, United States; ⊥ Department of Chemistry, 4416 University of Wyoming, Laramie, Wyoming 82071, United States; # National Center for Natural Products Research, School of Pharmacy, The University of Mississippi, University, Mississippi 38677, United States; 7 Natural Products Utilization Research Unit, Agricultural Research Service, United States Department of Agriculture, University, Mississippi 38677, United States; 8 Chemistry and Nanoscience Center, National Laboratory of the Rockies, Golden, Colorado 80401, United States; 9 Renewable and Sustainable Energy Institute, 1877 University of Colorado Boulder, Boulder, Colorado 80309, United States

## Abstract

Organic cations and radicals are promising building components for organic optoelectronics, spintronics and quantum information processing. Realizing their full potential requires strategies to enhance their stability and precisely control their solid-state organization. Herein, we describe a modular synthetic strategy via photoelectrocyclization in the presence of tetracyanoethylene (TCNE) to access a series of closed-shell phenalenyl-derived cations featuring an oxonium-embedded benzo­[*b*]­perylene (**OBP** ^**+**^) π-skeleton. The resulting **OBP** ^**+**^ cations can be reversibly reduced to generate neutral open-shell phenalenyl-derived radicals. Noteworthily, we discovered a single crystal of **OBP3·TCNE** ^**•**^ that readily formed from the reaction mixture, displaying one-dimensional (1D) π-stacked arrays of alternating closed-shell **OBP3** ^**+**^ cation and open-shell TCNE^•–^ radical anion. This solid-state arrangement stabilizes otherwise reactive TCNE^•–^ species, preserving its open-shell character even after one month of ambient exposure. **OBP3·TCNE** ^**•**^ is a rare example of purely organic paramagnetic salt and has been systematically investigated using integrated spectroscopic, magnetic, and computational analyses, together with preliminary photoconductivity measurements (ϕΣμ = 1.4 × 10^**–**5^ cm^2^ V^**–**1^ s^**–**1^). Overall, this work heralds a versatile photochemical route to redox-switchable cationic π-scaffolds and persistent open-shell crystalline organic materials.

## Graphical abstract

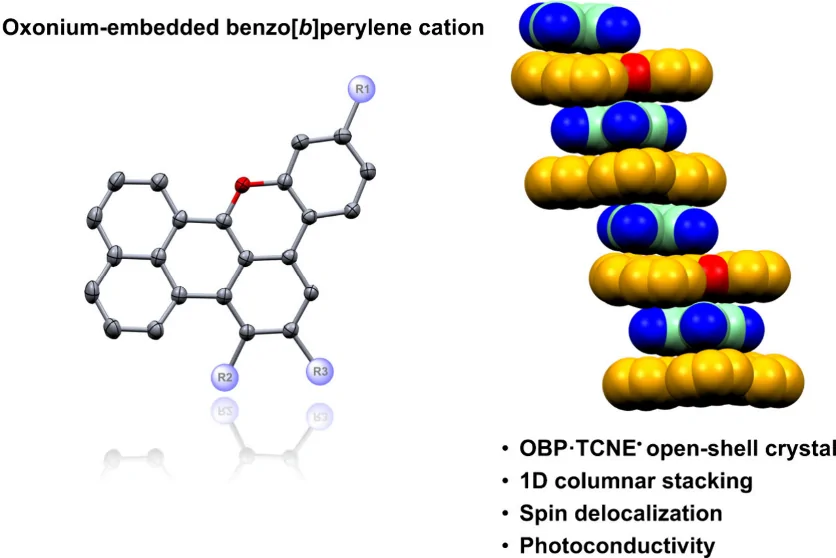

## Introduction

Cations of π-conjugated systems play^1–5^ a crucial role in advancing organic electronics, spintronics, molecular machines, and photoredox catalysis. Introducing positive charge into extended π-frameworks reshapes^6–9^ their electronic landscapes, enabling optical, magnetic, and charge-transport properties that are inaccessible in neutral analogues. Because of these distinctive features, both open-shell and closed-shell π-conjugated cations have been extensively studied^10–13^ in the last several decades. Salts of open-shell π-conjugated cations have been demonstrated^14^ as high-performance organic semiconductors. These radical cations can be easily generated by oxidation of neutral π-conjugated precursors, and their structural diversity provides a powerful handle to tune the solid-state conductivity. In parallel, closed-shell π-conjugated cations typically exhibit^5,15–17^ switchable redox behavior, opening opportunities in energy storage, molecular recognition and catalysis. A prototypical example is viologen (4,4′-bipyridinium), a closed-shell dication which forms^18^ a pimer (π-dimer) upon one-electron reduction to its radical cation. This reversible dimerization has been widely leveraged^19–21^ in the design of molecular pumps and artificial molecular muscles.

Phenalenyl, the smallest open-shell graphene fragment with zigzag edges, has attracted great attention in the chemistry and materials community because of its amphoteric redox behavior and distinctive magnetic, photophysical, and charge transport properties, which are promising for technologies in organic optoelectronics, spintronics and quantum sensing.^22–28^ Sterically bulky substituents, π-extension, and heteroatom incorporation are commonly employed^29–37^ to suppress the dimerization of unpaired electrons into σ-bonded species. More recently, oxonium-embedded phenalenyl-derived cations have emerged as stable precursors to phenalenyl-derived radicals. These closed-shell π-cations are isolable and can be reversibly reduced to open-shell states stabilized electronically by the incorporated oxygen atoms that promote charge delocalization and suppress radical dimerization. This strategy was first demonstrated by Bucher and co-workers in the synthesis of naphthoxanthenyl^38^ and xanthenium^39^ cations and their corresponding neutral radicals. Subsequently, Chen and co-workers expanded^40^ this approach to access bisphenalenyl-derived radical cations with appreciable solid-state electrical conductivity. In both cases, a key step involves a Michael-type addition of organometallic reagents to phenalenone. However, the use of reactive Grignard or organolithium reagents inherently limits the substrate scope. Although You and co-workers later introduced^41^ a C–H annulation strategy that allows quick access to a more diverse library of phenalenyl-fused pyrylium cations, this method requires expensive precious metal catalysis and air- and moisture- sensitive reagents to construct the core polycyclic skeleton. These limitations highlight the need for more general and operationally simple synthetic approaches.

Herein, we report a photochemical strategy to access oxonium-embedded benzo­[*b*]­perylene (**OBP** ^**+**^) as an unexplored class of closed-shell phenalenyl-derived cations (Figure 1a). This approach relies on the photolysis of 7-arylbenzanthones in the presence of tetracyanoethylene (TCNE), proceeding through electrocyclization via a β-phenyl quenching (BPQ) mechanism followed by a sequential hydrogen-electron transfer. The 7-arylbenzanthone precursors can be easily assembled through a Diels–Alder (D–A) reaction between a common cyclopentadienone (**3** in Scheme 1) and a variety of dienophiles, enabling rapid structural diversification. Notably, one cation, **OBP3** ^**+**^, crystallizes with TCNE^•–^ directly from the reaction mixture to form single crystals. Single-crystal X-ray diffraction (SCXRD) analysis reveals unique one-dimensional (1D) alternating π-stacks of closed-shell cation and open-shell anion (Figure 1b), a packing motif that may be favorable for charge and spin transport. Consistent with this, an appreciable photoconductivity with a yield–mobility product (ϕΣμ) value of 1.4 × 10^**–**5^ cm^2^ V^**–**1^ s^**–**1^ was measured from time-resolved microwave conductivity (TRMC) spectroscopy. Computational analyses further suggest possible spin delocalization between TCNE^•–^ and **OBP3** ^**+**^. Magnetic susceptibility measurement indicates that the **OBP3·TCNE** ^**•**^ salt behaves as a classic S = 1/2 paramagnet with negligible antiferromagnetic exchange interactions, and solid-state electron paramagnetic resonance (EPR) spectroscopy confirms retention of the open-shell character after one month of ambient exposure. Furthermore, **OBP** ^**+**^ cations undergo reversible single-electron reduction to neutral open-shell states (Figure 1c), highlighting their potential as redox-switchable materials.

**Figure 1:**
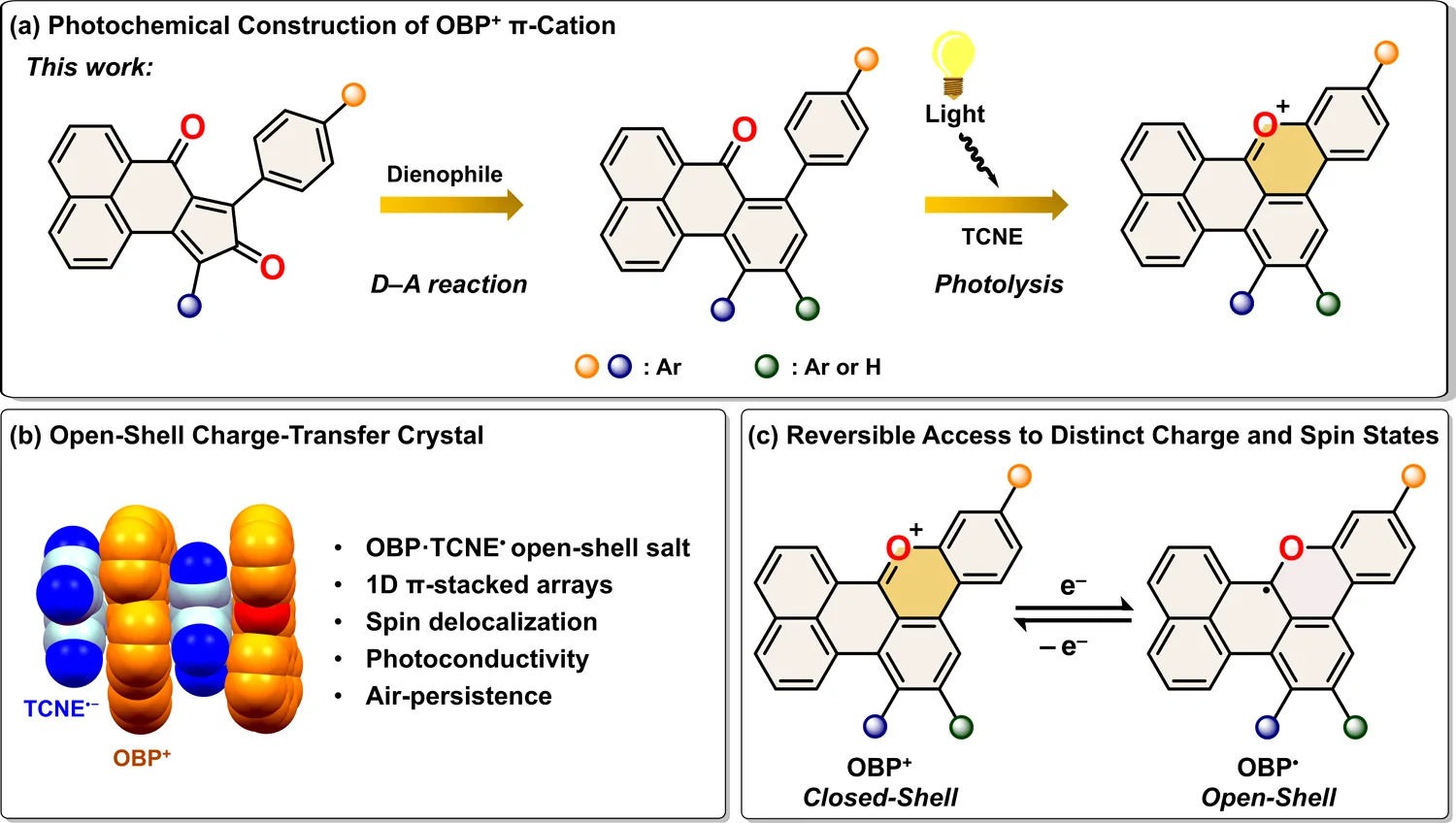
(a) Our synthetic strategy toward oxonium-embedded benzo­[*b*]­perylene (**OBP** ^**+**^), a new type of closed-shell π-conjugated cations. (b) An open-shell crystal of **OBP·TCNE** ^**•**^ featuring 1D π-stacked arrays of alternating cations and radical anions. (c) Reversible interconversion between the closed-shell π-cation (**OBP** ^**+**^) and the open-shell neutral radical (**OBP** ^**•**^).

**Scheme 1:**
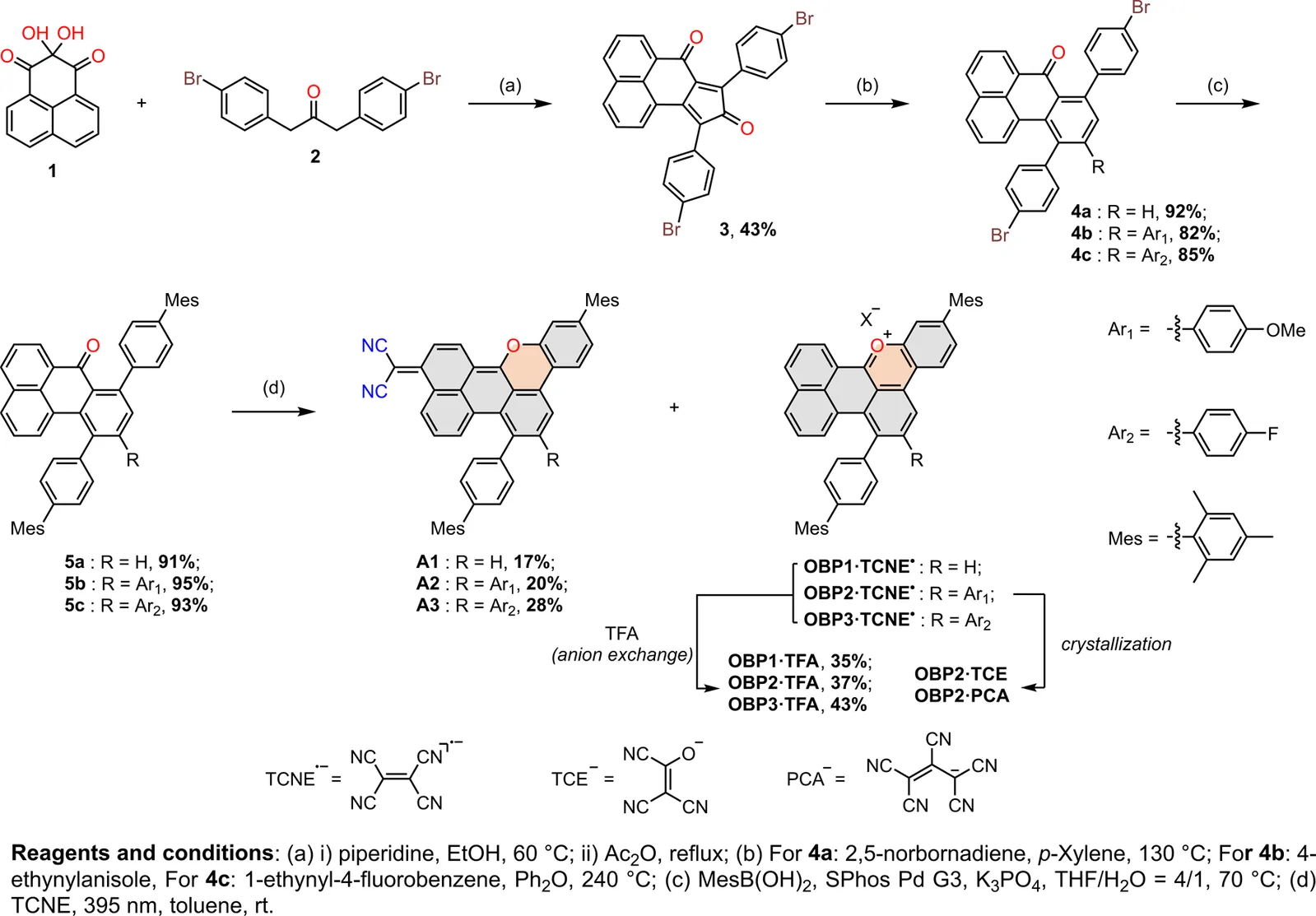
Synthetic Routes and Reaction Conditions for the Preparation of π-Cations **OBP1** ^**+**^–**OBP3** ^**+**^ and Adducts **A1**–**A3**

## Results and Discussion

### Synthesis of Oxonium-Embedded Benzo­[*b*]­perylene π-Cations (OBP^+^)

Our synthetic strategy centers on the construction of 7-arylbenzanthones which are amenable to photoelectrocyclization via a BPQ mechanism.^42–44^ Depicted in Scheme 1, the synthesis began with a Knoevenagel condensation^45,46^ of **1** and **2** to form cyclopentadienone **3** in 43% yield. The subsequent D–A reaction of **3** with norbornadiene or arylacetylene afforded **4a**–**c** in 82%–92% yields. Substituents with varying electronic properties (hydrogen, 4-methoxyphenyl, and 4-fluorophenyl) were introduced to systematically probe their effects on the photochemical reactivity of 7-arylbenzanthones and physical properties of the proposed **OBP** ^**+**^ cations. Notably, the D–A reactions with arylacetylenes exhibited high regioselectivity, predominantly yielding one regioisomer. This selectivity can be rationalized by the polarization of frontier molecular orbitals (FMOs) of the diene by the electron-deficient carbonyl and the dienophile by the electron rich aryl group, which favors the formation of the *para*-isomer to maximize FMO overlap in the transition state (Figure S1).^47^ To enhance solubility, mesityl (Mes) groups were installed via Suzuki–Miyaura cross-coupling to produce **5a**–**c** in high yields (91–95%).

With a series of 7-arylbenzanthones in hand, we next investigated their photochemical transformation to the targeted **OBP** ^**+**^ cations. Irradiation at 395 nm was selected because **5a**–**c** exhibit a broad absorption band centered at this wavelength (Figure S2a). The transient photocyclization products of **5a**–**c** then react with TCNE to afford **OBP1** ^**+**^–**OBP3** ^**+**^. After counterion exchange (TFA/CH_2_Cl_2_/MeOH), salts of **OBP** ^**+**^ cations, **OBP1·TFA**, **OBP2·TFA**, and **OBP3·TFA**, were isolated as dark-blue solids in 35%, 37% and 43% yields, respectively. Additionally, we also isolated neutral quinoidal dicyanomethylene adducts **A1**–**A3** as the side products in 17–28% yields. The structures of these **OBP** ^**+**^ cations and adducts were confirmed by nuclear magnetic resonance (NMR) spectroscopy, high-resolution mass spectrometry (HRMS), and SCXRD (vide infra).

The reaction process can be monitored by UV–vis absorption spectroscopy. Upon irradiation, a broad absorption band spanning 500–700 nm emerges (Figure S2b), accompanied by a rapid color change from yellow to green, consistent with the formation of cationic species and adducts. Control experiments confirm that no reaction occurs in the absence of TCNE or light (Figure S2c). Notably, TCNE is essential for the adduct formation, as replacing it with 2,3-dichloro-5,6-dicyano-1,4-benzoquinone (DDQ) as the oxidant leads exclusively to the formation of cations.

### Mechanistic Investigations

Guided by the electrocyclization pathways in the BPQ mechanism,^42–44^ we employed density functional theory (DFT) calculations to probe the formation of **OBP** ^**+**^ cation and the associated adduct, using **5a** as a model system (Figure 2). Our calculations predicted that the S_0_→S_1_ transition lies in the visible region (λ_max,calc_ = 390 nm), in agreement with the experimental value (λ_max,exp_ = 408 nm; Figure S2a), thereby validating the suitability of 395 nm irradiation in the experiments. Geometric relaxation on the S_1_ potential energy surface reduces the dihedral angle between rings A and B and shortens the reactive C–O distance (Figure S2d), yielding **5a­(S**_**1**_^**react**^ **)** with increased coplanarity and enabling a symmetry-allowed 6π electrocyclization via the transition state **5a­(S**_**1**_^**TS**^ **)** (ΔΔ*G* ^‡^ = +2.2 kcal mol^–1^) to generate the transient cyclized intermediate **cyc­(S**_**1**_ **)**. Crucial dihedral angles and C–O distances along the reaction coordinates are compiled in Table S1. These results indicate that the reaction pathway enabled by **5a­(S**_**1**_^**react**^ **)** is kinetically accessible. The rapid electron transfer from **cyc­(S**_**1**_ **)** to TCNE affords the thermodynamically favorable radical cation **Int1** ^**•+**^ (Δ*G*_*rxn*_ = −19.0 kcal mol^–1^). Alternatively, electron transfer from **cyc­(S**_**0**_ **)**, accessed via internal conversion, is likewise viable, albeit mildly endergonic (Δ*G*_*rxn*_ = +8.9 kcal mol^–1^). Notably, both **cyc­(S**_**1**_ **)** and **cyc­(S**_**0**_ **)** are likely transient species in reversible equilibrium with the starting material,^43^ consistent with control experiment in the absence of TCNE, which affords only unreacted precursor. Electron transfer to TCNE from either state likely competes with ring-opening, irreversibly draining this equilibrium toward **Int1** ^**•+**^, which undergoes subsequent homolytic C–H cleavage (Δ*G*_*rxn*_ = +16.1 kcal mol^–1^) to generate the cationic photoproduct **OBP1** ^**+**^.

**Figure 2:**
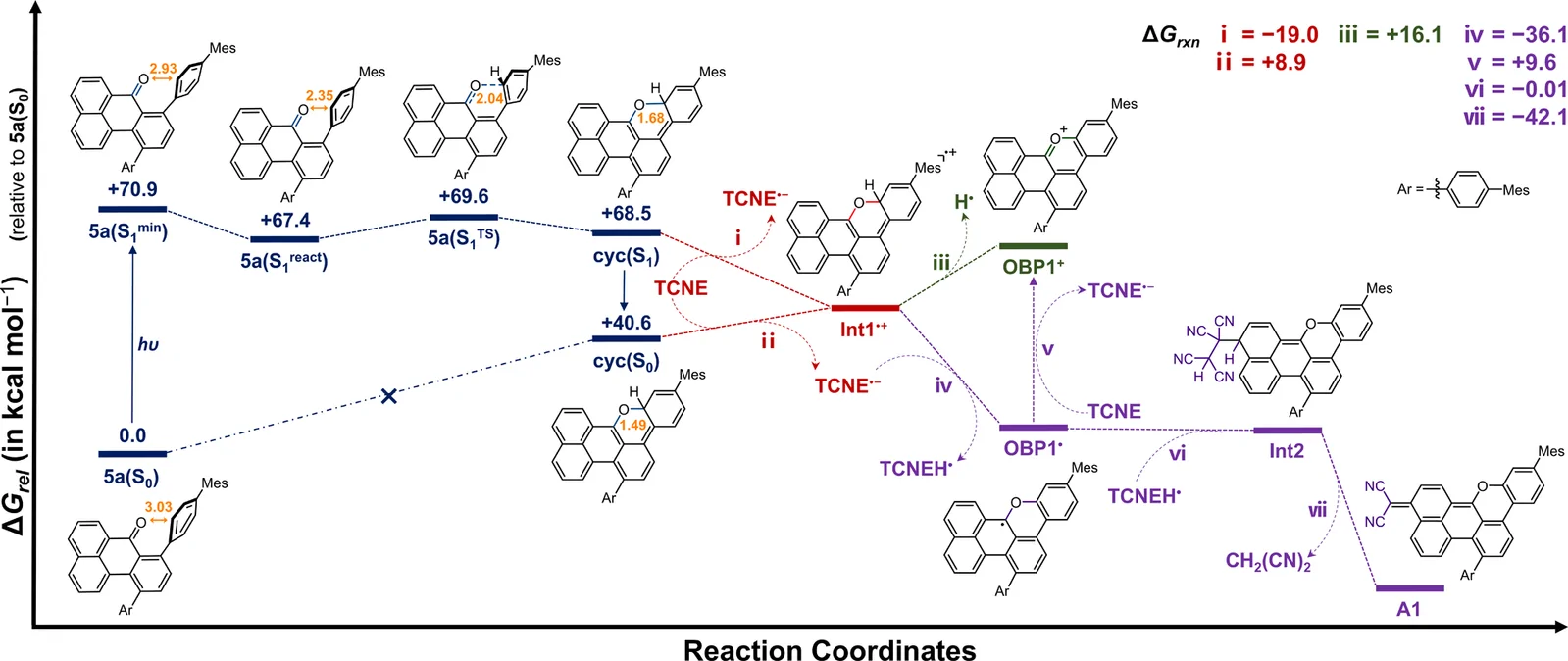
Free energy profile of photolysis of **5a** in the presence of TCNE, determined by DFT calculations at the (U)­CAM-B3LYP/6–311+G** level of theory. Relative Gibbs free energies are given in kcal mol^–1^, and C–O distances (orange) are given in Å.

To evaluate the possible reaction pathways leading to the adduct formation, we first analyzed the spin density and atomic dipole-corrected Hirshfeld (ADCH) charge of **Int1** ^**•+**^. Pathways (Schemes S3–S4) involving direct radical coupling or radical addition at the reactive 4-position were ruled out because of negligible spin and charge density at that site (Figure S5). Instead, we proposed a more plausible mechanism involving a sequential electron-transfer–proton-transfer–radical coupling pathway (Scheme S2). In this scenario, proton transfer from **Int1** ^**•+**^ to TCNE^•–^ (Δ*G*_*rxn*_ = –36.1 kcal mol^–1^) generates^48,49^ **OBP1** ^**•**^ and a neutral radical TCNEH^•^. Subsequent thermoneutral radical coupling and *β-*fragmentation (Δ*G*_*rxn*_ = –42.1 kcal mol^–1^) afford the adduct **A1**. In comparison, single-electron transfer from **OBP1** ^**•**^ to TCNE, which regenerates **OBP1** ^**+**^, is thermodynamically less favorable (Δ*G*_*rxn*_ = +9.6 kcal mol^–1^). Nevertheless, this step remains accessible under the reaction conditions and provides an additional pathway to the formation of **OBP1** ^**+**^, which likely accounts for its higher yield relative to **A1**. When DDQ is used as the trapping agent, adduct formation is found to be thermodynamically unfavorable because C–C bond formation disrupts the aromaticity of DDQH^•^. A detailed discussion is provided in the Supporting Information (Figures S3–S4).

### Single-Crystal X-ray Diffraction Analysis

During initial reaction screening, photolysis of **5c** with TCNE in toluene produced a reaction mixture that appeared silky after overnight irradiation under stirring, suggesting the formation of aggregates. Upon dilution and removal of perturbation, metallic purple crystals suitable for SCXRD were obtained. Structure analysis revealed a 1:1 salt of **OBP3** ^**+**^ and TCNE^•–^ (**OBP3·TCNE** ^**•**^), representing a rare example of purely organic salt containing closed-shell cations and open-shell anions.^50^ The π-framework of **OBP3** ^**+**^ shows high planarity, with only a small bay torsion (13.6°) arising from steric repulsion of the aryl substituent at the 10-position (Figure 3a). Bond-length alternation (Figure S16a) across the **OBP3** ^**+**^ scaffold is minimal (1.367–1.474 Å), consistent with a highly delocalized cationic π-system, except for a slightly elongated bond (1.474 Å)^51^ at the sterically congested bay region. The pyrylium unit possesses two inequivalent C–O bonds (1.330 and 1.382 Å), similar to those observed^38,39,52^ in oxonium-embedded analogues. Additionally, two crystallographically independent TCNE^•–^ units are present, each displaying elongated central C–C bonds (1.417 and 1.424 Å vs ca. 1.34 Å in neutral TCNE;^53^ Figure S17), in agreement with literature values.

**Figure 3:**
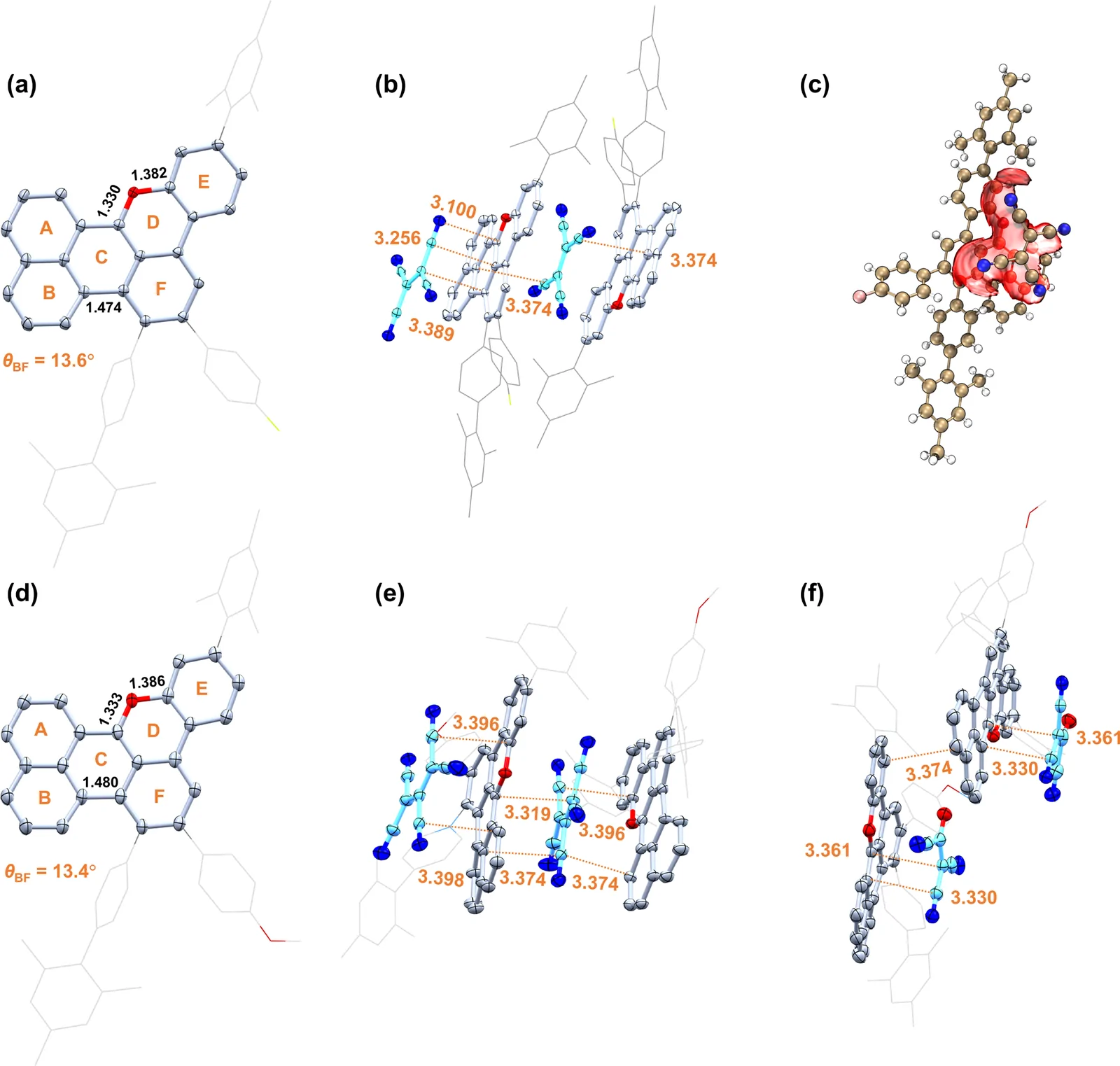
ORTEP representations of π-cations (a) **OBP3** ^**+**^ and (d) **OBP2** ^**+**^ with thermal ellipsoids drawn at the 50% probability level. Packing views showing 1D columnar assemblies for (b) **OBP3·TCNE** ^•^, (e) **OBP2·PCA**, and (f) **OBP2·TCE**. (c) IGMH analysis of **OBP3·TCNE** ^•^ showing noncovalent interaction patterns as red isosurfaces (isovalue = 0.002 au). Distances in Å. Hydrogen atoms, solvent molecules, and peripheral substituents are omitted or depicted in a wireframe style for clarity.

A notable feature (Figure 3b) is the formation of 1D supramolecular columns composed of alternating **OBP3** ^**+**^ and TCNE^•–^ units through face-centered π-π interactions (3.100–3.389 Å). Hirshfeld partition (IGMH) analysis^54^ supports the presence of significant noncovalent interactions between the cation–anion pair (Figures 3c and S20). This packing motif stabilizes the monomeric TCNE^•–^ moiety, whereas preparation of such paramagnetic salts by conventional counterion exchange is generally not feasible because of the intrinsic instability of the monomeric TCNE^•–^ species and its strong tendency to form the diamagnetic dimer.^55^

In contrast, photolysis of a still solution of **5b** and TCNE did not yield crystals within 24 h, yet red-brown crystals formed after standing for 72 h. SCXRD analysis revealed two distinct salts of **OBP2** ^**+**^ with different organic counterions: 1,1,2,3,3-pentacyanoallyl anion (PCA^**–**^) or 1,2,2-tricyanoethenolate anion (TCE^**–**^). These anions likely arise^53^ from the hydrolysis or oxidation of TCNE^•–^ during the reaction or crystallization process. The bond lengths (Figure 3d and Figure S16b) of **OBP2** ^**+**^ (1.369–1.480 Å) closely match those of **OBP3** ^**+**^, indicating minimal electronic perturbation by the electron-rich 4-methoxyphenyl group. **OBP2·PCA** and **OBP2·TCE** salts both exhibit 1D linear stacks of alternating cations and corresponding anions with π-distances of ca. 3.34 Å and 3.30 Å, respectively. In **OBP2·PCA**, adjacent cations adopt a 90° rotational offset within the column (Figure 3e), whereas **OBP2·TCE** displays a slipped head-to-tail stacking arrangement (Figure 3f).

No crystals of **OBP1** ^**+**^ salts were obtained from the photolysis of **5a** under the same conditions. The distinct crystallization outcomes observed for **OBP1** ^**+**^, **OBP2** ^**+**^, and **OBP3** ^**+**^ cannot be readily rationalized, as the crystallization process involves^56^ a complex interplay among the cations, anions, and solvent molecules. We propose that the crystallization of **OBP3·TCNE** ^**•**^, **OBP2·PCA**, and **OBP2·TCE** is driven by a combination of efficient space filling through shape matching (the principle of close packing) and the maximization of favorable noncovalent interactions.^57^

Single crystals of **A1** were also obtained via slow diffusion of MeOH into a CH_2_Cl_2_ solution. The observed bond lengths are consistent^51^ with the quinoidal-type resonance contributor (Figure S6). The crystal structure features a slipped 1D linear π-stacks motif (), further highlighting the strong propensity of **OBP**-based systems to assemble into linear π-stacks. This assembly behavior underscores their potential for applications in magnetic and conductive materials.

### Magnetic Properties of the Charge-Transfer Salt **OBP3·TCNE^•^**

The open-shell nature of **OBP3·TCNE** ^**•**^ prompted us to examine its magnetic response. The solid-state EPR spectrum of crystalline **OBP3·TCNE** ^**•**^ exhibits a carbon-centered π-radical signal (*g* = 2.0034; Figure 4a; Table S6), consistent with the TCNE^•–^ species^58^ identified crystallographically. DFT calculations reveal partial delocalization of the unpaired electron from TCNE^•–^ onto the neighboring **OBP3** ^**+**^ scaffold (Figure 4b,c and Figures S19 and S27), which likely contributes to the observed broad EPR line widths and the absence of resolved ^14^N hyperfine coupling.^59^

**Figure 4:**
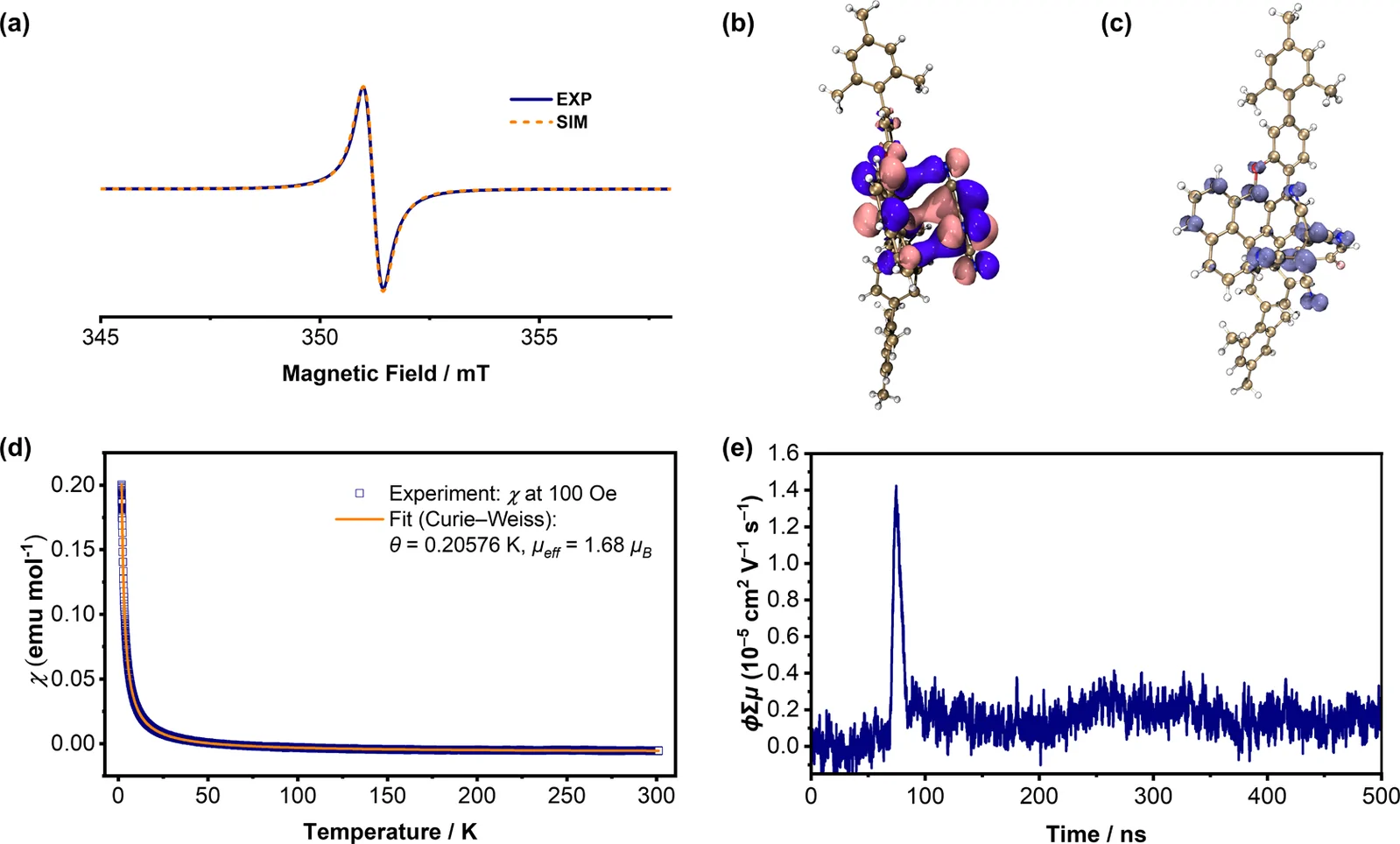
(a) Solid-state EPR spectrum of crystalline **OBP3·TCNE** ^**•**^ at room temperature. Representative (b) SOMO and (c) spin density maps of **OBP3·TCNE** ^**•**^ from single-point DFT calculations (UPBE0/def2-SVP) on the crystallographic geometry (isovalues = 0.02 and 0.005, respectively). (d) Temperature-dependent χ of crystalline **OBP3·TCNE** ^**•**^. (e) TRMC kinetic trace of crystalline **OBP3·TCNE** ^**•**^ under 850 nm excitation (1.5 × 10^16^ photons cm^–2^), normalized to incident photon flux and corrected for illuminated area, assuming unity absorption.

Magnetic susceptibility measurement further elucidates the intrinsic spin state of crystalline **OBP3·TCNE** ^**•**^. Fitting of the magnetic susceptibility (χ) vs *T* data in the temperature range of 1.7–300 K affords an effective magnetic moment (μ_*eff*_) of 1.68 μ_*B*_ per formula unit (Figure 4d), indicative of predominant S = 1/2 character and in reasonably good agreement with the theoretical value of 1.73 μ_*B*_ for an isolated spin.^60^ The corresponding Brillouin fitting analysis further supports the spin-state assignment (Figure S14). Notably, no pronounced low-temperature suppression of *χT* is observed down to 1.7 K (Figure S13), indicating the absence of strong long-range antiferromagnetic coupling^61^ and distinguishing^62–64^ this salt from previously reported TCNE^•–^ systems, in which π-dimerization of TCNE^•–^ typically drives strong spin-pairing interactions. This behavior might be attributed to the alternating ion-pair stacking arrangement, which inhibits^65^ intermolecular antiferromagnetic exchange among TCNE^•–^ units, despite the presence of weak electronic communication.

Upon prolonged exposure to air, the EPR signal of crystalline **OBP3·TCNE** ^**•**^ gradually attenuated over one month (Figure S12a), accompanied by a marked loss of crystallinity in the powder X-ray diffraction (PXRD) patterns (Figure S12b), indicating slow degradation under ambient conditions. This interpretation is further supported by the decrease in μ_*eff*_ to 0.85 μ_*B*_ observed for a fresh sample after 18 h of air exposure (Figure S13). Nevertheless, this extended degradation time scale is consistent with the air-persistent character of **OBP3·TCNE** ^**•**^, allowing routine handling and characterization under ambient laboratory conditions.

### Charge Carrier Mobility of OBP3·TCNE^•^

The presence of unpaired electrons within the 1D linear π-stack motif in **OBP3·TCNE** ^**•**^ crystals suggests a possible pathway for charge transport, motivating an investigation of solid-state photoconductivity by TRMC spectroscopy. TMRC measurements typically provide^66–68^ the parameter ϕΣμ, where ϕ is the charge carrier generation quantum yield and Σμ is the sum of hole and electron mobilities (Σμ = μ_h_ + μ_h_).

Ground-state measurement of crystalline **OBP3·TCNE** ^**•**^ indicates negligible baseline conductivity, whereas photoexcitation elicits a clear transient response. The transient signal exhibits a maximum ϕΣμ value of 1.4 × 10^–5^ cm^2^ V^–1^ s^–1^ (Figure 4e), which falls within the range commonly reported^69–71^ for π-conjugated molecular materials. Notably, the transient recorded at the resonant frequency (r_0_) exhibits a nonzero component persisting at longer time scales, which may indicate the presence of long-lived charge carriers (Figure S15). Further mechanistic investigations are currently underway to elucidate the origin of this long-lived component. While preliminary, these data suggest that the π-stacked system of alternating closed-shell cation and open-shell anion may offer a platform for exploring photoinduced charge transport in supramolecular materials.

### Optical and Redox Properties of OBP^+^ Cations and Quinoidal Adducts

UV–vis–NIR absorption spectroscopy was conducted to investigate the optical properties of **OBP** ^**+**^ cations. All three **OBP·TFA** salts in CH_2_Cl_2_ exhibit a broad low-energy absorption band (500–700 nm, *ε* = 1.5–1.8 × 10^4^ M^–1^ cm^–1^; Figure 5a), corresponding to a HOMO→LUMO transition according to time-dependent DFT (TD-DFT) calculations (Figure S25a–c; Tables S11–S13). Notably, **OBP2** ^**+**^ displays additional weak shoulders at ca. 430 and 500 nm, attributed to partial excited-state polarization by natural transition orbitals (NTO) analysis, whereas the excitations of **OBP1** ^+^ and **OBP3** ^+^ remain confined to the π-framework (Figure S21). This finding suggests a minor perturbation on the excited-state electronic structure from 4-methoxyphenyl, a weak electron-donating group.

**Figure 5:**
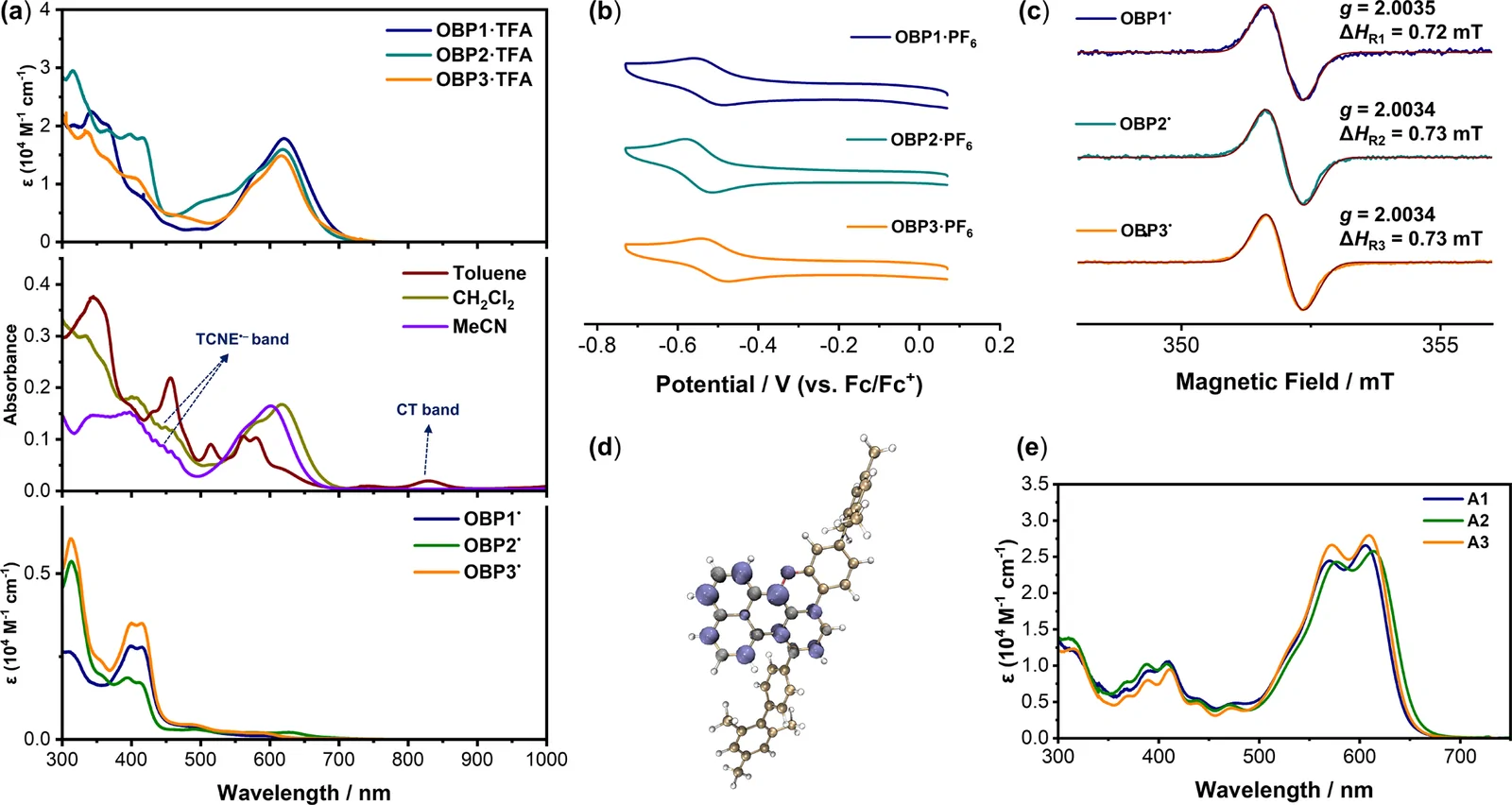
(a) UV–vis–NIR absorption spectra of **OBP1·TFA**–**OBP3·TFA** in CH_2_Cl_2_ (top), solvent-dependent spectra of **OBP3**·**TCNE** ^**•**^ in toluene, CH_2_Cl_2_, and MeCN (middle), and spectra of chemically reduced **OBP1** ^**•**^–**OBP3** ^**•**^ in MeCN (bottom); all spectra were measured at ca. 1 × 10^–5^ M. (b) CV curves of **OBP1·PF**_**6**_–**OBP3·PF**_**6**_ in CH_2_Cl_2_ under N_2_ with 0.1 M tetrabutylammonium hexafluorophosphate (TBAPF_6_) as the supporting electrolyte (scan rate = 100 mV s^–1^), referenced to the Fc/Fc^+^ couple. (c) X-band EPR spectra of **OBP1** ^**•**^ (navy), **OBP2** ^**•**^ (cyan), and **OBP3** ^**•**^ (orange) in MeCN recorded at room temperature, with simulated spectra overlaid in dark red. (d) Spin density map of **OBP1** ^**•**^ computed at the UPBE0/def2-SVP level (isovalue = 0.005). (e) UV–vis absorption spectra of **A1**–**A3** recorded in CH_2_Cl_2_ at ca. 1 × 10^–5^ M.

To probe the electronic interplay between TCNE^•–^ and **OBP3** ^**+**^, UV–vis–NIR absorption spectra were recorded in solvents with varying ion-solvating abilities (Figure 5a). In CH_2_Cl_2_ and MeCN, where the salt is fully dissociated, the spectra exhibit the characteristic bands^72^ of independent **OBP3** ^**+**^ and TCNE^•–^. In contrast, in toluene, which favors tight ion pairing, a charge-transfer band emerges in the NIR region at 827 nm. In comparison, **OBP3·TFA** shows no solvent-dependent behaviors (Figure S7a), displaying only the absorption bands of **OBP3** ^**+**^.

The quinoidal adducts **A1**–**A3** display absorption profiles closely resembling those of the π-cations, featuring well-resolved vibronic progressions at ca. 570 and 610 nm (Figure 5e). NTO analysis indicates a predominantly localized π–π* character (Figure S23). Upon increasing the hexane fraction in CH_2_Cl_2_, the UV–vis spectra of the adducts show suppressed (0,0) vibronic band, an increased (0,1)/(0,0) ratio, and hypsochromically shifted absorption maxima (Figure S11a–c), signatures^73^ consistent with weak H-aggregation. The lack of correlation with empirical solvent polarity parameters further excludes the possibility of solvatochromism (–f).

Cyclic voltammetry (CV) reveals a similar redox behavior for all **OBP** ^**+**^ cations. Each undergoes a reversible one-electron reduction to the corresponding neutral radical (Figure 5b), with nearly identical reduction potentials (*E*_1/2_ = –0.51 to –0.54 V vs Fc/Fc^+^). The formation of radicals **OBP1** ^**•**^–**OBP3** ^**•**^ was monitored using spectroelectrochemical experiments by applying a constant potential (−0.9 V vs Fc/Fc^+^) to the samples. We observed the decrease in absorption of **OBP** ^**+**^ cations and the increase in absorption of a new shoulder band at ca. 400 nm (Figure S8), in agreement with the values obtained from TD-DFT simulations (Figure S25d–f). NTO analysis of all three radicals reveals a subtle spatial separation, with hole density localized on the phenalenyl moiety and particle density extending toward the oxygen-bearing periphery (Figure S22), suggesting a minor charge-transfer contribution.

Scan-rate-dependent CV confirms that the radicals are persistent under inert conditions but susceptible to oxygen-induced decomposition (Figure S9). Under ambient air, the anodic return waves of **OBP1** ^**+**^–**OBP3** ^**+**^ progressively diminishes at lower scan rates, whereas reversibility is fully maintained under N_2_.

Neutral radicals **OBP1** ^**•**^–**OBP3** ^**•**^, generated by reducing corresponding **OBP** ^**+**^ cations with Cu–Sn alloy powder, display UV–vis profiles (Figure 5a) identical to those obtained electrochemically. EPR spectroscopy reveals a single isotropic signal for each species (Figure 5c), with nearly identical *g*-factors (*g* = 2.0034–2.0035) and line widths (Δ*H* = 0.72–0.73 mT), and no resolved hyperfine coupling. These features are consistent with extensive spin delocalization, as commonly observed^74–80^ in π-conjugated organic radicals. DFT-calculated spin density maps confirm predominant localization of the unpaired electron on the **OBP** ^**•**^ core irrespective of substituent identity (**OBP1** ^**•**^, Figure 5d; **OBP2** ^**•**^–**OBP3** ^**•**^, Figure S26).

The persistence of **OBP** ^**•**^ radicals was further evaluated by monitoring their UV–vis absorption spectra over time in dilute MeCN solutions under ambient conditions (Figure S10a–c). **OBP1** ^**•**^ and **OBP3** ^**•**^ undergo gradual aerobic oxidation to the corresponding cations, as evidenced by decay of the ca. 400 nm absorption bands accompanied by recovery of characteristic cationic features. In contrast, **OBP2** ^**•**^ displays lower oxidation, indicating enhanced radical persistence. The greater persistence of **OBP2** ^**•**^ is likely attributed to a push–pull electronic effect^81^ from the electron-donating 4-methoxyphenyl group and the electron withdrawing oxonium moiety. Nevertheless, consistent with this solution-phase behavior, attempts to isolate these radicals as solids under inert atmosphere were unsuccessful, as solvent removal consistently regenerated the corresponding cation (Figure S10d–f). We attribute this primarily to adventitious trace O_2_ during sample handling, although a concentration-driven disproportionation pathway cannot be excluded.

In comparison, all three adducts exhibit (Figure S7b; Table S5) irreversible oxidation (+0.62, + 0.48, and +0.73 V for **A1**–**A3**, respectively) and reduction (ca. −1.1 V).

### Assessment of Aromaticity

To evaluate the effect of redox state on the electronic structures of phenalenyl-derived π-skeleton, we carried out a comprehensive aromaticity analysis using nucleus-independent chemical shift zz component (NICS_zz),^82^ two-dimensional isochemical shielding surface (2D-ICSS),^83^ anisotropy of the induced current density (ACID),^84^ and localized orbital locator for π electrons (LOL-π).^85^ The computed results for rings A–F, using **OBP1** ^**+**^ as the model system, are shown in Figure 6. In the closed-shell cationic state (Figure 6a,d,g), rings A, B, E, and F exhibit pronounced aromaticity, as indicated by strongly negative NICS(1)_zz values (−19.16 to –24.72 ppm), diatropic ring currents in the ACID plots, and shielding regions in the 2D-ICSS maps. Ring D only displays marginal aromatic character (−1.82 ppm), whereas ring C shows a weakly positive NICS(1)_zz value (3.61 ppm), consistent with deshielding features in the 2D-ICSS maps. The delocalization pattern across rings A and B resembles that of phenalenyl cation. In contrast, the weakly positive NICS(1)_zz value of ring C differs from that of phenalenyl cation, likely due to the contributions from ring currents of the neighboring rings A, B and F. Upon reduction to the open-shell neutral radical (Figure 6b,e,h), the NICS(1)_zz value of ring C decreases from +3.61 to –4.50 ppm, while that of ring D increases sharply, becoming moderately antiaromatic (12.50 ppm) and displaying paratropic currents in the ACID plots. The peripheral rings A, B, E, and F largely retain their diatropic character, albeit with slightly reduced aromaticity. Although this inversion is clearly captured by the magnetic descriptors, LOL-π analysis reveals that the spatial distribution of π electrons remains largely preserved upon reduction (Figure S24a–b). This finding suggests that the unpaired electron predominantly occupies a nonbonding molecular orbital (NBMO), consistent^27,28^ with the established electronic structure of phenalenyl radicals.

**Figure 6:**
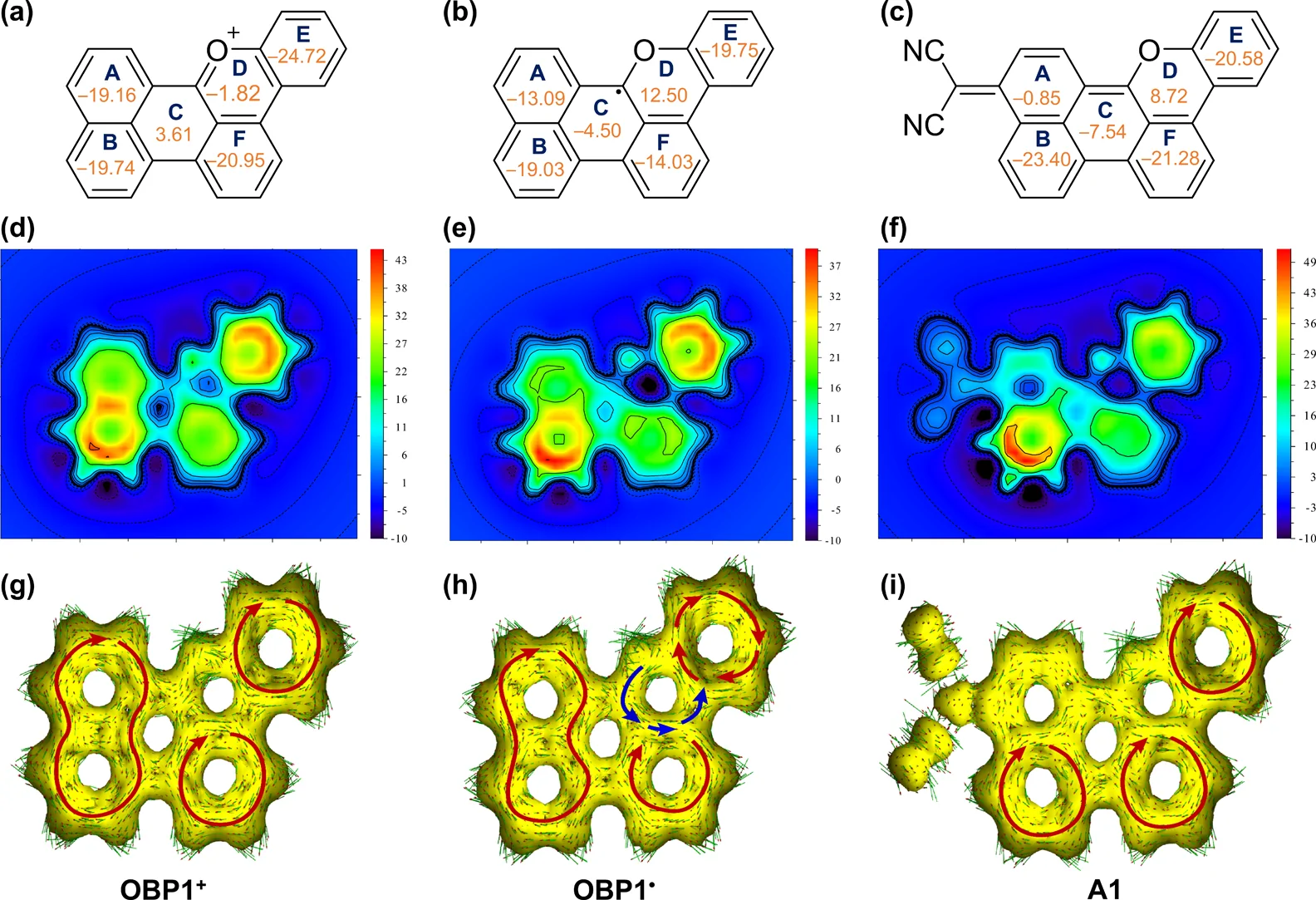
Aromaticity analyses of **OBP1** ^**+**^, **OBP1** ^**•**^, and **A1**. (a–c) NICS(1)_zz values; for nonplanar π-frameworks, values are averaged from NICS­(+1)_zz and NICS(−1)_zz. (d–f) 2D-ICSS maps. (g–i) ACID plots. Calculations were performed on simplified models with peripheral substituents replaced by hydrogen atoms for clarity.

We further analyzed the aromaticity of the adducts using **A1** as a representative model (Figure 6c,f,i). The quinoidal character is reflected by the nearly nonaromatic ring A (−0.85 ppm), weakly aromatic ring C (−7.54 ppm), and weakly antiaromatic pyran ring D (8.72 ppm). These features are consistent with the pronounced π-localization along the quinoidal backbone in LOL-π analysis (Figure S24c), a pattern that is also observed in TCNQ-like quinoidal systems.

## Conclusion

In summary, we have demonstrated a versatile photochemical synthetic strategy that can provide access to a rich library of phenalenyl-derived cations based on **OBP** ^**+**^ scaffold. The synthesis is operationally simple and should be readily adaptable for the preparation of more complex cationic π-systems and radicaloids. Worthy of noting, the crystal structures of **OBP3·TCNE** ^**•**^, **OBP2·PCA**, **OBP2·TCE** and adduct **A1** all display 1D linear π-stacks of conjugated species, a recurring packing motif that may hold promise for future charge and spin transport studies. In particular, **OBP3·TCNE** ^**•**^, a rare example of ambient-persistent organic radical salt, features 1D alternating stacks of closed-shell π-cation and open-shell radical anion and exhibits classic S = 1/2 Curie paramagnetism with negligible antiferromagnetic exchange interactions. Consistent with DFT calculations indicating partial spin and electron delocalization between **OBP3** ^**+**^ and TCNE^**•–**^, preliminary photoconductivity measurements reveal a maximum ϕΣμ value of 1.4 × 10^**–**5^ cm^2^ V^**–**1^ s^**–**1^ for **OBP3·TCNE** ^**•**^, along with a long-lived transient response whose origin remains under investigation. Additionally, **OBP1** ^**+**^–**OBP3** ^**+**^ undergo reversible single-electron reduction, both electrochemically and chemically, to the neutral open-shell radical states, demonstrating potential as redox-switchable materials. Furthermore, reactions at the 4-position of the **OBP** π-scaffolds, which led to the formation of the side products **A1**–**A3**, may provide a strategy for installing stabilizing groups for stable radicals and for constructing larger π-systems. Overall, this work establishes a platform for the design of cationic π-conjugated molecules and open-shell organic crystals, as well as for investigating the interplay among structure, charge and spin in functional π-conjugated materials.

## Supplementary Material











## Data Availability

CCDC 2545972–2545975 contain the supplementary crystallographic data for this paper. These data can be obtained free of charge from the Cambridge Crystallographic Data Centre via www.ccdc.cam.ac.uk/data_request/cif.

## Notes

Crystallographic data (CIF)
Crystallographic data (CIF)
Crystallographic data (CIF)
Crystallographic data (CIF)
Additional text, figures, and tables as described in the text (PDF)
