# Supplementary material for: Oxonium-Embedded Benzo[b]perylene: A Redox-Switchable Closed-Shell π‑Cation, and Its Paramagnetic Charge-Transfer Salt with TCNE•–
Source: ACS Cent Sci. 2026 Aug 31;12(9):1477–88. doi: 10.1021/acscentsci.6c01018 (PMC13614080; doi:10.1021/acscentsci.6c01018)
Supplement: Supplementary file 1 [file oc6c01018_si_005.pdf]

Supporting Information

Oxonium-Embedded Benzo[*b*]perylene: a Redox-Switchable Closed-Shell *π*-Cation, and Its Paramagnetic Charge-Transfer Salt with $TCNE^{•-}$

Chao Duan,[1] Yuan Xue,[1] Daniel B. Straus,[2] Alexander Mariscal,[3] Justin L. Ratkovec,[4] Navamoney Arulsamy,[5] Jianping Zhao,[6] Mei Wang,[7] Obadiah G. Reid,[8,9] Lukasz Wojtas,[3] Xuesong Li,[5] Wenqi Liu,[3] and Penghao Li[1,*]

[1] Department of Chemistry & Biochemistry, The University of Mississippi, University, MS 38677, United States

[2] Department of Chemistry, Tulane University, New Orleans, LA 70118, United States

[3] Department of Chemistry, University of South Florida, Tampa, FL 33620, United States

[4] Department of Chemistry, University of Colorado Boulder, Boulder CO 80309, United States

[5] Department of Chemistry, University of Wyoming, Laramie, WY 82071, United States

[6] National Center for Natural Products Research, School of Pharmacy, The University of Mississippi, University, MS 38677, United States

[7] Natural Products Utilization Research Unit, Agricultural Research Service, United States Department of Agriculture, University, MS 38677, United States

[8] Chemistry and Nanoscience Center, National Laboratory of the Rockies, Golden, CO 80401, United States

[9] Renewable and Sustainable Energy Institute, University of Colorado Boulder, Boulder, CO 80309, United States

*Corresponding Author / Email: penghao@olemiss.edu

Table of Contents

1. **General experimental protocols** 3
2. **Synthetic Procedures** 4
3. **Structural, Electronic, and Magnetic Property Studies** 13
   - 3.1 Electronic Origin of Diels–Alder Reaction Selectivity 13
   - 3.2 Photochemical Reaction Mechanism Studies 14
   - 3.3 Electronic Structure Analysis 19
   - 3.4 Electrochemical and Optical Properties 20
   - 3.5 Magnetic and Electrical Conductivity Analysis 25
4. **X-ray crystallography** 28
5. **Computations** 34
6. **NMR spectra of synthesized compounds** 48
7. **References** 64
8. **Cartesian coordinates** 66

1. General experimental protocols

All reagents were purchased from commercial suppliers and used without further purification. Thin layer chromatography (TLC) was performed on silica gel F254 (E. Merck). Column chromatography was carried out on silica gel 60F (Merck 9385, 0.040–0.063 mm). ^{1}H NMR, ^{13}C NMR and 2D NMR spectra were measured on Agilent 500 MHz and Bruker 300 or 400 MHz spectrometers. Spectra were analyzed with MestraNova software. Chemical shif ts (δ) are reported in ppm and referenced to residual solvent signals: $CDCl_3$ (δ_H = 7.26, δ_C = 77.16), CD_2Cl_2 (δ_H = 5.32, δ_C = 53.84), and $C_2D_2Cl_4$ (δ_H = 5.96).[1] ^{1}H NMR Data are represented as follows: chemical shift, multiplicity (s = singlet, d = doublet, t = triplet, m = multiplet), coupling constants in Hertz (Hz), and integration area of the signal. High-resolution mass spectra (HRMS) were acquired using an Agilent 6560 LC-IM-QTOF mass spectrometer and a Bruker ultrafleXtreme MALDI–TOF/TOF mass spectrometer. UV/vis/NIR absorption spectra were recorded in a quartz cuvette with an optical path-length of 10 mm at room temperature on a Jasco V-730 spectrophotometer. Cyclic voltammetry (CV) measurements were performed using a CHI760E potentiostat in CH_2Cl_2 using tetrabutylammonium hexafluorophosphate ($TBAPF_6$; 0.1 M) as the supporting electrolyte at scan rates ranging of 10–100 mV s^{-1}. A standard three-electrode cell consisting of a platinum disk working electrode, a platinum wire counter electrode, and a silver wire pseudo-reference electrode (calibrated vs Fc/Fc^+) was used. The solution was purged with nitrogen and maintained under a nitrogen atmosphere during measurements. EPR spectra were recorded on a Bruker EMX spectrometer operating at X-band (ca. 9.5 GHz), equipped with an ER041 XG microwave bridge and ER4119HS/ER4116DM resonators, with measurements conducted at room temperature using an Oxford liquid nitrogen/helium cryostat. The simulations were performed in Matlab using Easyspin. Magnetic susceptibility measurement was performed on a Quantum Design PPMS (Dynacool) using a sealed sample of **OBP3·TCNE$^{\bullet}$** (4.9 mg) under argon. PXRD spectra were recorded on an ARL EQUINOX 100 X-ray Diffractometer.

2. Synthetic Procedures

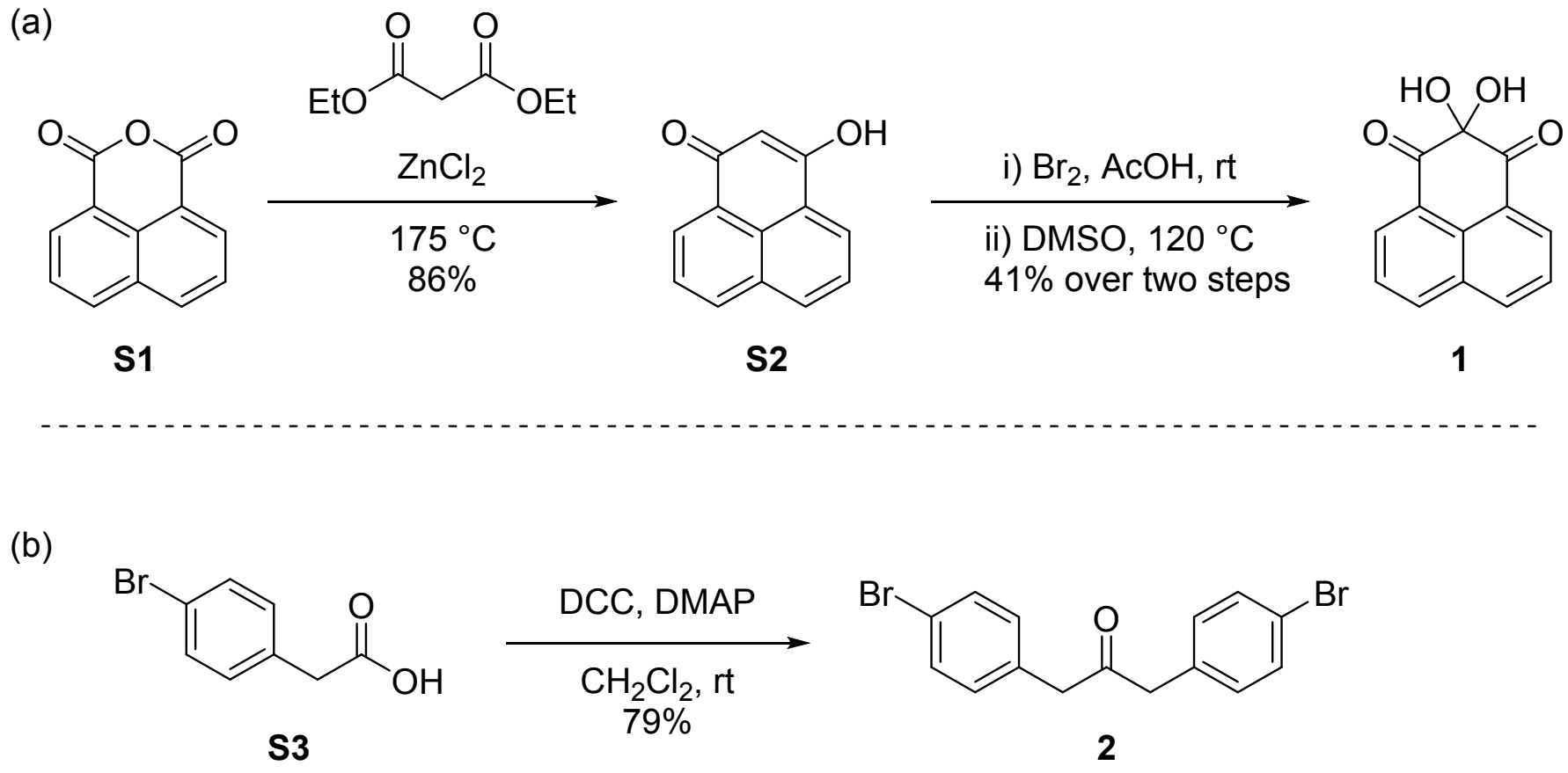

Scheme S1. Synthetic routes of building blocks **1** and **2**.

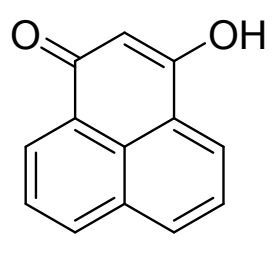

S2

Compound S2: Compound **S1** (15.8 g, 80 mmol, 1.0 equiv) was added to a suspension of anhydrous zinc chloride (32.7 g, 240 mmol, 3.0 equiv) in diethyl malonate (100 mL, 106 g, 662.5 mmol, 8.3 equiv). The reaction mixture was stirred at 175 °C for 14 h. After cooling to room temperature, the mixture was diluted with acetone (100 mL). The resulting solid was collected by filtration and dried to afford compound **S2** as a yellow solid (13.5 g, 86% yield). **^{1}H NMR** (400 MHz, DMSO-d_6) δ 8.25 (d, J = 6.9 Hz, 2H), 8.13 (d, J = 8.2 Hz, 2H), 7.66 (t, J = 7.7 Hz, 2H), 5.84 (s, 1H). The ^{1}H NMR data are consistent with those reported in the literature.[2]

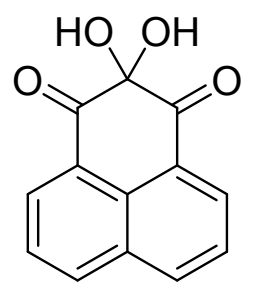

1

Compound 1: To a solution of compound **S2** (13.5 g, 68.8 mmol, 1.0 equiv) in AcOH (100 mL) was added Br_2 (12.5 g, 4.0 mL, 82.6 mmol, 1.2 equiv), and the reaction mixture was stirred at room temperature overnight. The mixture was then added 200 mL water, and the resulting solid was collected by filtration. The crude solid was dissolved in DMSO (100 mL) and heated at 120 °C for 3 h. After cooling slightly, hot water (100 mL) was added, and the mixture was filtered to remove insoluble materials. Upon cooling the filtrate to room temperature, the product crystallized from the mother liquor and was collected by filtration to afford compound **1** as orange needle-like crystals (6.4 g, 41% yield over two steps). **^{1}H NMR** (400 MHz, Acetonitrile-d_3) δ 8.39 (dd, J = 7.3, 1.2 Hz, 2H), 8.36 (dd, J = 8.3, 1.2 Hz,

2H), 7.81 (dd, J = 8.3, 7.2 Hz, 2H), 5.41 (s, 2H). **^{13}C NMR** (101 MHz, Acetonitrile-d_3) δ 192.6, 135.9, 133.8, 132.1, 129.9, 128.1, 127.7, 93.2. **HRMS** (ESI) (m/z): $[M+Na]^+$ Cacld. for $C_{13}H_8NaO_4$: 251.0315; Found: 251.0315.

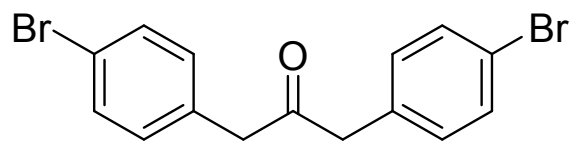

2

Compound 2: To a solution of *N*,*N'*-dicyclohexylcarbodiimide (DCC) (20.6 g, 100.0 mmol, 1.0 equiv) and 4-(dimethylamino)pyridine (DMAP) (6.1 g, 50.0 mmol, 0.5 equiv) in CH_2Cl_2 (150 mL) was added a solution of compound **S3** (21.5 g, 100.0 mmol, 1.0 equiv) in CH_2Cl_2 (300 mL), and the reaction mixture was stirred at room temperature for 24 h. The mixture was filtered, and the filtrate was concentrated in vacuo. The crude product was purified by column chromatography on silica gel, eluting with hexanes/EtOAc (v/v = 9:1), to afford compound **2** as a pale-yellow solid (14.6 g, 79% yield). **^{1}H NMR** (400 MHz, Chloroform-*d*) δ 7.47 – 7.41 (m, 4H), 7.04 – 6.98 (m, 4H), 3.68 (s, 4H). The ^{1}H NMR data are consistent with those reported in the literature.[3]

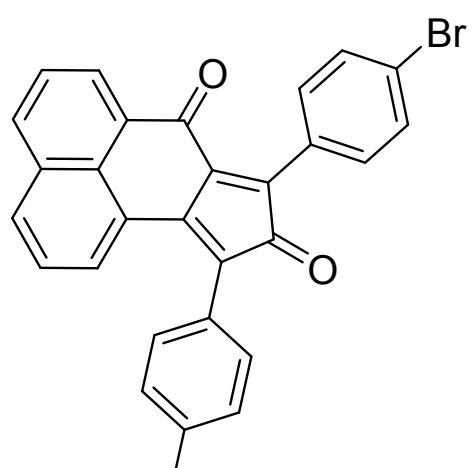

3

Compound 3: To a degassed solution of compounds **1** (2.0 g, 8.9 mmol, 1.0 equiv) and **2** (3.3 g, 8.9 mmol, 1.0 equiv) in EtOH (200 mL) was added piperidine (5.2 g, 6.1 mL, 62.2 mmol, 7.0 equiv) under a nitrogen atmosphere, and the reaction mixture was stirred at 60 °C for 1 h. After cooling to room temperature, the resulting solid was collected by filtration and used directly in the next step without further purification. The crude solid was dissolved in degassed Ac_2O (15 mL) under nitrogen and refluxed overnight. After cooling to room temperature, the mixture was filtered and the solid was washed with acetone to afford compound **3** as a black solid (2.1 g, 43% yield over two steps). **^{1}H NMR** (400 MHz, Chloroform-*d*) δ 8.50 (dd, J = 7.3, 1.3 Hz, 1H), 8.16 (dd, J = 8.2, 1.3 Hz, 1H), 8.10 (dd, J = 7.6, 1.1 Hz, 1H), 8.01 (d, J = 8.1 Hz, 1H), 7.72 (t, J = 7.8 Hz, 1H), 7.68 – 7.59 (m, 6H), 7.44 (t, J = 7.9 Hz, 1H), 7.33 (d, J = 8.4 Hz, 2H). ^{13}C NMR spectra could not be obtained due to the poor solubility. **HRMS** (ESI) (m/z): $[M+H]^+$ Cacld. for $C_{28}H_{15}Br_2O_2$: 540.9433; Found: 540.9427.

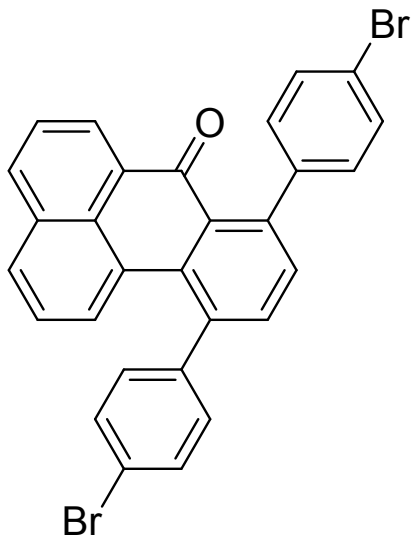

4a

Compound 4a: To a solution of compound **3** (325.3 mg, 0.60 mmol, 1.0 equiv) in degassed *p*-xylene (5 mL) was added 2,5-norbornadiene (122 µL, 110.6 mg, 1.20 mmol, 2.0 equiv) under a nitrogen atmosphere, and the reaction mixture was stirred at 130 °C for 6 h. After cooling to room temperature, the solvent was removed in vacuo. The crude product was purified by column chromatography on silica gel, eluting with hexanes/CH_2Cl_2 (v/v = 1:1), to afford compound **4a** as a pale-yellow solid (298.2 mg, 92% yield). **^{1}H NMR** (400 MHz, Chloroform-*d*) δ 8.53 (dd, *J* = 7.4, 1.3 Hz, 1H), 8.17 (dd, *J* = 8.1, 1.4 Hz, 1H), 7.86 (d, *J* = 8.1 Hz, 1H), 7.72 (t, *J* = 7.7 Hz, 1H), 7.66 (dd, *J* = 7.7, 1.0 Hz, 1H), 7.61 – 7.52 (m, 5H), 7.30 (t, *J* = 8.3 Hz, 3H), 7.27 – 7.22 (m, 3H). **^{13}C NMR** (101 MHz, Chloroform-*d*) δ 184.1, 143.9, 143.0, 142.9, 139.7, 137.1, 136.0, 135.0, 132.8, 132.6, 131.51, 131.46, 131.28, 131.25, 131.0, 129.8, 129.7, 129.2, 128.3, 126.8, 126.6, 125.8, 121.9, 121.1. **HRMS** (DART) (m/z): $[M+H]^+$ Cacld. for $C_{29}H_{17}Br_2O$: 538.9641; Found: 538.9663.

General Procedure A: the Synthesis of Compounds 4b–c

To a solution of compound **3** (1.0 equiv) in diphenyl ether (Ph_2O) was added the corresponding aryl alkyne (1.5 equiv) under a nitrogen atmosphere. The reaction mixture was heated at 240 °C for 2 h. After cooling to room temperature, the mixture was diluted with CH_2Cl_2, followed by addition of MeOH. The resulting solid was collected by filtration and washed with hexanes to afford the corresponding benzannulated products **4b–c**.

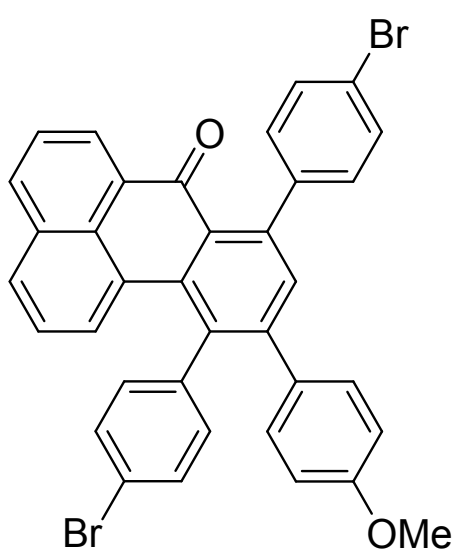

4b

Compound 4b: Prepared according to the **general procedure A** from compound **3** (325.3 mg, 0.60 mmol) and 4-ethynylanisole (118.9 mg, 0.90 mmol) to afford compound **4b** as a pale-yellow solid (314.1 mg, 82% yield). **^{1}H NMR** (300 MHz, Chloroform-*d*) δ 8.54 (dd, *J* = 7.3, 1.3 Hz, 1H), 8.17 (d, *J* = 7.9 Hz, 1H), 7.82 (d, *J* = 8.1 Hz, 1H), 7.71 (t, *J* = 7.7 Hz, 1H), 7.56 (d, *J* = 8.3 Hz, 2H),

7.43 – 7.35 (m, 4H), 7.29 (d, *J* = 8.3 Hz, 2H), 7.17 (t, *J* = 7.9 Hz, 1H), 6.99 – 6.89 (m, 4H), 6.74 (d, *J* = 8.7 Hz, 2H), 3.79 (s, 3H). **^{13}C NMR** (75 MHz, Chloroform-*d*) δ 183.8, 158.7, 146.4, 143.4, 142.8, 140.4, 138.2, 137.4, 135.0, 134.1, 133.2, 132.8, 132.7, 132.2, 131.8, 131.3, 130.7, 130.2, 129.9, 129.44, 129.36, 129.2, 128.6, 127.4, 126.5, 125.7, 121.4, 121.2, 113.5, 55.4. **HRMS** (ESI) (m/z): $[M+H]^+$ Cacld. for $C_{36}H_{23}Br_2O_2$: 645.0059; Found: 645.0060.

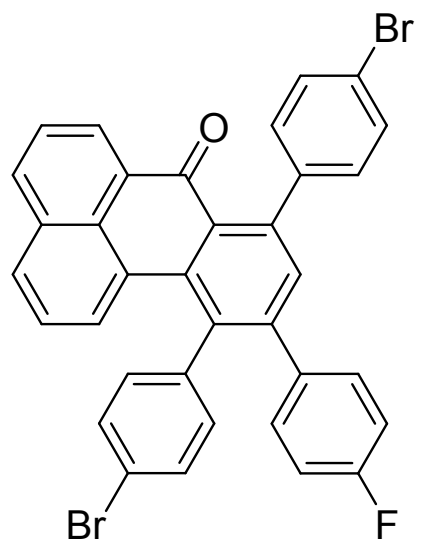

4c

Compound 4c: Prepared according to the **general procedure A** from compound **3** (253.7 mg, 0.60 mmol) and 1-ethynyl-4-fluorobenzene (108.1 mg, 0.90 mmol) to afford compound **4c** as a pale-yellow solid (323.5 mg, 85% yield). **^{1}H NMR** (300 MHz, Chloroform-*d*) δ 8.54 (dd, *J* = 8.1, 1.6 Hz, 1H), 8.18 (d, *J* = 8.0 Hz, 1H), 7.84 (d, *J* = 8.1 Hz, 1H), 7.72 (t, *J* = 7.7 Hz, 1H), 7.57 (d, *J* = 8.3 Hz, 2H), 7.45 – 7.34 (m, 4H), 7.29 (d, *J* = 8.3 Hz, 2H), 7.18 (t, *J* = 7.9 Hz, 1H), 6.98 – 6.89 (m, 5H). **^{13}C NMR** (75 MHz, Chloroform-*d*) δ 183.8, 145.7, 143.5, 142.7, 140.1, 138.2, 137.5, 136.6, 135.1, 133.8, 133.1, 132.2, 131.8, 131.3, 131.1, 131.0, 130.6, 139.9, 129.6, 129.5, 129.1, 127.2, 126.6, 125.7, 121.6, 121.3, 115.3, 115.0. **HRMS** (ESI) (m/z): $[M+H]^+$ Cacld. for $C_{35}H_{20}Br_2FO$: 632.9859; Found: 632.9857.

General Procedure B: Suzuki–Miyaura Cross-Coupling

To a mixture of compounds **4a**–**c** (1.0 equiv), mesitylboronic acid [$MesB(OH)_2$] (2.2 equiv), SPhos Pd G3 (6 mol%), and K_3PO_4 (6.0 equiv) was added degassed THF/H_2O (v/v = 4:1) under a nitrogen atmosphere. The reaction mixture was heated at 70 °C overnight. After cooling to room temperature, the aqueous phase was separated and extracted with CH_2Cl_2 (3 × 10 mL). The combined organic layers were dried over anhydrous Na_2SO_4, filtered, and concentrated in vacuo. The crude residue was purified by column chromatography on silica gel, eluting with hexanes/CH_2Cl_2 (v/v = 2:1), to afford the corresponding products **5a**–**c** as pale-yellow solids.

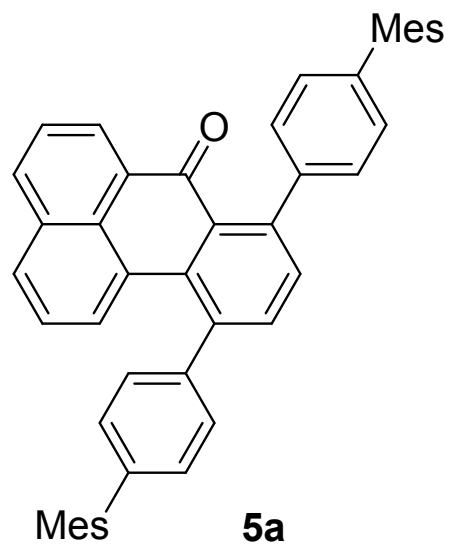

5a

Compound 5a: Prepared according to the **general procedure B** from compound **4a** (270.1 mg, 0.5 mmol) to afford compound **5a** as a pale-yellow solid (281.5 mg, 91% yield). **^{1}H NMR** (400 MHz, Chloroform-*d*) δ 8.57 (d, *J* = 7.3 Hz, 1H), 8.16 (d, *J* = 8.0 Hz, 1H), 7.84 (d, *J* = 8.1 Hz, 1H), 7.71 (ddd, *J* = 10.5, 7.7, 2.1 Hz, 3H), 7.47 (t, *J* = 8.4 Hz, 5H), 7.24 (dd, *J* = 8.1, 1.7 Hz, 4H), 7.15 (t, *J* = 7.9 Hz, 1H), 7.05 – 6.99 (m, 4H), 2.37 (s, 6H), 2.19 (s, 12H). **^{13}C NMR** (101 MHz, Chloroform-*d*) δ 184.3, 144.8, 142.5, 142.3, 140.7, 140.4, 139.24, 139.18, 138.7, 137.2, 137.0, 136.6, 136.5, 136.0, 135.74, 134.67, 132.7, 131.7, 131.6, 131.4, 130.4, 129.50, 129.45, 129.3, 129.0, 128.4, 128.3, 128.2, 127.4, 126.4, 125.3, 21.22, 21.19, 21.0. **HRMS** (DART) (m/z): $[M+H]^+$ Cacld. for $C_{47}H_{39}O$: 619.3001; Found: 619.3011.

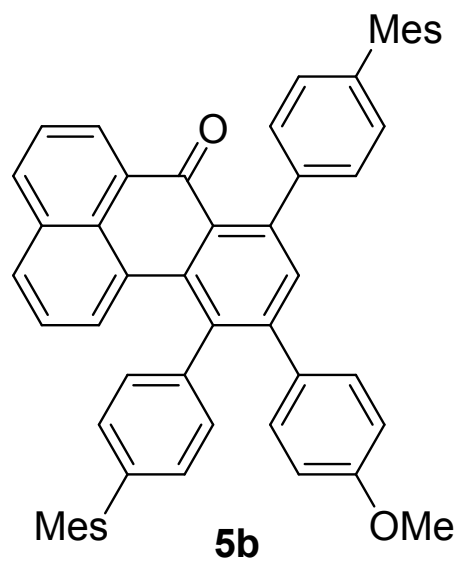

5b

Compound 5b: Prepared according to the **general procedure B** from compound **4b** (258.6 mg, 0.4 mmol) to afford compound **5b** as a pale-yellow solid (275.5 mg, 95% yield). **^{1}H NMR** (300 MHz, Chloroform-*d*) δ 8.58 (dd, *J* = 7.3, 1.3 Hz, 1H), 8.16 (dd, *J* = 8.2, 1.3 Hz, 1H), 7.82 (d, *J* = 8.0 Hz, 1H), 7.71 (t, *J* = 7.8 Hz, 1H), 7.67 (d, *J* = 7.3 Hz, 1H), 7.54 (s, 1H), 7.50 (d, *J* = 8.0 Hz, 2H), 7.23 (d, *J* = 8.1 Hz, 2H), 7.16 (d, *J* = 8.0 Hz, 2H), 7.11 (t, *J* = 7.9 Hz, 1H), 7.07 – 6.95 (m, 8H), 6.73 (d, *J* = 8.7 Hz, 2H), 3.77 (s, 3H), 2.36 (s, 3H), 2.35 (s, 3H), 2.18 (s, 6H), 2.17 (s, 3H), 1.98 (s, 3H). **^{13}C NMR** (75 MHz, Chloroform-*d*) δ 184.1, 158.6, 146.4, 144.5, 142.5, 140.2, 139.8, 139.25, 139.22, 138.8, 138.6, 137.9, 136.8, 136.6, 136.5, 135.9, 135.7, 134.7, 134.4, 133.5, 132.7, 131.8, 131.7, 130.9, 130.3, 129.8, 129.5, 129.3, 129.2, 129.0, 128.3, 128.20, 128.16, 128.0, 126.3, 125.3, 113.2, 55.3, 21.22, 21.20, 20.8, 20.7. **HRMS** (ESI) (m/z): $[M+H]^+$ Cacld. for $C_{54}H_{45}O_2$: 725.3414; Found: 725.3411.

Compound 5c: Prepared according to the **general procedure B** from compound **4c**

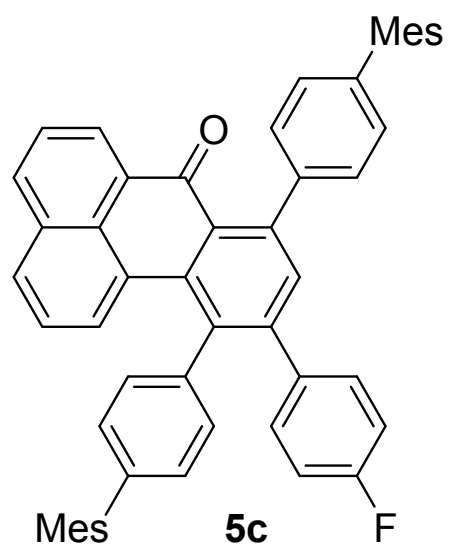

5c

(253.7 mg, 0.4 mmol) to afford compound **5c** as a pale-yellow solid (265.2 mg, 93% yield). **^{1}H NMR** (300 MHz, Chloroform-*d*) δ 8.58 (dd, *J* = 7.4, 1.3 Hz, 1H), 8.16 (dd, *J* = 8.1, 1.3 Hz, 1H), 7.83 (d, *J* = 8.0 Hz, 1H), 7.72 (t, *J* = 7.7 Hz, 1H), 7.66 (d, *J* = 7.7 Hz, 1H), 7.52 (s, 1H), 7.49 (d, *J* = 7.9 Hz, 2H), 7.23 (d, *J* = 7.8 Hz, 2H), 7.17 – 7.07 (m, 5H), 7.05 – 6.95 (m, 6H), 6.88 (t, *J* = 8.7 Hz, 2H), 2.36 (s, 3H), 2.35 (s, 3H), 2.18 (s, 6H), 2.16 (s, 3H), 1.96 (s, 3H). **^{13}C NMR** (75 MHz, Chloroform-*d*) δ 184.1, 145.6, 144.6, 142.3, 140.0, 139.8, 139.4, 139.2, 138.7, 138.6, 138.0, 137.2, 136.9, 136.6, 136.5, 135.9, 135.6, 134.8, 134.2, 132.7, 131.8, 131.6, 131.4, 131.3, 129.9, 129.4, 129.3, 129.1, 128.3, 128.2, 127.8, 126.3, 125.3, 114.8, 114.5, 21.21, 21.20, 20.8, 20.6. **HRMS** (DART) (m/z): $[M+H]^+$ Cacld. for $C_{53}H_{42}FO$: 713.3220; Found: 713.3209.

General Procedure C: TCNE-Mediated Photocyclization

A mixture of compounds **5a**–**c** (1.0 equiv) and TCNE (4.0 equiv) was placed under a nitrogen atmosphere, and degassed, anhydrous toluene was added. The reaction mixture was irradiated with a 395 nm LED at room temperature for 24 h without stirring. Depending on the substrate, the resulting π-cationic salt may precipitate directly and be isolated by filtration; otherwise, the solvent was removed under reduced pressure, and the crude residue was purified by silica gel column chromatography, initially eluting with hexanes/CH_2Cl_2 (v/v = 1:1) to afford the dicyanomethylene adducts **A1**–**A3**, followed by further elution with CH_2Cl_2/MeOH/TFA (v/v/v = 100:1:0.5) to give the π-cationic products **OBP1·TFA**–**OBP3·TFA**.

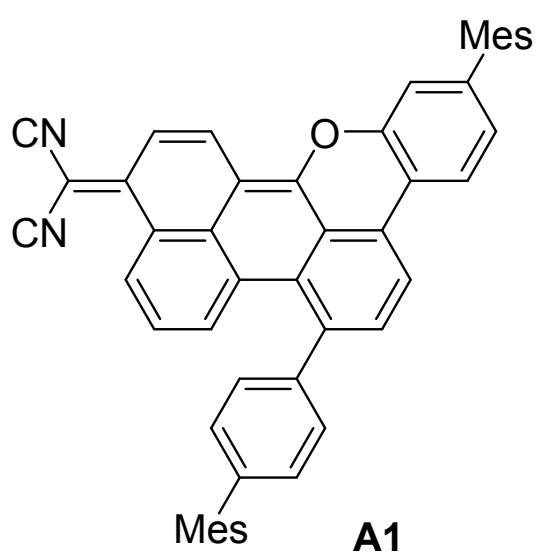

A1

Compound A1: Prepared according to the **general procedure C** from compound **5a** (5.0 mg, 8.1 μmol) using degassed, anhydrous toluene (3 mL) to afford **A1** as a blue solid (0.9 mg, 17% yield). **^{1}H NMR** (500 MHz, Methylene Chloride-d_2) δ 9.12 (d, *J* = 8.0 Hz, 1H), 8.28 (d, *J* = 9.6 Hz, 1H), 8.22 (d, *J* = 7.9 Hz, 1H), 8.19 (d, *J* = 8.1 Hz, 1H), 8.08 (d, *J* = 8.3 Hz, 1H), 7.92 (d, *J* = 7.9 Hz, 1H), 7.52

(d, J = 9.6 Hz, 1H), 7.49 (d, J = 7.9 Hz, 2H), 7.32 – 7.28 (m, 3H), 7.23 – 7.17 (m, 2H), 7.00 (s, 4H), 2.35 (s, 4H), 2.35 (s, 3H), 2.19 (s, 3H), 2.12 (s, 9H). ^{13}C NMR spectra could not be obtained due to the very low solubility. **HRMS** (DART) (m/z): $[M+H]^+$ Cacld. for $C_{50}H_{37}ON_2$: 681.2906; Found: 681.2930.

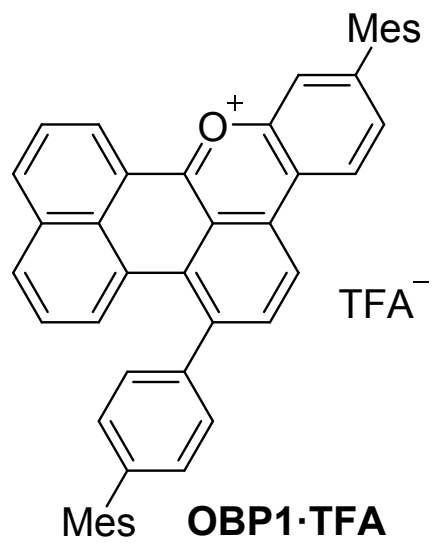

OBP1·TFA

Compound OBP1·TFA: Prepared according to the **general procedure C** from compound **5a** (5.0 mg, 8.1 μmol) using degassed, anhydrous toluene (3 mL) to afford **OBP1·TFA** as a blue solid (2.1 mg, 35% yield). **^{1}H NMR** (400 MHz, $CDCl_3$/TFA-d, 9:1) δ 9.45 (d, J = 7.6 Hz, 1H), 8.79 (d, J = 7.9 Hz, 1H), 8.73 (d, J = 8.1 Hz, 1H), 8.66 (d, J = 8.3 Hz, 1H), 8.55 (dd, J = 8.3, 3.3 Hz, 1H), 8.43 (dd, J = 8.4, 4.4 Hz, 1H), 8.32 (d, J = 8.1 Hz, 1H), 8.19 (t, J = 7.8 Hz, 1H), 7.93 (s, 1H), 7.72 (d, J = 7.7 Hz, 1H), 7.57 (d, J = 7.7 Hz, 2H), 7.51 (t, J = 8.0 Hz, 1H), 7.45 (d, J = 7.5 Hz, 2H), 7.09 – 7.00 (m, 4H), 2.40 (s, 3H), 2.39 (s, 3H), 2.22 (s, 3H), 2.12 (s, 3H), 2.10 (s, 6H). **^{13}C NMR** (101 MHz, $CDCl_3$/TFA-d, 9:1) δ 170.0, 150.7, 149.2, 145.9, 145.7, 143.5, 143.0, 139.4, 138.9, 137.9, 137.8, 137.7, 137.4, 136.9, 136.2, 135.71, 135.67, 135.5, 133.0, 132.3, 132.1, 129.2, 128.9, 128.4, 128.2, 127.4, 126.5, 124.4, 121.3, 121.2, 120.4, 119.7, 119.0, 29.9, 21.1, 20.8, 20.7. **HRMS** (ESI) (m/z): $[M]^+$ Cacld. for $C_{47}H_{37}O$: 617.2839; Found: 617.2836.

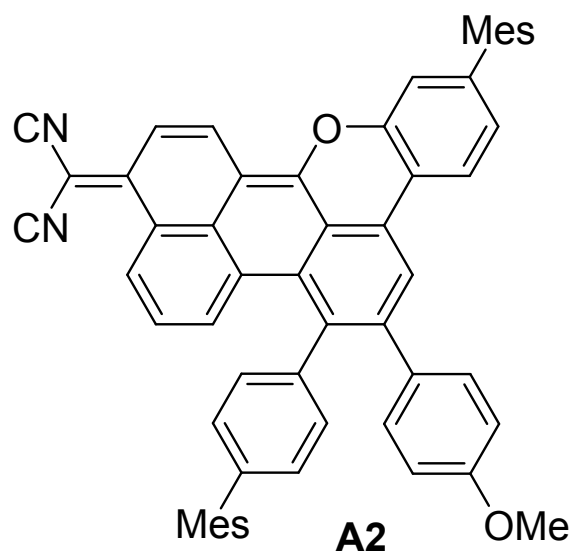

A2

Compound A2: Prepared according to the **general procedure C** from compound **5b** (5.0 mg, 6.9 μmol) using degassed, anhydrous toluene (3 mL) to afford **A2** as a blue solid (1.1 mg, 20% yield). **^{1}H NMR** (500 MHz, Methylene Chloride-d_2) δ 9.07 (d, J = 8.0 Hz, 1H), 8.27 (d, J = 9.6 Hz, 1H), 8.22 (s, 1H), 8.17 (d, J = 8.0 Hz, 1H), 8.03 (d, J = 8.4 Hz, 1H), 7.50 (d, J = 9.6 Hz, 1H), 7.30 (s, 1H), 7.17 – 7.12 (m, 4H), 7.09 – 7.06 (m, 4H), 6.99 (s, 2H), 6.97 (s, 1H), 6.93 (s, 1H), 6.93 (s, 1H), 6.77 (d, J = 8.2 Hz, 2H), 3.77 (s, 3H), 2.34 (s, 3H), 2.32 (s, 3H), 2.15 (s, 3H), 2.10 (s, 6H), 1.95 (s, 3H). **^{13}C NMR** (126 MHz, Methylene Chloride-d_2) δ 159.3, 157.1,

153.6, 151.1, 147.7, 145.4, 141.0, 140.0, 139.4, 137.8, 138.9, 137.6, 137.1, 138.98, 135.96, 135.9, 135.1, 133.8, 133.4, 131.3, 131.2, 130.9, 130.6, 129.7, 129.5, 128.8, 128.7, 128.45, 128.4, 127.4, 125.9, 123.7, 123.71, 122.1, 120.5, 118.6, 118.3, 113.6, 111.9, 32.4, 30.1, 23.1, 21.21, 21.19, 20.8, 14.3. **HRMS** (MALDI-TOF) (m/z): $[M]^+$ Cacld. for $C_{57}H_{42}N_2O_2$: 786.3246; Found: 786.3305.

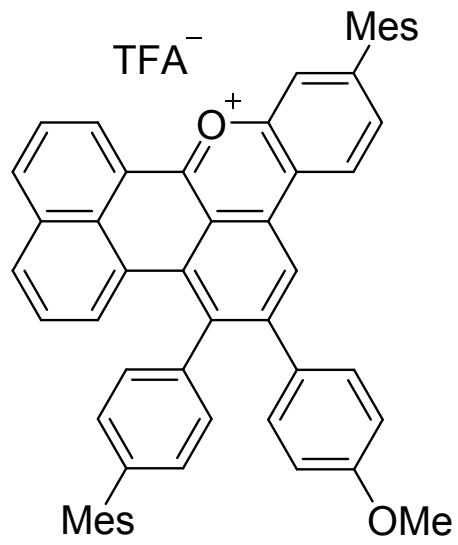

OBP2·TFA

Compound OBP2·TFA: Prepared according to the **general procedure C** from compound **5b** (5.0 mg, 6.9 μmol) using degassed, anhydrous toluene (3 mL). Without isolation of **OBP2·TCE** and **OBP2·PCA**, the solvent was removed under reduced pressure, and the crude residue was directly subjected to column chromatography to afford **OBP2·TFA** as a blue solid (2.1 mg, 37% yield). **^{1}H NMR** (400 MHz, $CDCl_3$/TFA-*d*, 9:1) δ 9.42 (d, *J* = 7.3 Hz, 1H), 8.75 (d, *J* = 7.7 Hz, 1H), 8.69 (s, 1H), 8.63 (d, *J* = 8.2 Hz, 1H), 8.32 (d, *J* = 8.2 Hz, 1H), 8.28 (d, *J* = 7.8 Hz, 1H), 8.18 (t, *J* = 7.2 Hz, 1H), 7.92 (s, 1H), 7.67 (d, *J* = 8.0 Hz, 1H), 7.44 (t, *J* = 7.8 Hz, 1H), 7.26 – 7.23 (m, 4H), 7.17 (d, *J* = 8.3 Hz, 2H), 7.06 (s, 2H), 7.03 (s, 1H), 7.00 (s, 1H), 6.87 (d, *J* = 8.2 Hz, 2H), 3.86 (s, 3H), 2.40 (s, 3H), 2.37 (s, 3H), 2.19 (s, 3H), 2.10 (s, 6H), 1.99 (s, 3H). **^{13}C NMR** (101 MHz, $CDCl_3$/TFA-*d*, 9:1) δ 168.9, 160.0, 157.70, 150.69, 149.0, 145.3, 142.3, 142.0, 138.7, 138.1, 137.8, 137.7, 137.5, 136.5, 135.7, 135.6, 135.4, 135.2, 134.9, 133.0, 131.9, 131.8, 131.7, 130.9, 130.2, 128.94, 128.86, 128.5, 128.4, 128.1, 127.7, 126.9, 124.4, 123.7, 121.2, 119.7, 114.1, 29.9, 21.22, 21.19. 20.77, 20.76, 20.6. **HRMS** (ESI) (m/z): $[M]^+$ Cacld. for $C_{54}H_{43}O_2$: 723.3258; Found: 723.3256.

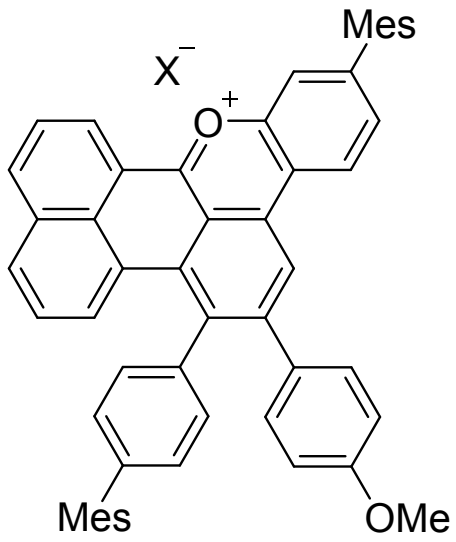

OBP2·TCE and **OBP2·PCA**

Compound OBP2·TCE and **OBP2·PCA**: Prepared according to the **general procedure C** from compound **5b** (5.0 mg, 6.9 μmol) using degassed, anhydrous toluene (3 mL). The reaction afforded a mixture of **OBP2·TCE** and **OBP2·PCA**, which could not be separated by conventional purification methods. The

resulting crystals were collected by filtration to afford a red-brown crystalline solid (1.9 mg, 43% yield). Single crystals suitable for X-ray diffraction were obtained by manual selection of individual crystals corresponding to each salt.

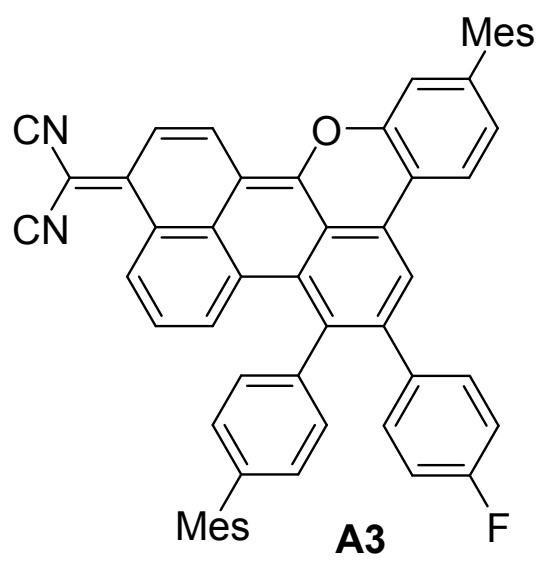

A3

Compound A3: Prepared according to the **general procedure C** from compound **5c** (5.0 mg, 7.0 μmol) using degassed, anhydrous toluene (3 mL) to afford **A3** as a blue solid (1.5 mg, 40% yield). **^{1}H NMR** (500 MHz, $CDCl_2CDCl_2$-d_2) δ 9.08 (d, *J* = 8.1 Hz, 1H), 8.28 (d, *J* = 9.6 Hz, 1H), 8.16 (s, 1H), 8.15 (d, *J* = 8.3 Hz, 1H), 8.02 (d, *J* = 8.4 Hz, 1H), 7.53 (d, *J* = 9.6 Hz, 1H), 7.18 – 7.13 (m, 2H), 7.13 – 7.07 (m, 6H), 6.98 (s, 3H), 6.95 – 6.91 (m, 3H), 2.34 (s, 3H), 2.32 (s, 3H), 2.13 (s, 3H), 2.09 (s, 6H), 1.91 (s, 3H). ^{13}C NMR spectra could not be obtained due to the very low solubility. **HRMS** (MALDI-TOF) (m/z): $[M]^+$ Cacld. for $C_{56}H_{39}FN_2O$: 774.3046; Found: 774.3082.

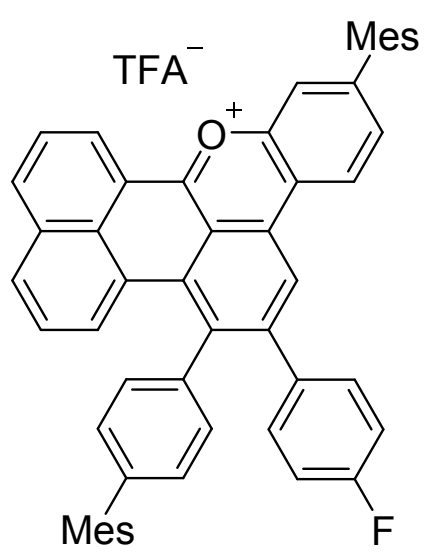

OBP3·TFA

Compound OBP3·TFA: Prepared according to the **general procedure C** from compound **5c** (5.0 mg, 7.0 μmol) using degassed, anhydrous toluene (3 mL). Without isolation of **OBP3·TCNE$^•$**, the solvent was removed under reduced pressure, and the crude residue was directly subjected to column chromatography to afford **OBP3·TFA** as a blue solid (2.5 mg, 43% yield). **^{1}H NMR** (400 MHz, $CDCl_3$/TFA-*d*, 9:1) δ 9.45 (d, *J* = 7.1 Hz, 1H), 8.78 (d, *J* = 7.4 Hz, 1H), 8.68 (s, 1H), 8.63 (d, *J* = 8.1 Hz, 1H), 8.31 (t, *J* = 7.7 Hz, 2H), 8.19 (t, *J* = 7.3 Hz, 1H), 7.94 (s, 1H), 7.68 (d, *J* = 7.3 Hz, 1H), 7.44 (t, *J* = 7.8 Hz, 1H), 7.24 (s, 2H), 7.20 (d, *J* = 10.0 Hz, 2H), 7.06 (s, 2H), 7.04 – 6.98 (m, 4H), 2.40 (s, 3H), 2.37 (s, 3H), 2.18 (s, 3H), 2.10 (s, 6H), 1.96 (s, 3H). **^{13}C NMR** (101 MHz, $CDCl_3$/TFA-*d*, 9:1) δ 169.3, 150.8, 145.8, 142.4, 138.2, 138.0, 137.8, 137.6, 136.9, 135.6, 135.4, 135.2, 135.1, 133.0, 131.9, 131.13, 131.06, 130.1, 128.9, 128.5, 128.4, 128.2, 127.7, 126.8, 124.4, 123.6, 121.2, 119.7, 119.5, 115.9, 115.6, 29.9, 21.2, 20.7, 20.5. **HRMS** (ESI) (m/z): $[M]^+$ Cacld. for $C_{53}H_{40}FO$: 711.3058; Found:

711.3068.

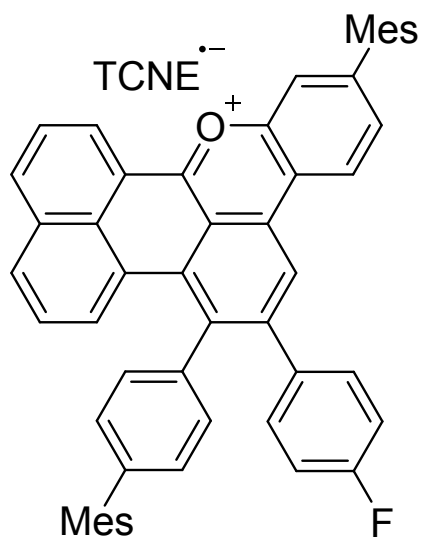

Compound OBP3·TCNE•: Prepared according to the **general procedure C** from compound **5c** (5.0 mg, 7.0 μmol) using degassed, anhydrous toluene (3 mL). The resulting crystals were collected by filtration to afford **OBP3·TCNE•** as a dark-purple crystalline solid (1.9 mg, 32% yield). The structure was confirmed by SCXRD. This crystallization was reproducible over multiple independent attempts.

General Procedure D: DDQ-Mediated Photocyclization

A mixture of compounds **5a**–**c** (1.0 equiv) and DDQ (2.0 equiv) was placed under a nitrogen atmosphere, and degassed, anhydrous toluene was added. The reaction mixture was irradiated with a 395 nm LED at room temperature for 24 h without stirring. After removal of the solvent under reduced pressure, the crude residue was purified by silica gel column chromatography, eluting with CH_2Cl_2/MeOH/TFA (v/v/v = 100:1:0.5), to afford the corresponding π-cationic products **OBP1·TFA**–**OBP3·TFA** in high yields (85–90%), which were identical to those obtained under TCNE-mediated conditions.

3. Structural, Electronic, and Magnetic Property Studies

3.1 Electronic Origin of Diels–Alder Reaction Selectivity

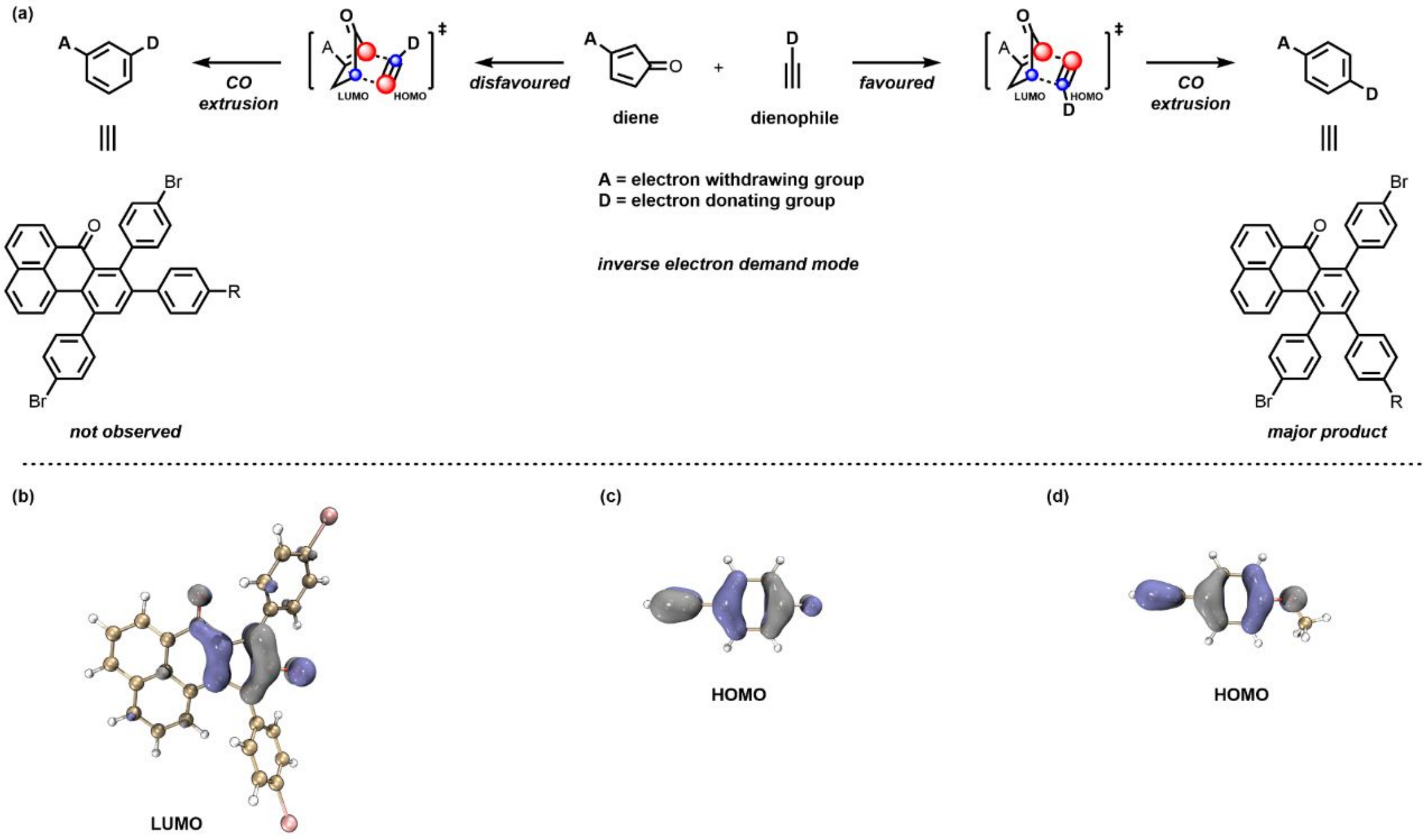

Figure S1. (a) Orbital interaction analysis rationalizing the regioselectivity of the inverse electron-demand Diels–Alder reaction. (b–d) The corresponding frontier molecular orbitals at the PBE0/def2-SVP level: (b) LUMO of cyclopentadienone **3**, HOMO of (c) 1-ethynyl-4-fluorobenzene and (d) 4-ethynylanisole.

3.2 Photochemical Reaction Mechanism Studies

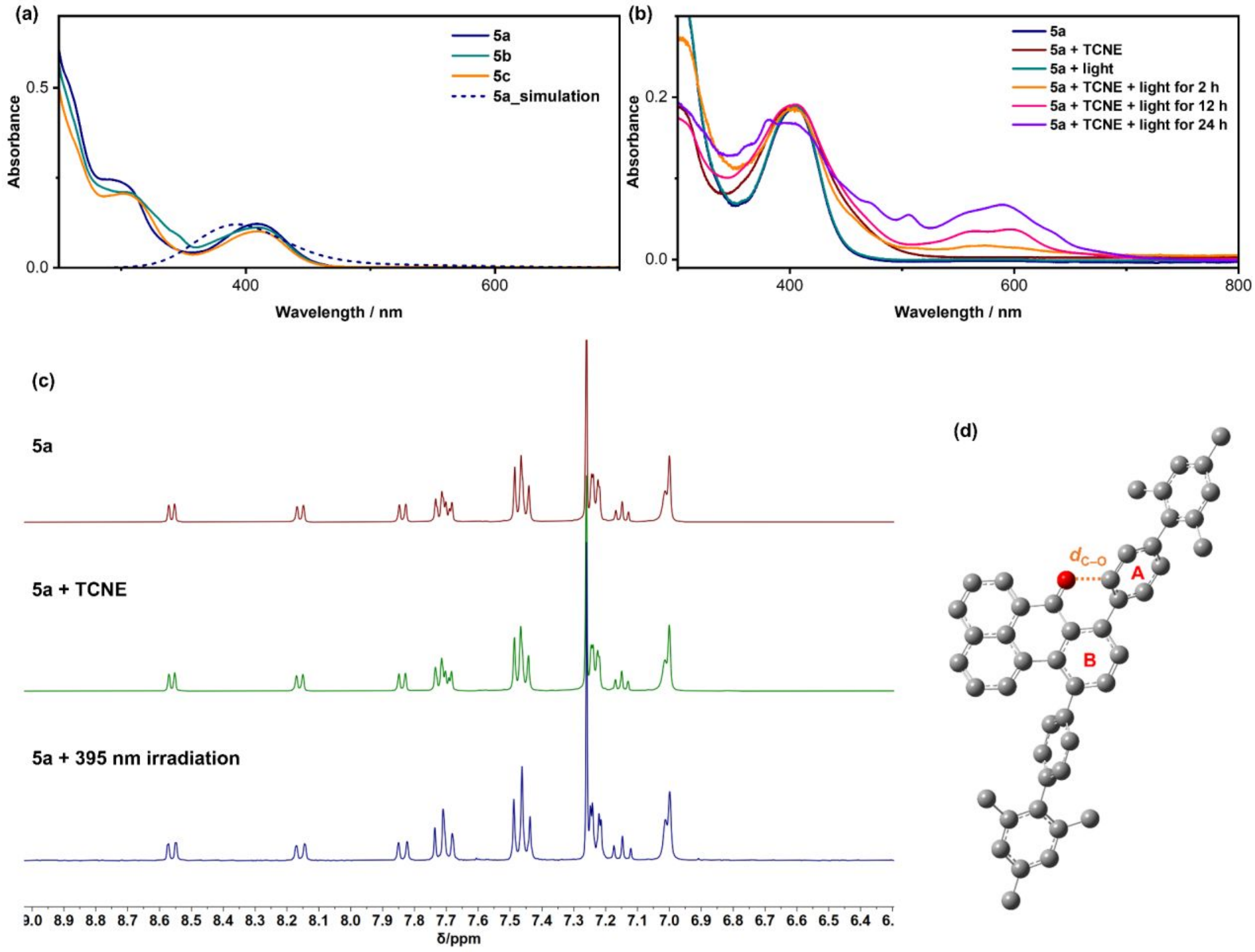

Figure S2. (a) UV–vis absorption spectra of **5a**–**c** in CH_2Cl_2 (1.0×10^{-5} M; solid lines), and the TD-DFT–simulated spectrum of **5a** (dashed line). (b) UV–vis absorption spectra of **5a** in CH_2Cl_2 in the absence and presence of TCNE under dark and light-irradiated conditions, including time-dependent spectral evolution upon irradiation. (c) Partial ^{1}H NMR spectra of **5a** in $CDCl_3$ under three conditions: without TCNE and light, with TCNE in the dark, and under light irradiation without TCNE. All spectra were recorded after 2 h. (d) Definition of rings A and B and the $d_{C–O}$ parameter in **5a**.

Table S1. Calculated C–O distances and dihedral angles along the reaction coordinate of **5a**.

Structure	$d_{C–O}$ / Å	ϕ_{AB} / °
**5a(S$_0$)**	3.03	56.6
**5a(S$_1^{min}$)**	2.93	53.2
**5a(S$_1^{react}$)**	2.35	49.3
**5a(S$_1^{TS}$)**	2.04	36.8
**cyc(S$_1$)**	1.68	32.0
**cyc(S$_0$)**	1.49	8.1

Photo-induced electron-transfer–proton-transfer–radical coupling

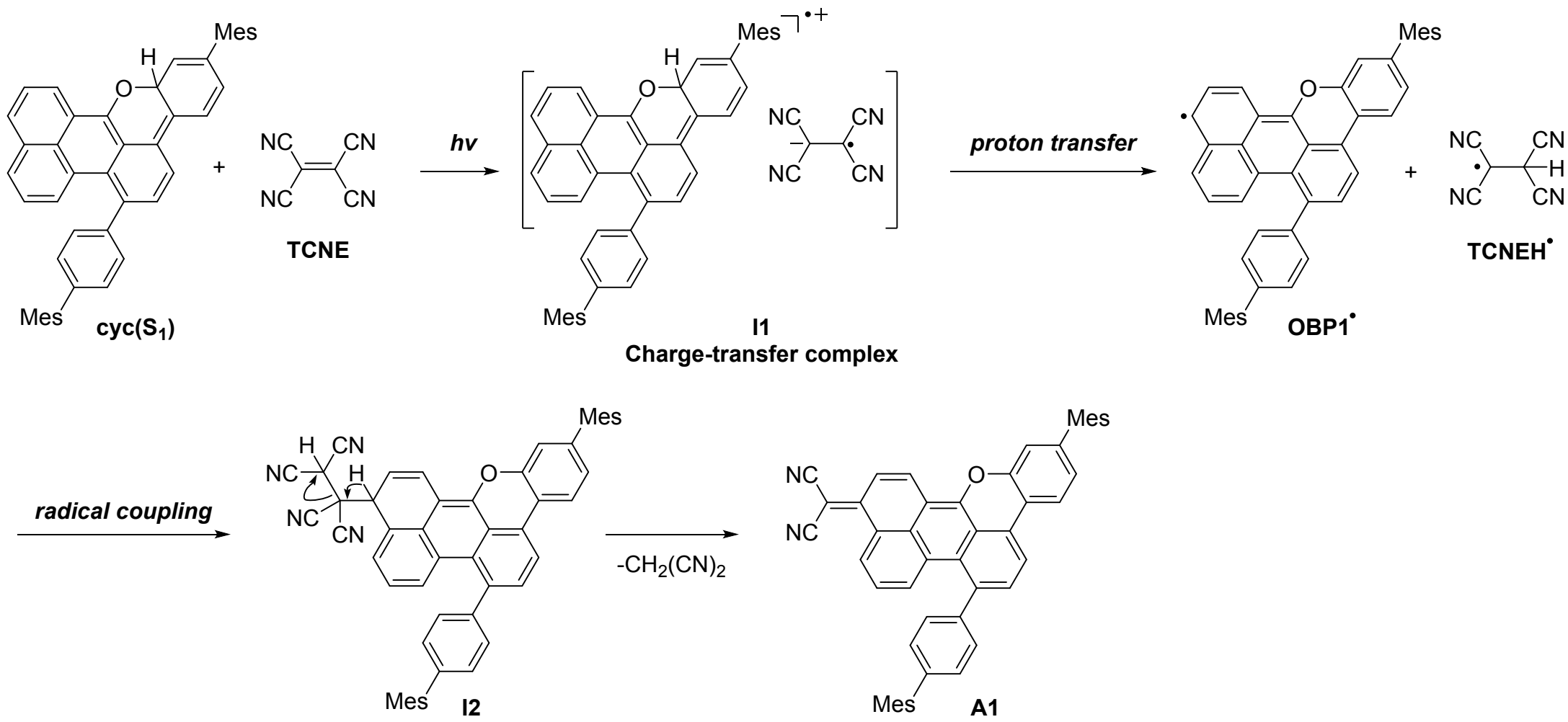

Scheme S2. Proposed reaction mechanism proceeding via an electron-transfer–proton-transfer–radical coupling sequence.

Radical coupling of radical cation and TNCE radical anion

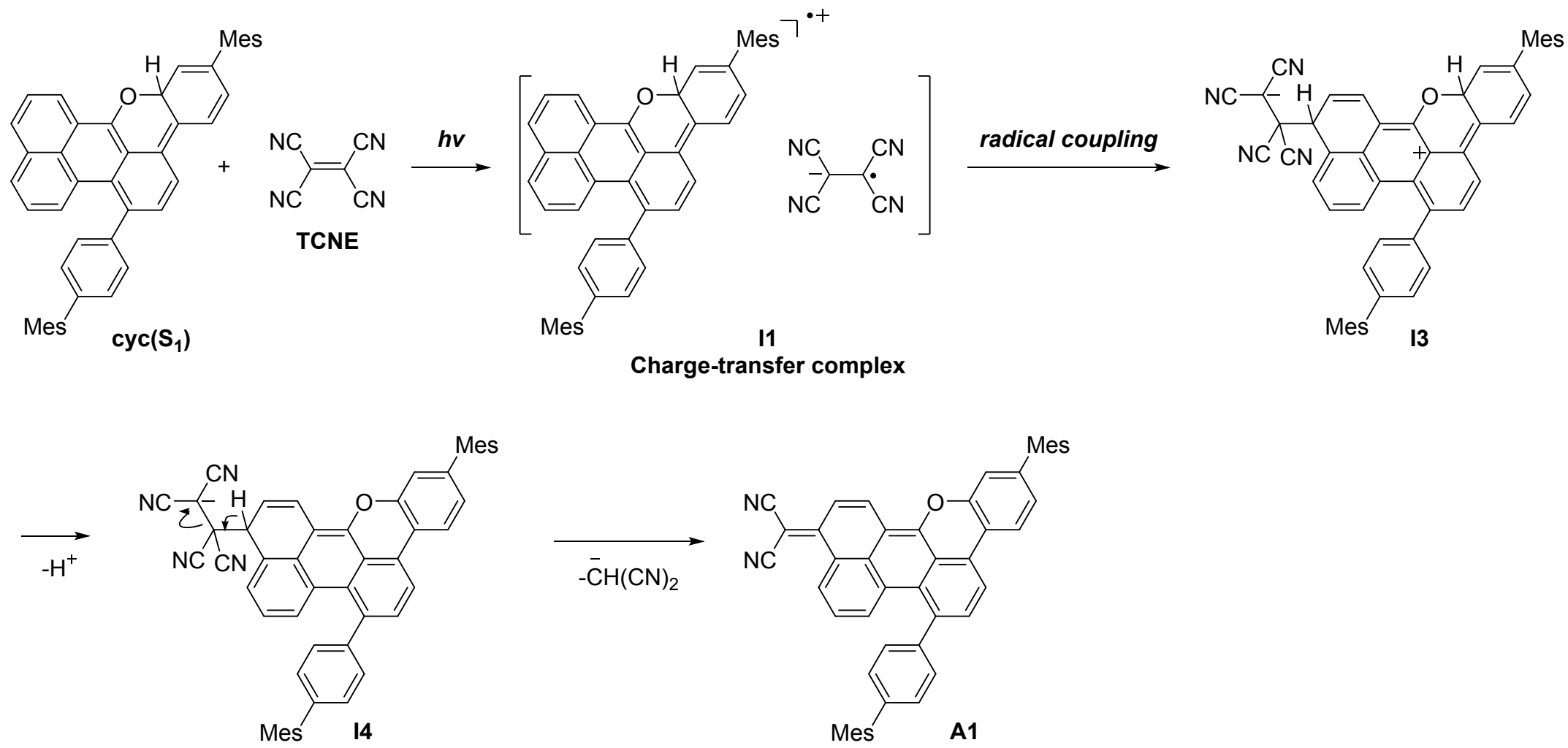

Scheme S3. Proposed reaction mechanism proceeding via a direct radical coupling sequence.

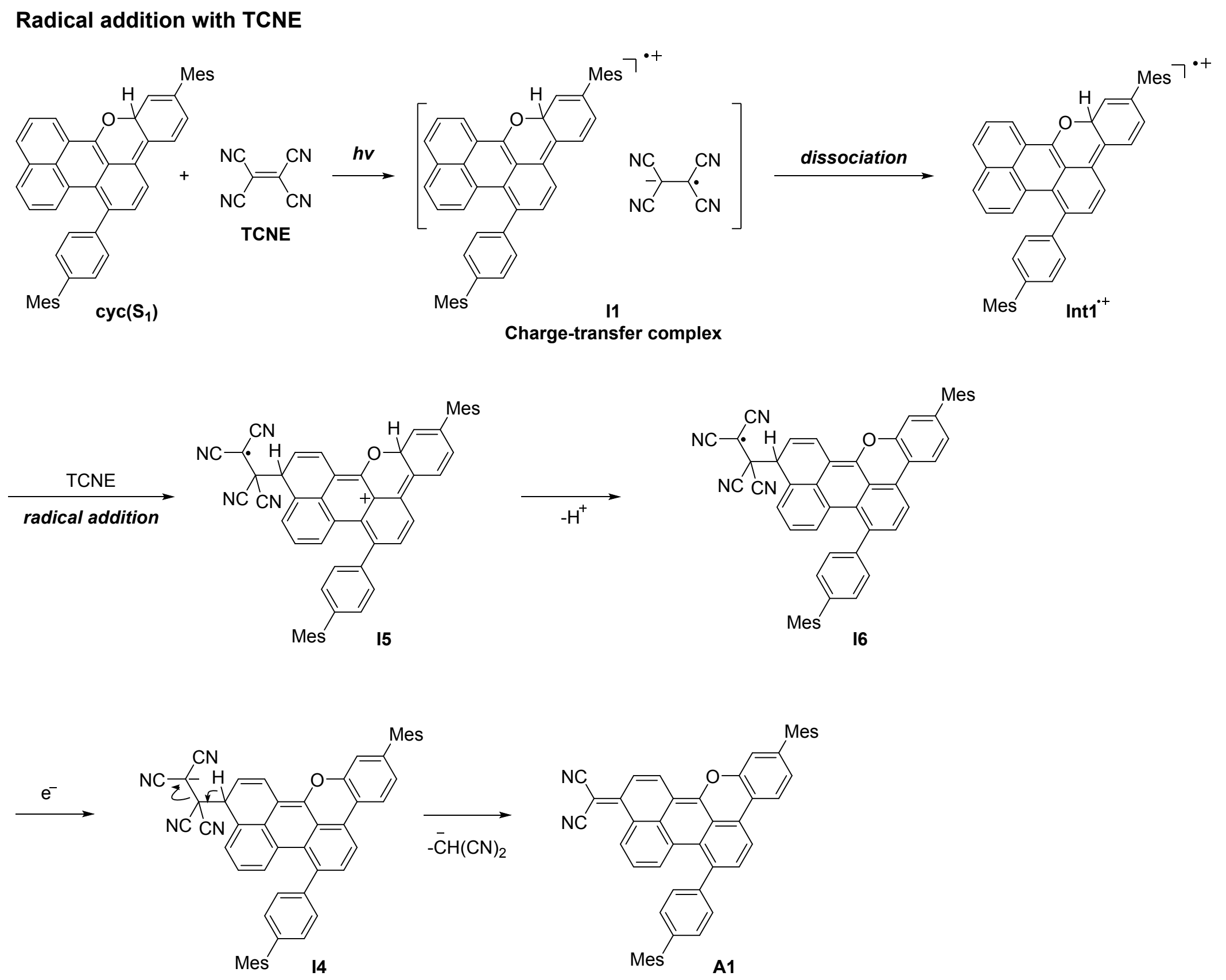

Scheme S4. Proposed reaction mechanism proceeding via a radical addition sequence.

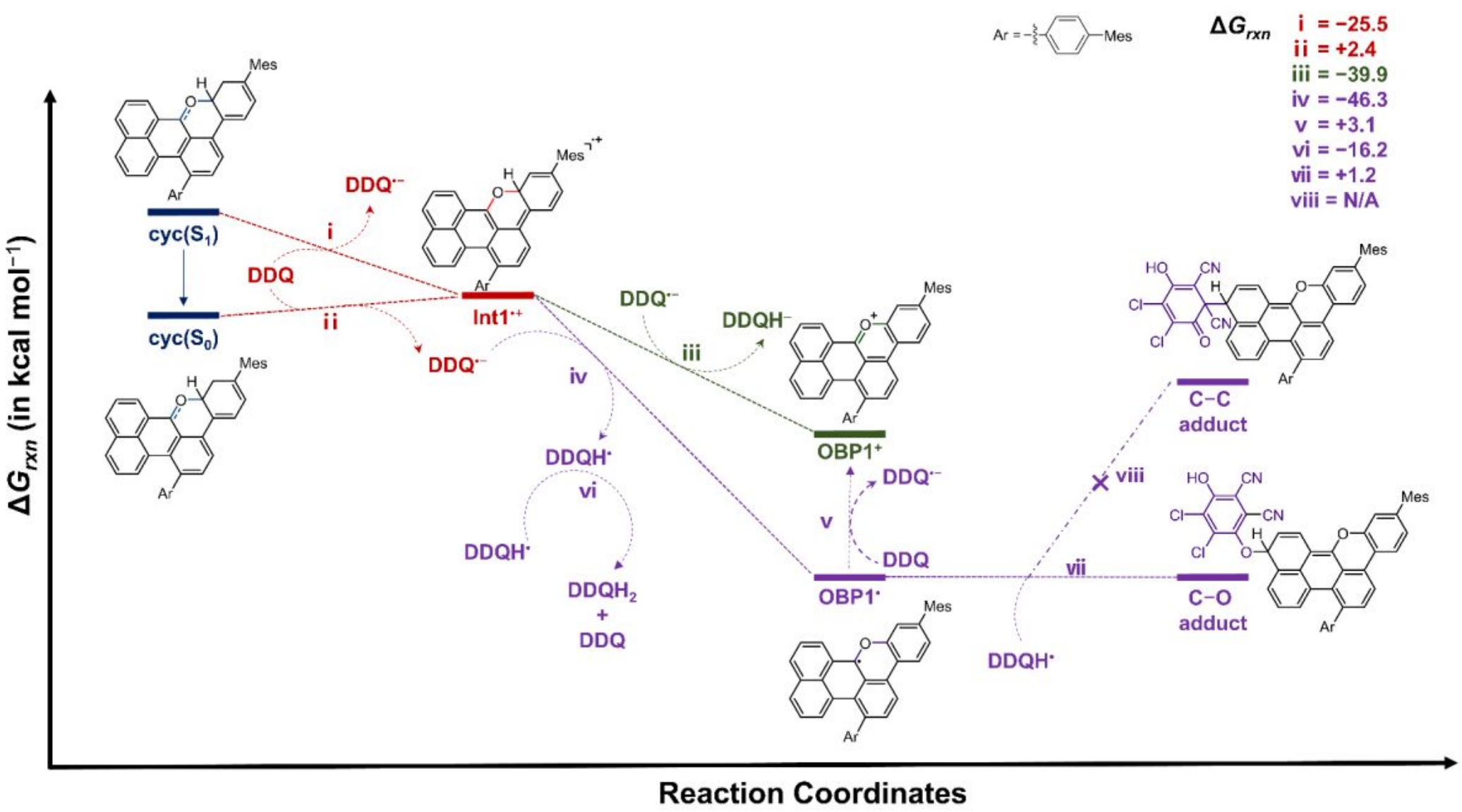

Figure S3. Free energy profile for the photochemical cyclization of **5a** in the presence of DDQ, determined by DFT calculations at the (U)CAM-B3LYP/6-311+G** level of theory. As shown in the TCNE system (Figure 2), the profile starts from the charge-transfer (CT) step. Relative Gibbs free

energies are given in kcal mol^{-1}.

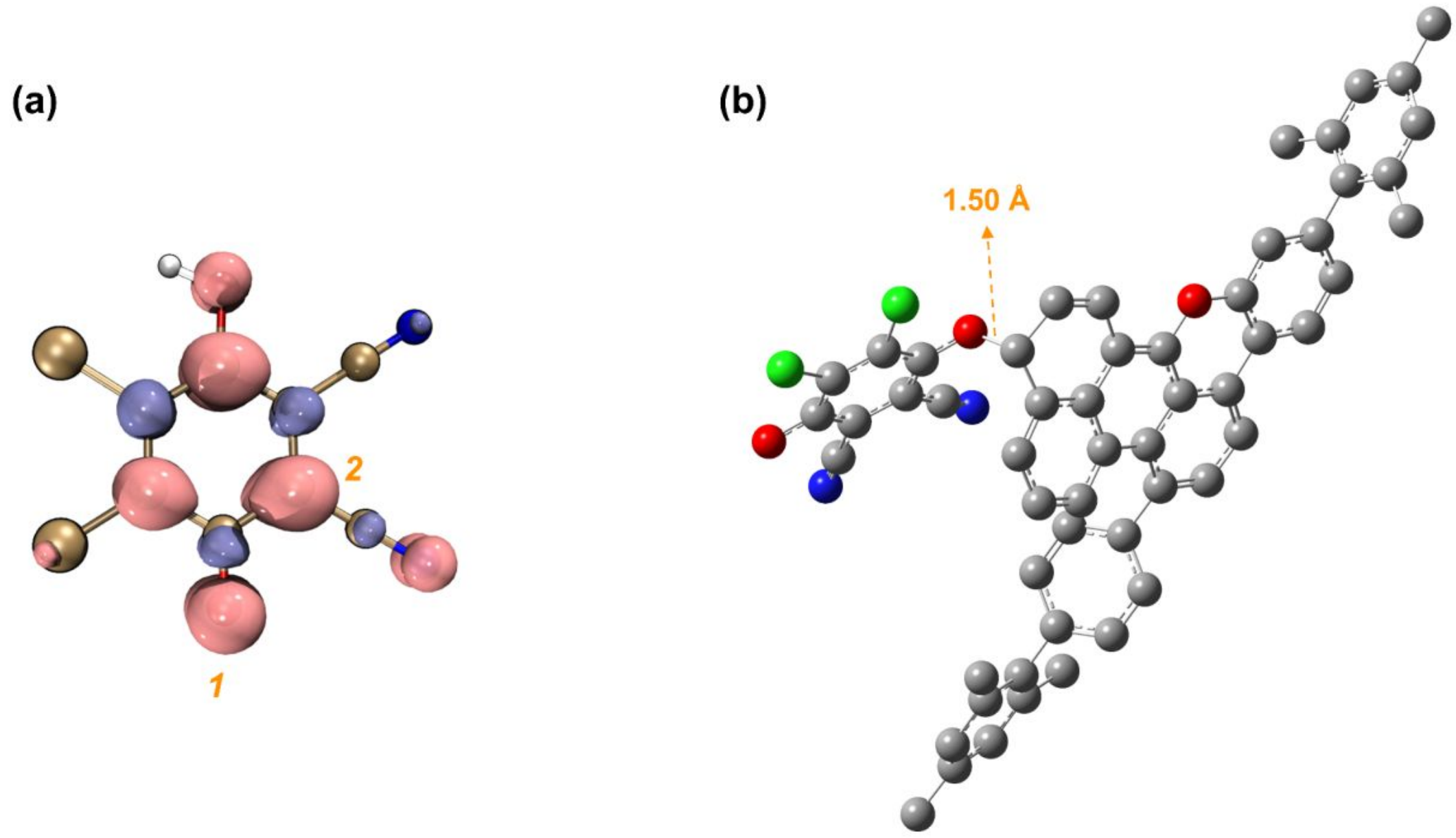

Figure S4. (a) Spin density map of DDQH$^•$ (isovalue = 0.005); (b) optimized C–O adduct structure with a C–O bond length of 1.50 Å. Hydrogen atoms are omitted. Geometry optimizations were performed at the (U)CAM-B3LYP/6-311+G** level of theory.

Upon reaction with DDQ$^{•-}$, the transient radical cation **Int1$^{•+}$** can undergo hydrogen atom transfer (HAT) to directly afford **OBP1$^+$** and DDQH$^-$ ($\Delta G_{rxn} = -39.9$ kcal mol^{-1}), or proton transfer (PT) to generate **OBP1$^•$** and the semiquinone radical DDQH$^•$ ($\Delta G_{rxn} = -46.3$ kcal mol^{-1}). Both pathways are strongly exergonic. However, only PT produces the **OBP1$^•$**/DDQH$^•$ radical pair capable of further coupling.

Guided by the spin-density distribution of DDQH$^•$, radical coupling was examined at the oxygen atom or the cyano-substituted carbon (*1* and *2*, respectively; Figure S4a). The C–O adduct converges to a local minimum but is thermodynamically unstable ($\Delta G_{rxn} = +1.2$ kcal mol^{-1}, Figure S3), with an elongated C–O bond (1.50 Å vs. 1.41–1.43 Å for typical C–O single bonds; Figure S4b) indicative of a weakened linkage, which is prone to spontaneous cleavage under the reaction conditions. No stable C–C coupling adduct could be located, as bond formation at this carbon disrupts the

aromaticity of the DDQH• framework. The geometry optimizations instead relaxed to the separated **OBP1**$^{+}$/DDQH^{-} pair.

Moreover, DDQH• readily disproportionates to DDQ and $DDQH_2$ ($\Delta G_{rxn} = -16.2$ kcal mol^{-1}),[4,5] rapidly depleting the semiquinone radical and kinetically outcompeting coupling to **OBP1•**. Additionally, **OBP1•** may undergo thermodynamically accessible single-electron transfer to DDQ to regenerate **OBP1**$^{+}$. Consequently, neither O- nor C-centered coupling affords a stable DDQ-derived adduct, and both HAT and PT ultimately converge on **OBP1**$^{+}$ as the thermodynamic sink, consistent with the exclusive formation of **OBP1**$^{+}$ in the DDQ reaction.

3.3 Electronic Structure Analysis

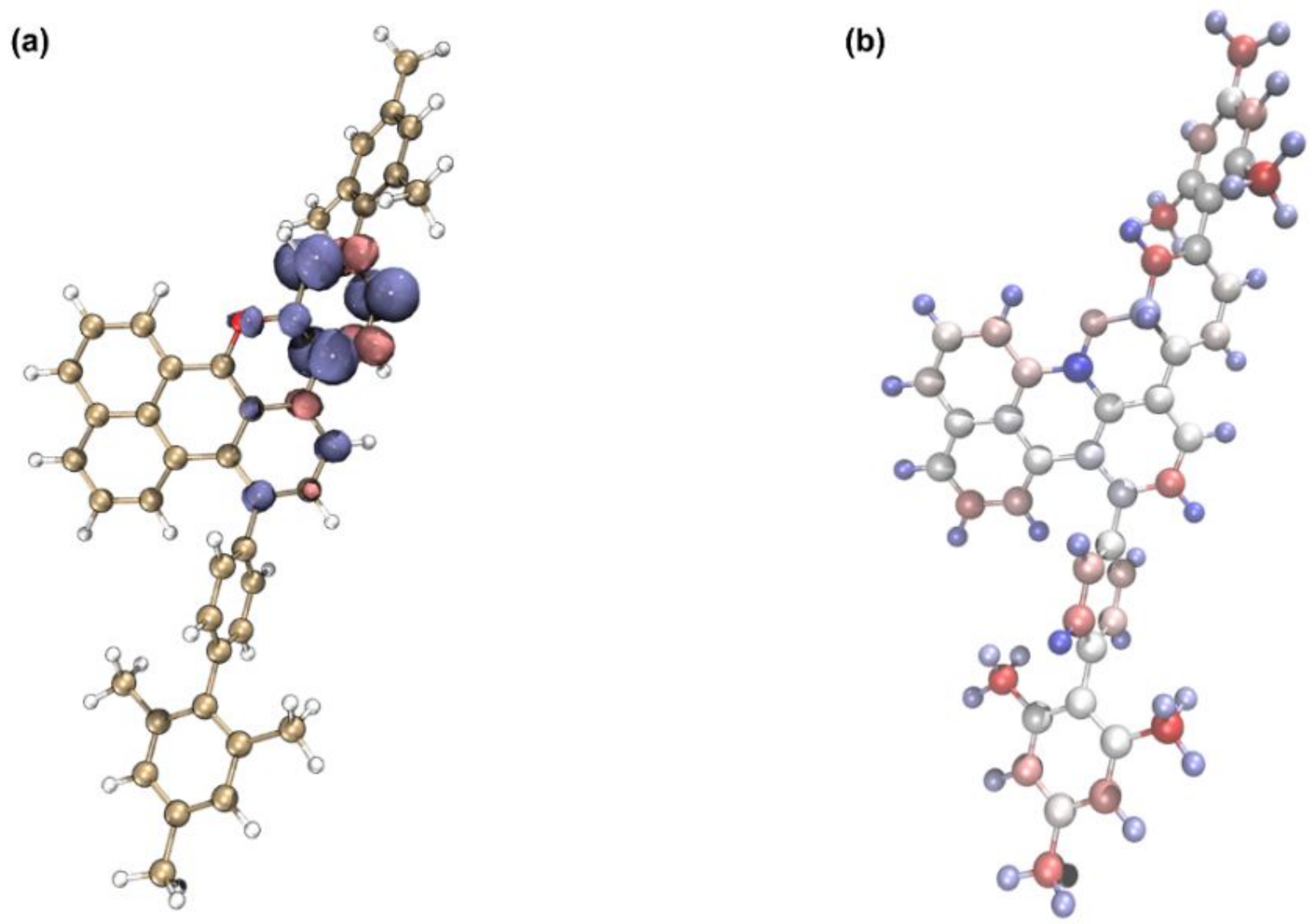

Figure S5. Calculated (a) spin density map (isosurface = 0.005) and (b) atomic dipole-corrected Hirshfeld (ADCH) atomic charges of radical cation **Int1**$^{\bullet+}$. Blue and red colors in (b) denote positive and

negative charge centers, respectively. All calculations were performed at the UCAM-B3lYP/6-311+G** level of theory.

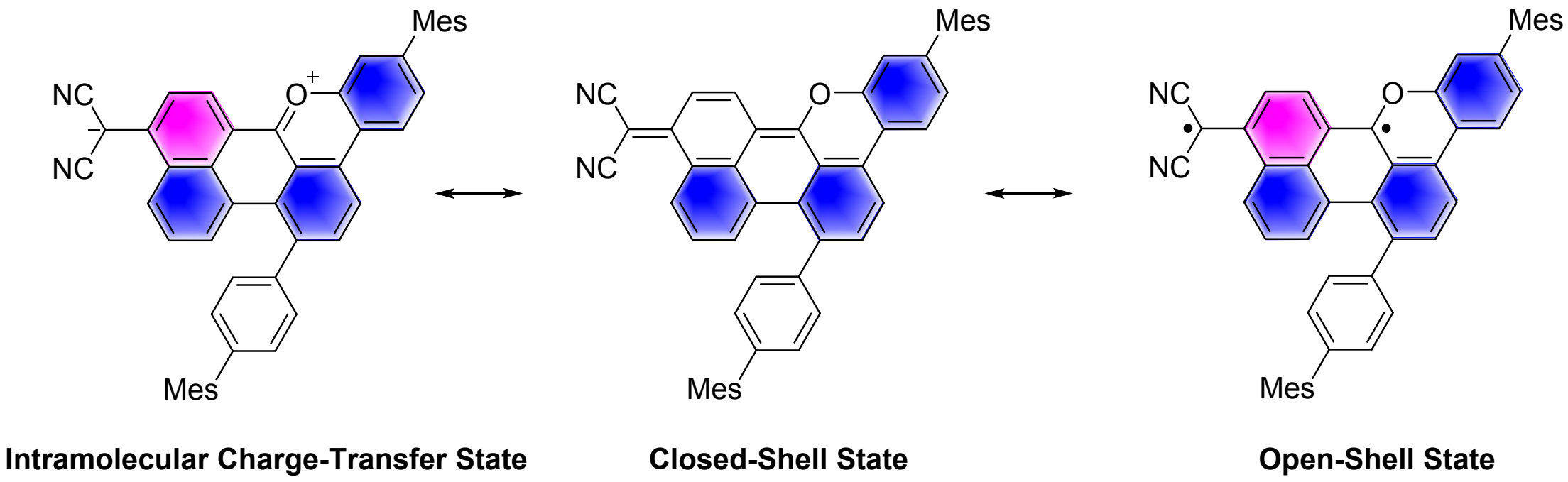

Figure S6. Resonance representations of **A1**, illustrating three electronic descriptions: intramolecular charge-transfer, closed-shell, and open-shell forms. SCXRD data are consistent with a predominantly closed-shell electronic structure.

3.4 Electrochemical and Optical Properties

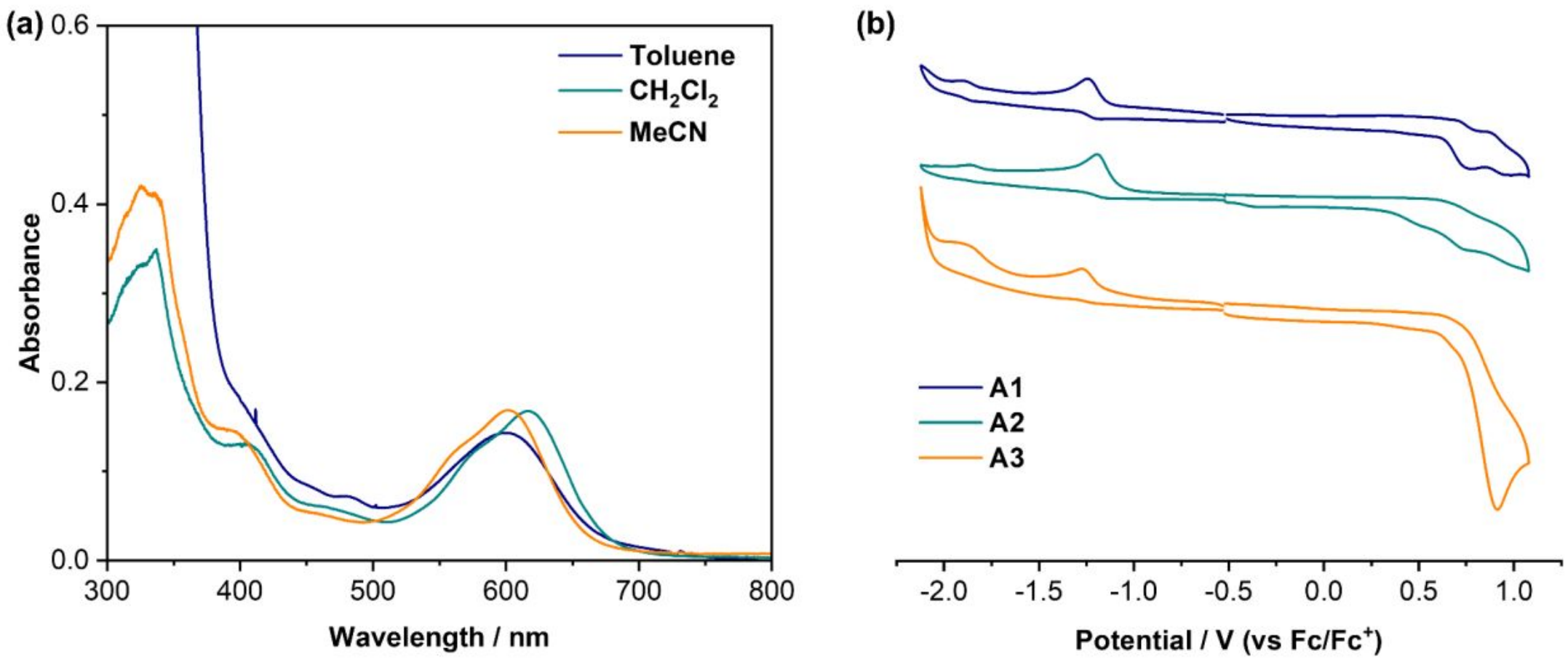

Figure S7. (a) Solvent-dependent UV–vis spectra of **OBP3·TFA** in toluene, CH_2Cl_2, and MeCN at 1 × 10^{-5} M. (b) Cyclic voltammograms of **A1**–**A3** measured in CH_2Cl_2 under N_2 with 0.1 M $TBAPF_6$ as the supporting electrolyte (scan rate = 100 mV s^{-1}), referenced to the Fc/Fc^+ couple.

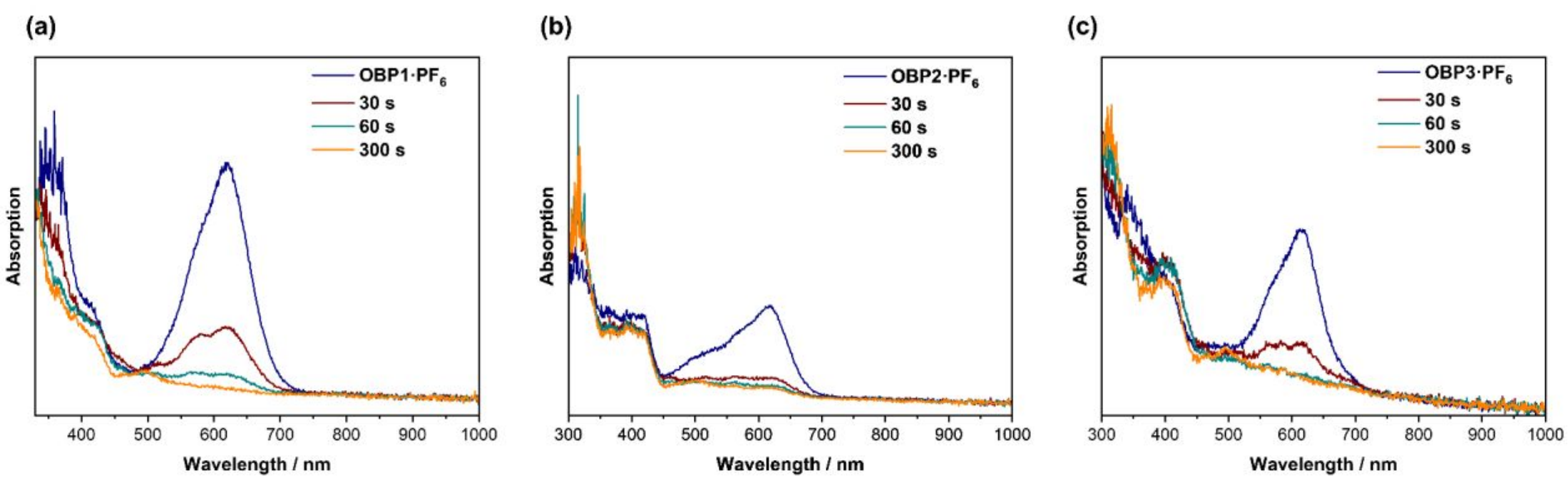

Figure S8. *In situ* spectroelectrochemical UV–vis–NIR absorption spectra of (a) **OBP1·PF$_6$**, (b) **OBP2·PF$_6$**, and (c) **OBP3·PF$_6$** recorded upon electrochemical reduction at −0.9 V vs Fc/Fc^+ in CH_2Cl_2

containing 0.1 M $TBAPF_6$ in air, showing the time evolution of the absorption profiles at 0−300 s.

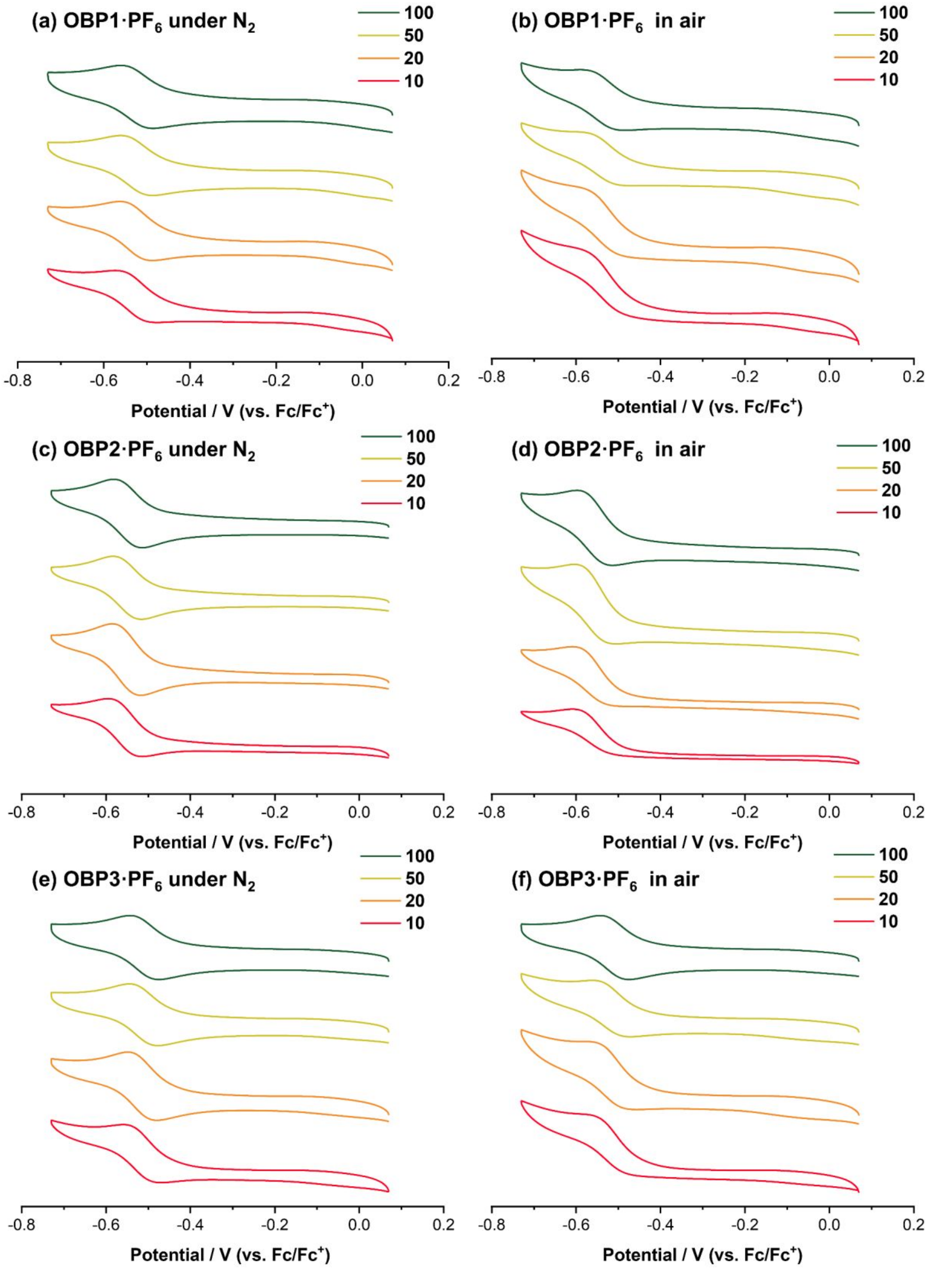

Figure S9. Scan-rate-dependent cyclic voltammograms of (a,b) **OBP1·PF$_6$**, (c,d) **OBP2·PF$_6$**, and (e,f) **OBP3·PF$_6$** recorded in CH_2Cl_2 under N_2 and air, respectively. Scan rates are 10–100 mV s^{-1}. Potentials are referenced to the Fc/Fc^+ couple.

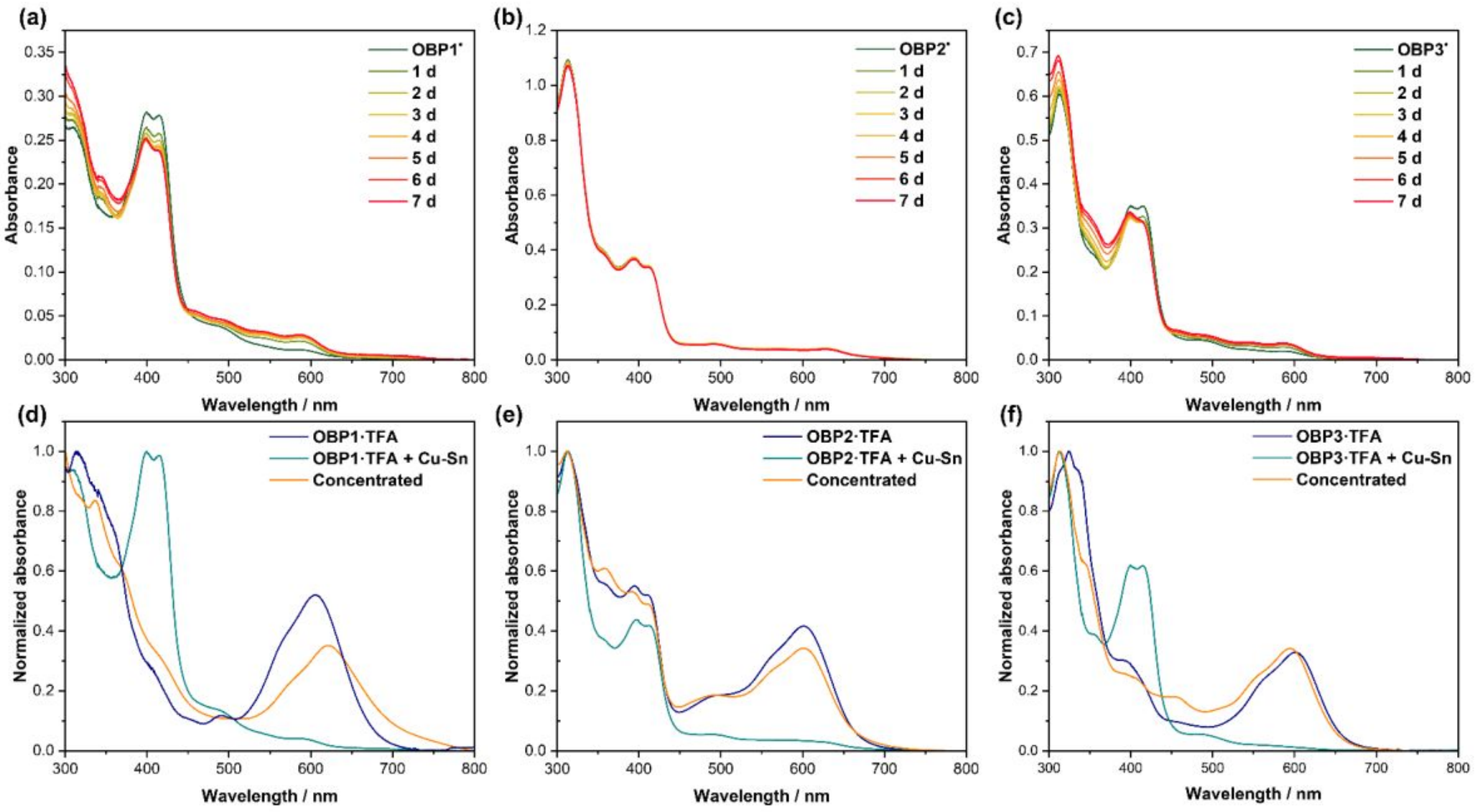

Figure S10. UV–vis absorption spectra of **OBP1·TFA**, **OBP2·TFA**, and **OBP3·TFA** in MeCN following chemical reduction with Cu-Sn alloy powder. Excess metal powder was removed by filtration through a 0.45 μm PTFE syringe filter, and the solutions were diluted from ca. 1.5 × 10^{-3} M to ca. 2.0 × 10^{-5} M prior to analysis. (a–c) Time-dependent absorption spectra recorded in air, demonstrating the persistence of the neutral radical species over 0–7 days. (d–f) Absorption spectra measured after solvent concentration and reconstitution, indicating reversion toward the cationic states.

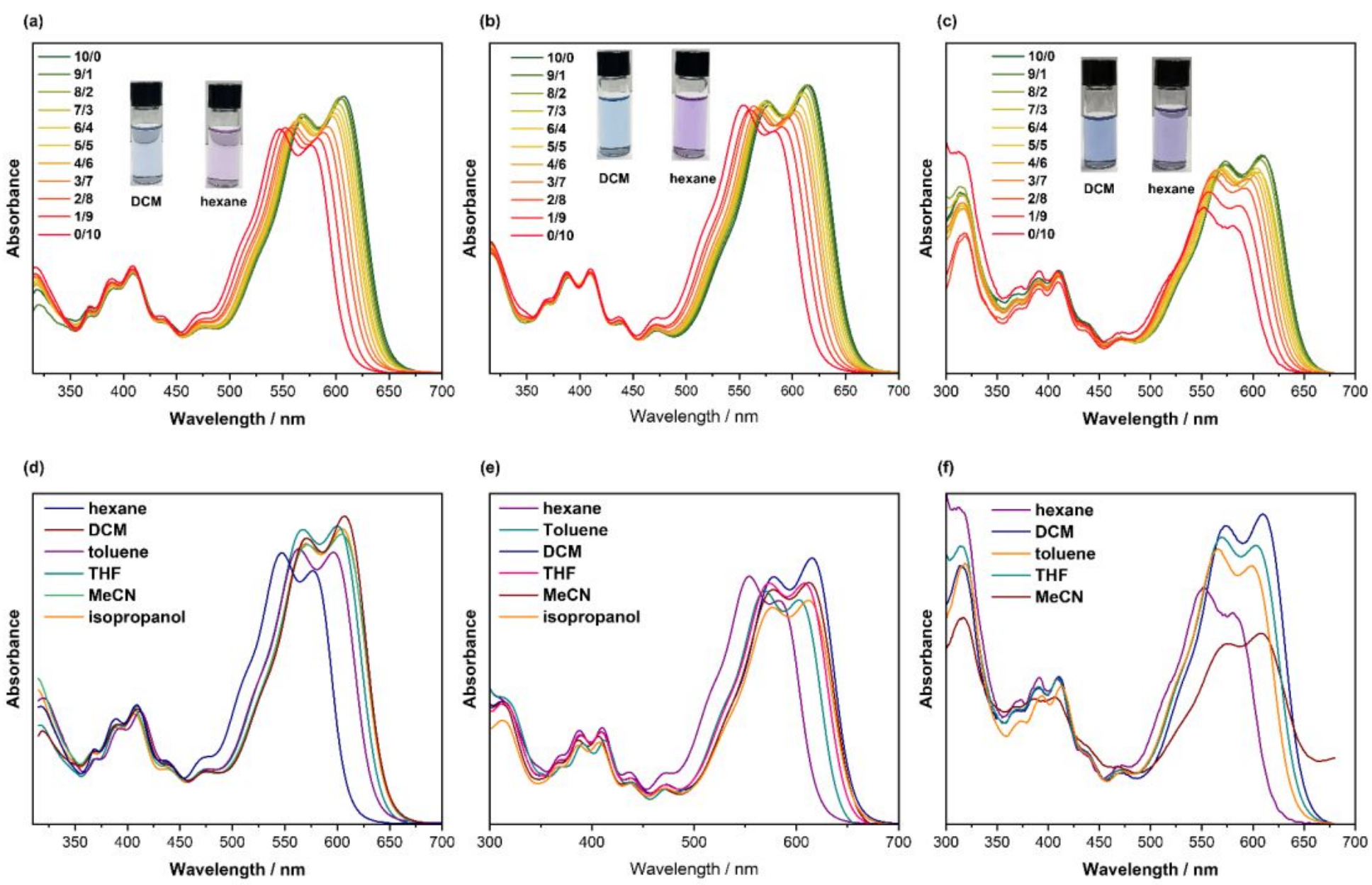

Figure S11. (a–c) UV–vis absorption spectra of **A1**–**A3** in CH_2Cl_2/hexane mixed solvents with varying volume ratios. (d–f) UV–vis absorption spectra of **A1**–**A3** recorded in solvents of different polarity. **A1** and **A2** were measured at ca. 1 × 10^{-5} M, while **A3** was recorded at ca. 2.5 × 10^{-6} M owing to its limited solubility.

Table S2. Absorption peak positions and vibronic intensity ratios of **A1** measured in different solvents at a concentration of 1 × 10^{-5} M. The (0,0) and (0,1) vibronic transition wavelengths ($\lambda_{(0,0)}$ and $\lambda_{(0,1)}$) and the corresponding intensity ratios $I_{(0,1)}/I_{(0,0)}$ are listed.

Solvents	$\lambda_{(0,0)}$ / nm	$\lambda_{(0,1)}$ / nm	$I_{(0,1)}/I_{(0,0)}$
Hexane	576	547	1.07
Toluene	596	563	1.01
DCM	607	570	0.93
THF	600	567	0.99
MeCN	603	571	0.96
Isopropanol	605	569	0.95

Table S3. Absorption peak positions and vibronic intensity ratios of **A2** measured in different solvents at a concentration of 1 × 10^{-5} M. The (0,0) and (0,1) vibronic transition wavelengths ($\lambda_{(0,0)}$ and $\lambda_{(0,1)}$) and the corresponding intensity ratios $I_{(0,1)}/I_{(0,0)}$ are listed.

Solvents	$\lambda_{(0,0)}$ / nm	$\lambda_{(0,1)}$ / nm	$I_{(0,1)}/I_{(0,0)}$
Hexane	584	553	1.09
Toluene	603	569	1.04
DCM	615	577	0.93
THF	607	572	1.01
MeCN	612	577	0.97
Isopropanol	612	576	0.97

Table S4. Absorption peak positions and vibronic intensity ratios of **A3** measured in different solvents at a concentration of 2.5 × 10^{-6} M. The (0,0) and (0,1) vibronic transition wavelengths ($\lambda_{(0,0)}$ and $\lambda_{(0,1)}$) and the corresponding intensity ratios $I_{(0,1)}/I_{(0,0)}$ are listed.

Solvents	$\lambda_{(0,0)}$ / nm	$\lambda_{(0,1)}$ / nm	$I_{(0,1)}/I_{(0,0)}$
Hexane	581	551	1.12
Toluene	600	565	1.07
DCM	610	573	0.96
THF	604	570	1.03
MeCN	697	573	0.94

Table S5. Summary of the photophysical and electrochemical properties of **OBP1·TFA−OBP3·TFA**, **A1−A3**, and **OBP1**$^•$**−OBP3**$^•$.

Compound	λ_{max} / nm	λ_{onset} / nm	Eopt g[a] / eV	E_{LUMO}[b] / eV	E_{HOMO}[c] / eV	Ecv g[d] / eV	E_{LUMO}[e] / eV	E_{HOMO}[e] / eV	E_g[e] / eV
**OBP1·TFA**	620	745	1.66	−4.28	-	-	−3.86	−6.64	2.81
**OBP2·TFA**	619	743	1.67	−4.26	-	-	−3.81	−6.46	2.65
**OBP3·TFA**	616	740	1.68	−4.29	-	-	−3.86	−6.63	2.77
**A1**	606	689	1.80	−3.72	−5.42	1.70	−3.02	−5.74	2.72
**A2**	614	696	1.78	−3.67	−5.28	1.61	−3.01	−5.68	2.67
**A3**	609	692	1.79	−3.66	−5.53	1.87	−3.03	−5.74	2.71
**OBP1**$^•$	592	750	1.65	-	-	-	−1.61[f]	−4.49[f]	2.88
**OBP2**$^•$	635	739	1.68	-	-	-	−1.59[f]	−4.45[f]	2.86
**OBP3**$^•$	593	723	1.72	-	-	-	−1.63[f]	−4.48[f]	2.85

[a] Estimated from Eopt g = 1240 / λ_{onset}. [b] Calculated from E_{LUMO} = –4.80 – Ered onset (calibration by Fc/Fc$^+$ couple). [c] Calculated from E_{HOMO} = –4.80 – Eox oneset (calibration by Fc/Fc$^+$ couple). The reduction and oxidation onset potentials were determined from CV by tangential intersection. [d] Calculated from Ecv g = E_{LUMO} – E_{HOMO}. [e] Estimated from DFT calculations. [f] For **OBP1**$^•$**–OBP3**$^•$, the singly occupied molecular orbital (SOMO) and the corresponding LUMO are reported.

3.5 Magnetic and Electrical Conductivity Analysis

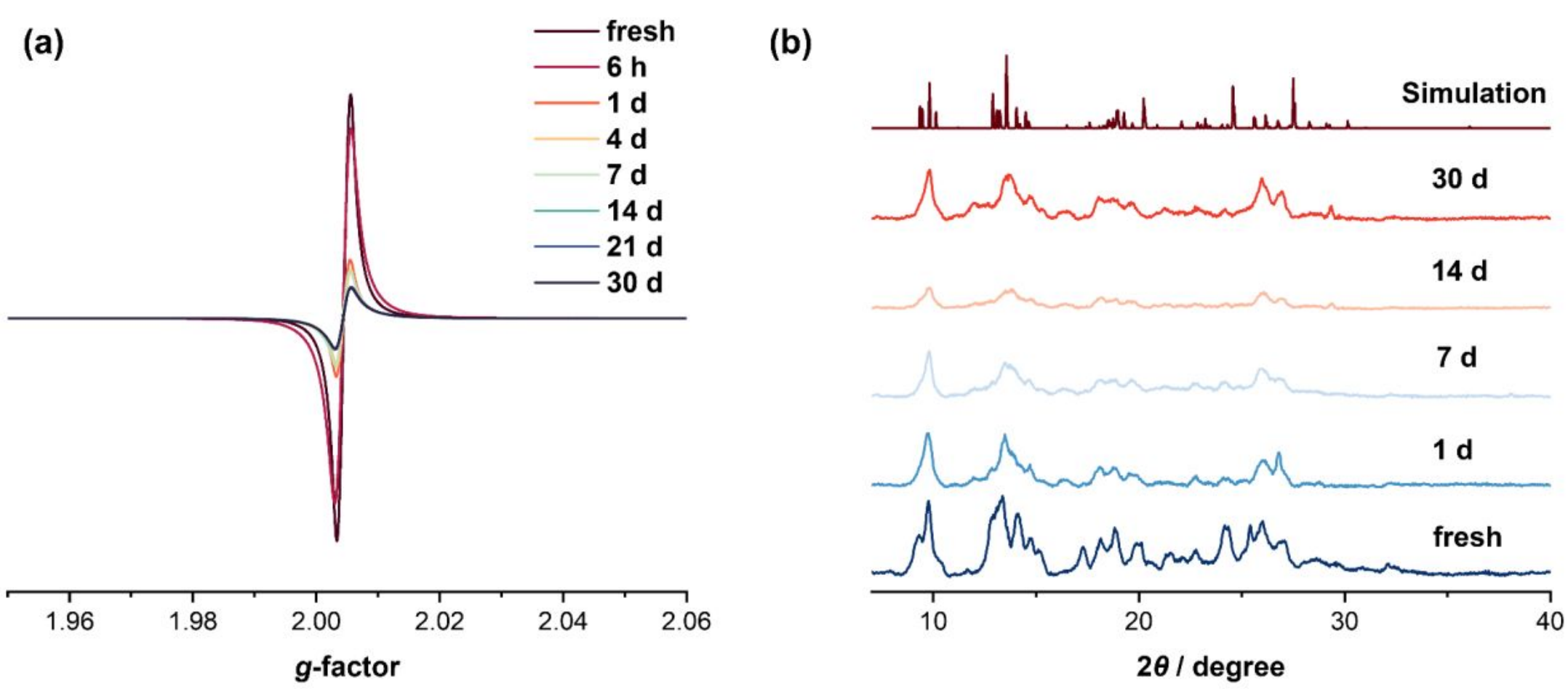

Figure S12. (a) Solid-state X-band EPR spectra of crystalline **OBP3·TCNE**$^•$ recorded at different time intervals under ambient conditions. (b) Corresponding PXRD patterns collected over the same time period.

Table S6. Best-fit parameters for the single-component simulation of the X-band EPR spectrum of **OBP3·TCNE•**.

Continuous-wave X-band EPR spectrum simulated using a single-component model (function: *pepper*; algorithm: Nelder–Mead simplex; baseline: none; target: data as is).

Parameter	Value
g-factor	2.0034
lwpp (mT)[1]	0.0915 / 0.4250
gStrain	3.21×10^{-4}

[1] lwpp = peak-to-peak linewidth (Gaussian/Lorentzian contributions).

Curie–Weiss Fitting

The temperature-dependent magnetic susceptibility was analyzed using the Curie–Weiss law:

$$\chi = \frac{C}{T - \theta} + \chi_0$$

where C is the Curie constant, θ is the Weiss temperature, and χ_0 is the temperature-independent susceptibility. The experimental χ(T) data were fitted over the temperature range of 1.7–300 K. The fitting yielded C = 0.35256 emu K mol^{-1}, θ = 0.20576 K, and $\chi_0 = -6.73 \times 10^{-3}$ emu mol^{-1}.

The effective magnetic moment was calculated according to:

$$\mu_{\text{eff}} = \sqrt{8C}$$

where C is the Curie constant obtained from the Curie–Weiss fitting, giving μ_{eff} = 1.68 μ_B per formula unit.

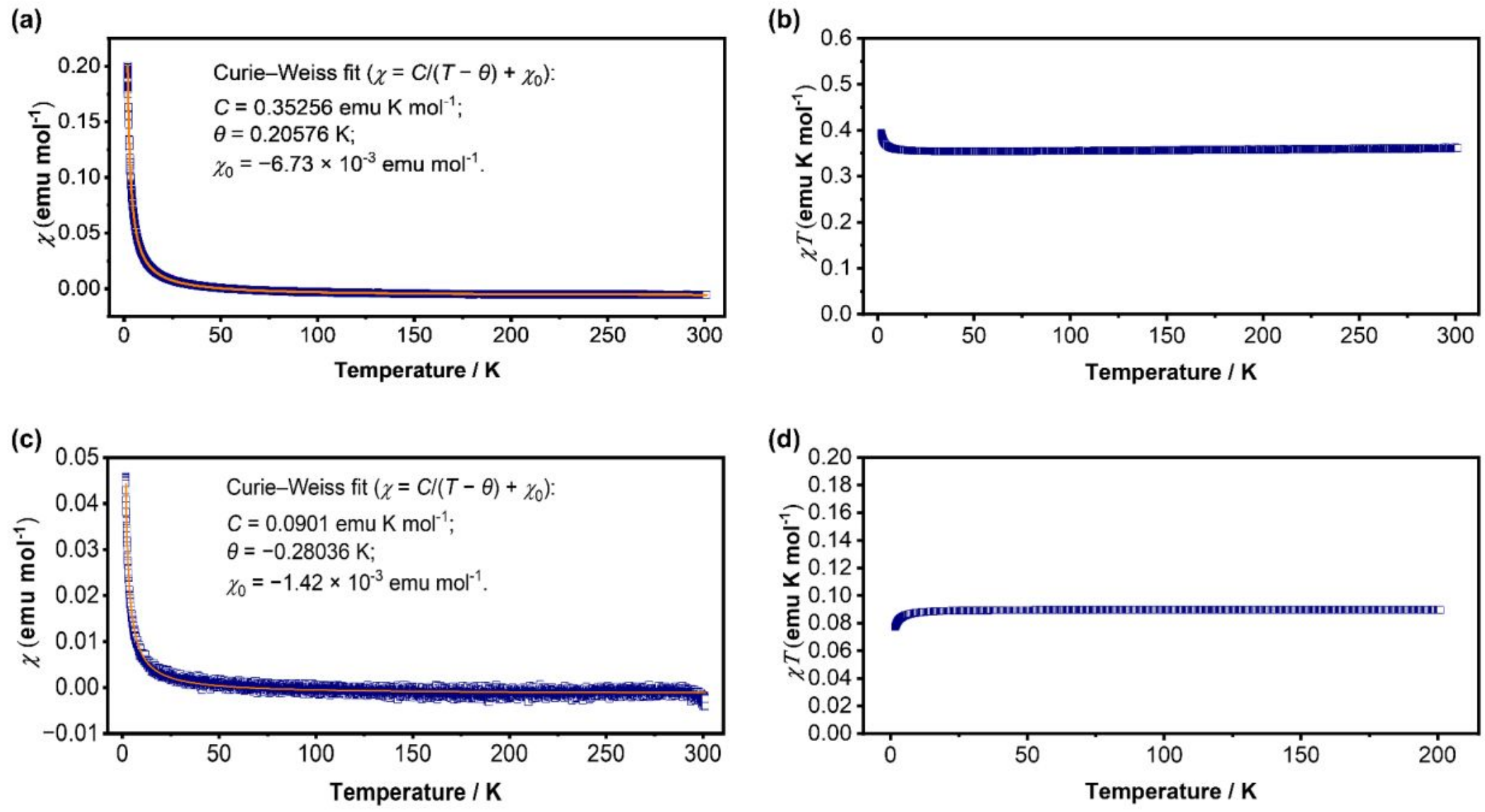

Figure S13. Temperature-dependent magnetic susceptibility of **OBP3·TCNE$^{•}$**. χ vs. T plots in (a) argon and (c) 18 h air exposure, with Curie–Weiss fits (orange lines) using $\chi = C/(T-\theta) + \chi_0$. χT vs. T plots in (b) argon and (d) 18 h air exposure, showing the temperature evolution of the effective magnetic moment.

Brillouin Fitting

Field-dependent magnetization measurements were performed at 1.7 K to further investigate the magnetic behavior. The magnetization curve was fitted using the Brillouin function:

$$M = Ng\mu_B J B_J(x)$$

where N is the number of magnetic centers, g is the Landé g-factor, μ_B is the Bohr magneton, and J is the total angular momentum quantum number. The Brillouin function $B_J(x)$ is defined as:

$$B_J(x) = \frac{2J+1}{2J}\coth\left(\frac{2J+1}{2J}x\right) - \frac{1}{2J}\coth\left(\frac{x}{2J}\right)$$

with

$$x = \frac{g\mu_B J B}{k_B T}$$

where B is the applied magnetic field and k_B is the Boltzmann constant.

The experimental magnetization curve is well reproduced by the Brillouin function assuming an S = ½ spin system, yielding a total angular momentum of 0.38 under argon.

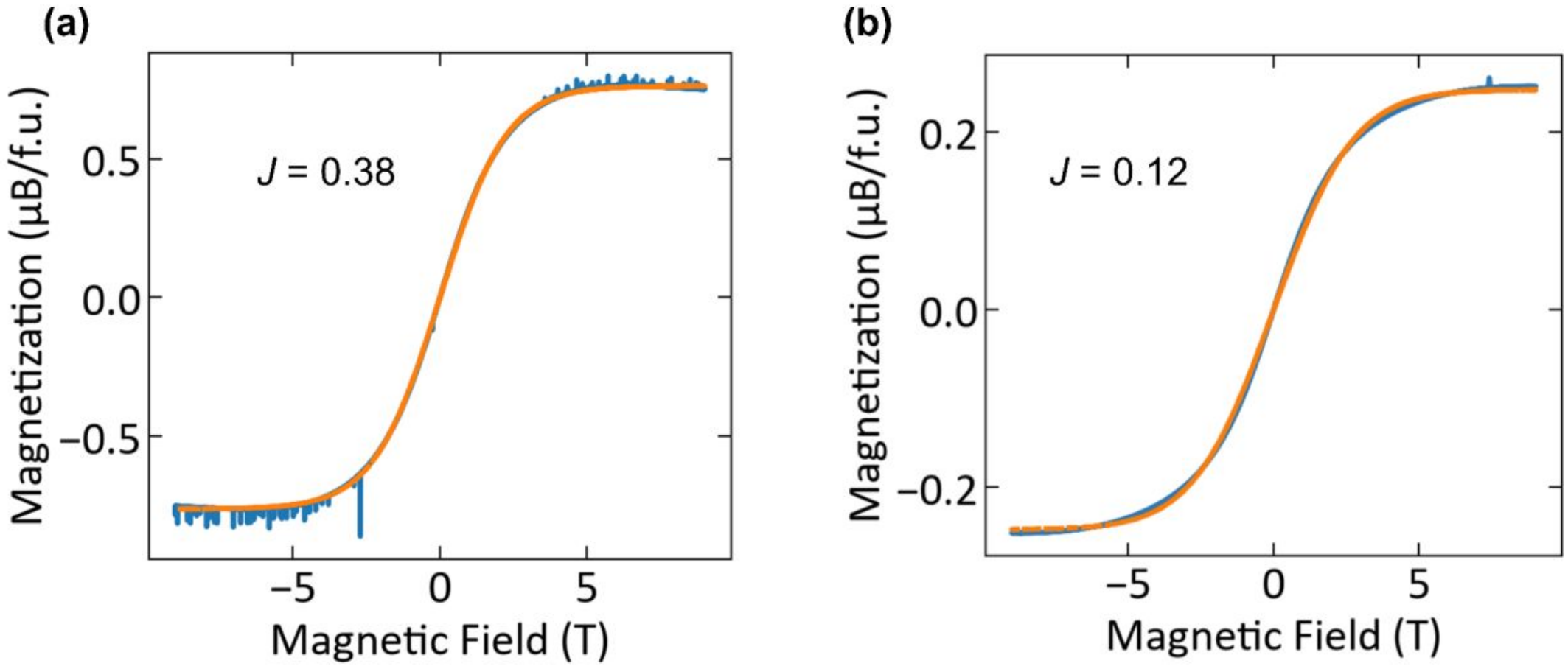

Figure S14. Field-dependent magnetization of **OBP3·TCNE•** measured at 1.7 K under (a) argon and (b) 18 h air exposure. Orange line represents the Brillouin function fitting assuming an S = ½ spin system.

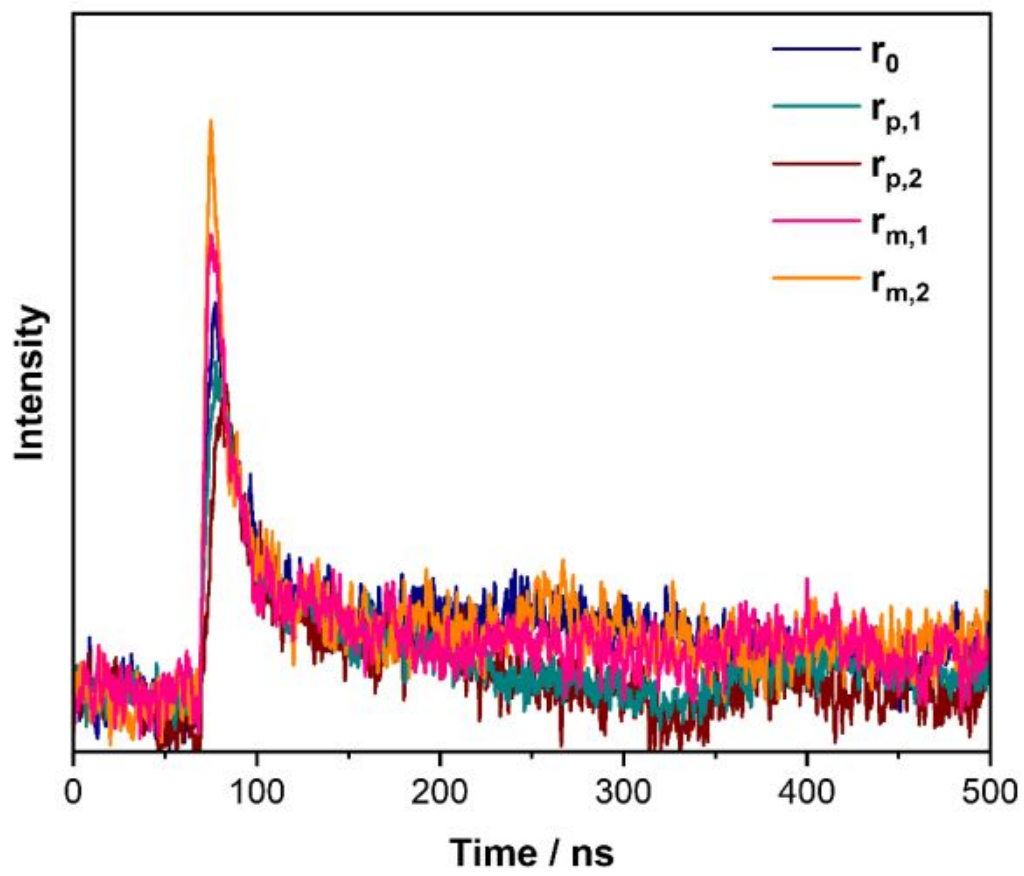

Figure S15. Raw transient responses of crystalline **OBP3·TCNE•** recorded at different microwave frequencies relative to the cavity resonant frequency (r_0, navy), +0.0005 GHz ($r_{p,1}$, cyan), +0.001 GHz ($r_{p,2}$, wine), −0.0005 GHz ($r_{m,1}$, pink) and −0.001 GHz ($r_{m,2}$, orange). The on-resonance trace (r_0) retains a non-zero component at longer timescales, consistent with long-lived charge carriers.

4. X-ray crystallography

X-ray diffraction data were measured on Bruker D8 Venture PHOTON III CMOS diffractometer equipped with a Cu Kα INCOATEC ImuS micro-focus source (λ = 1.54178 Å). Indexing was performed using APEX5[6] (Difference Vectors method). Data integration and reduction were performed using SaintPlus[7]. Absorption correction was performed by multi-scan method implemented in TWINABS[8]. Space group was determined using XPREP implemented in APEX5. Structure was solved using SHELXT[9] and refined using SHELXL-2019/3[10] (full-matrix least-squares on F^2)

through OLEX2 interface program[11]. The ellipsoid plot was made with Platon.

Table S7. Crystal data and structure refinement for **OBP3·TCNE[•]** (CCDC: 2545973).

Empirical formula	$C_{66}H_{48}FN_4O$
Moiety formula	$C_{53}H_{40}FO$, C_7H_8, C_6N_4
Formula weight	932.08
Temperature/K	100.00
Crystal system	triclinic
Space group	P-1
a/Å	10.0755(3)
b/Å	14.5764(4)
c/Å	18.9644(5)
α/°	72.6550(10)
β/°	89.340(2)
γ/°	70.5760(10)
Volume/Å^3	2495.32(12)
Z	2
ρ_{calc}g/cm^3	1.241
μ/mm^{-1}	0.598
F(000)	978.0
Radiation	CuKα (λ = 1.54178)
2θ range for data collection/°	4.904 to 144.56
Index ranges	$-12 \leq h \leq 12$, $-18 \leq k \leq 17$, $-23 \leq l \leq 23$
Reflections collected	88129
Independent reflections	9830 [R_{int} = 0.0512, R_{sigma} = 0.0274]
Data/restraints/parameters	9830/122/748
Goodness-of-fit on F^2	1.053
Final R indexes [I>=2σ (I)]	R_1 = 0.0440, wR_2 = 0.1168
Final R indexes [all data]	R_1 = 0.0509, wR_2 = 0.1224
Largest diff. peak/hole / e Å^{-3}	0.29/–0.23

Table S8. Crystal data and structure refinement for **OBP2·PCA** (CCDC: 2545974).

Empirical formula	$C_{69}H_{51}N_5O_2$
Moiety formula	$C_{54}H_{43}O_2$, C_8N_5, C_7H_8
Formula weight	982.14
Temperature/K	100.00
Crystal system	monoclinic
Space group	Cc
a/Å	10.0358(4)
b/Å	39.711(2)
c/Å	13.6146(5)
α/°	90
β/°	104.619(3)
γ/°	90
Volume/Å^3	5250.2(4)
Z	4
ρ_{calc}g/cm^3	1.243
μ/mm^{-1}	0.587
F(000)	2064.0
Crystal size/mm^3	0.189 × 0.088 × 0.024
Radiation	CuKα (λ = 1.54178)
2θ range for data collection/°	4.45 to 145.616
Index ranges	$-12 \leq h \leq 12$, $-48 \leq k \leq 49$, $-16 \leq l \leq 16$
Reflections collected	53198
Independent reflections	9909 [R_{int} = 0.1168, R_{sigma} = 0.0934]
Data/restraints/parameters	9909/478/812
Goodness-of-fit on F^2	1.034
Final R indexes [I>=2σ (I)]	R_1 = 0.0564, wR_2 = 0.1308
Final R indexes [all data]	R_1 = 0.0833, wR_2 = 0.1480
Largest diff. peak/hole / e Å^{-3}	0.17/–0.21

Flack parameter	0.1(5)

Table S9. Crystal data and structure refinement for **OBP2·TCE** (CCDC: 2545975).

Empirical formula	$C_{62.5}H_{47}N_3O_3$
Moiety formula	$C_{54}H_{43}O_2$, $0.5(C_7H_8)$, C_5N_3O
Formula weight	888.03
Temperature/K	100.00
Crystal system	monoclinic
Space group	$P2_1/n$
a/Å	10.0070(4)
b/Å	38.790(2)
c/Å	12.5761(5)
α/°	90
β/°	106.635(2)
γ/°	90
Volume/Å^3	4677.4(4)
Z	4
ρ_{calc}g/cm^3	1.261
μ/mm^{-1}	0.606
F(000)	1868.0
Radiation	CuKα (λ = 1.54178)
2θ range for data collection/°	4.556 to 144.66
Index ranges	$-12 \le h \le 12$, $-47 \le k \le 47$, $-15 \le l \le 15$
Reflections collected	105834
Independent reflections	9231 [R_{int} = 0.0848, R_{sigma} = 0.0662]
Data/restraints/parameters	9231/1554/865
Goodness-of-fit on F^2	1.010
Final R indexes [I>=2σ (I)]	R_1 = 0.0740, wR_2 = 0.2100
Final R indexes [all data]	R_1 = 0.0947, wR_2 = 0.2364
Largest diff. peak/hole / e Å^{-3}	0.23/–0.33

Table S10. Crystal data and structure refinement for **A1** (CCDC: 2545972).

Empirical formula	$C_{50.91}H_{37.83}Cl_{1.82}N_2O$
Moiety formula	$C_{50}H_{36}N_2O$, 0.91(CH_2Cl_2)
Formula weight	758.27
Temperature/K	100.00
Crystal system	triclinic
Space group	P-1
a/Å	8.5496(2)
b/Å	11.4377(3)
c/Å	20.4491(5)
α/°	103.3120(10)
β/°	100.4390(10)
γ/°	93.6510(10)
Volume/Å^3	1902.09(8)
Z	2
ρ_{calc}g/cm^3	1.324
μ/mm^{-1}	1.750
F(000)	793.0
Crystal size/mm^3	0.19 × 0.159 × 0.02
Radiation	CuKα (λ = 1.54178)
2θ range for data collection/°	7.992 to 144.558
Index ranges	$-10 \le h \le 10$, $-14 \le k \le 13$, $-25 \le l \le 25$
Reflections collected	56634
Independent reflections	7489 [R_{int} = 0.0394, R_{sigma} = 0.0240]
Data/restraints/parameters	7489/59/525
Goodness-of-fit on F^2	1.044
Final R indexes [I>=2σ (I)]	R_1 = 0.0430, wR_2 = 0.1194
Final R indexes [all data]	R_1 = 0.0451, wR_2 = 0.1217

Largest diff. peak/hole / e Å^{-3}	0.61/–0.45

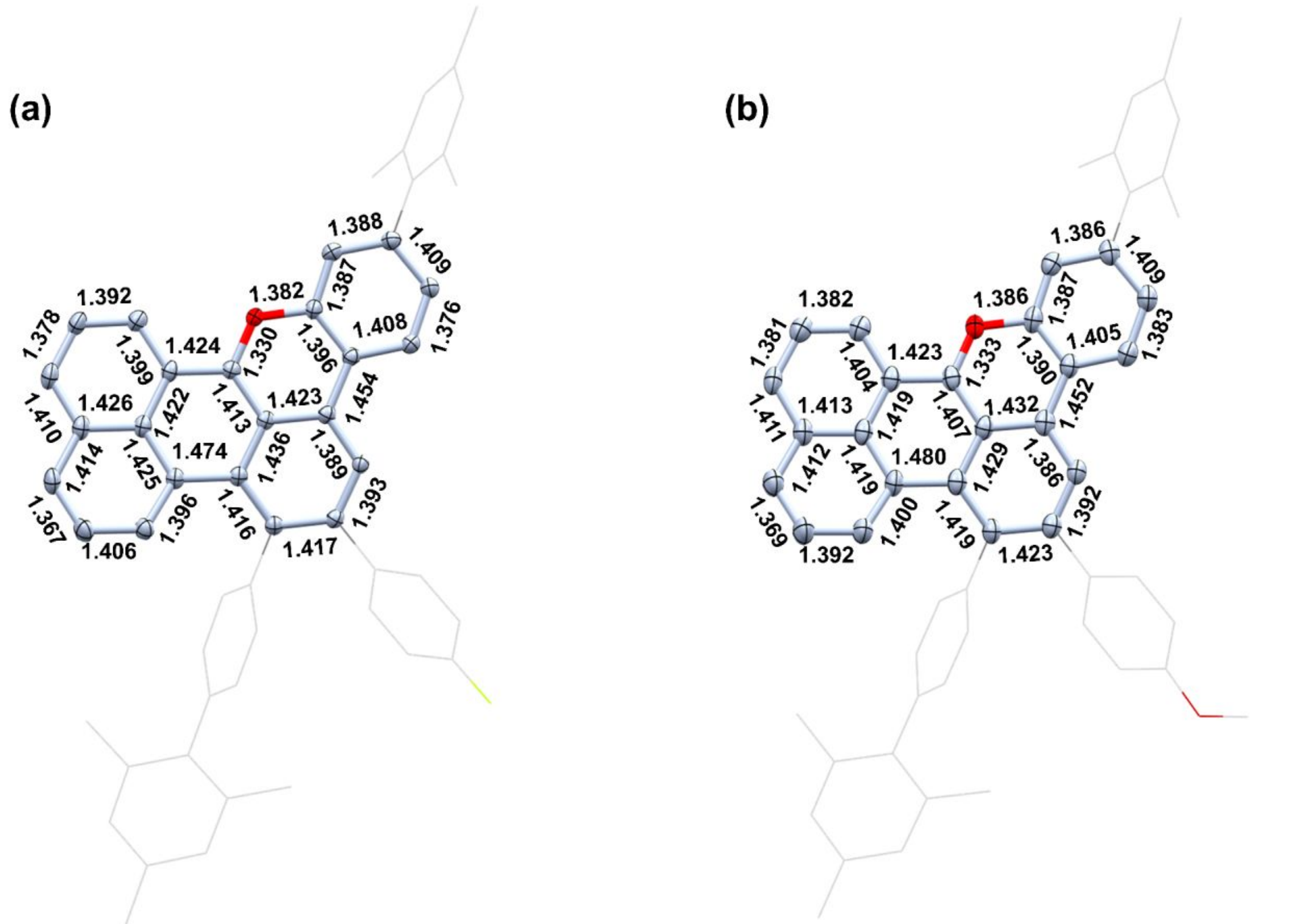

Figure S16. Single-crystal bond lengths of (a) **OBP3$^+$** and (b) **OBP2$^+$**, respectively. Interatomic distances are given in angstroms (Å).

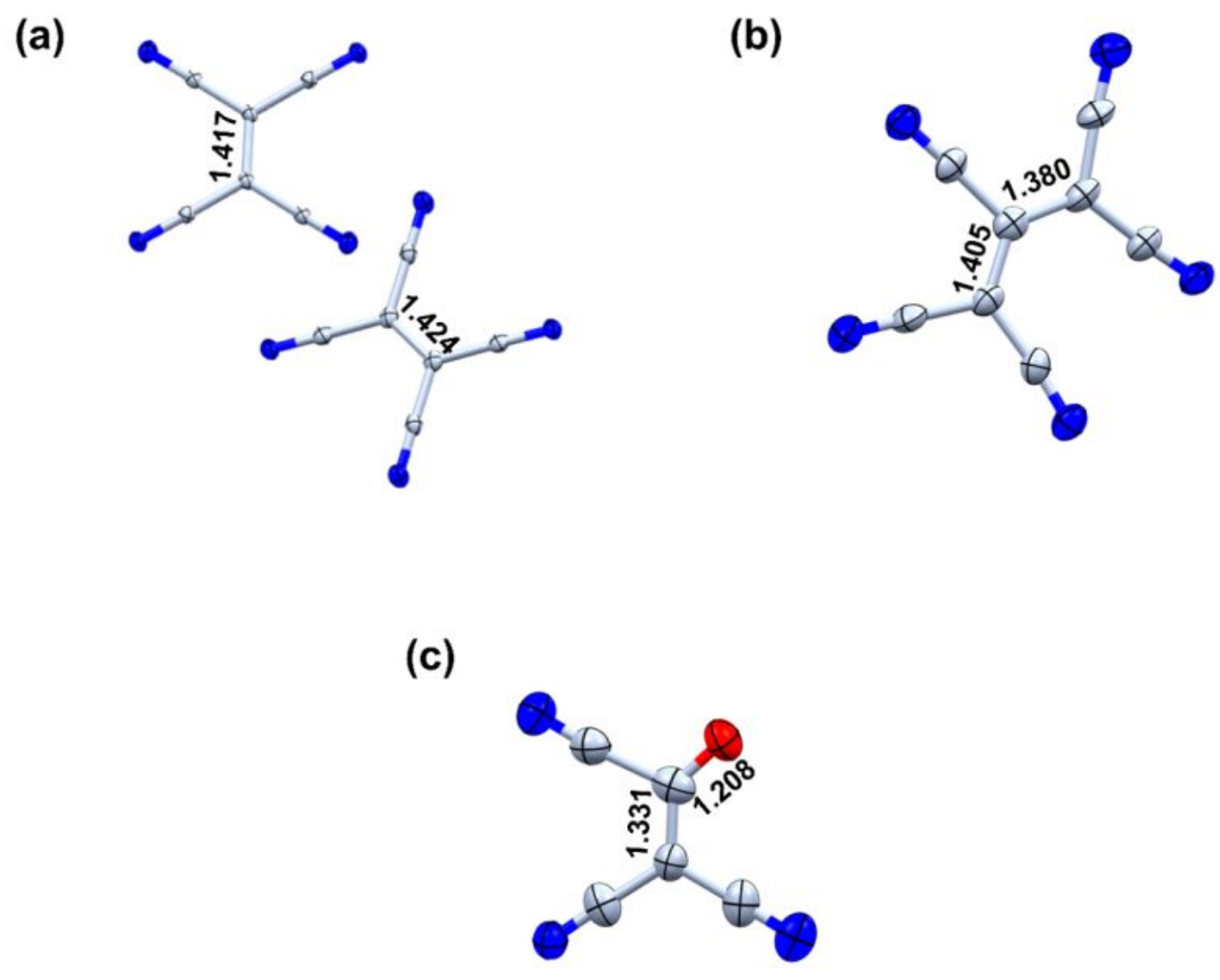

Figure S17. Selected bond metrics of the (a) **TCNE•−**, (b) **PCA−**, and (c) **TCE−** anions, respectively. Interatomic distances are given in angstroms (Å).

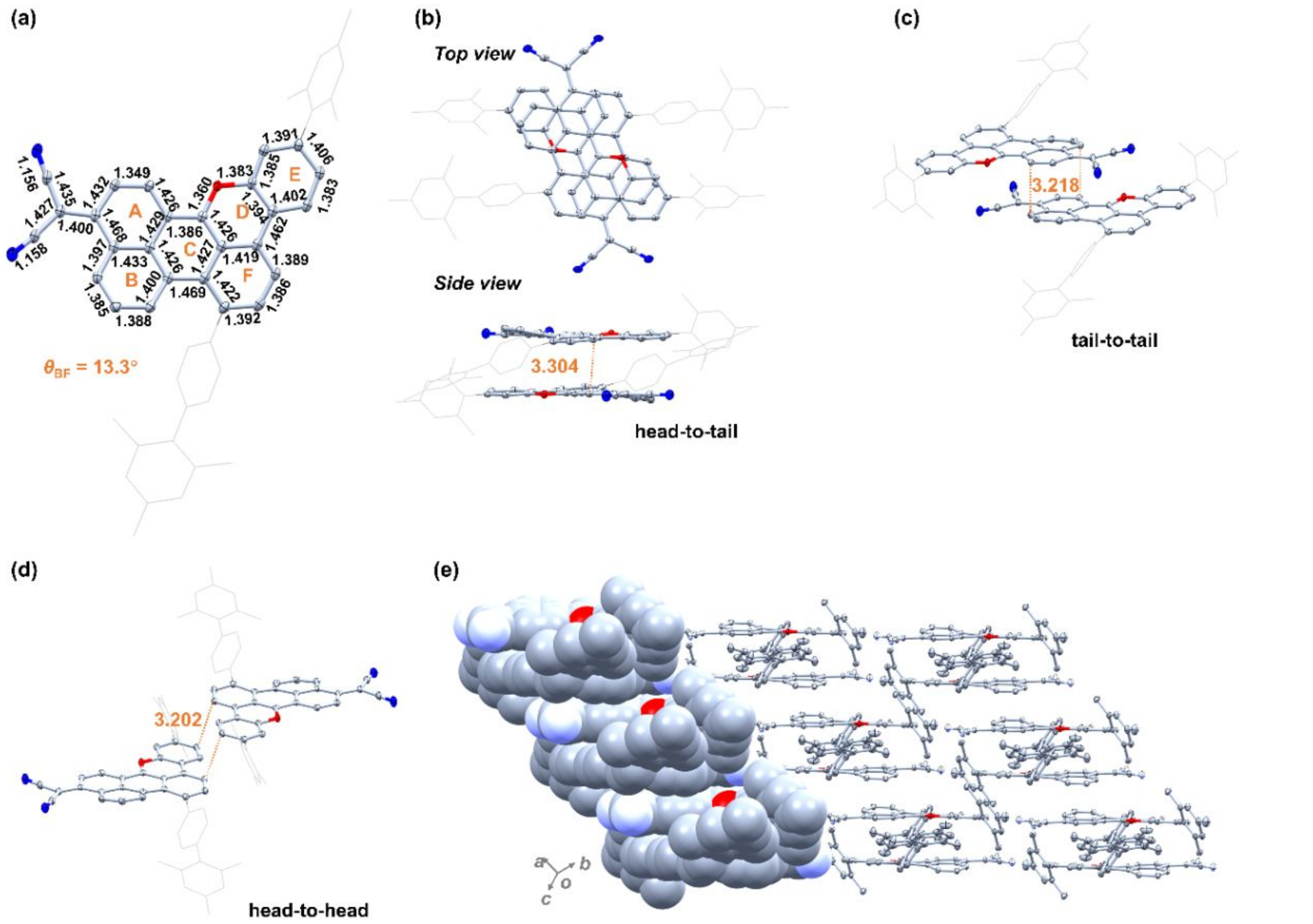

Figure S18. Single-crystal structure of **A1**. (a) ORTEP representation of **A1** (30% probability ellipsoids) with selected bond lengths and bay-region torsion angles highlighted. (b–d) Solid-state packing views showing head-to-tail, tail-to-tail, and head-to-head π-stacking motifs, respectively. (e) Solid-state packing view of **A1** showing slipped 1D columnar π-stacking with a finite lateral offset between adjacent π-components. Selected interatomic distances are given in angstroms (Å). Hydrogen atoms and solvent molecules are omitted for clarity.

5. Computations

DFT calculations were employed throughout this study. All theoretical calculations were carried out using the Gaussian 16 software package.[12] With implicit solvation (SMD, CH_2Cl_2), geometry optimizations were performed using the PBE0[13] and CAM-B3LYP[14] functionals in conjunction with def2-SVP and 6-311+G** basis sets. Transition states (TSs) were searched using either Berny or transit-guided quasi-newton (QST2 and QST3) methods.[15] The nature of the fully optimized stationary points was verified by the frequency calculations at the same level of theory. (i.e., the number of

imaginary frequencies $n_i = 0$ for minima and $n_i = 1$ for TS). TD-DFT calculations were conducted at the (U)PBE0/def2-TZVP level to simulate UV–vis spectra. ACID calculations were carried out at the (U)PBE0/def2-TZVP level using the continuous set of gauge transformations (CSGT) formalism, whereas NICS(1)_zz values were computed using the GIAO method,[16] and 2D-ICSS maps were generated at the (U)PBE0/def2-SVP level. Wavefunction analyses, including NICS(1)_zz, LOL-π, 2D-ICSS, and NTO analyses, were performed using *Multiwfn*[17] program.

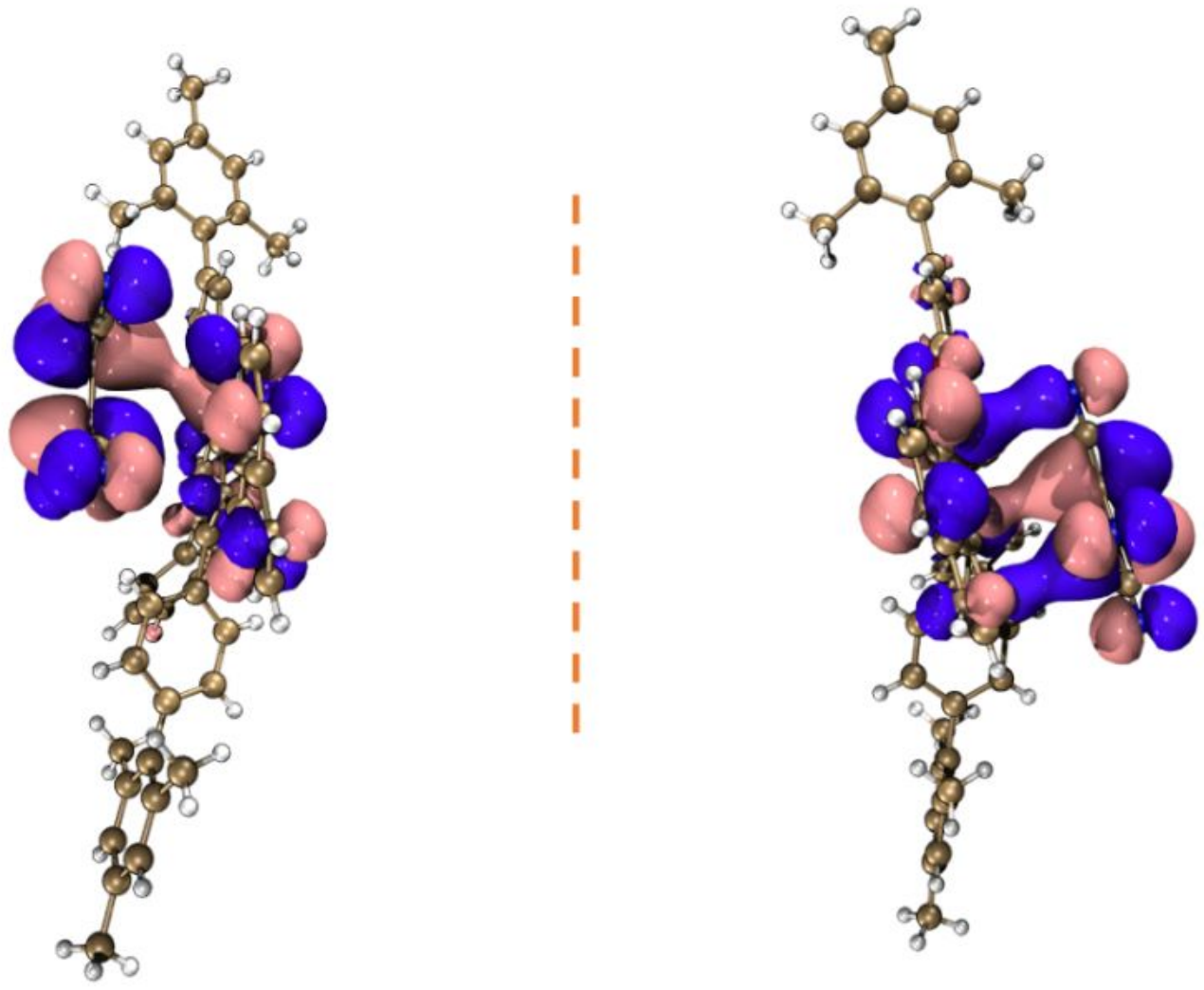

Figure S19. SOMO of the **OBP3·TCNE$^{\bullet}$** salt, obtained from single-point DFT calculations at the UPBE0/def2-SVP level based on the crystallographic geometry, highlighting two crystallographically inequivalent TCNE$^{\bullet-}$ sites (isovalue = 0.02).

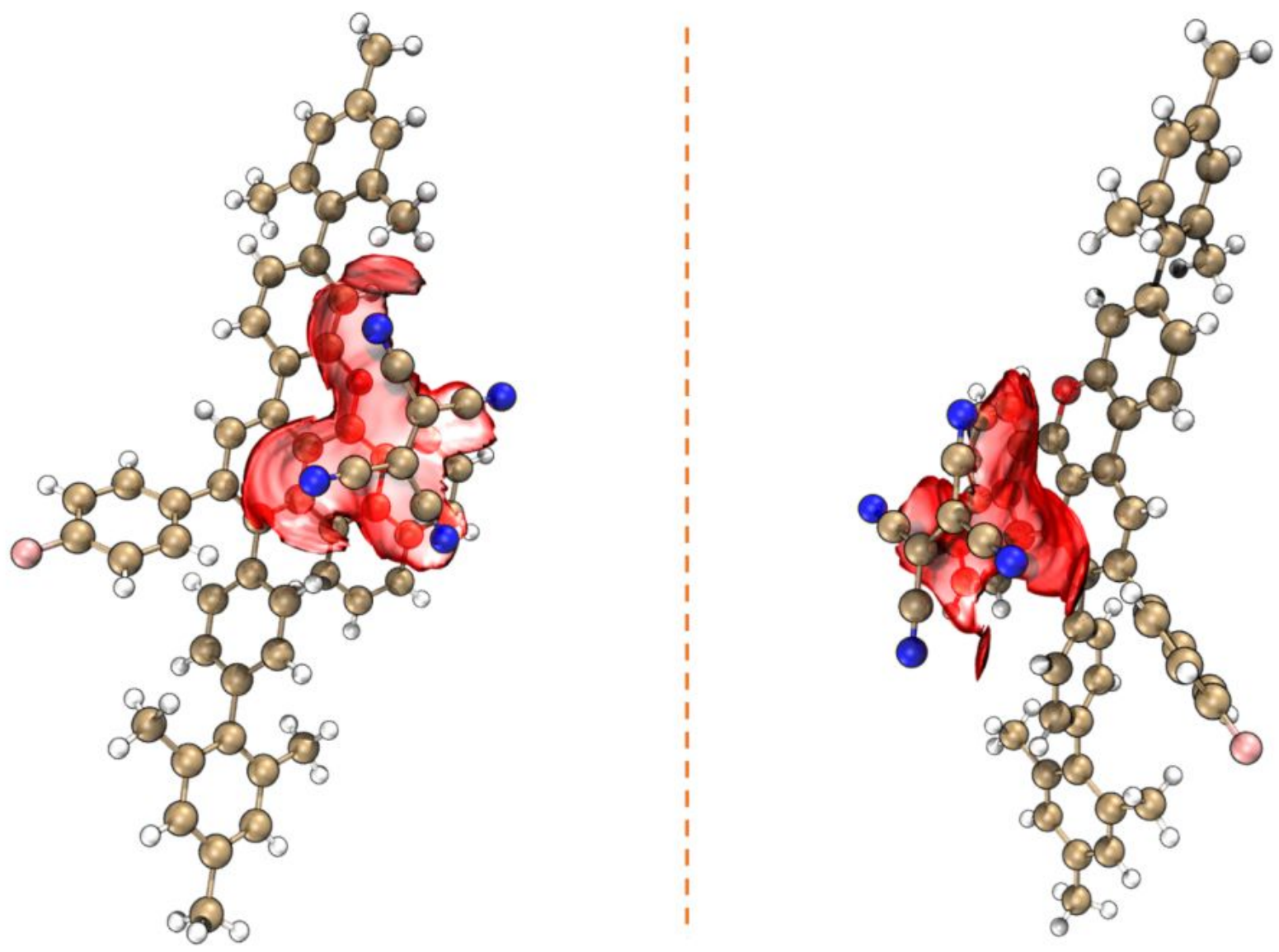

Figure S20. IGMH analysis of the **OBP3⁺** ion pair with two inequivalent $TCNE^{\bullet-}$ counteranions, revealing distinct noncovalent interaction patterns, shown as red isosurfaces (isovalue = 0.002 a.u.).

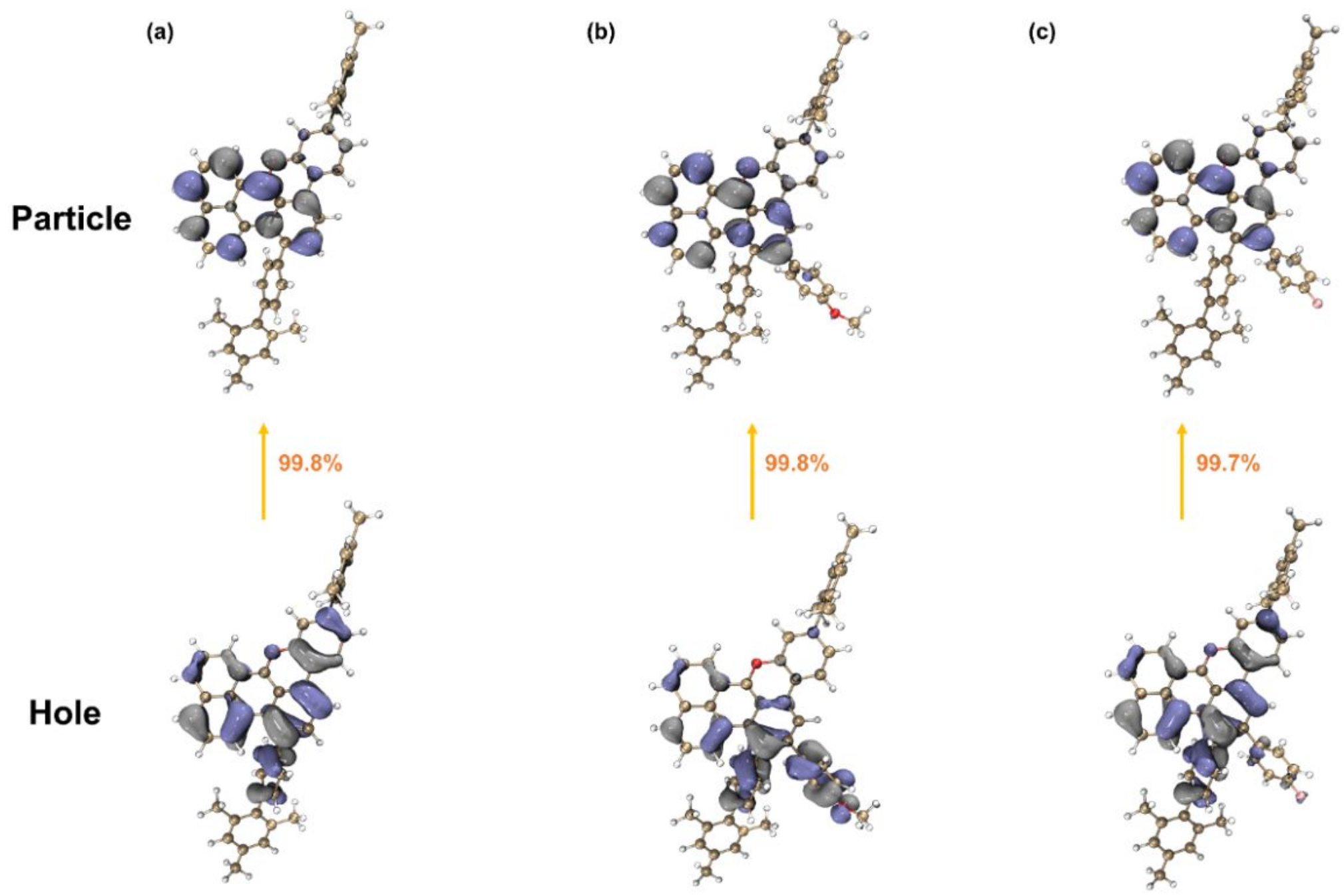

Figure S21. NTO representations (isovalue = 0.03) of the lowest-energy electronic excitations for (a) **OBP1⁺**, (b) **OBP2⁺**, and (c) **OBP3⁺**, obtained from TD-DFT calculations at the PBE0/def2-SVP level (SMD, CH_2Cl_2), based on the optimized S_0 geometries.

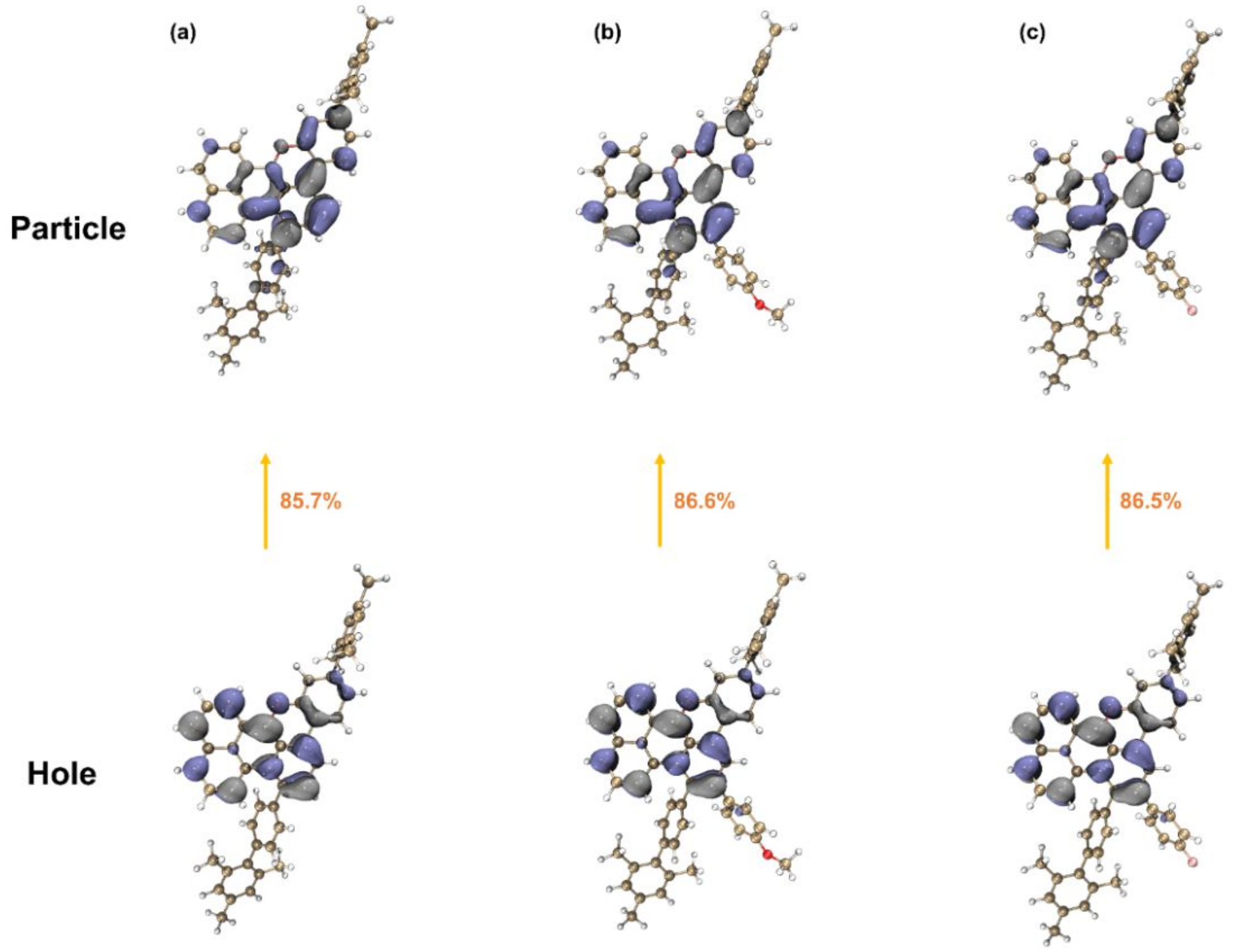

Figure S22. NTO representations (isovalue = 0.03) of the lowest-energy electronic excitations for (a) **OBP1**$^{\bullet}$, (b) **OBP2**$^{\bullet}$, and (c) **OBP3**$^{\bullet}$, obtained from TD-DFT calculations at the UPBE0/def2-SVP level (SMD, CH_2Cl_2), based on the optimized S_0 geometries.

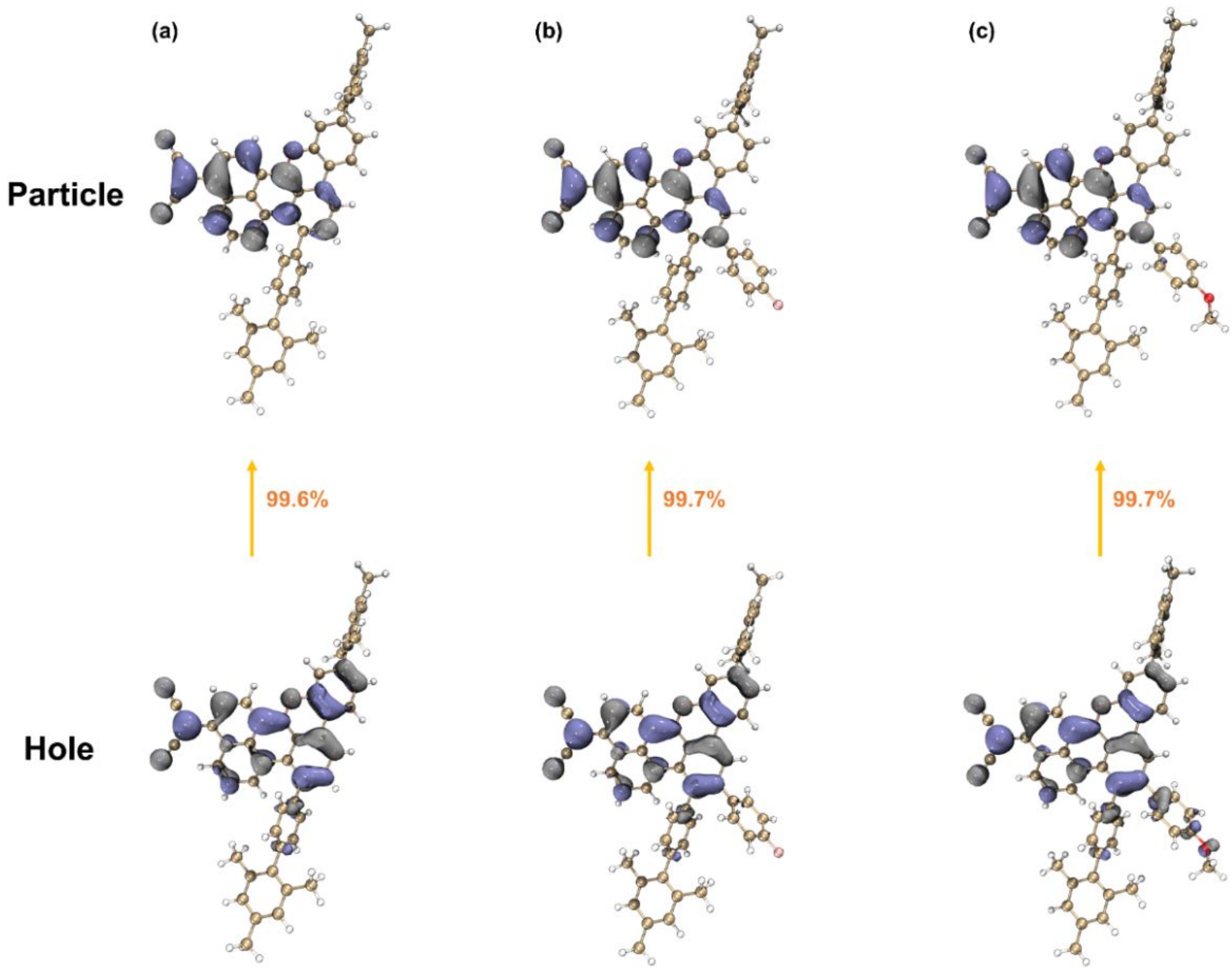

Figure S23. NTO representations (isovalue = 0.03) of the lowest-energy electronic excitations for (a) **A1**, (b) **A2**, and (c) **A3**, obtained from TD-DFT calculations at the PBE0/def2-SVP level (SMD, CH_2Cl_2), based on the optimized S_0 geometries.

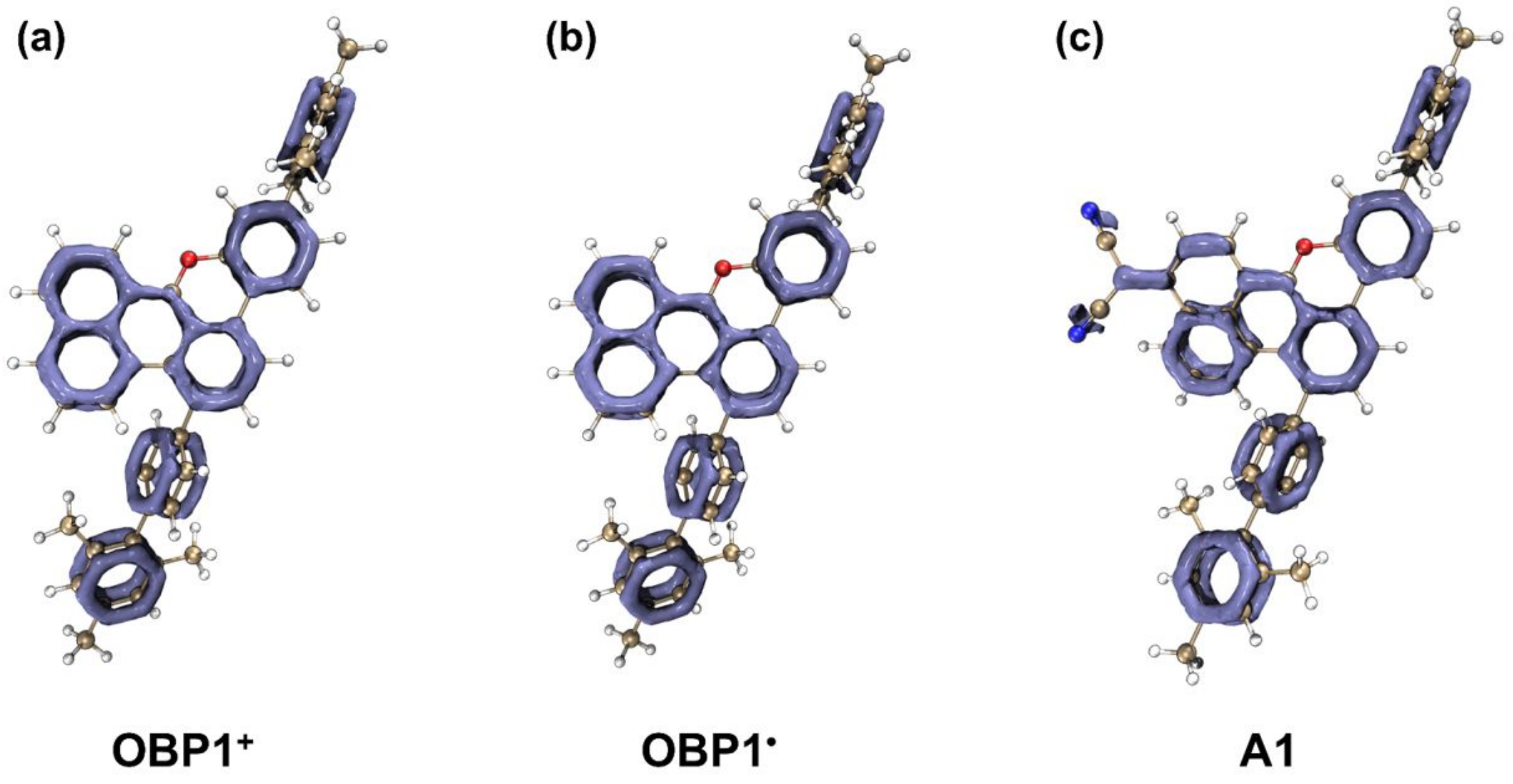

Figure S24. LOL-π isosurface maps of (a) **OBP1**$^{+}$, (b) **OBP1**$^{\bullet}$, and (c) **A1**, plotted at the same isovalue (0.5) for comparison.

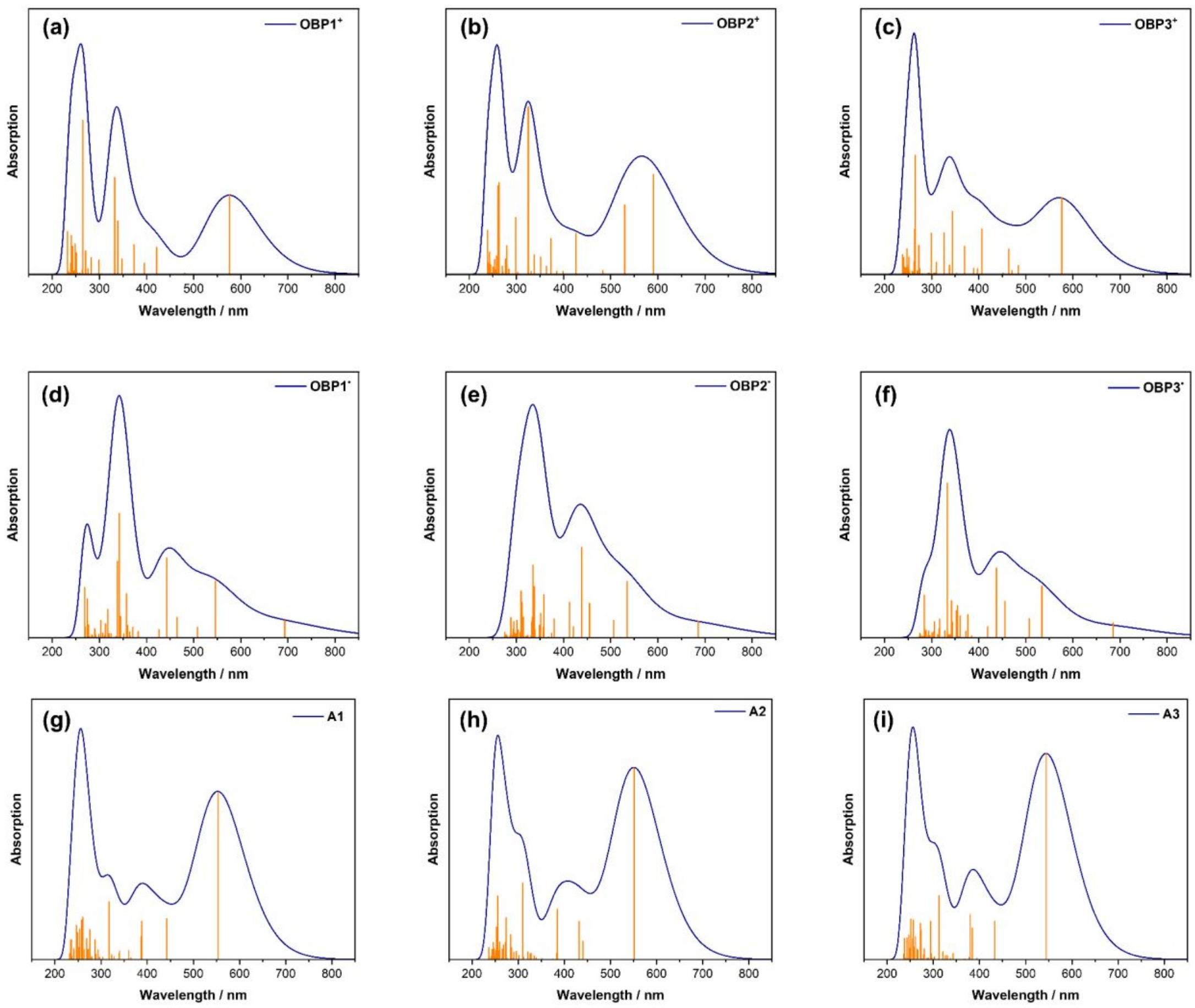

Figure S25. TD-DFT calculated UV–vis absorption spectra of (a–c) **OBP1**$^{+}$–**OBP3**$^{+}$, (d–f) **OBP1**$^{\bullet}$–**OBP3**$^{\bullet}$, and (g–i) **A1**–**A3**.

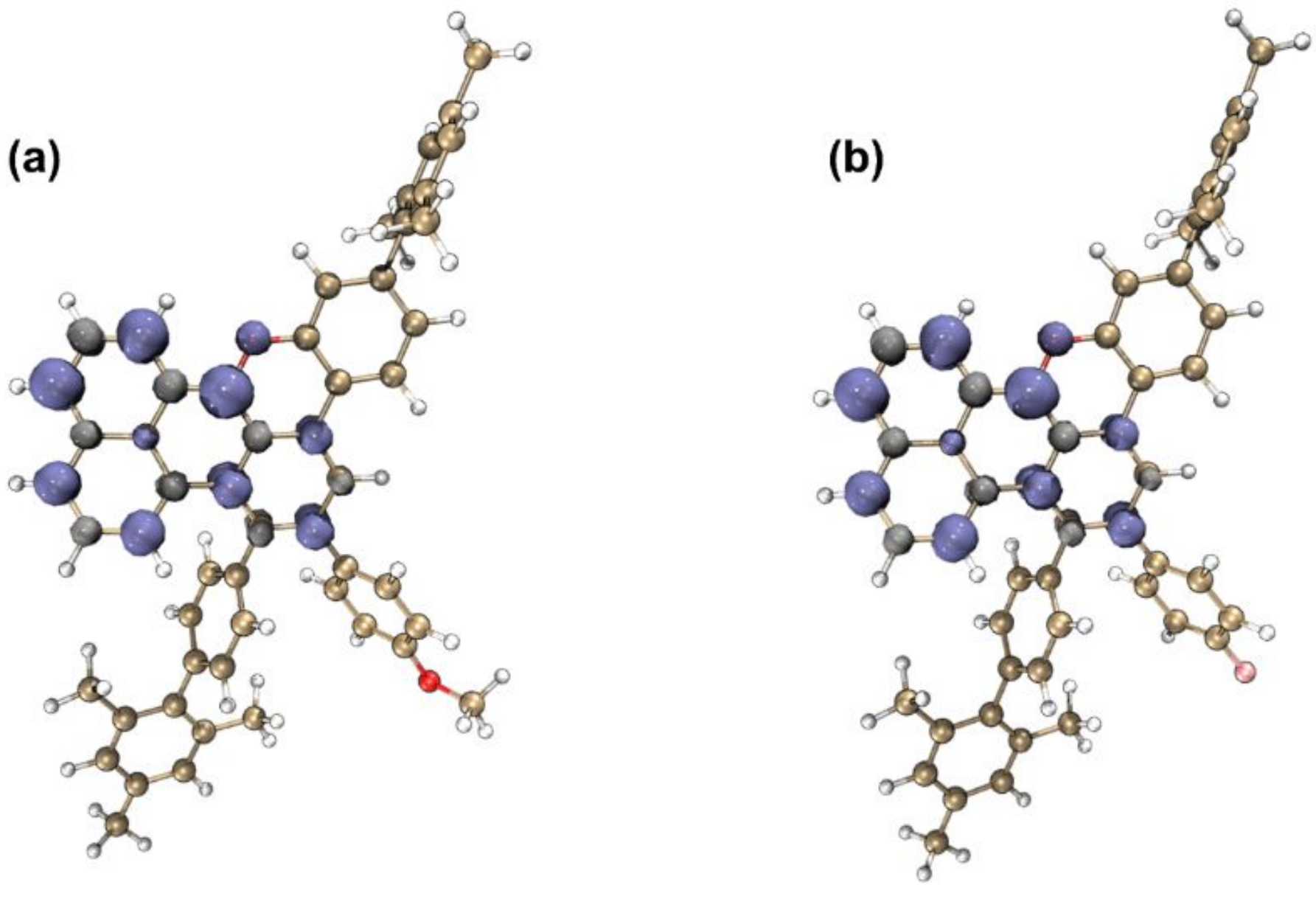

Figure S26. DFT-calculated spin density maps of (a) **OBP2**$^{\bullet}$ and (c) **OBP3**$^{\bullet}$ (isovalue = 0.005).

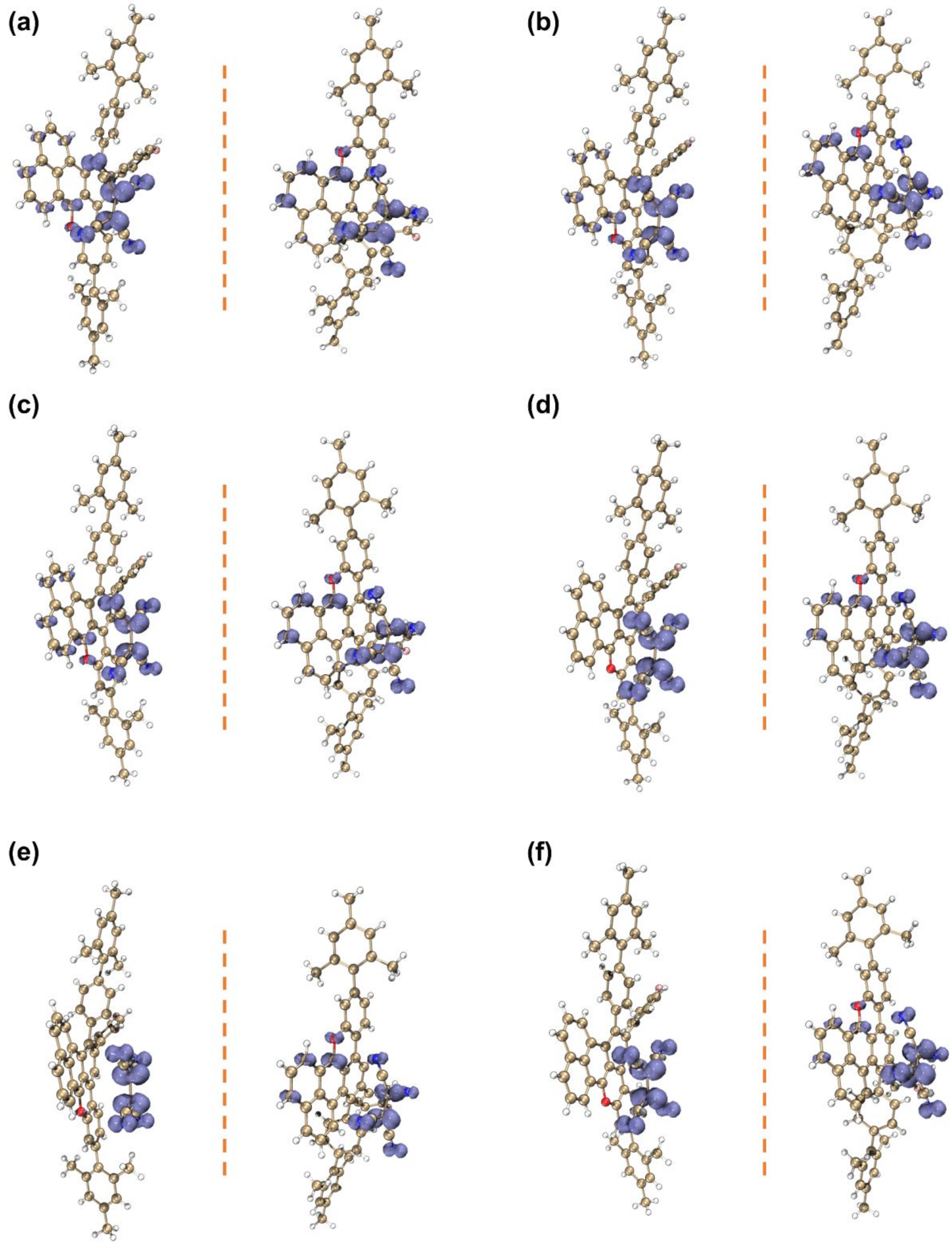

Figure S27. Spin density maps of the **OBP3·TCNE•** salt obtained from single-point DFT calculations on the single crystal structure using the def2-SVP basis set. Results for two crystallographically distinct segments of the 1D π-stacked chain are shown for (a) UPBE0, (b) UB3LYP, (c) UB3PW91, (d) UCAM-B3LYP, (e) UM06-2X, and (f) UωB97X-D. All isosurfaces are plotted at an identical value (0.005) to enable qualitative comparison of spin delocalization features across different exchange–correlation functionals.

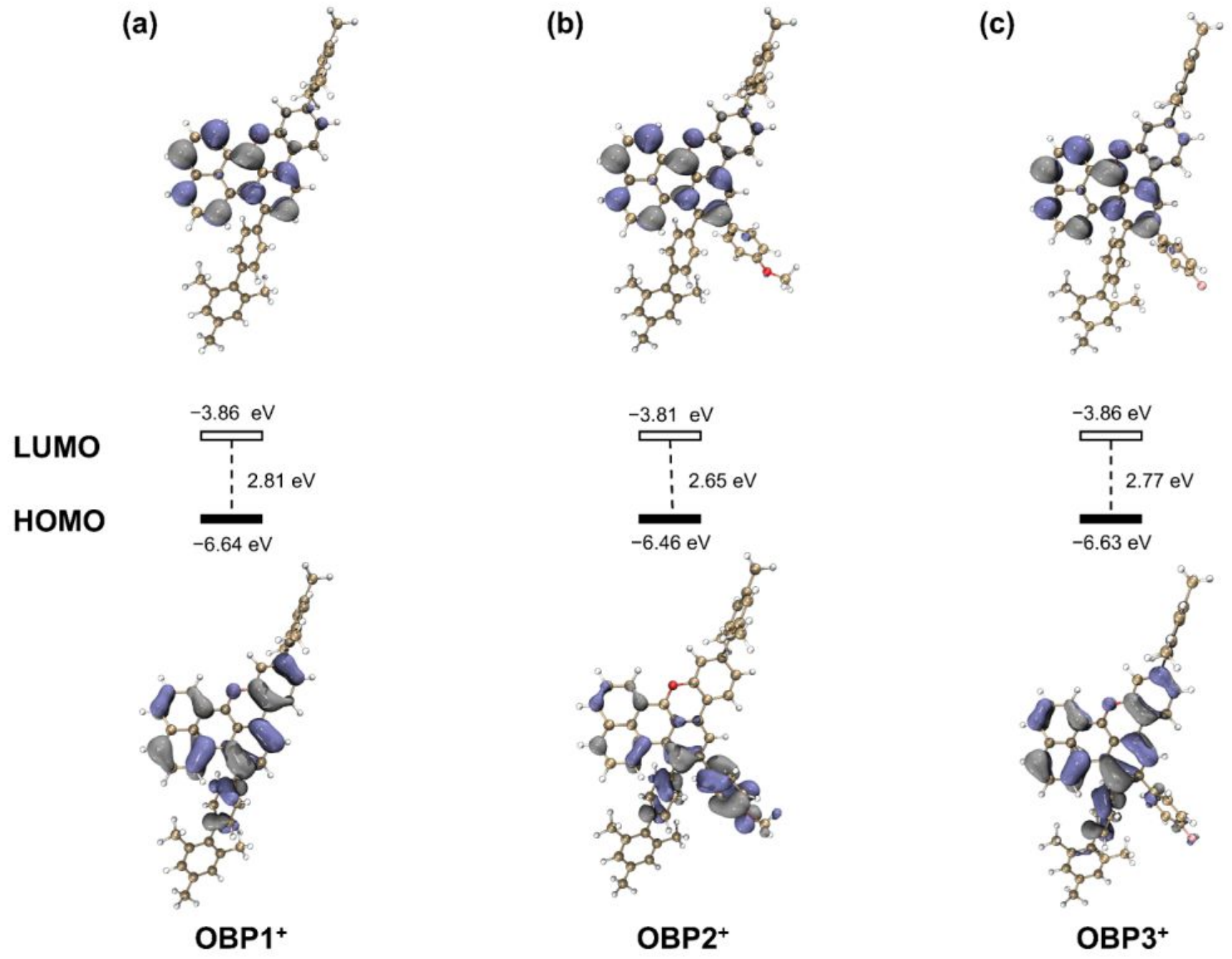

Figure S28. Frontier molecular orbital isosurfaces (isovalue = 0.03) and corresponding energy levels of (a) **OBP1**$^{+}$, (b) **OBP2**$^{+}$ and (c) **OBP3**$^{+}$ obtained from DFT calculations at PBE0/def2-SVP level, shown for reference.

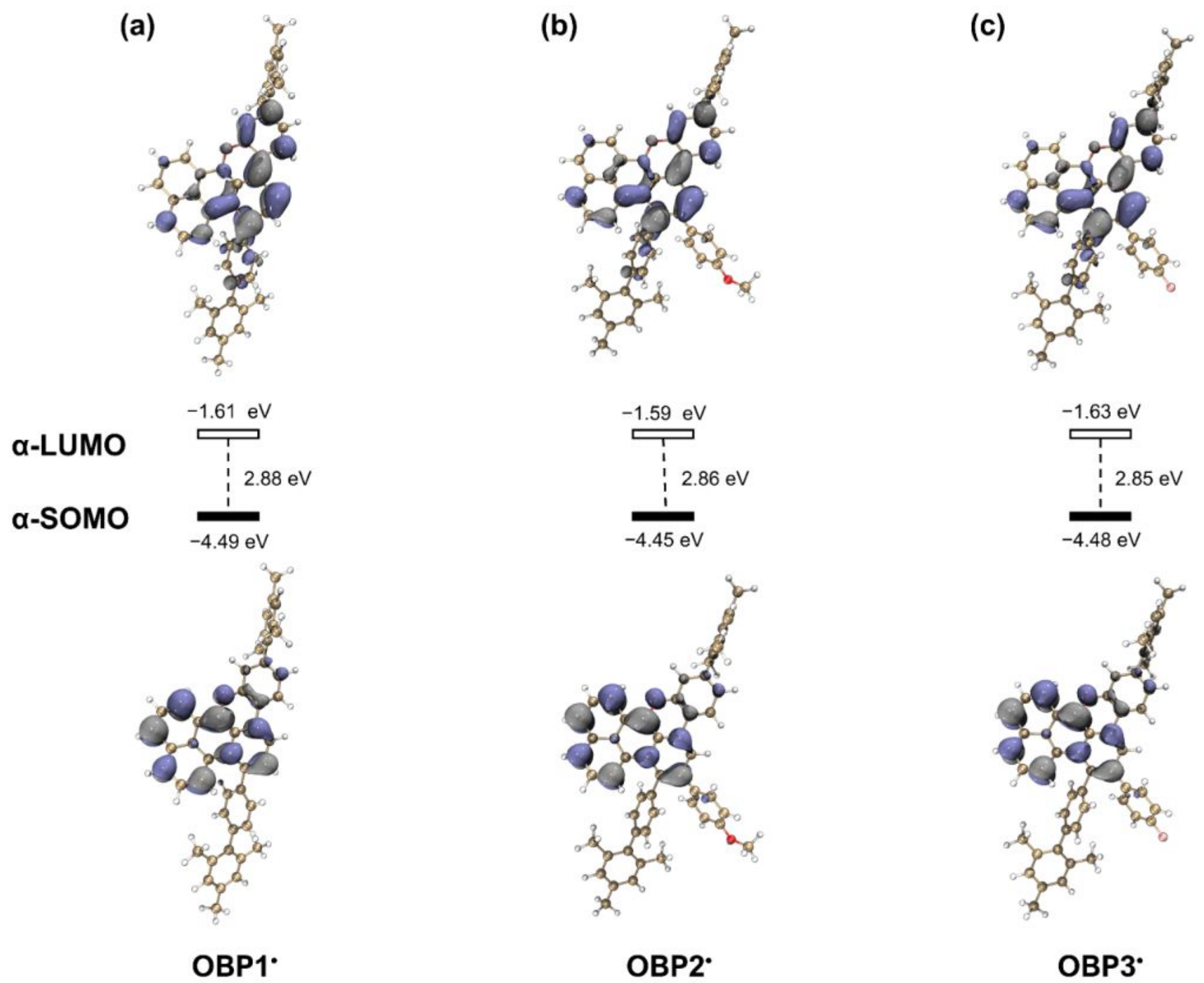

Figure S29. Frontier molecular orbital isosurfaces (isovalue = 0.03) and corresponding energy levels of (a) **OBP1**$^{\bullet}$, (b) **OBP2**$^{\bullet}$ and (c) **OBP3**$^{\bullet}$ obtained from DFT calculations at UPBE0/def2-SVP level, shown for reference.

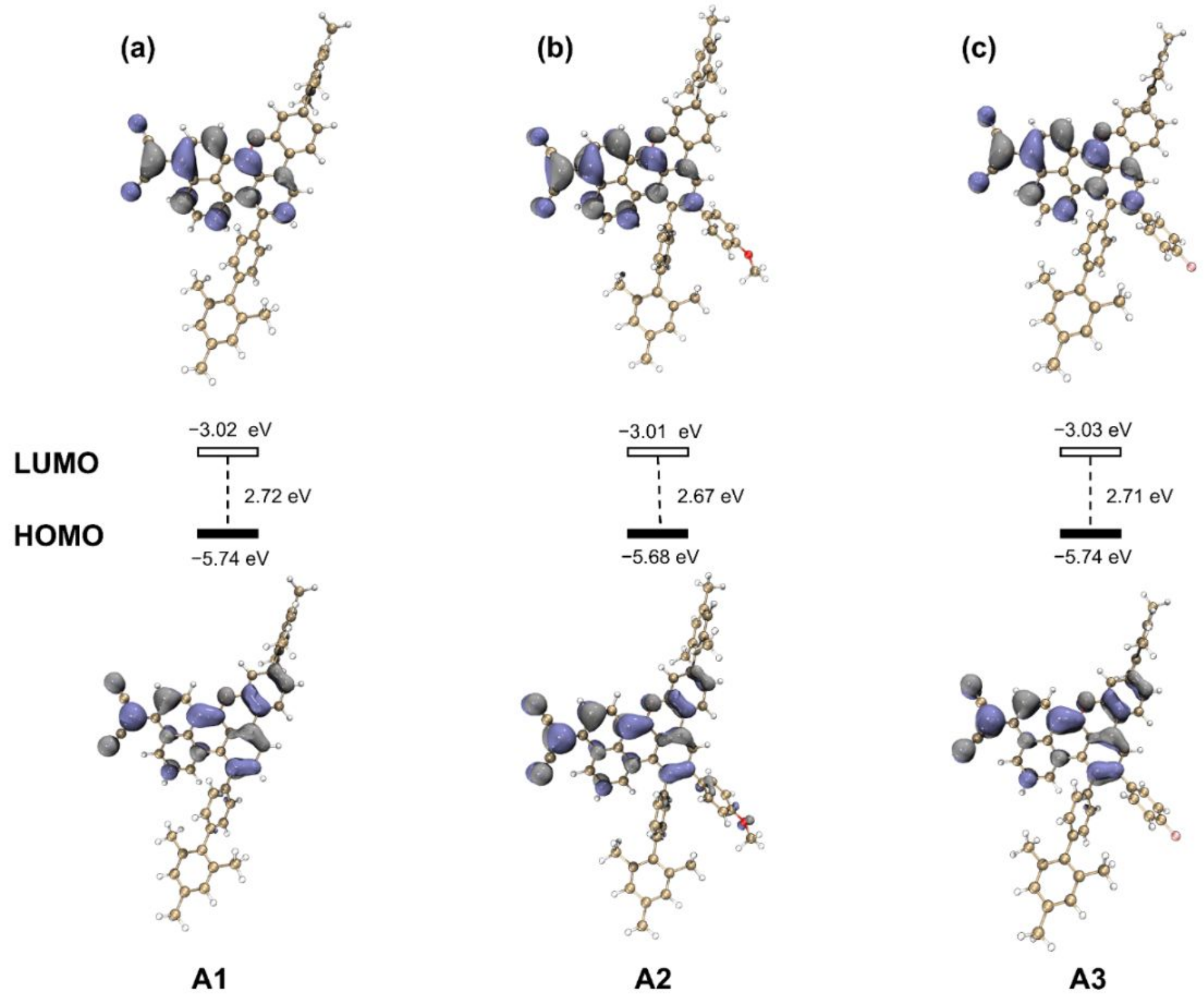

Figure S30. Frontier molecular orbital isosurfaces (isovalue = 0.03) and corresponding energy levels of (a) **A1**, (b) **A2** and (c) **A3** obtained from DFT calculations at PBE0/def2-SVP level, shown for reference.

Table S11. Optical predictions for **OBP1**$^+$ via TD-DFT at the PBE0/def2-TZVP level.

Exited state	Excitation energy / eV (Wavelength / nm)	Excitation amplitudes	Oscillator strength *f*
**1**	576.03	H → L (100%)	0.3305
**2**	480.78	H–1 → L (100%)	0.0051
**3**	471.69	H–2 → L (100%)	0.0001
**4**	452.80	H–3 → L (100%)	0.0000
**5**	449.64	H–4 → L (100%)	0.0012

Table S12. Optical predictions for **OBP2**$^+$ via TD-DFT at the PBE0/def2-TZVP level.

Exited state	Excitation energy / eV (Wavelength / nm)	Excitation amplitudes	Oscillator strength *f*
**1**	590.91	H–1 → L (3%)<br>H → L (97%)	0.3382
**2**	530.20	H–1 → L (97%)<br>H → L (3%)	0.2350
**3**	483.44	H–2 → L (100%)	0.0122
**4**	470.06	H–3 → L (100%)	0.0002

**5**	455.40	H–4 → L (100%)	0.0003

Table S13. Optical predictions for **OBP3$^+$** via TD-DFT at the PBE0/def2-TZVP level.

Exited state	Excitation energy / eV (Wavelength / nm)	Excitation amplitudes	Oscillator strength *f*
**1**	570.78	H → L (100%)	0.3297
**2**	491.06	H–5 → L (5%)<br>H–1 → L (95%)	0.0288
**3**	477.72	H–2 → L (100%)	0.0093
**4**	467.31	H–5 → L (70%)<br>H–4 → L (4%)<br>H–3 → L (22%)<br>H–1 → L (4%)	0.1212
**5**	460.08	H–5 → L (17%)<br>H–4 → L (3%)<br>H–3 → L (80%)	0.0215

Table S14. Optical predictions for **A1** via TD-DFT at the PBE0/def2-TZVP level.

Exited state	Excitation energy / eV (Wavelength / nm)	Excitation amplitudes	Oscillator strength *f*
**1**	553.72	H–1 → L (2%)<br>H → L (98%)	0.8323
**2**	442.02	H–1 → L (98%)<br>H → L (2%)	0.2048
**3**	388.07	H–6 → L (3%)<br>H–2 → L (29%)<br>H → L+1 (68%)	0.1906
**4**	386.55	H–2 → L (71%)<br>H → L+1 (29%)	0.1156
**5**	379.41	H–3 → L (100%)	0.0002

Table S15. Optical predictions for **A2** via TD-DFT at the PBE0/def2-TZVP level.

Exited state	Excitation energy / eV (Wavelength / nm)	Excitation amplitudes	Oscillator strength *f*
1	551.39	H → L (100%)	0.9530
2	440.14	H–1 → L (100%)	0.0920
3	431.90	H–2 → L (97%)<br>H → L (3%)	0.1899
4	385.21	H–3 → L (9%)<br>H–2 → L (4%)<br>H–1 → L+1 (87%)	0.2516
5	383.36	H–6 → L (90%)	0.0320

		H–5 → L (10%)	

Table S16. Optical predictions for **A3** via TD-DFT at the PBE0/def2-TZVP level.

Exited state	Excitation energy / eV (Wavelength / nm)	Excitation amplitudes	Oscillator strength *f*
1	544.51	H–1 → L (2%)<br>H → L (97%)	0.9508
2	432.28	H–1 → L (97%)<br>H → L (3%)	0.1770
3	394.01	H–6 → L (7%)<br>H–5 → L (32%)<br>H–4 → L (5%)<br>H–2 → L (32%)<br>H–1 → L (4%)<br>H → L+1 (19%)	0.0044
4	384.85	H–2 → L (48%)<br>H → L+1 (52%)	0.1464
5	379.67	H–6 → L (71%)<br>H–5 → L (34%)<br>H–4 → L (7%)<br>H–3 → L (6%)<br>H–2 → L (20%)<br>H → L+1 (2%)	0.2068

Table S17. Optical predictions for **OBP1**$^{\bullet}$ via TD-DFT at the UPBE0/def2-TZVP level. For the open-shell species, H(A) corresponds to the α-HOMO (SOMO), and L(A) denotes the α-LUMO; L(B) represents the spin counterpart of the SOMO.

Exited state	Excitation energy / eV (Wavelength / nm)	Excitation amplitudes	Oscillator strength *f*
**1**	693.38	H(A) → L(A) (89%)<br>H–1(B) → L(B) (10%)	0.0391
**2**	546.45	H–1(A) → L(A) (5%)<br>H(A) → L(A) (8%)<br>H(A) → L+1(A) (2%)<br>H(A) → L+2(A) (10%)<br>H(B) → L(B) (71%)<br>H(B) → L+1(A) (4%)	0.1276
**3**	508.30	H(A) → L+1(A) (9%)<br>H–1(A) → L(A) (2%)<br>H(A) → L+3(A) (73%)<br>H(B) → L+1(B) (1%)<br>H-9(B) → L(B) (4%)	0.0234

		H(A) → L(A) (4%)<br>H(A) → L+2(A) (7%)	

Table S17. Optical predictions for **OBP1•** via TD-DFT at the UPBE0/def2-TZVP level. For the open-shell species, H(A) corresponds to the *α*-HOMO (SOMO), and L(A) denotes the *α*-LUMO; L(B) represents the spin counterpart of the SOMO (Continued).

**4**	465.01	H–1(A) → L(A) (15%)<br>H(A) → L(A) (2%)<br>H(A) → L+1(A) (25%)<br>H(A) → L+3(A) (23%)<br>H(A) → L+5(A) (4%)<br>H–6(B) → L(B) (3%)<br>H(B) → L(B) (7%)<br>H(B) → L+1(B) (20%)	0.0455
**5**	442.33	H–1(A) → L(A) (3%)<br>H(A) → L(A) (2%)<br>H(A) → L+1(A) (1%)<br>H(A) → L+2(A) (42%)<br>H(A) → L+3(A) (19%)<br>H(A) → L+5(A) (3%)<br>H–9(B) → L(B) (1%)<br>H–8(B) → L(B) (1%)<br>H–7(B) → L(B) (2%)<br>H–5(B) → L(B) (6%)<br>H(B) → L(B) (13%)<br>H(B) → L+1(B) (5%)	0.1799

Table S18. Optical predictions for **OBP2•** via TD-DFT at the UPBE0/def2-TZVP level. For the open-shell species, H(A) corresponds to the *α*-HOMO (SOMO), and L(A) denotes the *α*-LUMO; L(B) represents the spin counterpart of the SOMO.

Exited state	Excitation energy / eV (Wavelength / nm)	Excitation amplitudes	Oscillator strength *f*
**1**	685.17	H(A) → L(A) (90%)<br>H(B) → L(B) (10%)	0.0375
**2**	535.25	H–1(A) → L(A) (4%)<br>H(A) → L(A) (9%)<br>H(A) → L+1(A) (4%)<br>H(A) → L+2(A) (10%)	0.1269

		H–1(B) → L(B) (1%)<br>H(B) → L(B) (68%)<br>H(B) → L+1(B) (4%)	

Table S18. Optical predictions for **OBP2•** via TD-DFT at the UPBE0/def2-TZVP level. For the open-shell species, H(A) corresponds to the *α*-HOMO (SOMO), and L(A) denotes the *α*-LUMO; L(B) represents the spin counterpart of the SOMO (Continued).

**3**	506.51	H–2(A) → L(A) (1%)<br>H–1(A) → L(A) (6%)<br>H(A) → L+1(A) (76%)<br>H(A) → L+2(A) (5%)<br>H(A) → L+3(A) (3%)<br>H–9(B) → L(B) (3%)<br>H–1(B) → L(B) (2%)<br>H(B) → L+1(B) (4%)	0.0393
**4**	455.19	H–2(A) → L(A) (2%)<br>H–1(A) → L(A) (14%)<br>H(A) → L(A) (3%)<br>H(A) → L+1(A) (18%)<br>H(A) → L+3(A) (22%)<br>H(A) → L+4(A) (2%)<br>H(A) → L+5(A) (2%)<br>H(A) → L+8(A) (2%)<br>H–7(B) → L(B) (3%)<br>H–1(B) → L+1(B) (1%)<br>H(B) → L(B) (10%)<br>H(B) → L+1(B) (20%)	0.0773
**5**	438.11	H–1(A) → L(A) (1%)<br>H(A) → L(A) (2%)<br>H(A) → L+1(A) (2%)<br>H(A) → L+2(A) (45%)<br>H(A) → L+3(A) (19%)<br>H(A) → L+5(A) (4%)<br>H–10(B) → L(B) (2%)<br>H–8(B) → L(B) (2%)<br>H–6(B) → L(B) (7%)<br>H(B) → L(B) (2%) (14%)<br>H(B) → L+1(B) (3%)	0.2033

Table S19. Optical predictions for **OBP3**$^{\bullet}$ via TD-DFT at the UPBE0/def2-TZVP level. For the open-shell species, H(A) corresponds to the *α*-HOMO (SOMO), and L(A) denotes the *α*-LUMO; L(B) represents the spin counterpart of the SOMO.

Exited state	Excitation energy / eV (Wavelength / nm)	Excitation amplitudes	Oscillator strength *f*
1	685.53	H(A) → L(A) (89%)<br>H(B) → L(B) (11%)	0.0358
2	533.96	H–1(A) → L(A) (4%)<br>H(A) → L(A) (9%)<br>H(A) → L(A) (6%)<br>H(A) → L+2(A) (9%)<br>(H–2–L) (68%)<br>(H–L1) (4%)	0.1264
3	506.98	H–1(A) → L(A) (6%)<br>H(A) → L+1(A) (76%)<br>H(A) → L+2(A) (7%)<br>H(A) → L+3(A) (1%)<br>H(A) → L+4(A) (1%)<br>H–10(B) → L(B) (2%)<br>H–6(B) → L(B) (3%)<br>H(B) → L+1(B) (4%)	0.0462
	455.31	H(B) → L+1(B) (24%)<br>H–1(A) → L(A) (18%)<br>H–1 (A) → L+1(A) (16%)<br>H(A) → L+4(A) (15%)<br>H(B) → L(B) (12%)<br>H(A) → L+3(A) (5%)<br>H(A) → L(A) (3%)<br>H–7(B) → L(B) (2%)<br>H(A) → L+6(A) (2%)<br>H–6(B) → L(B) (2%)<br>H(A) → L+7(A) (2%)	0.0884
5	419.15	H(A) → L+2(A) (45%)<br>H(A) → L+4(A) (16%)<br>H(B) → L(B) (12%)<br>H–6(B) → L(B) (10%)<br>H(A) → L+3(A) (6%)<br>H(A) → L+5(A) (3%)<br>H(B) → L+1(B) (2%)	0.0267

	H(A) → L+6(A) (2%)
	H(A) → L(A) (2%)
	H(A) → L+1(A) (1%)
	H–1(A) → L(A) (1%)

6. NMR spectra of synthesized compounds

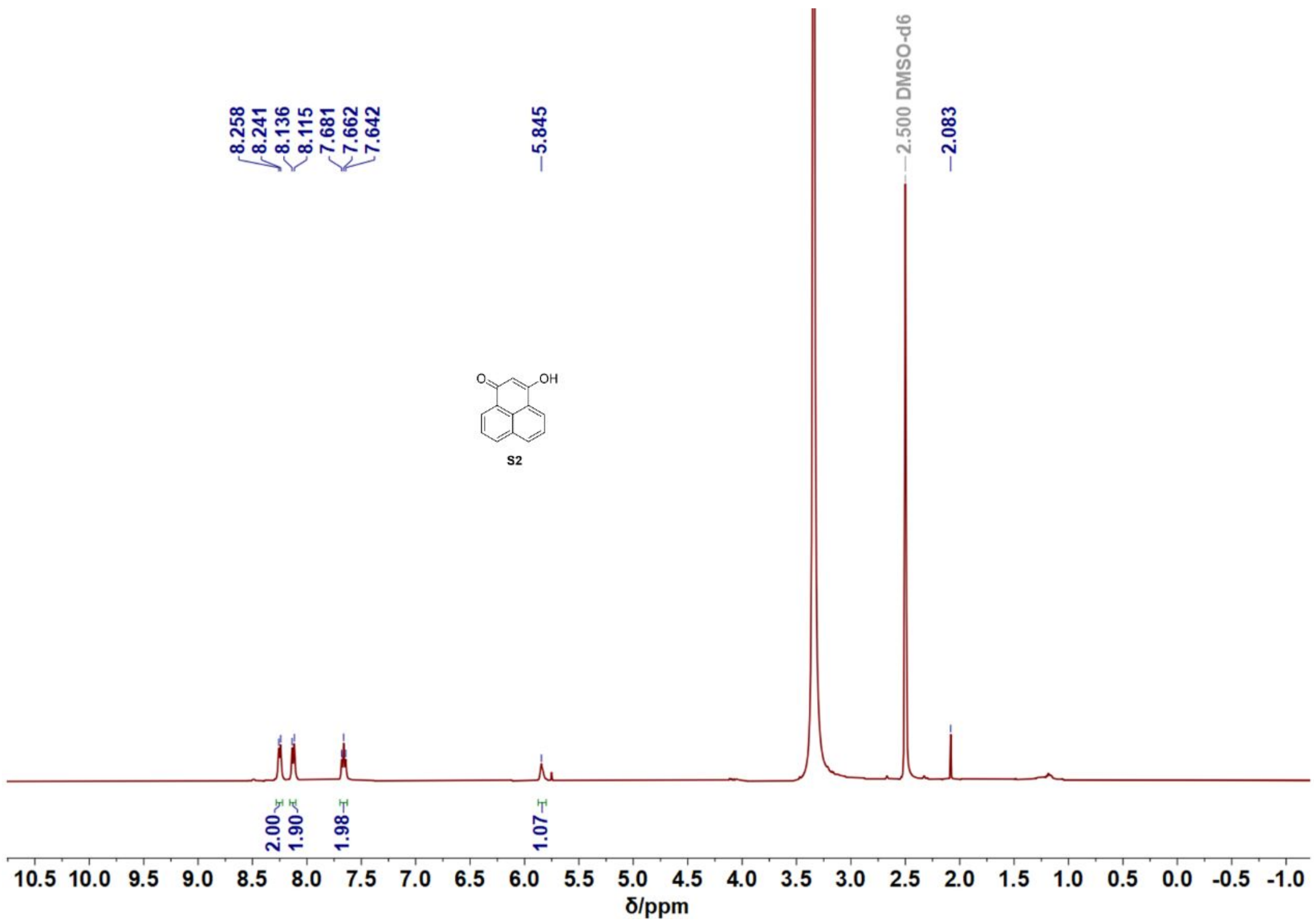

Figure S31. ^{1}H NMR spectrum of **S2** in DMSO-d_6 (400 MHz, 297 K).

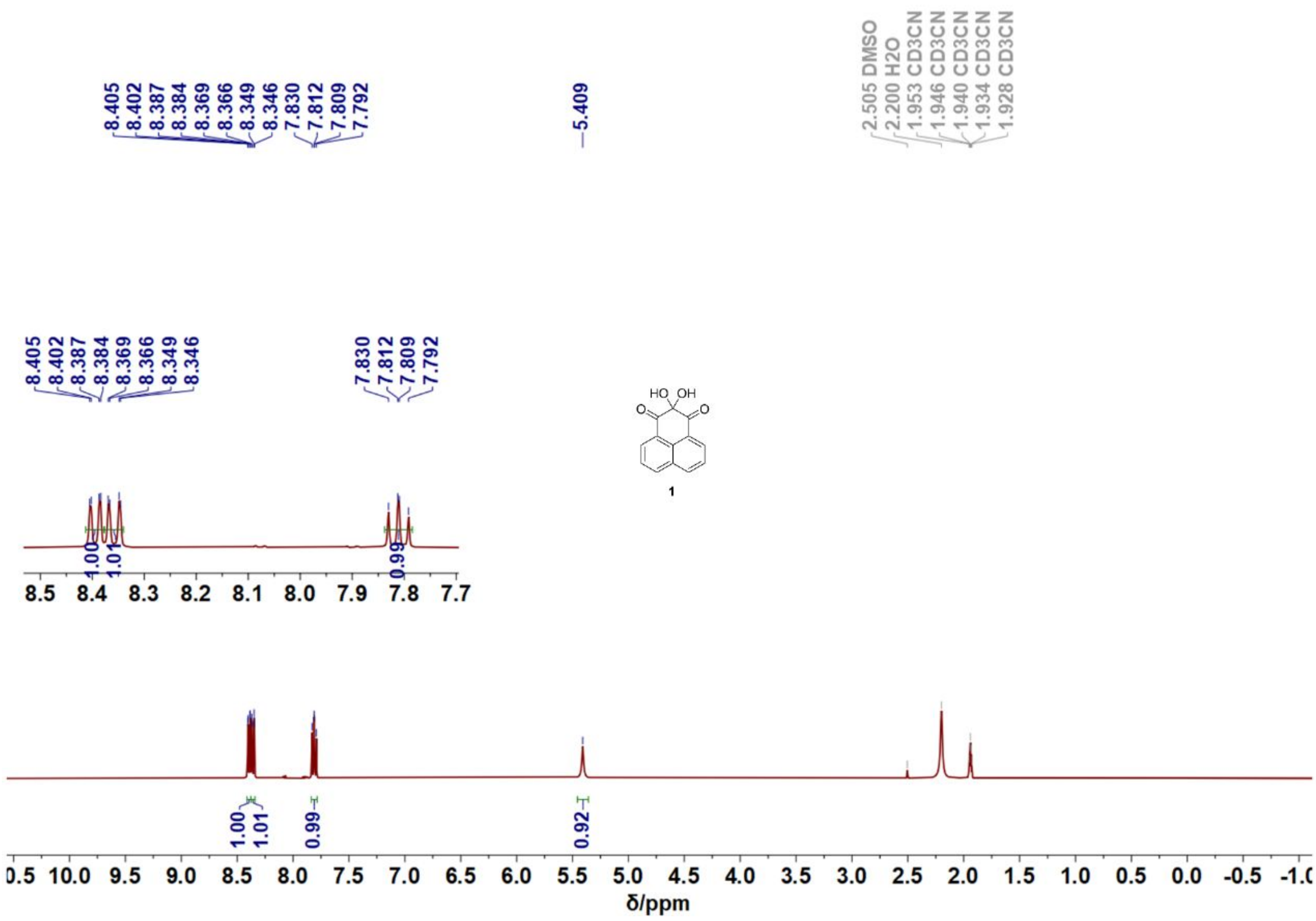

Figure S32. ^{1}H NMR spectrum of **1** in acetonitrile-d_3 (400 MHz, 297 K).

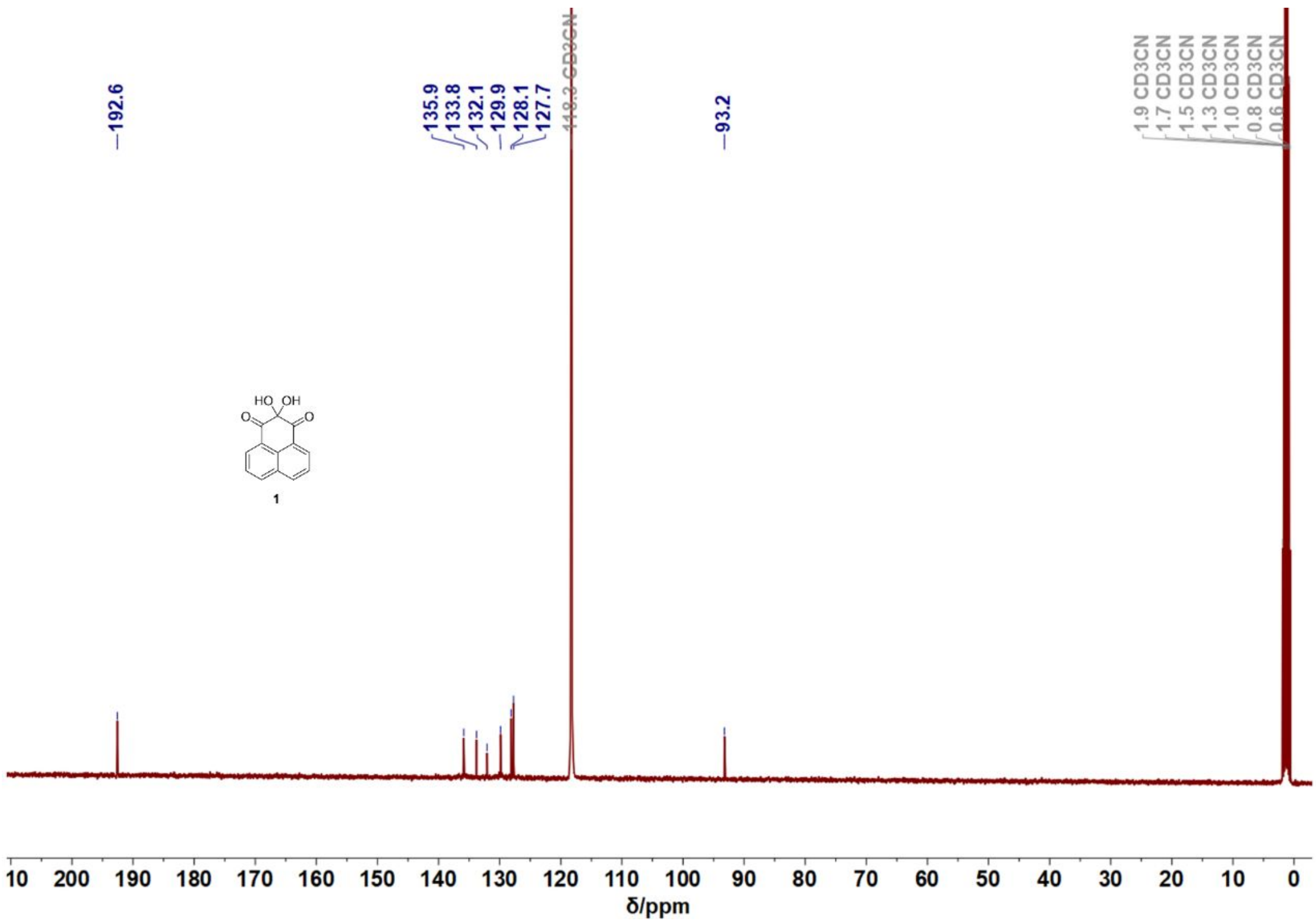

Figure S33. ^{13}C NMR spectrum of **1** in acetonitrile-d_3 (101 MHz, 297 K).

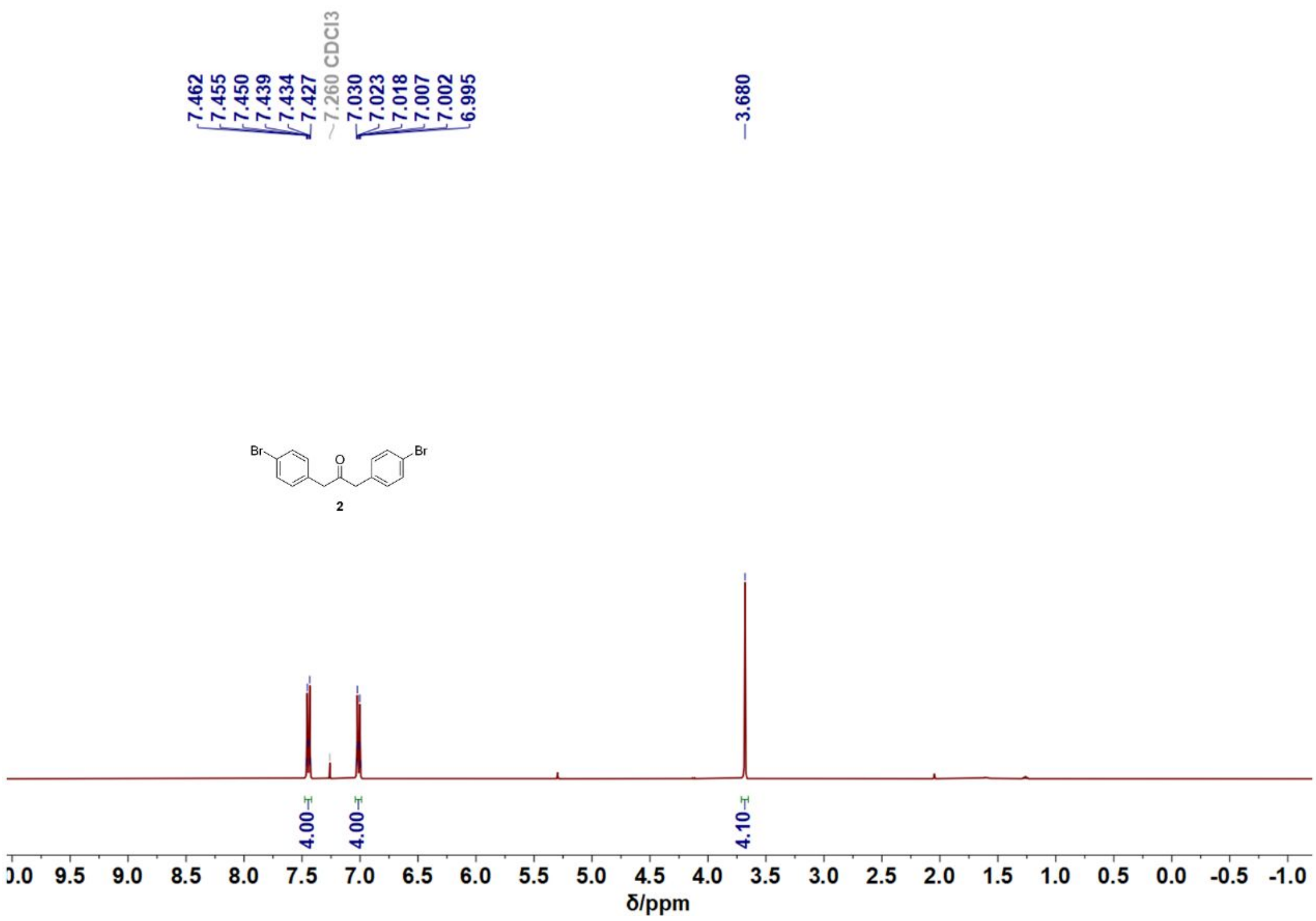

Figure S34. ^{1}H NMR spectrum of **2** in $CDCl_3$ (400 MHz, 297 K).

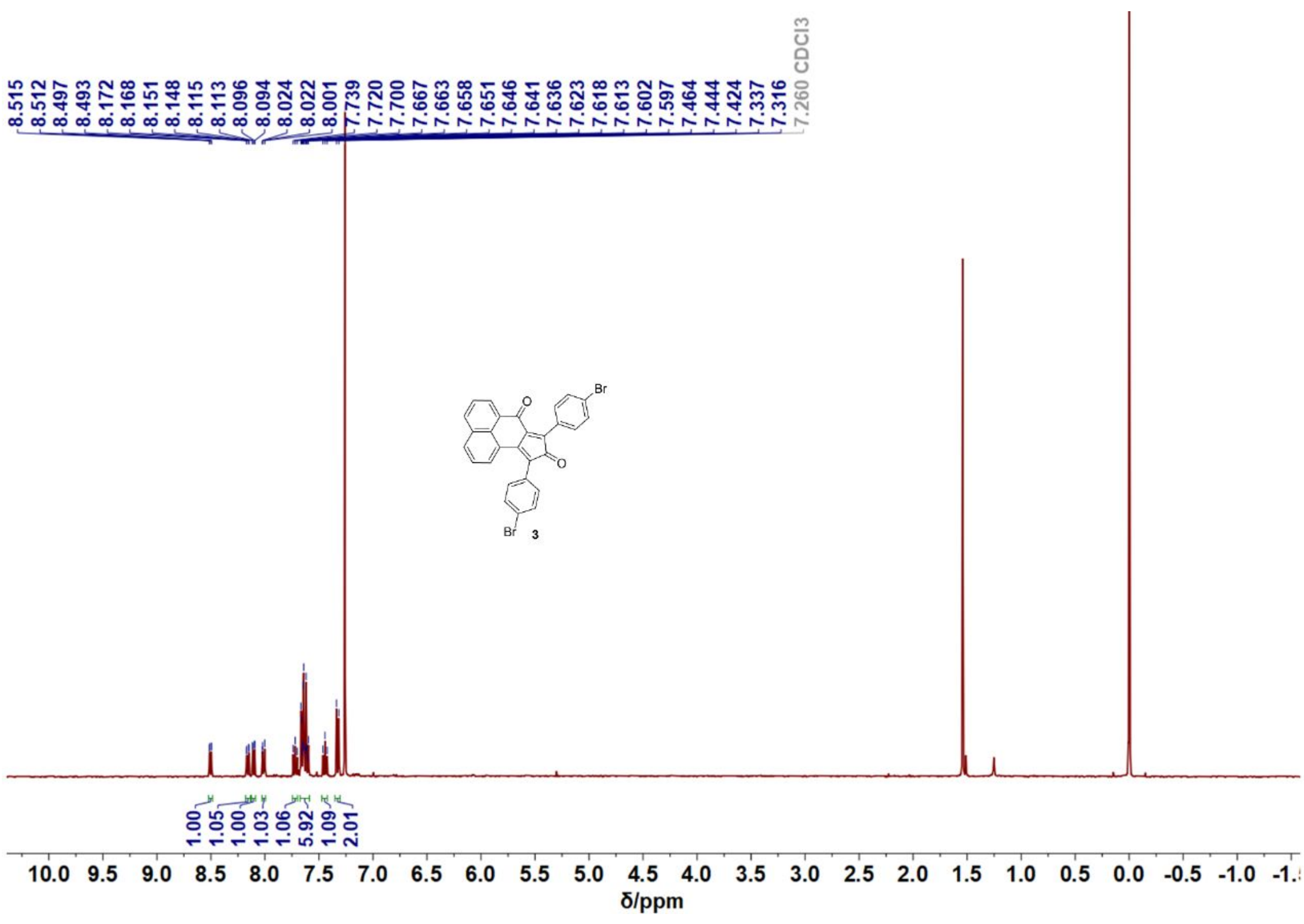

Figure S35. ^{1}H NMR spectrum of **3** in $CDCl_3$ (400 MHz, 297 K).

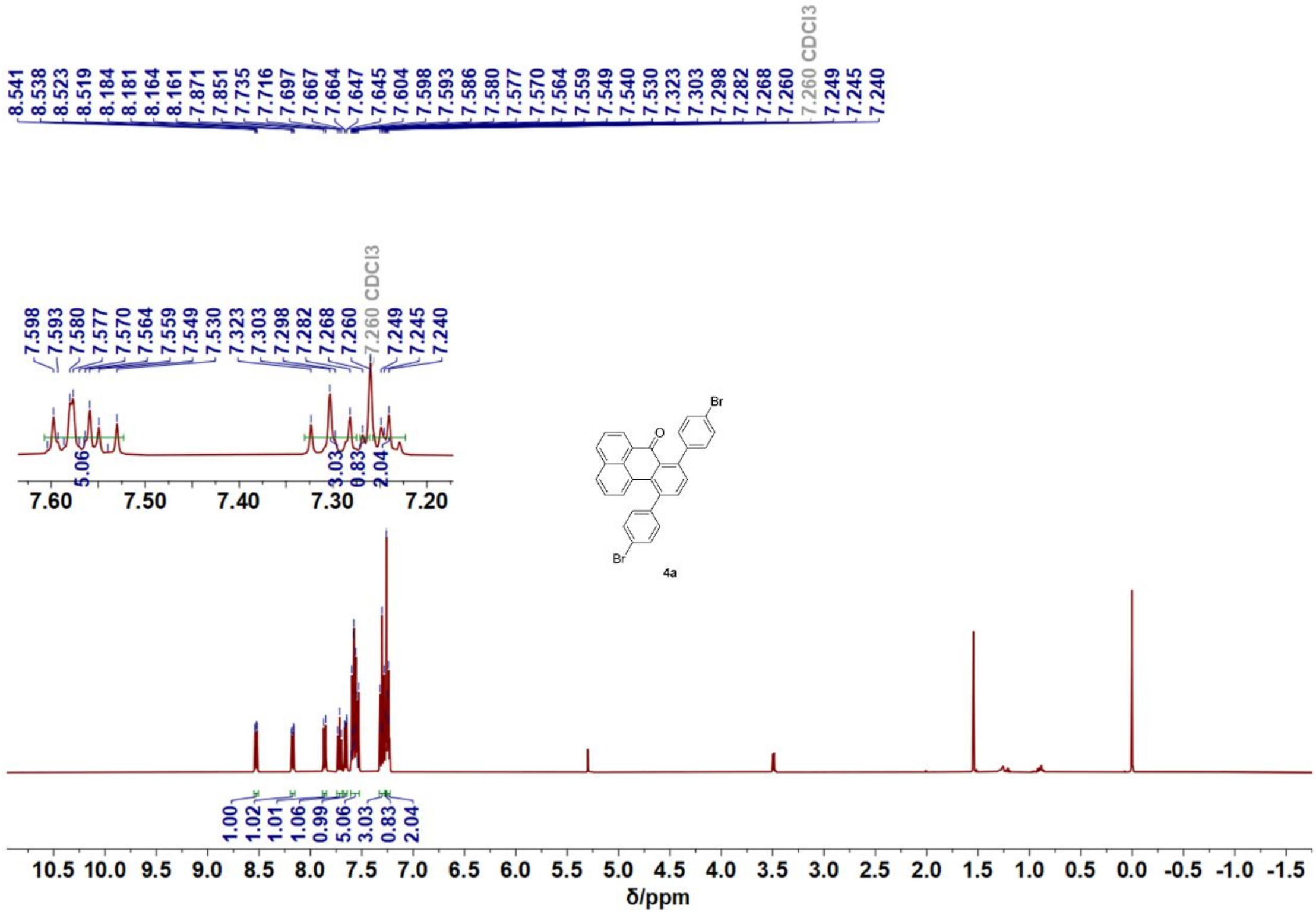

Figure S36. ^{1}H NMR spectrum of **4a** in $CDCl_3$ (400 MHz, 297 K).

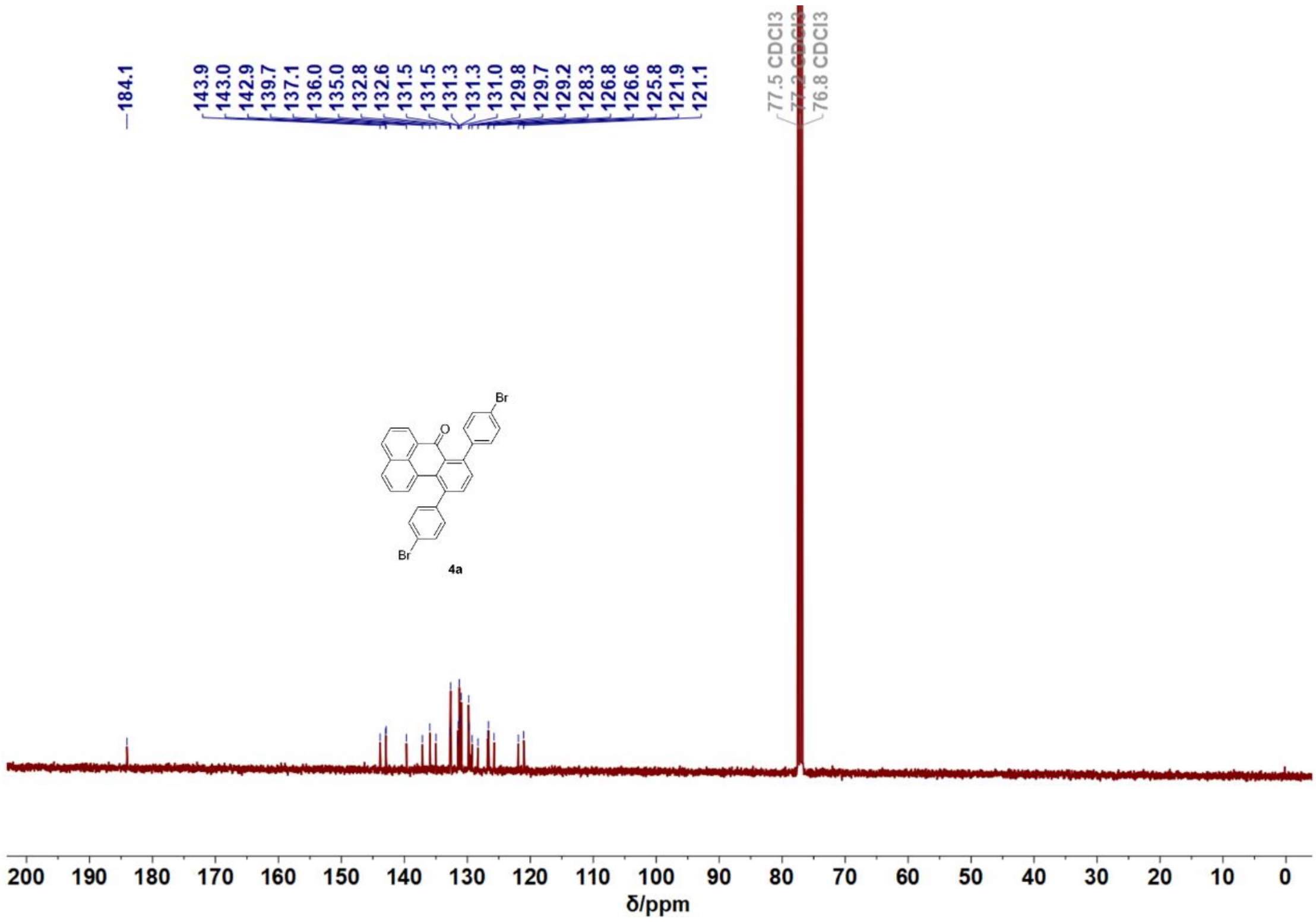

Figure S37. ^{13}C NMR spectrum of **4a** in $CDCl_3$ (101 MHz, 297 K).

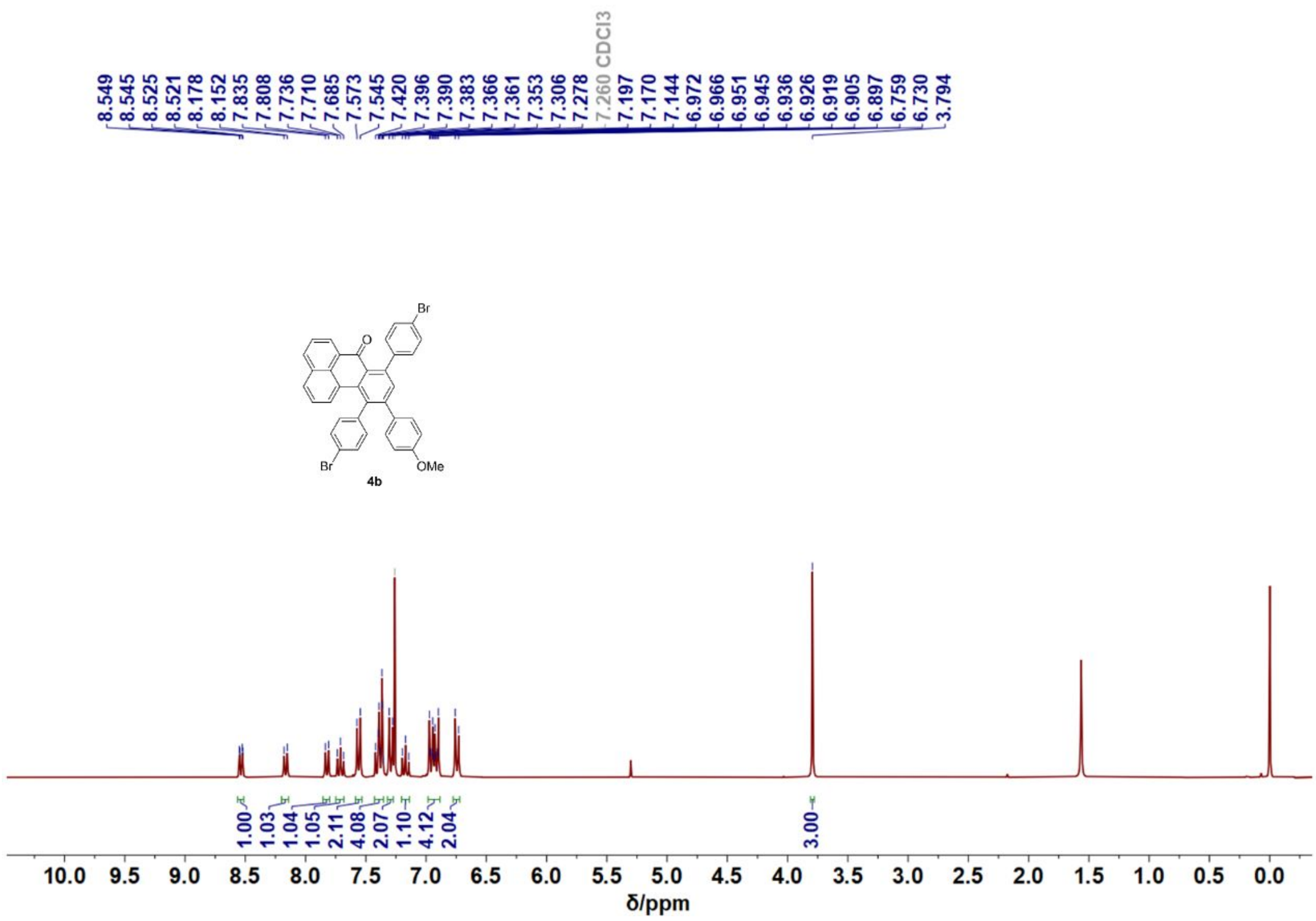

Figure S38. ^{1}H NMR spectrum of **4b** in $CDCl_3$ (300 MHz, 297 K).

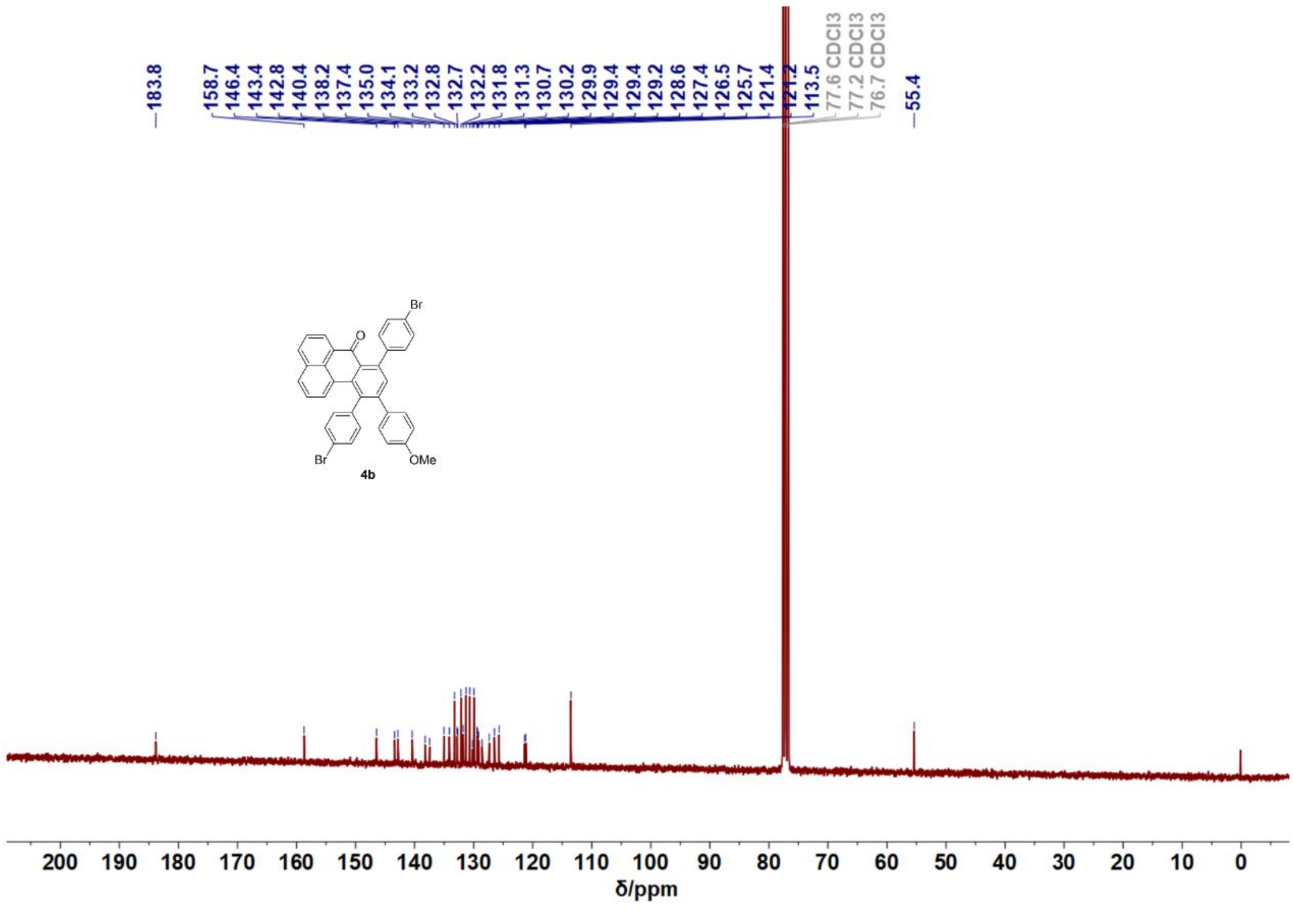

Figure S39. ^{13}C NMR spectrum of **4b** in $CDCl_3$ (75 MHz, 297 K).

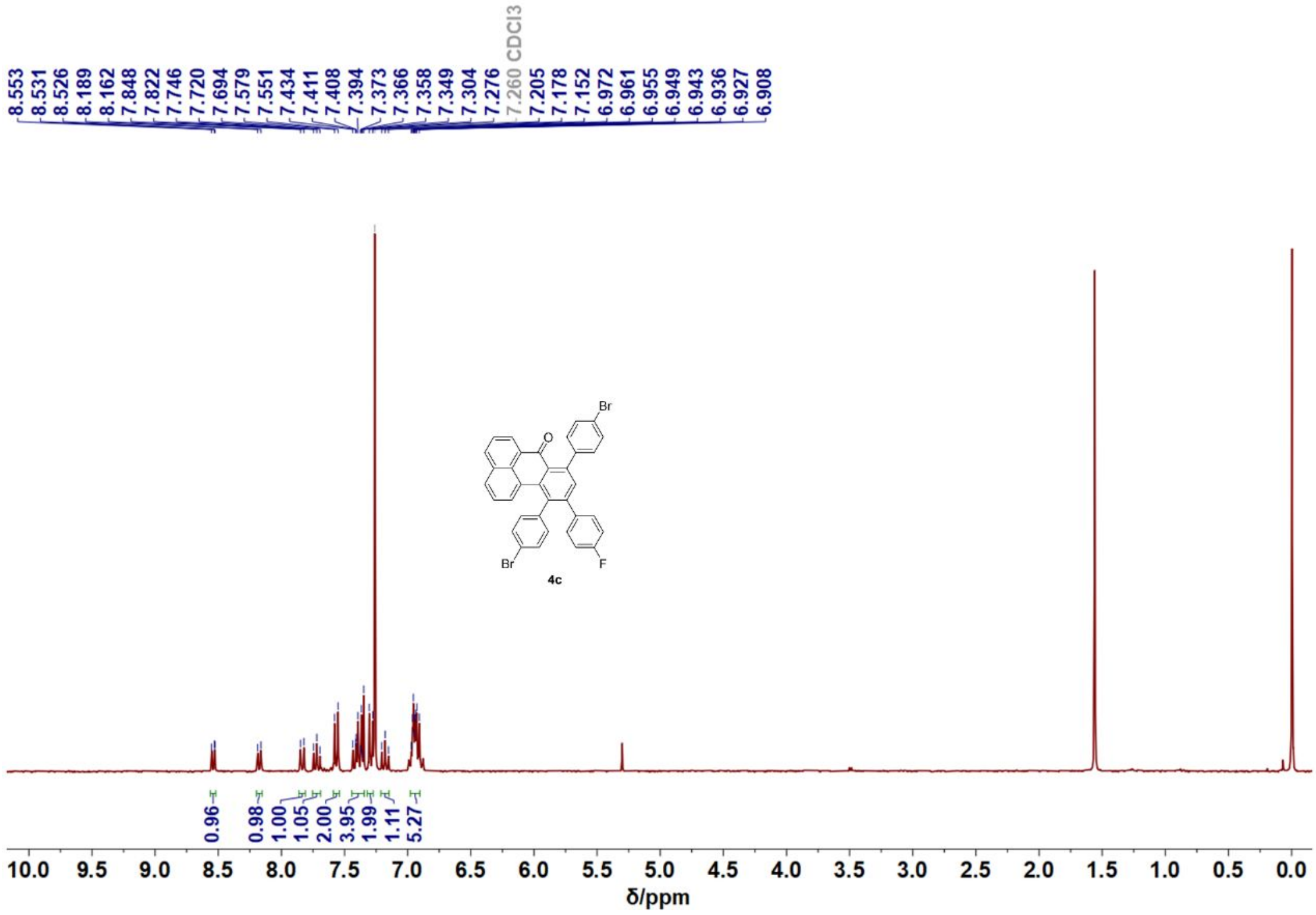

Figure S40. ^{1}H NMR spectrum of **4c** in $CDCl_3$ (300 MHz, 297 K).

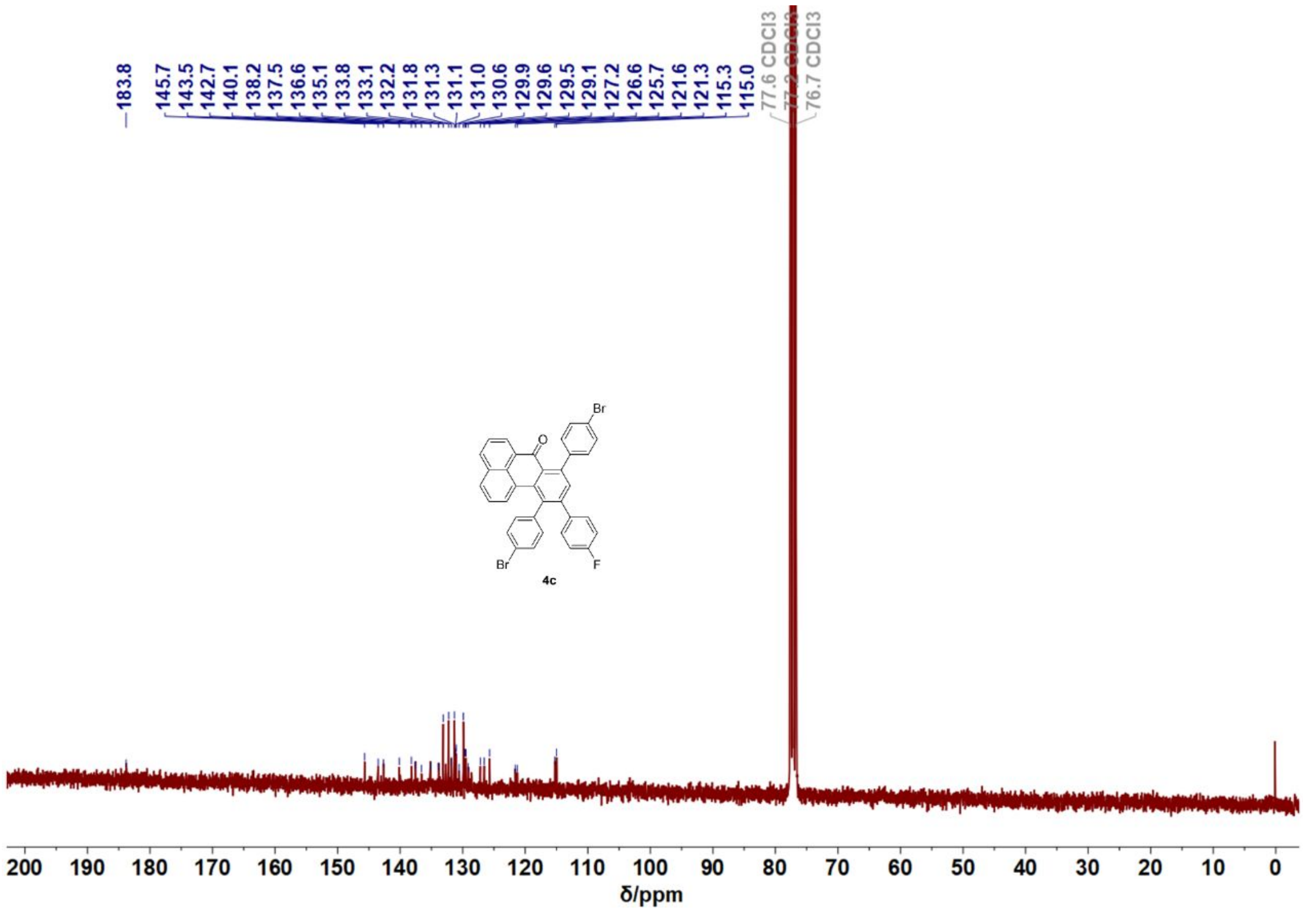

Figure S41. ^{13}C NMR spectrum of **4c** in $CDCl_3$ (75 MHz, 297 K).

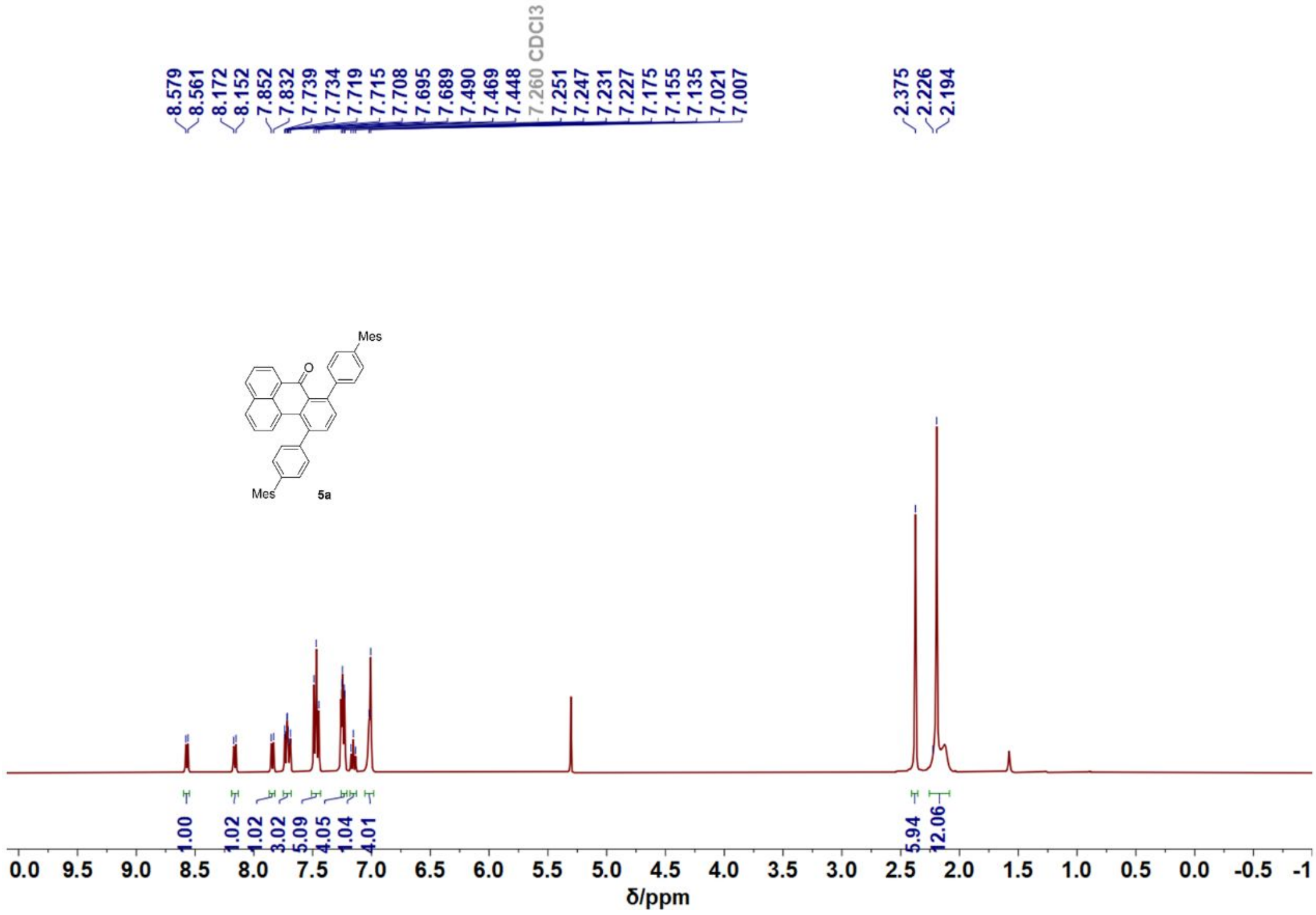

Figure S42. ^{1}H NMR spectrum of **5a** in $CDCl_3$ (400 MHz, 297 K).

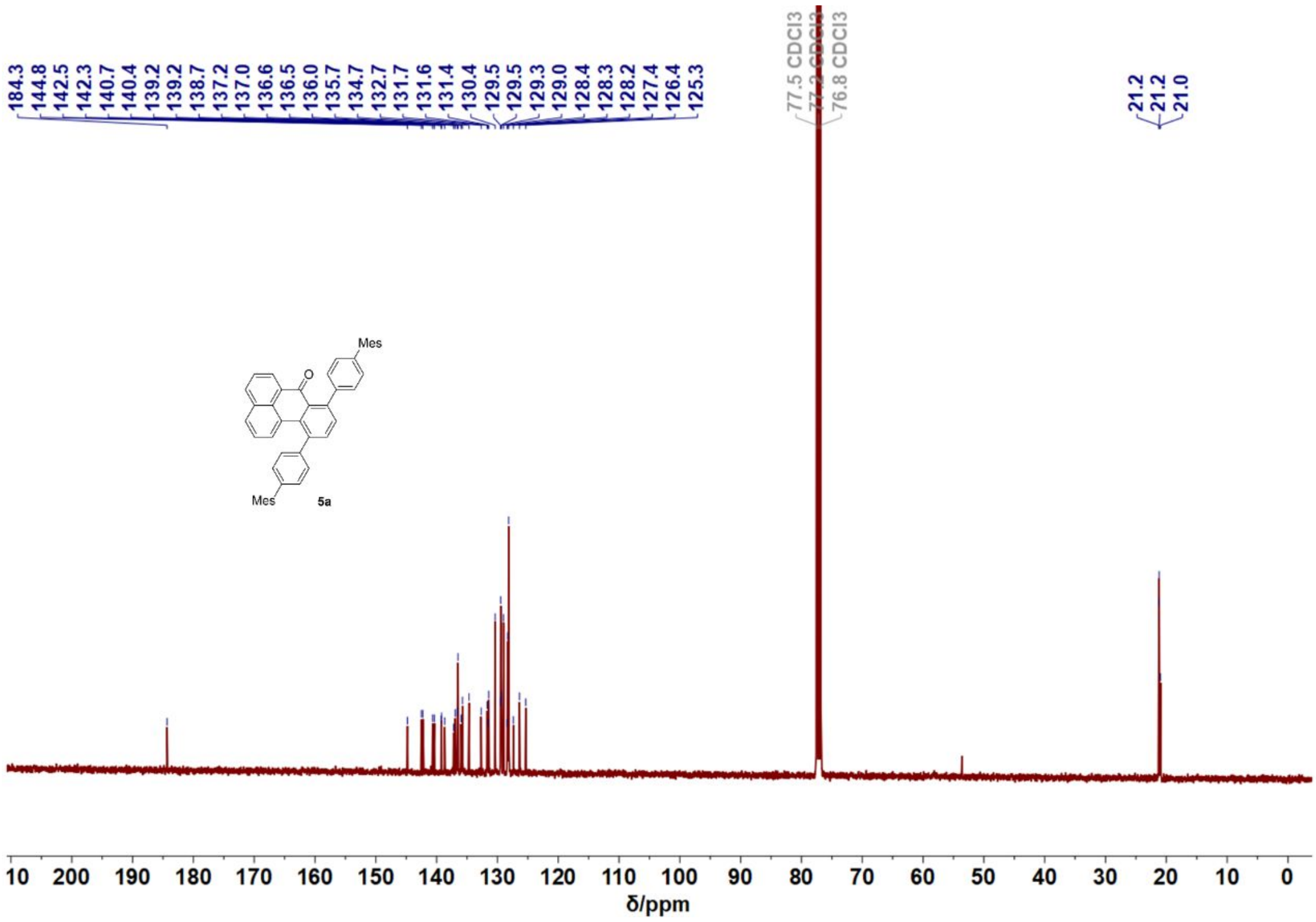

Figure S43. ^{13}C NMR spectrum of **5a** in $CDCl_3$ (101 MHz, 297 K).

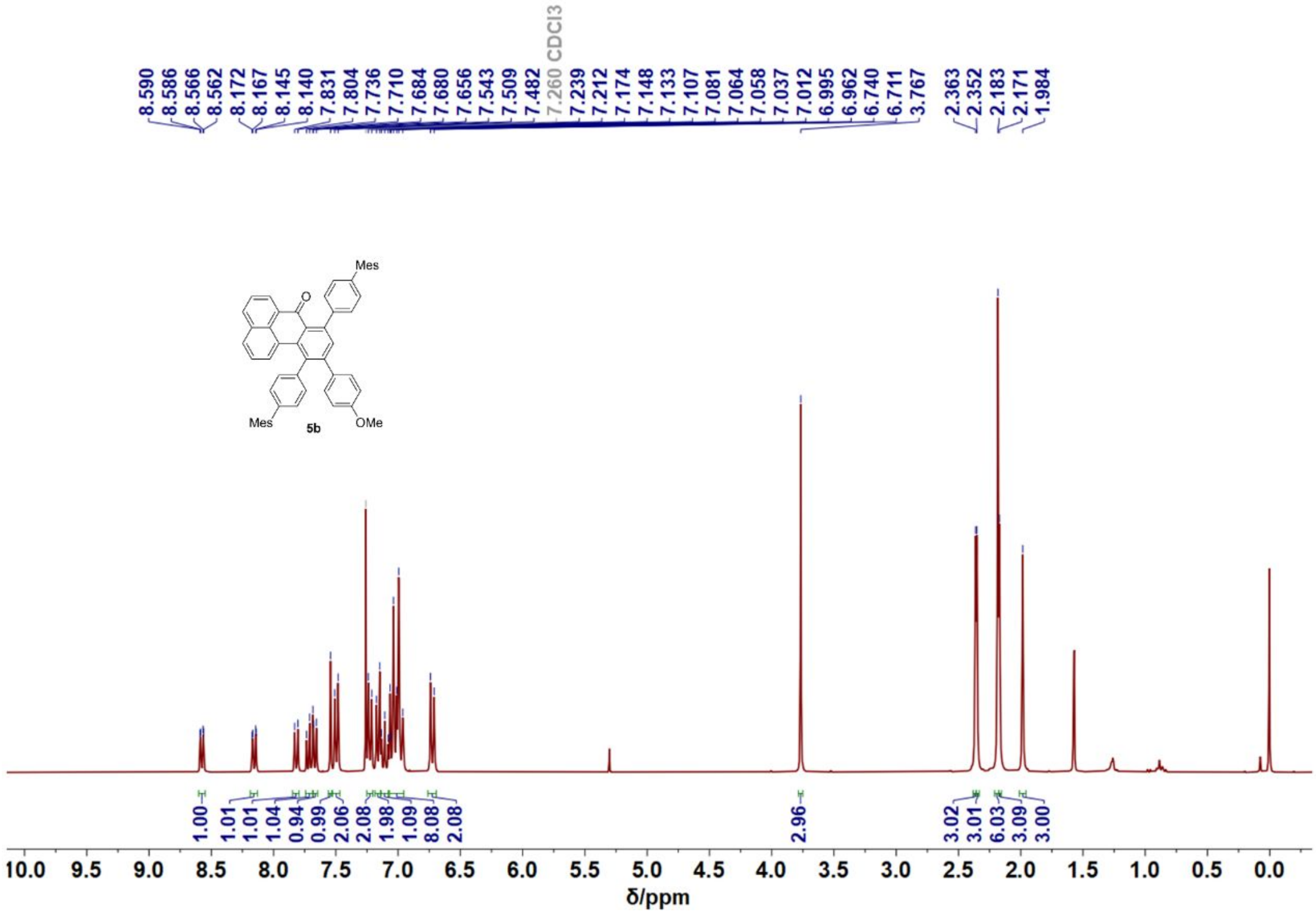

Figure S44. ^{1}H NMR spectrum of **5b** in $CDCl_3$ (300 MHz, 297 K).

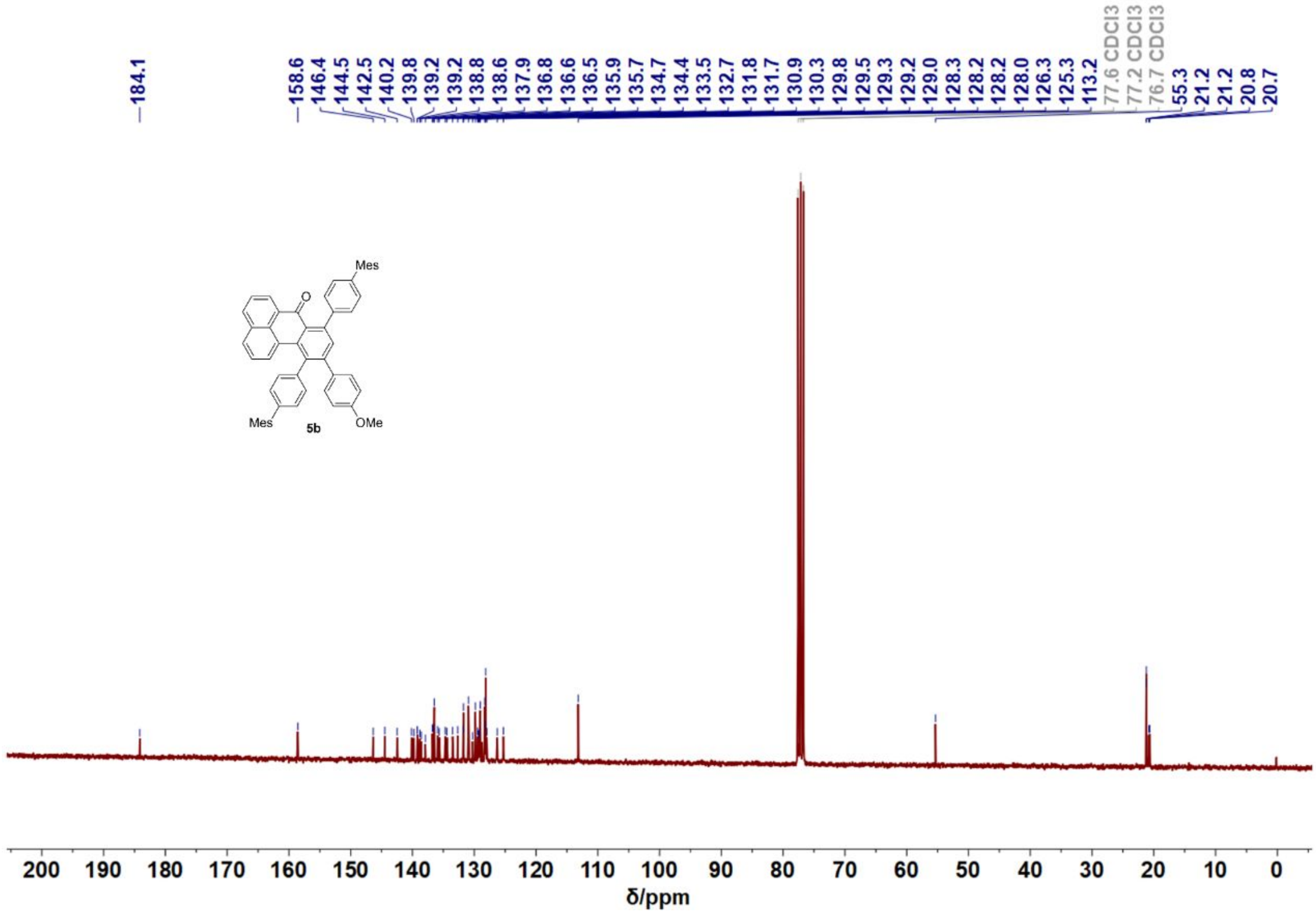

Figure S45. ^{13}C NMR spectrum of **5b** in $CDCl_3$ (75 MHz, 297 K).

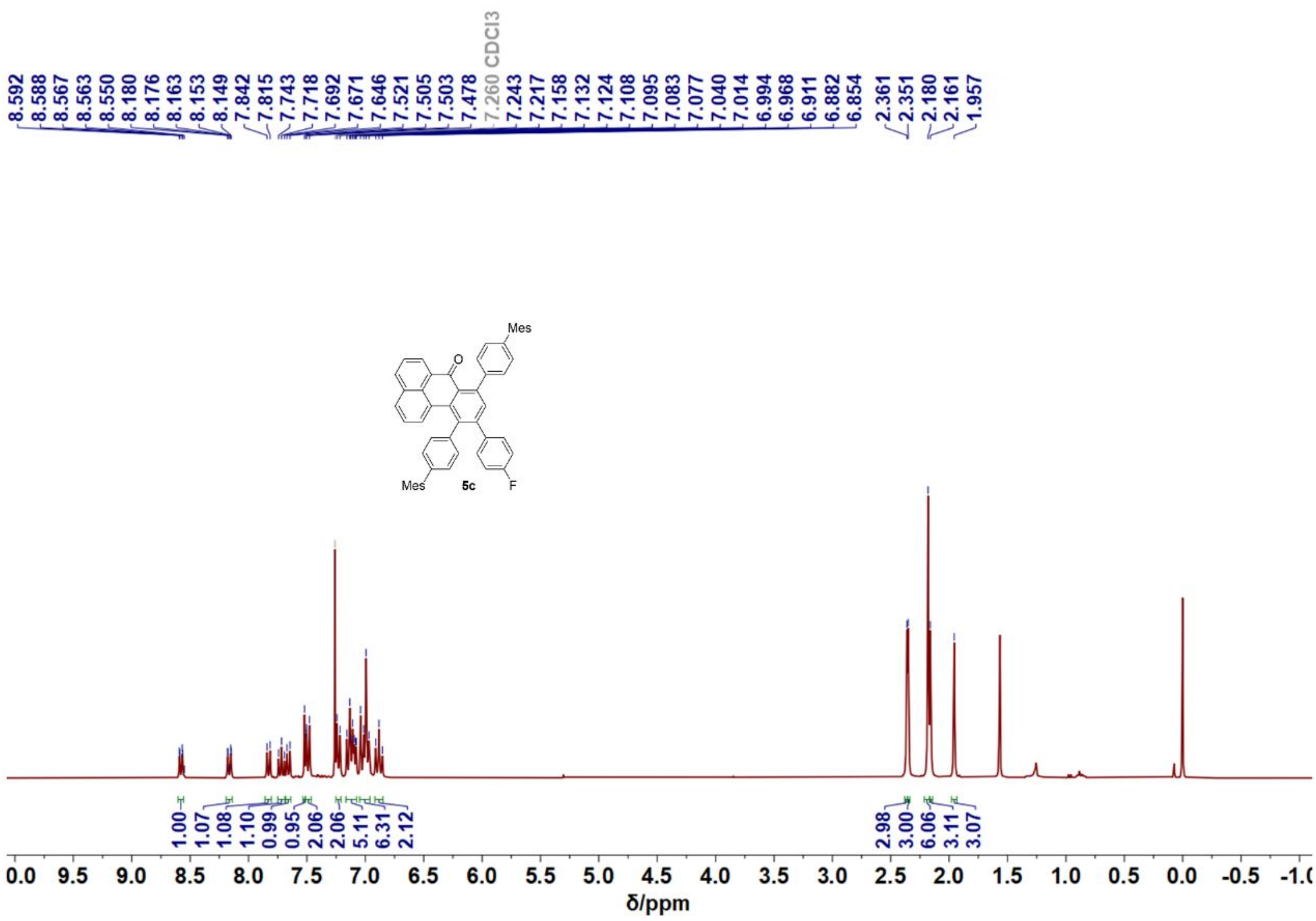

Figure S46. ^{1}H NMR spectrum of **5c** in $CDCl_3$ (300 MHz, 297 K).

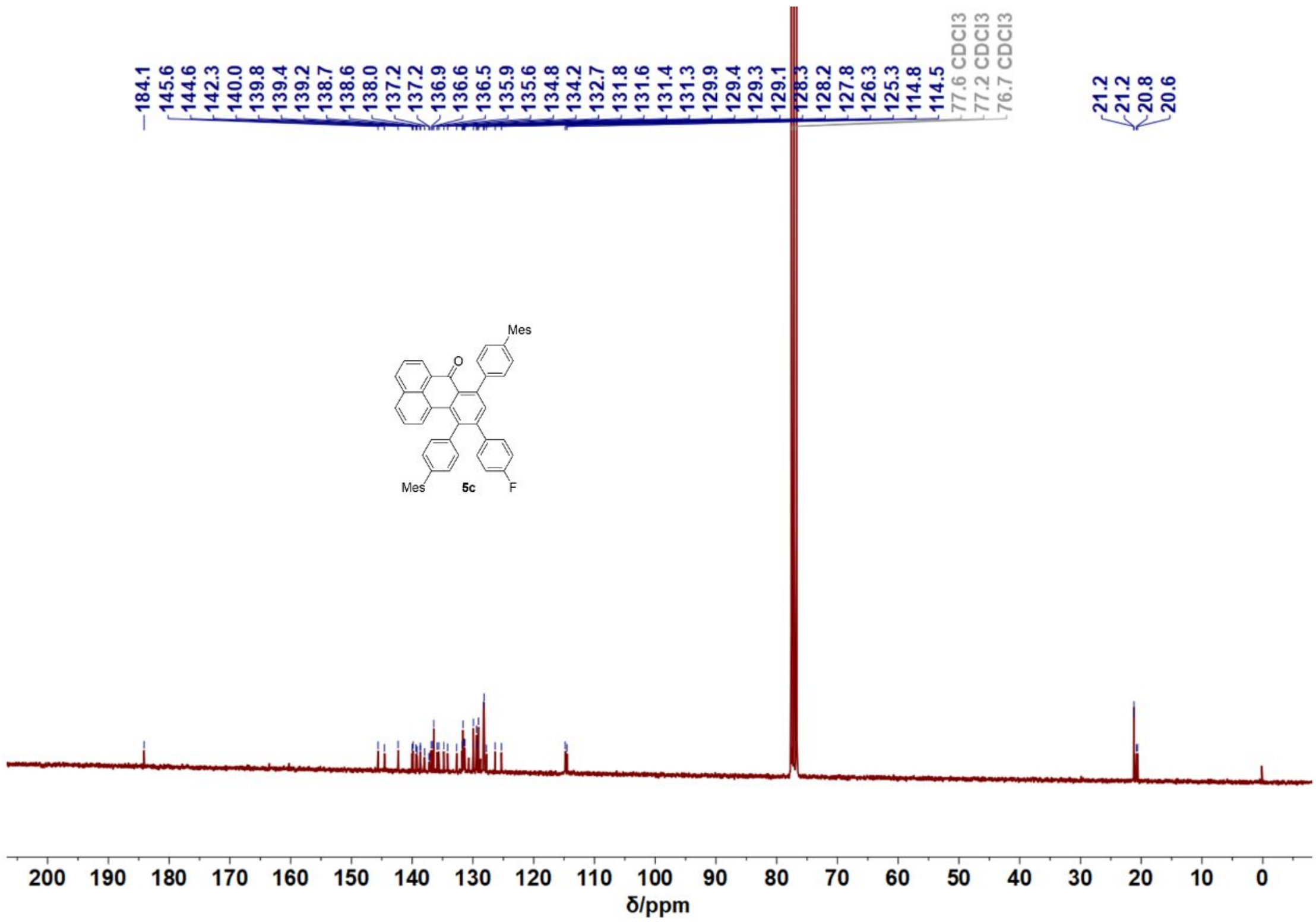

Figure S47. ^{13}C NMR spectrum of **5c** in $CDCl_3$ (75 MHz, 297 K).

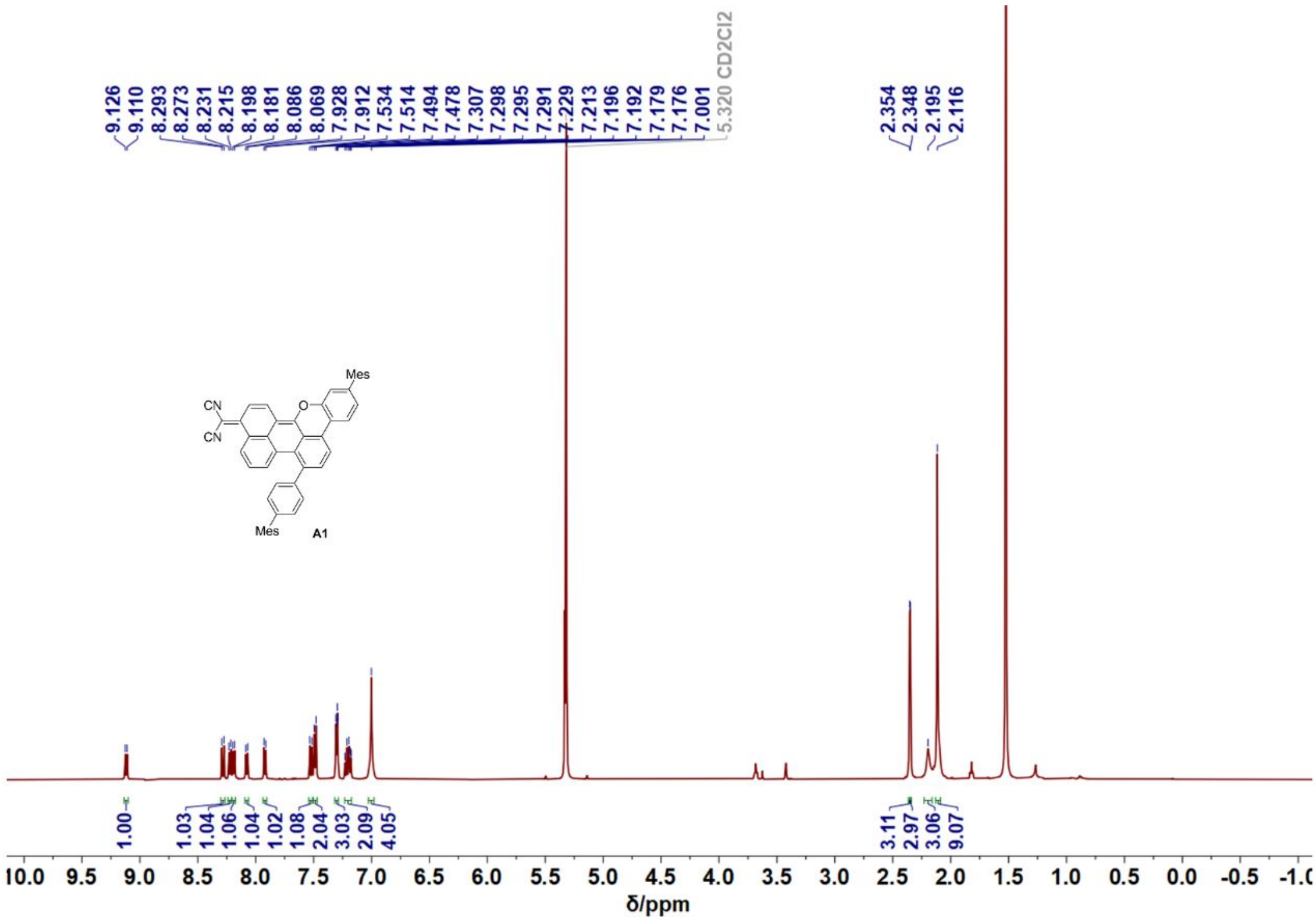

Figure S48. ^1H NMR spectrum of **A1** in CD_2Cl_2 (500 MHz, 297 K).

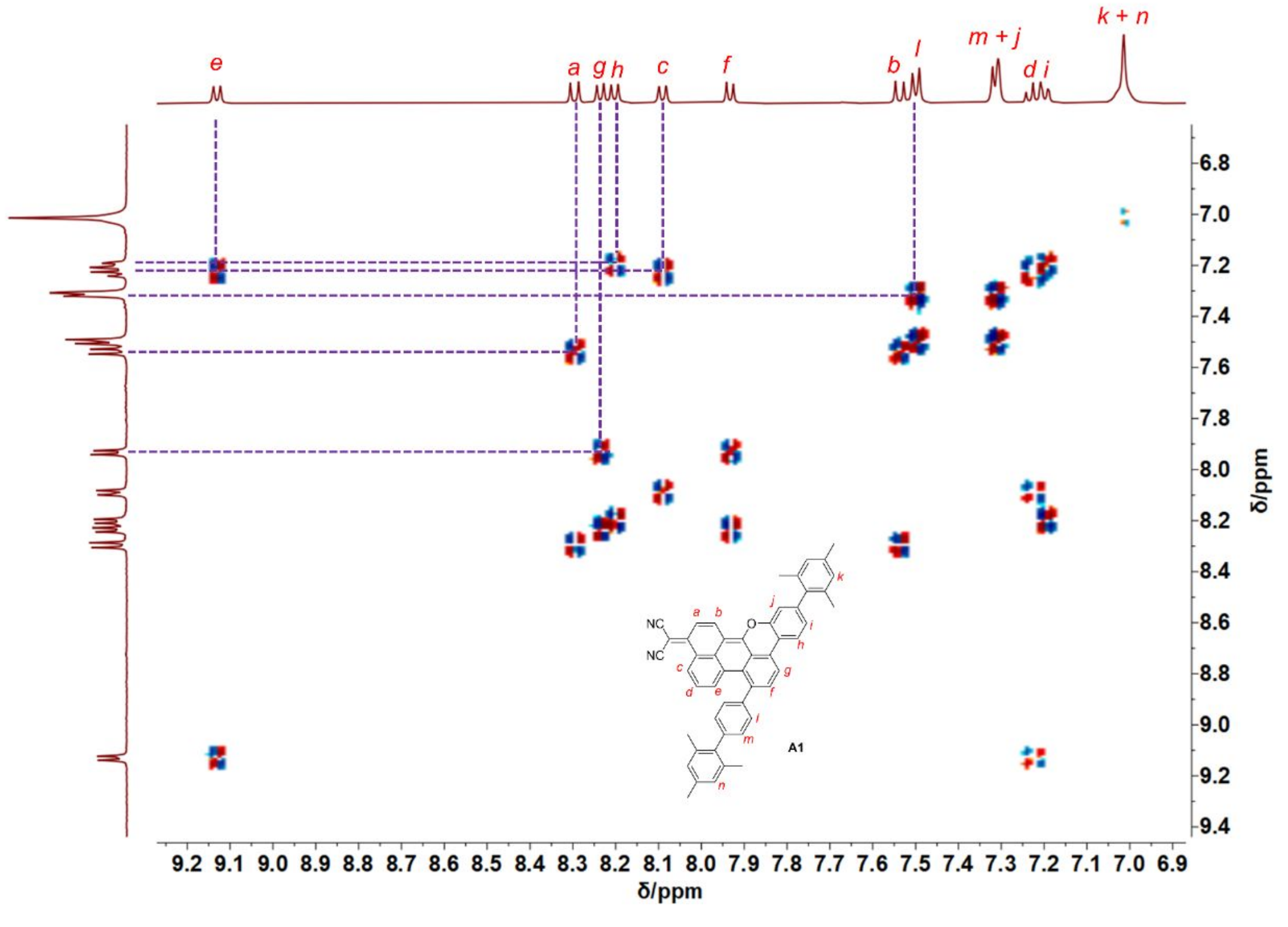

Figure S49. 2D ^1H–^1H COSY spectrum of **A1** in CD_2Cl_2 (500 MHz, 297 K).

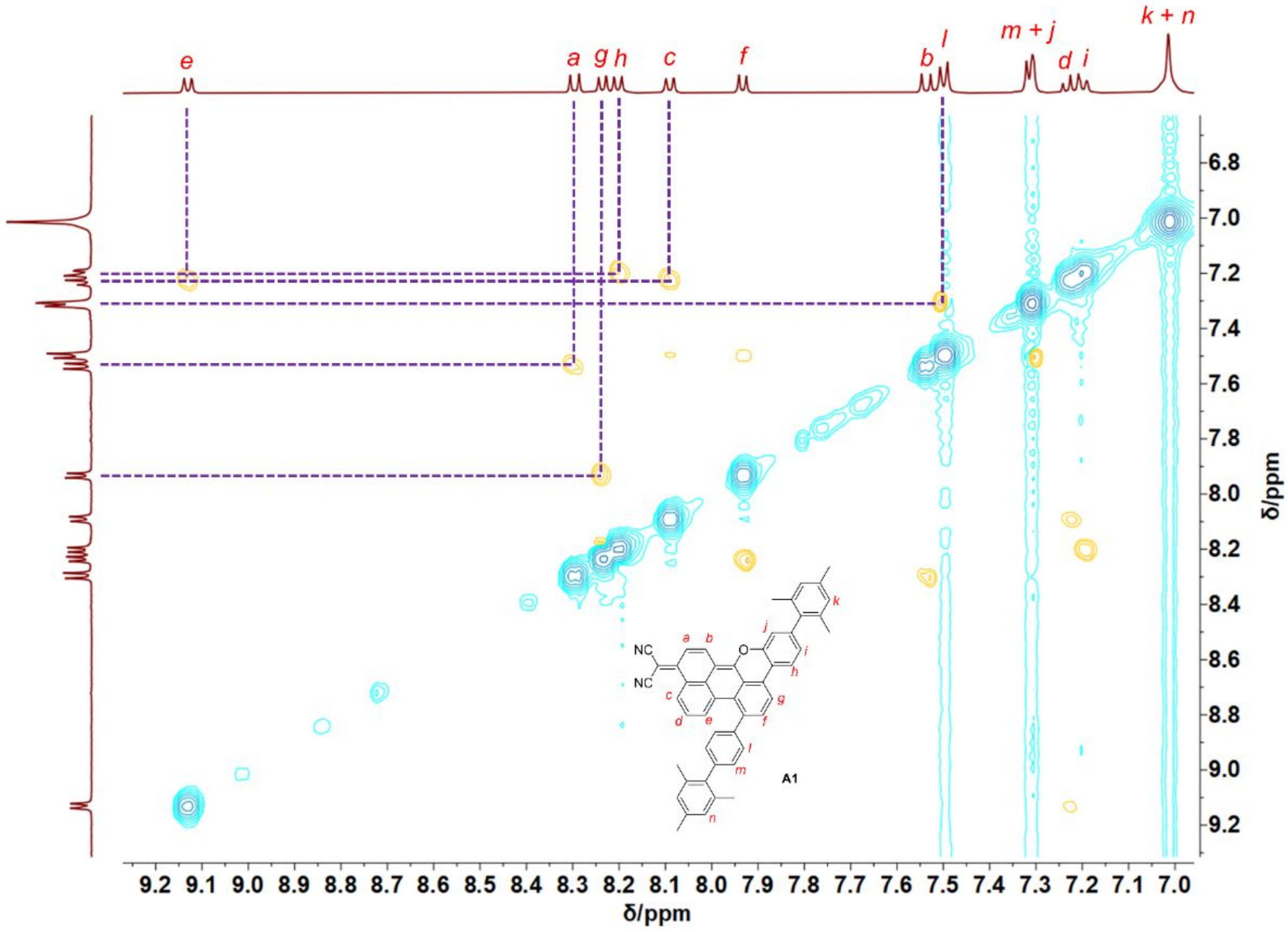

Figure S50. 2D ^1H–^1H ROESY spectrum (aromatic region) of **A1** in CD_2Cl_2 (500 MHz, 297 K).

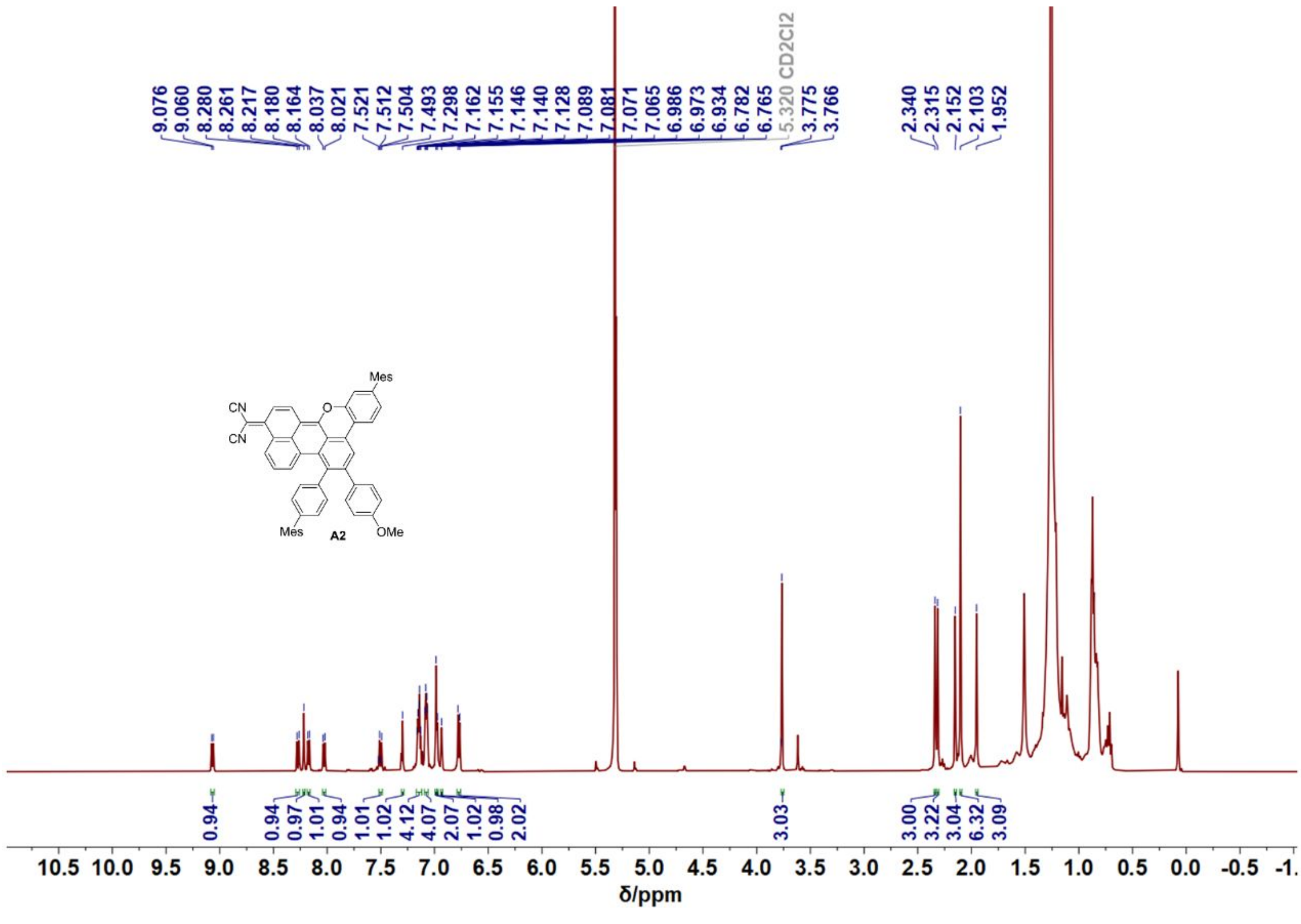

Figure S51. ^1H NMR spectrum of **A2** in CD_2Cl_2 (500 MHz, 297 K).

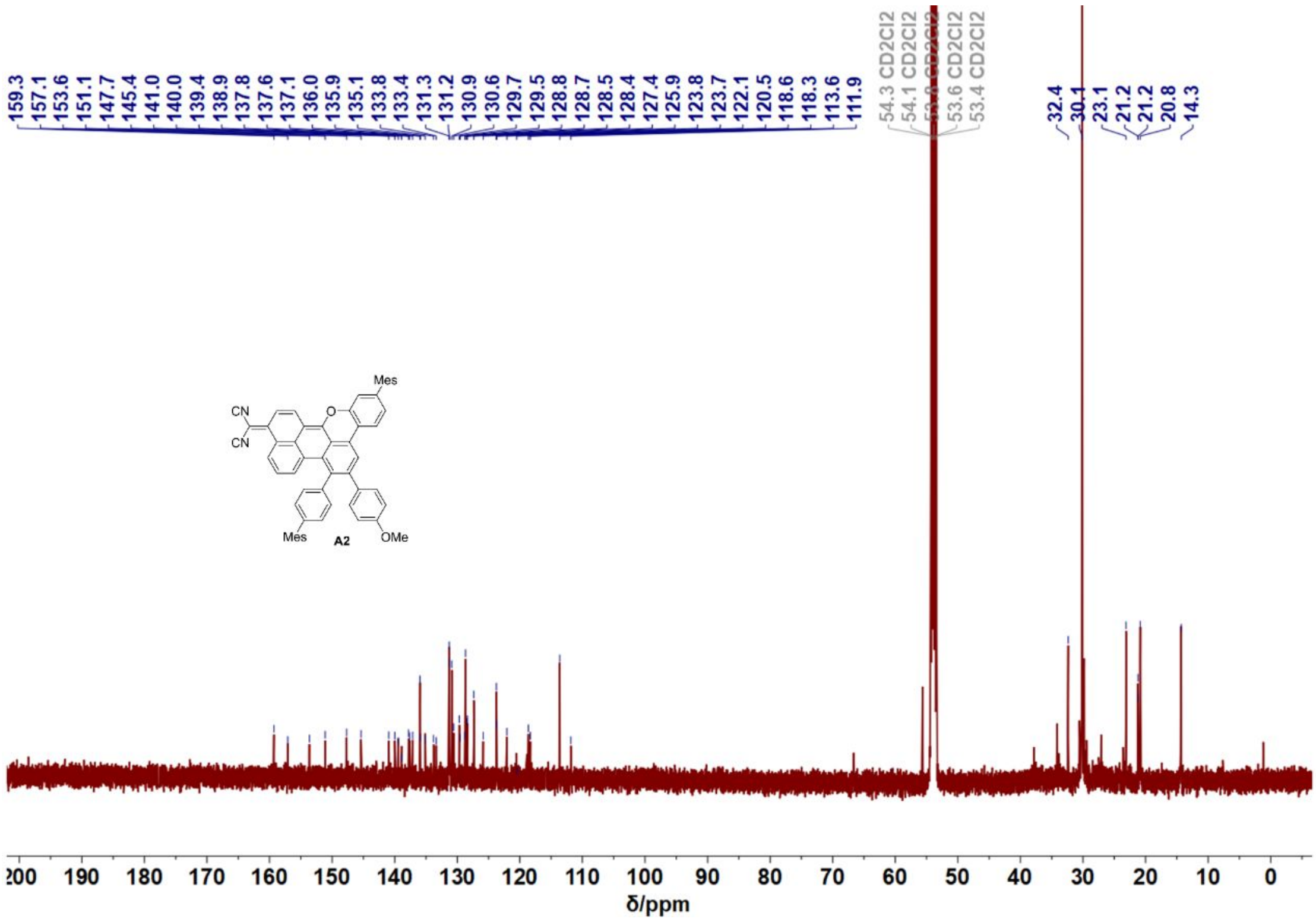

Figure S52. ^{13}C NMR spectrum of **A2** in CD_2Cl_2 (126 MHz, 297 K).

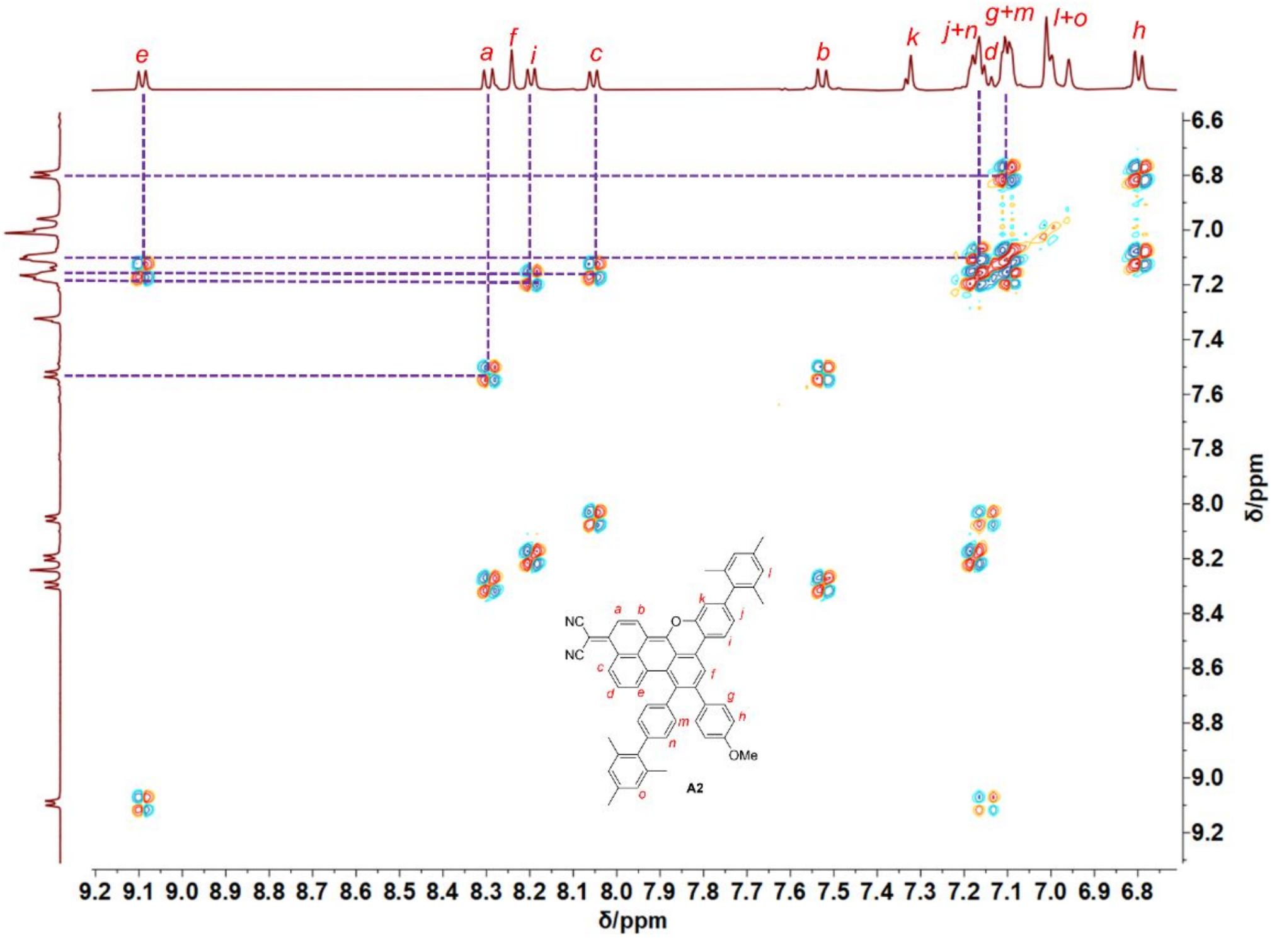

Figure S53. 2D ^{1}H–^{1}H COSY spectrum of **A2** in CD_2Cl_2 (500 MHz, 297 K).

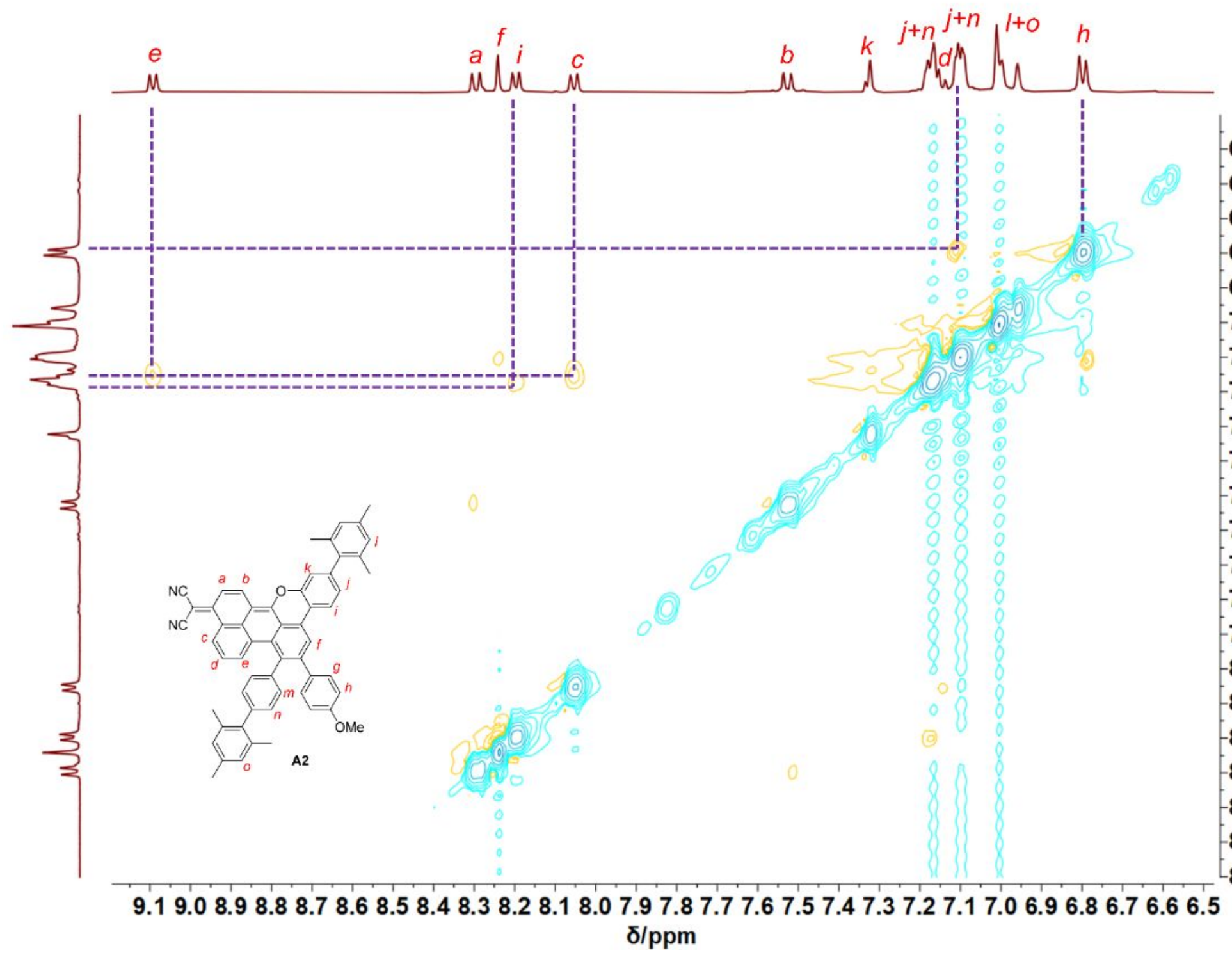

Figure S54. 2D ^1H–^1H ROESY spectrum (aromatic region) of **A2** in CD_2Cl_2 (500 MHz, 297 K).

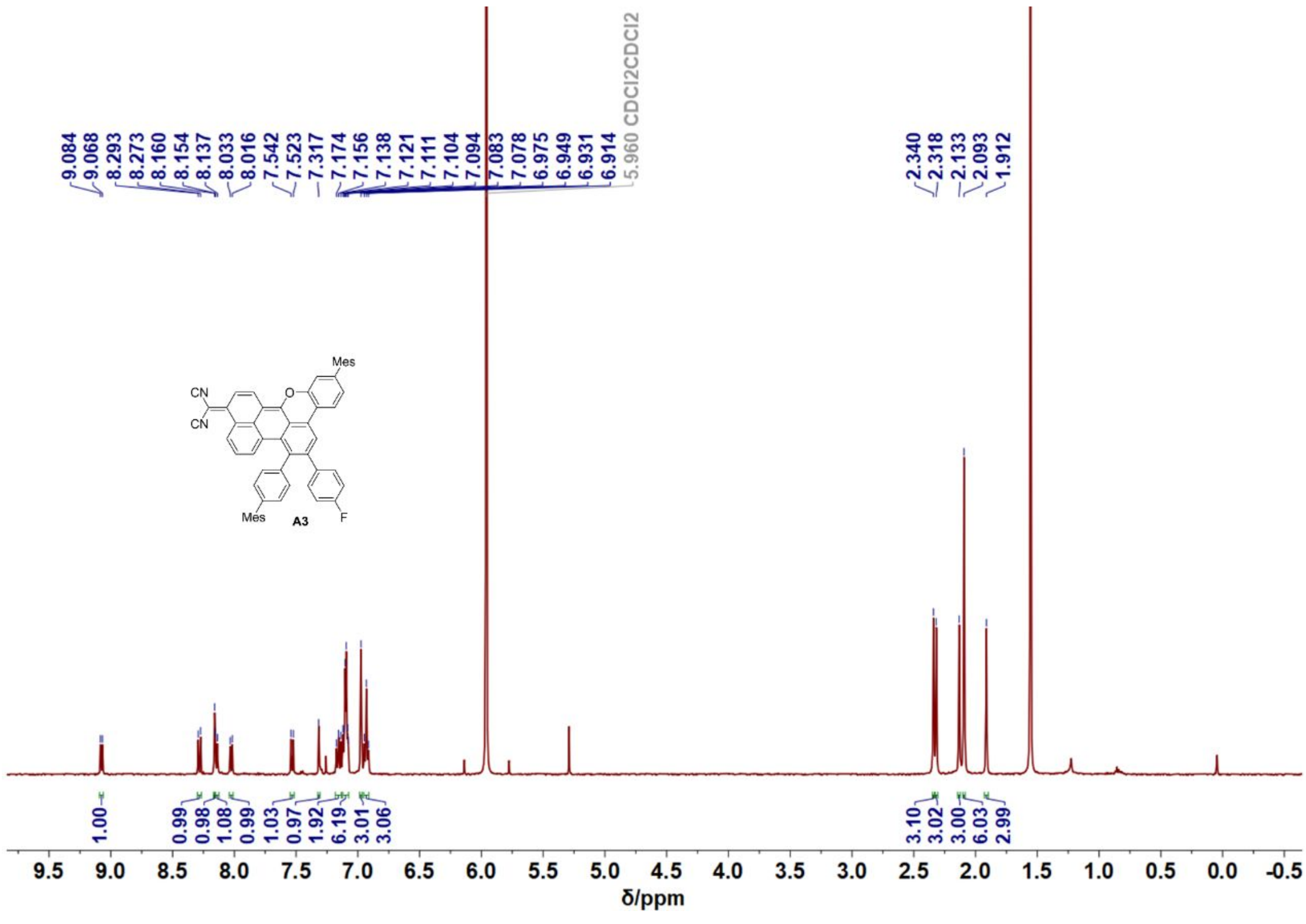

Figure S55. ^1H NMR spectrum of **A3** in $C_2D_2Cl_4$ (500 MHz, 297 K).

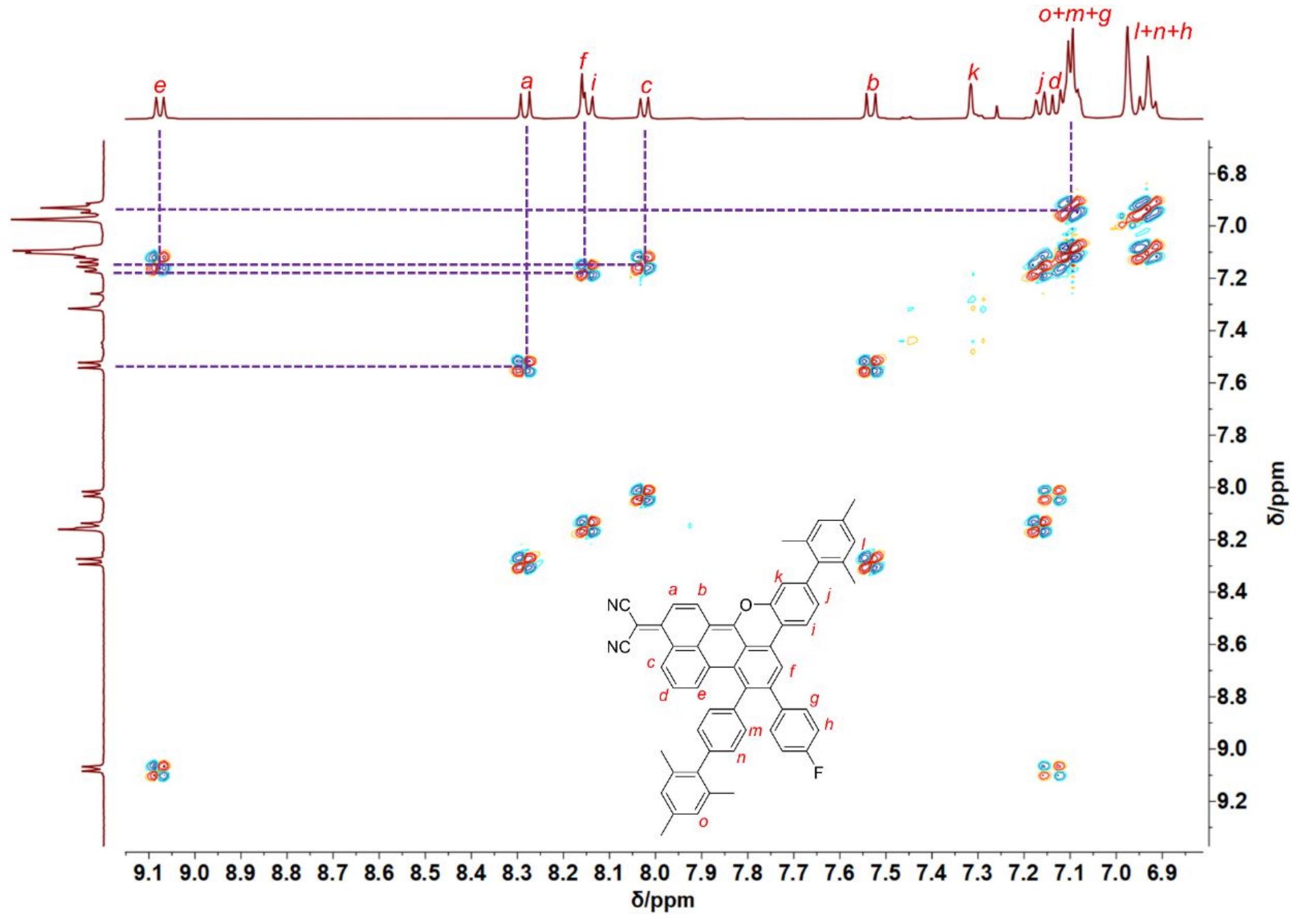

Figure S56. 2D ^1H–^1H COSY spectrum of **A3** in $C_2D_2Cl_4$ (500 MHz, 297 K).

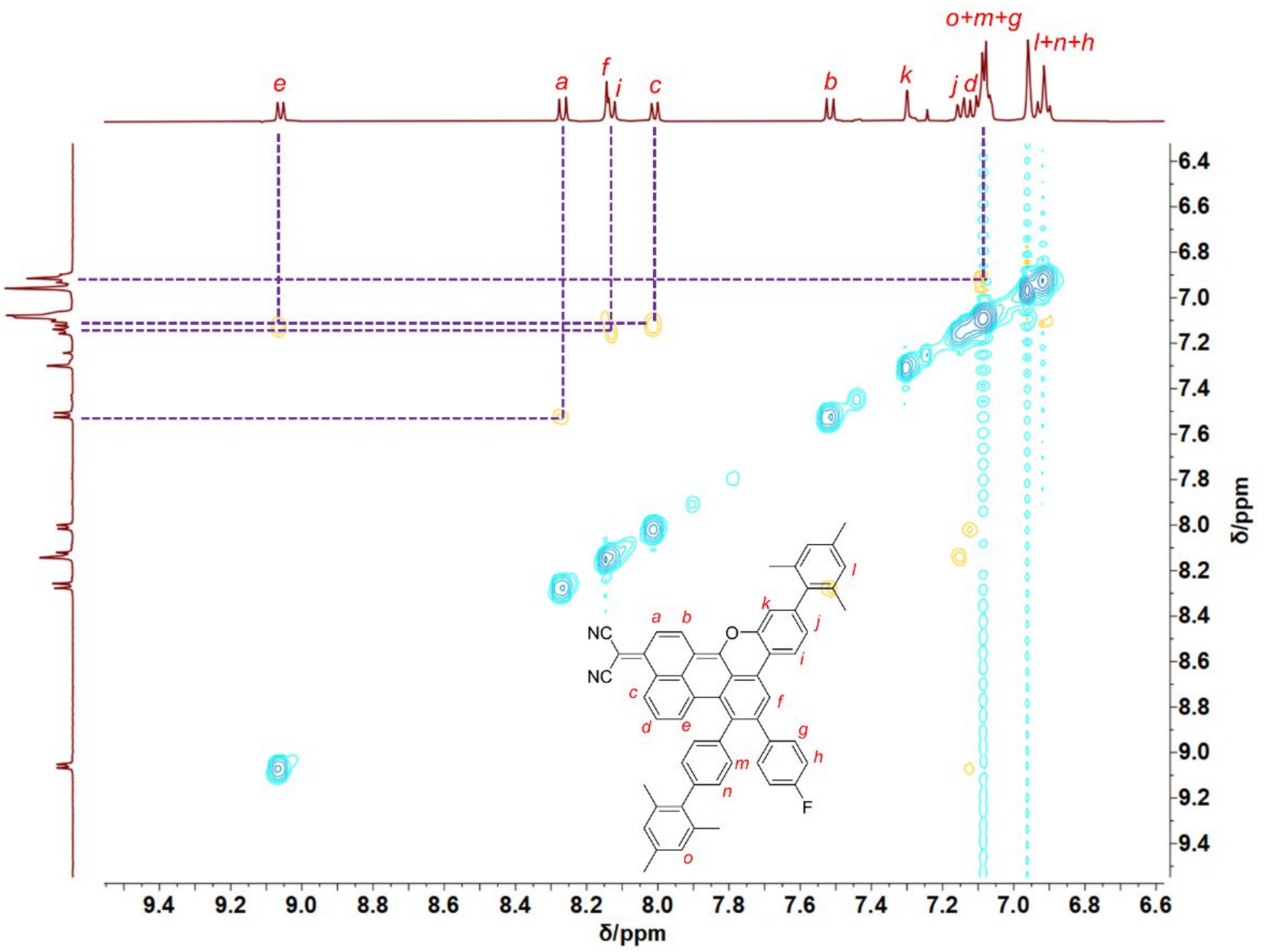

Figure S57. 2D ^1H–^1H ROESY spectrum (aromatic region) of **A3** in $C_2D_2Cl_4$ (500 MHz, 297 K).

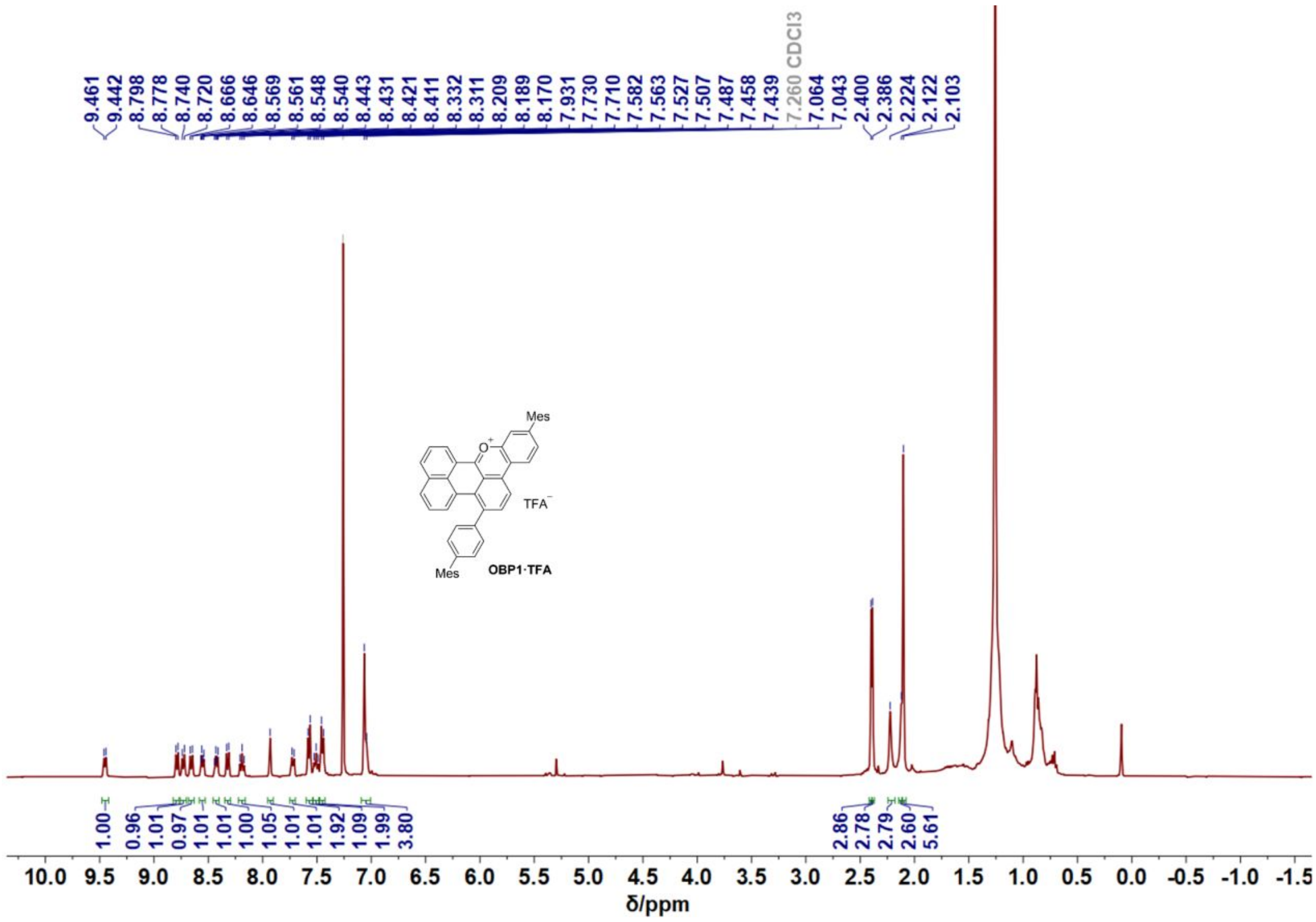

Figure S58. ^{1}H NMR spectrum of **OBP1·TFA** recorded in $CDCl_3$ containing 10% TFA-*d* at 297 K.

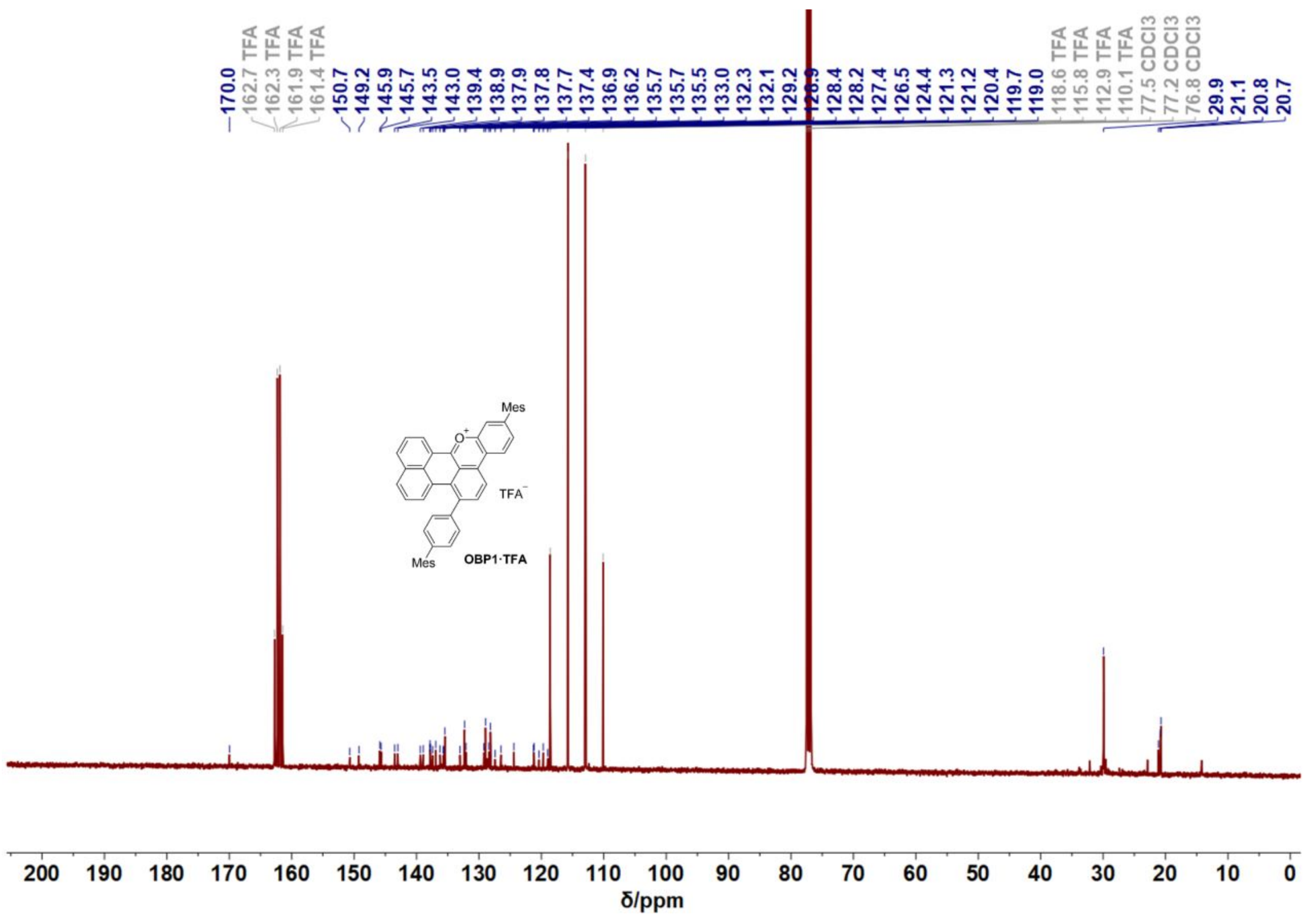

Figure S59. ^{13}C NMR spectrum of **OBP1·TFA** recorded in $CDCl_3$ containing 10% TFA-*d* at 297 K.

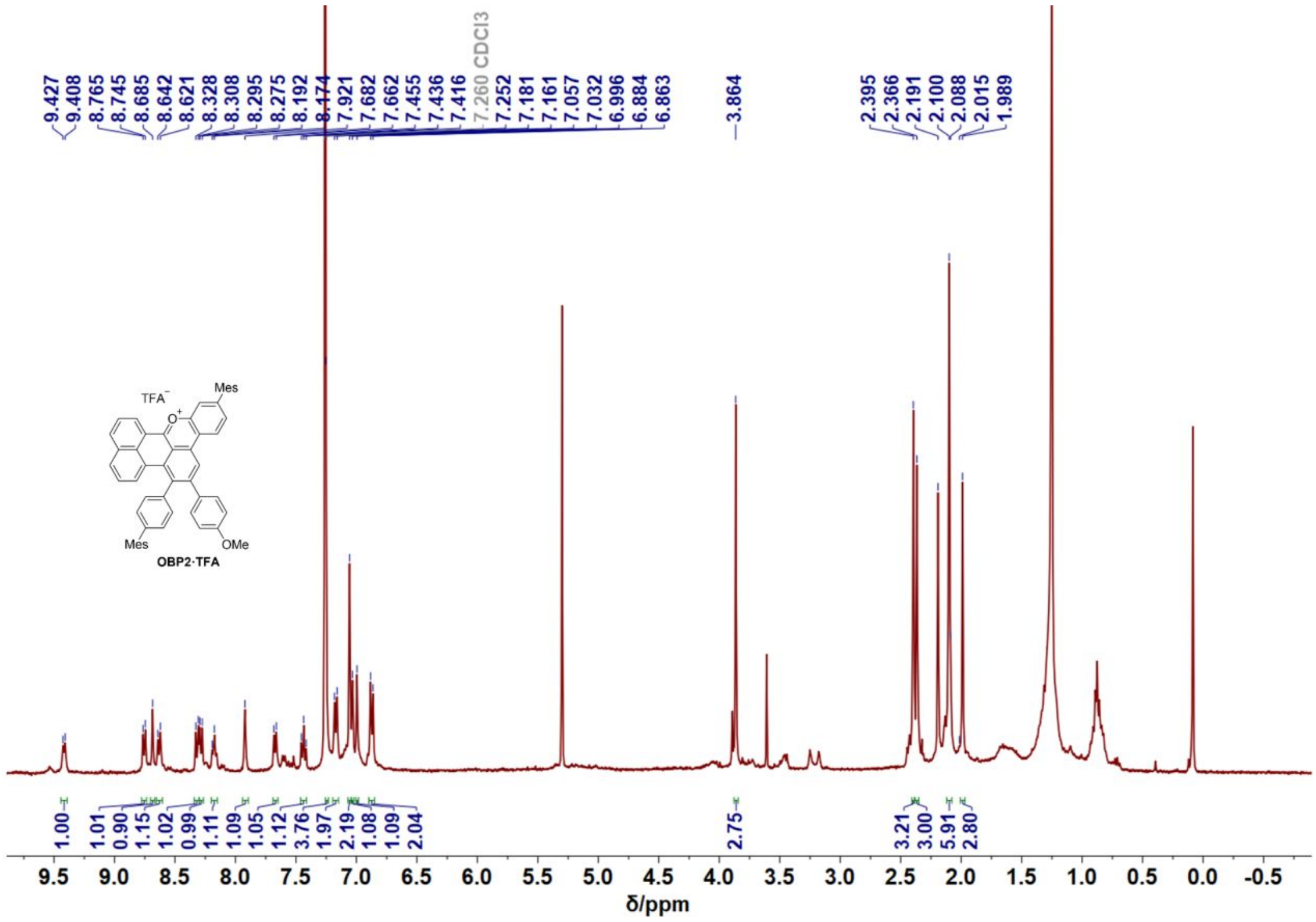

Figure S60. ^{1}H NMR spectrum of **OBP2·TFA** recorded in $CDCl_3$ containing 10% TFA-*d* at 297 K.

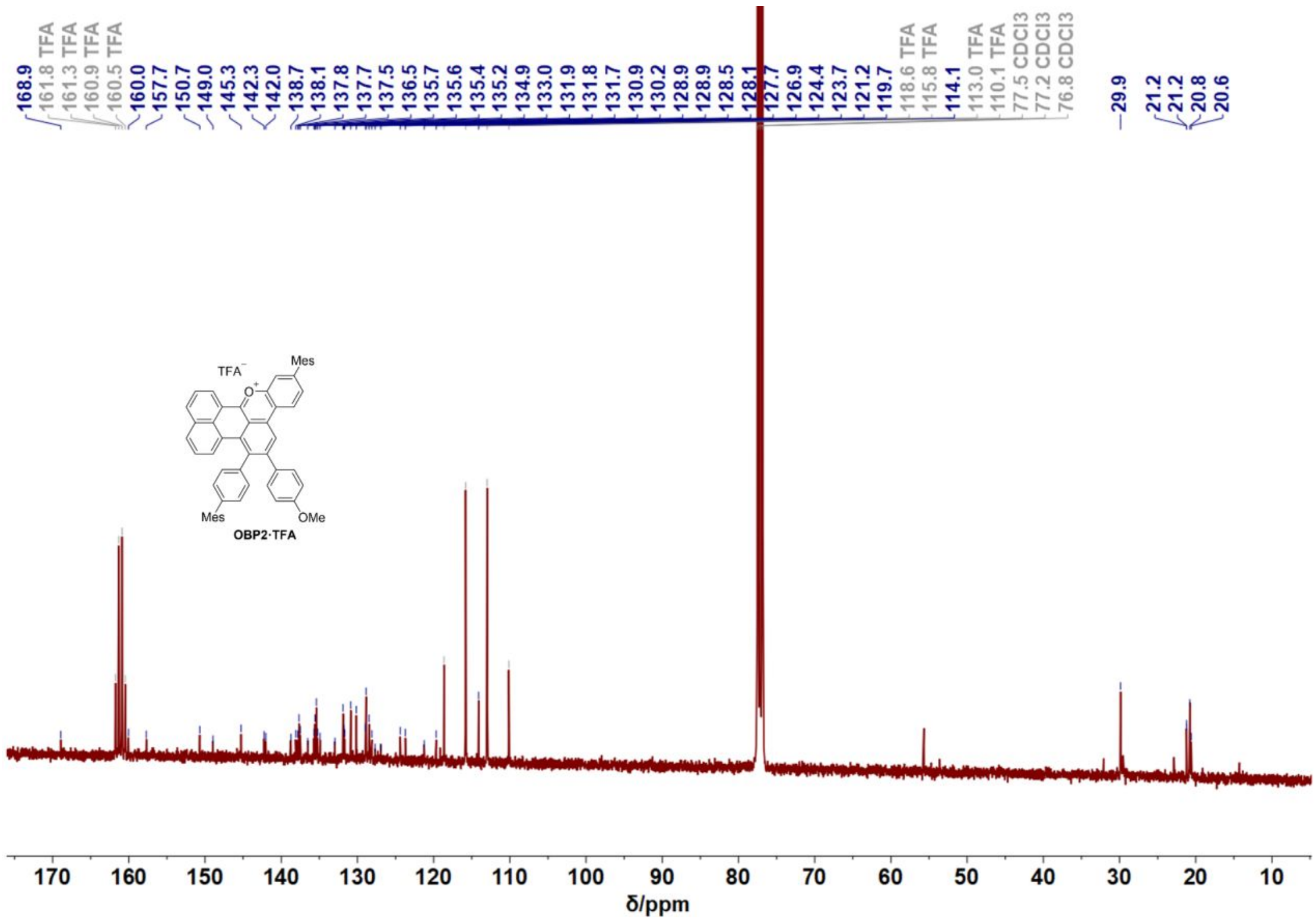

Figure S61. ^{13}C NMR spectrum of **OBP2·TFA** recorded in $CDCl_3$ containing 10% TFA-*d* at 297 K.

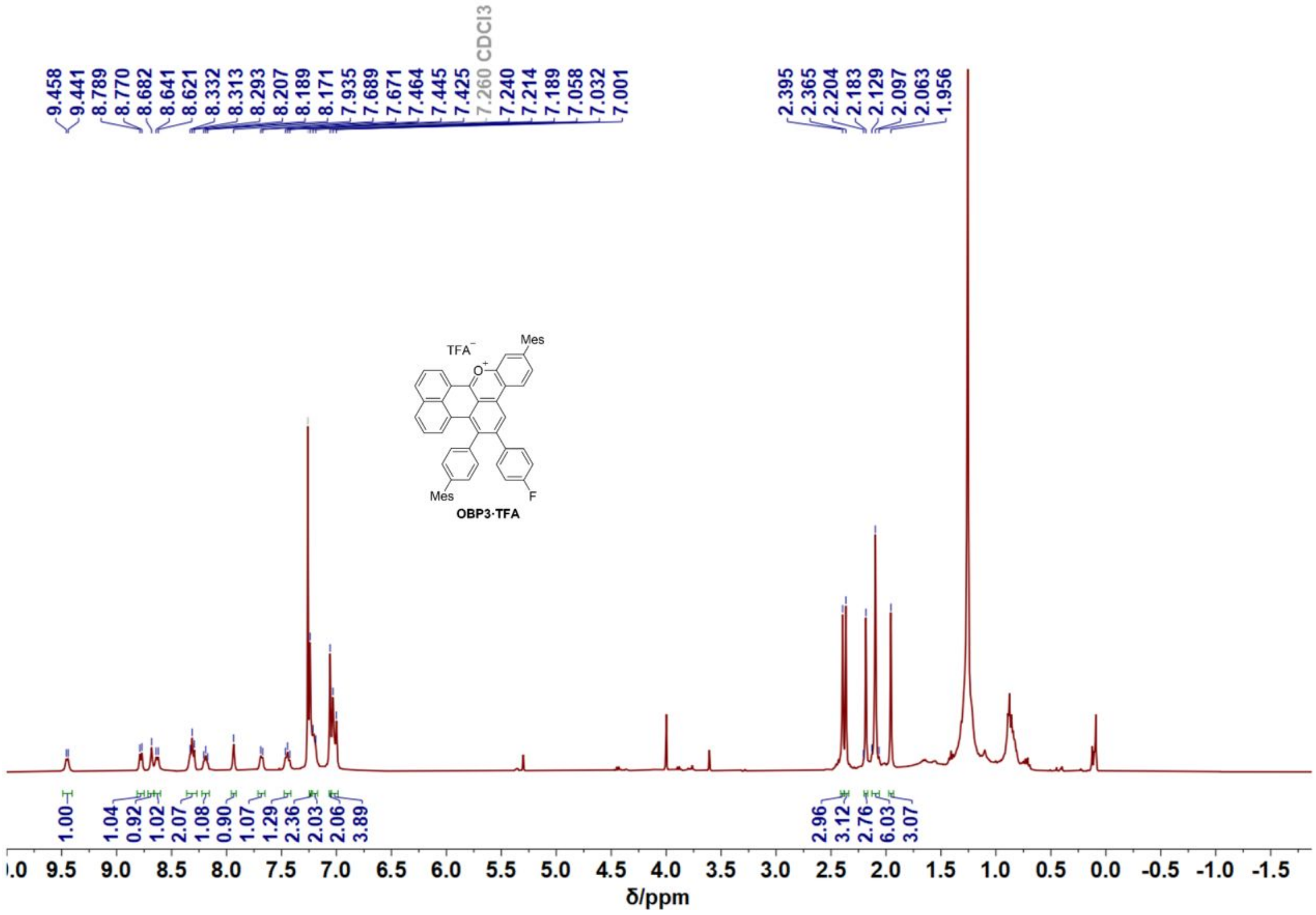

Figure S62. ^{1}H NMR spectrum of **OBP3·TFA** recorded in $CDCl_3$ containing 10% TFA-*d* at 297 K.

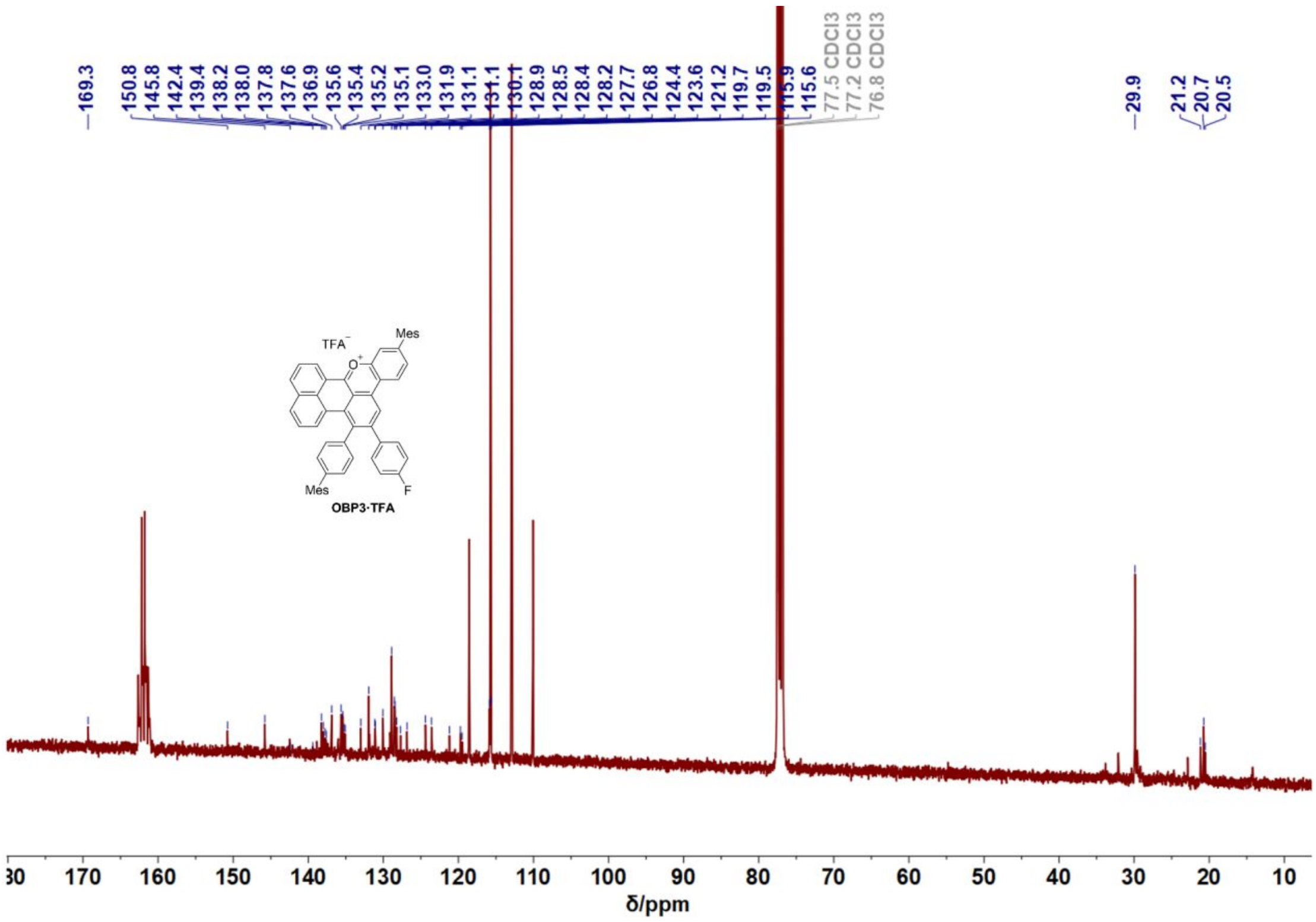

Figure S63. ^{13}C NMR spectrum of **OBP3·TFA** recorded in $CDCl_3$ containing 10% TFA-*d* at 297 K.

7. References

(1) Gottlieb, H. E.; Kotlyar, V.; Nudelman, A. NMR Chemical Shifts of Common Laboratory Solvents as Trace Impurities. *J. Org. Chem.* **1997**, *62* (21), 7512–7515.

(2) Wössner, J. S.; Esser, B. Spiroconjugated Donor−*σ*–Acceptor Charge-Transfer Dyes: Effect of the π-Subsystems on the Optoelectronic Properties. *J. Org. Chem.* **2020**, *85* (7), 5048–5057.

(3) Romer, D. R. Convenient Laboratory Method for the Synthesis of Symmetrical 1,3-Diphenylacetone Derivatives. *Synthesis* **2011**, *2011* (17), 2721–2723.

(4) Wendlandt, A.E.; Stahl, S.S. Quinone-Catalyzed Selective Oxidation of Organic Molecules. *Angew. Chem. Int. Ed*. **2015**, *54*, 14638–14658.

(5) Cha, D.; Min, S. 2,3-Dichloro-5,6-Dicyano-1,4-Benzoquinone (DDQ)-Mediated C–C Bond Formation: Redox Strategies from Stoichiometric to Catalytic Systems. *ChemistryOpen*, **2026**, *15*, e202500568.

(6) Bruker (2024). APEX6. Bruker AXS LLC, Madison, Wisconsin, USA.

(7) Bruker (2024) SAINT V8.41. Bruker AXS LLC, Madison, Wisconsin, USA.

(8) Bruker (2024), TWINABS V 2012/1.

(9) Sheldrick, G. M. SHELXT - Integrated space-group and crystal-structure determination. *Acta Cryst*. **2015**, *A71*, 3–8.

(10) Sheldrick, G. M. Crystal structure refinement with SHELXL. *Acta Cryst*. **2015**. *C71*, 3–8.

(11) Dolomanov, O.V.; Bourhis, L.J.; Gildea, R.J.; Howard, J.A.K.; Puschmann, H., OLEX2: A complete structure solution, refinement and analysis program. *J. Appl. Cryst.* **2009**, *42*, 339–341.

(12) Gaussian 16, Revision A.03, Frisch, M. J.; Trucks, G. W.; Schlegel, H. B.; Scuseria, G. E.; Robb, M. A.; Cheeseman, J. R.; Scalmani, G.; Barone, V.; Petersson, G. A.; Nakatsuji, H.; Li, X.; Caricato, M.; Marenich, A. V.; Bloino, J.; Janesko, B. G.; Gomperts, R.; Mennucci, B.; Hratchian, H. P.; Ortiz, J. V.; Izmaylov, A. F.; Sonnenberg, J. L.; Williams-Young, D.; Ding, F.; Lipparini, F.; Egidi, F.; Goings, J.; Peng, B.; Petrone, A.; Henderson, T.; Ranasinghe, D.; Zakrzewski, V. G.; Gao, J.; Rega, N.; Zheng, G.; Liang, W.; Hada, M.; Ehara, M.; Toyota, K.; Fukuda, R.; Hasegawa, J.;

Ishida, M.; Nakajima, T.; Honda, Y.; Kitao, O.; Nakai, H.; Vreven, T.; Throssell, K.; Montgomery, J. A., Jr.; Peralta, J. E.; Ogliaro, F.; Bearpark, M. J.; Heyd, J. J.; Brothers, E. N.; Kudin, K. N.; Staroverov, V. N.; Keith, T. A.; Kobayashi, R.; Normand, J.; Raghavachari, K.; Rendell, A. P.; Burant, J. C.; Iyengar, S. S.; Tomasi, J.; Cossi, M.; Millam, J. M.; Klene, M.; Adamo, C.; Cammi, R.; Ochterski, J. W.; Martin, R. L.; Morokuma, K.; Farkas, O.; Foresman, J. B.; Fox, D. J. Gaussian, Inc., Wallingford (USA, CT), **2016**.

(13) Adamo, C.; Barone, V. Toward reliable density functional methods without adjustable parameters: The PBE0 model. *J. Chem. Phys.* **1999**, 110 (13), 6158–6170.

(14) Yanai, T.; Tew, D. P.; Handy, N. C. A new hybrid exchange–correlation functional using the Coulomb-attenuating method (CAM-B3LYP). *Chem. Phys. Lett.* **2004**, *393* (1), 51–57.

(15) Schlegel, H. B. Optimization of equilibrium geometries and transition structures. *J. Comp. Chem.* **1982**, *3* (2), 214–218.

(16) Wolinski, K.; Hinton, J. F.; Pulay, P. Efficient implementation of the gauge-independent atomic orbital method for NMR chemical shift calculations. *J. Am. Chem. Soc.* **1990**, *112* (23), 8251–8260.

(17) Lu, T.; Chen, F. Multiwfn: A multifunctional wavefunction analyzer. *J. Comput. Chem.* **2012**, *33* (5), 580–592.

8. Cartesian coordinates

Structure optimizations of cations OBP1$^+$–OBP3$^+$

OBP1$^+$

E(PBE0/def2-SVP) = –1885.06250575

Thermal correction to Gibbs Free Energy = 0.623059

Sum of electronic and thermal Free Energies = –1884.439514

C	-1.84053	3.71252	0.61539
C	-1.2815	4.98273	0.75917
C	0.06526	5.17229	0.50031
C	0.89938	4.09868	0.11022
C	0.35383	2.78412	0.01819
C	-1.04143	2.62328	0.24926
C	2.2593	4.3141	-0.21662
C	3.03122	3.26162	-0.65616
C	2.49817	1.96558	-0.7171
C	1.18229	1.6818	-0.33749

C	-1.60432	1.32946	0.03784
C	0.61022	0.32979	-0.34817
O	-2.91119	1.24755	0.14769
C	-0.81618	0.1954	-0.2588
C	1.36133	-0.86108	-0.44583
C	-1.47437	-1.06231	-0.40843
C	-2.91984	-1.10512	-0.30208
C	2.83785	-0.93278	-0.30423
C	-3.70488	-2.26378	-0.4517
C	-5.08191	-2.205	-0.33362
C	-5.74436	-0.99081	-0.05782
C	-4.98523	0.16448	0.09986
C	-3.60123	0.08662	-0.02368
C	3.44542	-0.56285	0.90329
C	4.82749	-0.6206	1.04286
C	5.64121	-1.0473	-0.01622
C	5.02671	-1.44732	-1.20865
C	3.64101	-1.39954	-1.35039
C	-0.69185	-2.19285	-0.63535
C	0.68875	-2.0814	-0.62299
H	-2.90809	3.56192	0.78365
H	0.50626	6.16879	0.58502
H	2.66983	5.32369	-0.14113
H	4.06853	3.42261	-0.95666
H	3.14231	1.17054	-1.08382
H	-3.22617	-3.22068	-0.66346
H	-5.67289	-3.11525	-0.45641
H	-5.44969	1.12822	0.31702
H	2.82772	-0.21394	1.73463
H	5.2894	-0.32417	1.9882
H	5.64444	-1.79127	-2.04231
H	3.17959	-1.69858	-2.29506
H	-1.91014	5.82409	1.05604
C	-7.22738	-0.94546	0.06217
C	-8.01163	-0.68311	-1.07841
C	-7.83371	-1.1677	1.31133
C	-9.40142	-0.6464	-0.94463
C	-9.22946	-1.12218	1.39971
C	-10.03157	-0.86443	0.2856
H	-10.01078	-0.44062	-1.83012
H	-9.70144	-1.29346	2.37188
C	7.12416	-1.05834	0.12078
C	7.85428	0.11955	-0.13677
C	7.79086	-2.23664	0.50229
C	9.24518	0.09519	-0.00808
C	9.18532	-2.21758	0.62097
C	9.93161	-1.06351	0.3715
H	9.8101	1.01008	-0.2128
H	9.70209	-3.13551	0.91754
C	-7.36761	-0.44569	-2.41369
H	-6.70486	0.43429	-2.39139
H	-6.74281	-1.30102	-2.71682
H	-8.12367	-0.28248	-3.19425
C	-7.00331	-1.44444	2.53102
H	-6.37346	-2.33816	2.39585
H	-6.31908	-0.60917	2.75063
H	-7.63819	-1.60524	3.41339
C	-11.52796	-0.83597	0.39284
H	-11.94673	0.06486	-0.08241
H	-11.97673	-1.70438	-0.11767
H	-11.85832	-0.857	1.44107
C	7.15274	1.38221	-0.54904
H	6.55817	1.23164	-1.46446
H	6.45062	1.72537	0.22769
H	7.87272	2.19083	-0.73795
C	7.02357	-3.49681	0.78127
H	6.2722	-3.3435	1.57226
H	6.47351	-3.84018	-0.10941
H	7.69484	-4.3068	1.09946

C	11.4248	-1.05331	0.52148
H	11.7285	-0.47629	1.41106
H	11.82671	-2.07064	0.63146
H	11.91243	-0.58221	-0.34625
H	-1.15075	-3.17116	-0.77734
H	1.29172	-2.98651	-0.72713

OBP2⁺

E(PBE0/def2-SVP) = –2230.02451465

Thermal correction to Gibbs Free Energy = 0.728245

Sum of electronic and thermal Free Energies = –2229.297073

C	2.55071	-4.38647	0.24346
C	2.09048	-5.7041	0.23488
C	0.76182	-5.96238	-0.05232
C	-0.15291	-4.91659	-0.31931
C	0.2887	-3.56171	-0.24854
C	1.66914	-3.32678	0.00693
C	-1.49118	-5.19768	-0.6832
C	-2.34013	-4.16287	-1.00471
C	-1.91032	-2.83109	-0.90283
C	-0.62351	-2.48954	-0.47515
C	2.13135	-1.97801	-0.05276
C	-0.15675	-1.10296	-0.30693
O	3.43135	-1.81175	0.06621
C	1.25916	-0.88413	-0.21318
C	-0.99863	0.0253	-0.22862
C	1.81486	0.42902	-0.21868
C	3.25676	0.56994	-0.13108
C	-2.47606	-0.08818	-0.11444
C	3.95184	1.79303	-0.17055
C	5.33121	1.82715	-0.06987
C	6.08556	0.64513	0.07893
C	5.41643	-0.57396	0.12054
C	4.02869	-0.58962	0.0149
C	-3.04751	-0.62945	1.04291
C	-4.43001	-0.71252	1.17332
C	-5.27632	-0.26057	0.15204
C	-4.69845	0.28427	-1.00146
C	-3.31575	0.37645	-1.13205
C	0.94893	1.51174	-0.27217
C	-0.43609	1.33232	-0.22675
H	3.60504	-4.17501	0.42858
H	0.39698	-6.99199	-0.08933
H	-1.82159	-6.23782	-0.73233
H	-3.36113	-4.36456	-1.33517
H	-2.61517	-2.05372	-1.18257
H	3.40192	2.72799	-0.28335
H	5.85093	2.78712	-0.10367
H	5.95475	-1.51667	0.23397
H	-2.4009	-0.98626	1.84861
H	-4.86471	-1.13115	2.08468
H	-5.34378	0.64391	-1.807
H	-2.88193	0.80716	-2.03737
H	2.78254	-6.52431	0.43353
C	7.56871	0.70059	0.19195
C	8.35838	0.71103	-0.97506
C	8.16993	0.74751	1.46215
C	9.74776	0.76834	-0.84625
C	9.56578	0.80578	1.54471
C	10.37277	0.81677	0.40503
H	10.36109	0.77476	-1.75268
H	10.03341	0.84538	2.53304
C	-6.75564	-0.35074	0.29254
C	-7.42034	-1.54834	-0.03828
C	-7.4839	0.76231	0.75118

C	-8.80952	-1.61045	0.09771
C	-8.87409	0.65746	0.87368
C	-9.55646	-0.51897	0.55437
H	-9.32437	-2.54045	-0.16329
H	-9.43928	1.52425	1.22984
C	7.72036	0.65548	-2.33299
H	7.11876	-0.25928	-2.45721
H	7.03657	1.5042	-2.49352
H	8.47915	0.67488	-3.12764
C	7.33587	0.73874	2.71052
H	6.6193	1.57549	2.72293
H	6.7409	-0.18501	2.79203
H	7.96694	0.81591	3.60677
C	11.86833	0.87958	0.50897
H	12.33728	0.00314	0.03304
H	12.26527	1.77071	-0.00368
H	12.19909	0.91493	1.55664
C	-6.65215	-2.73823	-0.53862
H	-6.07289	-2.49098	-1.44287
H	-5.92635	-3.0951	0.20961
H	-7.3269	-3.571	-0.78196
C	-6.78547	2.04364	1.10639
H	-6.00734	1.87953	1.86873
H	-6.27702	2.48324	0.23346
H	-7.49573	2.78646	1.49583
C	-11.04529	-0.62279	0.71287
H	-11.30712	-1.24195	1.58746
H	-11.506	0.36518	0.85603
H	-11.51116	-1.09674	-0.16503
H	1.34055	2.52849	-0.26785
C	-1.27516	2.55087	-0.18226
C	-2.26301	2.75115	0.7995
C	-1.06918	3.56655	-1.12349
C	-3.01493	3.91237	0.82651
H	-2.43352	1.99187	1.56394
C	-1.83448	4.73089	-1.12185
H	-0.3099	3.43692	-1.89922
C	-2.81954	4.91135	-0.1424
H	-3.77359	4.07229	1.5957
H	-1.65905	5.48693	-1.8875
O	-3.60813	5.99127	-0.05153
C	-3.46327	7.02514	-0.997
H	-3.66646	6.67044	-2.02169
H	-4.20075	7.79411	-0.73381
H	-2.45445	7.47012	-0.96199

OBP3$^+$

E(PBE0/def2-SVP) = –2214.77350609

Thermal correction to Gibbs Free Energy = 0.688721

Sum of electronic and thermal Free Energies = –2214.086181

C	-2.38691	4.27167	0.36294
C	-1.89942	5.57862	0.39387
C	-0.56721	5.81852	0.10523
C	0.32383	4.76351	-0.20115
C	-0.14575	3.41663	-0.17251
C	-1.52924	3.20195	0.08411
C	1.66609	5.02851	-0.56249
C	2.49216	3.9875	-0.92193
C	2.03498	2.66246	-0.8619
C	0.74286	2.33436	-0.43907
C	-2.01981	1.86657	-0.01713
C	0.24849	0.9539	-0.3136
O	-3.32134	1.72177	0.10253
C	-1.16995	0.75942	-0.21672
C	1.06857	-0.19334	-0.27536

C	-1.75303	-0.5405	-0.25785
C	-3.19672	-0.65576	-0.16844
C	2.54838	-0.11622	-0.16132
C	-3.91675	-1.86283	-0.24195
C	-5.2963	-1.87109	-0.13769
C	-6.02578	-0.67874	0.04872
C	-5.33158	0.52457	0.12481
C	-3.94432	0.5144	0.0153
C	3.13242	0.3768	1.01131
C	4.51648	0.42355	1.1425
C	5.35169	-0.01702	0.10711
C	4.76096	-0.51359	-1.06155
C	3.37637	-0.56954	-1.19345
C	-0.9086	-1.63925	-0.34741
C	0.47781	-1.48478	-0.30574
H	-3.44377	4.076	0.55114
H	-0.18127	6.84108	0.09933
H	2.018	6.06255	-0.57917
H	3.51569	4.17891	-1.2506
H	2.72164	1.87968	-1.1708
H	-3.38729	-2.80557	-0.38506
H	-5.83588	-2.81873	-0.19815
H	-5.84969	1.47455	0.26787
H	2.49454	0.72449	1.82788
H	4.96122	0.80457	2.06542
H	5.39742	-0.86331	-1.87843
H	2.93239	-0.96204	-2.11118
H	-2.57238	6.40617	0.62487
C	-7.50944	-0.70556	0.16429
C	-8.3011	-0.682	-1.00122
C	-8.10938	-0.75716	1.43492
C	-9.69118	-0.70936	-0.8704
C	-9.50604	-0.78467	1.51938
C	-10.31502	-0.76102	0.38134
H	-10.30603	-0.68893	-1.77559
H	-9.97263	-0.82708	2.50808
C	6.8328	0.03657	0.24794
C	7.52255	1.22967	-0.04629
C	7.53771	-1.10553	0.66994
C	8.9129	1.25748	0.08801
C	8.93008	-1.03446	0.79239
C	9.63708	0.13622	0.50788
H	9.4472	2.18394	-0.14498
H	9.47697	-1.92387	1.12021
C	-7.66437	-0.62216	-2.35952
H	-7.02901	0.27155	-2.46696
H	-7.01361	-1.49273	-2.53948
H	-8.42511	-0.59733	-3.15213
C	-7.27376	-0.7858	2.68199
H	-6.56696	-1.63085	2.67494
H	-6.66824	0.1291	2.7837
H	-7.90495	-0.87543	3.57701
C	-11.81136	-0.79249	0.48745
H	-12.26328	0.08904	0.00475
H	-12.22668	-1.67985	-0.01732
H	-12.14146	-0.8132	1.53574
C	6.77939	2.45126	-0.50646
H	6.19442	2.24584	-1.41748
H	6.06191	2.79885	0.25406
H	7.47155	3.27694	-0.72358
C	6.81298	-2.38226	0.98685
H	6.03948	-2.22553	1.7555
H	6.29468	-2.78399	0.10151
H	7.50815	-3.15129	1.3519
C	11.1284	0.2026	0.66274
H	11.40803	0.81142	1.53896
H	11.56493	-0.79715	0.79933
H	11.60309	0.66895	-0.21464
H	-1.32061	-2.64758	-0.3732

C	1.29766	-2.72201	-0.29068
C	2.23484	-2.97487	0.72246
C	1.10979	-3.68474	-1.29216
C	2.97464	-4.1516	0.73078
H	2.38333	-2.24862	1.52271
C	1.85546	-4.86037	-1.30501
H	0.38412	-3.50325	-2.08868
C	2.77979	-5.07364	-0.29119
H	3.70075	-4.36211	1.51846
H	1.72954	-5.60827	-2.09041
F	3.49841	-6.19747	-0.29445

Structure optimizations of radicals OBP1•–OBP3•

OBP1•

E(PBE0/def2-SVP) = –1885.21603304

Thermal correction to Gibbs Free Energy = 0.618498

Sum of electronic and thermal Free Energies = –1884.597901

C	-1.83483	3.73924	0.66555
C	-1.26268	5.00204	0.81667
C	0.08162	5.20386	0.5459
C	0.90954	4.12881	0.12286
C	0.35601	2.81248	0.01402
C	-1.04398	2.64108	0.2636
C	2.26423	4.33723	-0.21987
C	3.02774	3.28618	-0.6961
C	2.49637	1.99764	-0.77985
C	1.17817	1.71243	-0.37683
C	-1.59976	1.36264	0.04314
C	0.60638	0.36575	-0.39371
O	-2.94377	1.27698	0.17834
C	-0.8169	0.23197	-0.27846
C	1.36499	-0.83136	-0.52457
C	-1.46345	-1.03005	-0.4521
C	-2.92109	-1.07314	-0.33445
C	2.84054	-0.90156	-0.36532
C	-3.69779	-2.22922	-0.51307
C	-5.0794	-2.1994	-0.384
C	-5.74619	-1.00421	-0.06758
C	-4.99077	0.15357	0.11546
C	-3.60419	0.10992	-0.01766
C	3.43831	-0.50904	0.84076
C	4.81691	-0.58133	1.00544
C	5.64251	-1.04747	-0.02758
C	5.04085	-1.46814	-1.21921
C	3.65806	-1.40346	-1.38431
C	-0.68571	-2.14852	-0.71053
C	0.7058	-2.0464	-0.71579
H	-2.89848	3.58683	0.8512
H	0.52513	6.19815	0.64033
H	2.68375	5.34284	-0.13465
H	4.05982	3.45659	-1.01293
H	3.1258	1.20798	-1.1811
H	-3.209	-3.17357	-0.75851
H	-5.6578	-3.11456	-0.53002
H	-5.4661	1.10508	0.36276
H	2.81233	-0.12998	1.65248
H	5.26701	-0.26482	1.9502
H	5.66671	-1.84099	-2.0345
H	3.20818	-1.71856	-2.32938
H	-1.8858	5.84091	1.13705
C	-7.22895	-0.96998	0.07014
C	-8.03153	-0.70192	-1.05693
C	-7.82181	-1.21013	1.32282
C	-9.4203	-0.6782	-0.90739
C	-9.21715	-1.17727	1.42863

C	-10.03472	-0.91254	0.32776
H	-10.0417	-0.47027	-1.78416
H	-9.67643	-1.3657	2.40387
C	7.12277	-1.07788	0.13489
C	7.87707	0.0813	-0.14013
C	7.76462	-2.25482	0.5603
C	9.26486	0.03895	0.01424
C	9.15732	-2.25448	0.70355
C	9.92636	-1.12011	0.43547
H	9.84774	0.93984	-0.20249
H	9.65363	-3.17129	1.03638
C	-7.40681	-0.44619	-2.39814
H	-6.73956	0.43005	-2.37111
H	-6.78878	-1.29808	-2.7235
H	-8.17362	-0.2677	-3.16504
C	-6.97682	-1.49809	2.5301
H	-6.34494	-2.38739	2.37693
H	-6.292	-0.66378	2.75075
H	-7.60098	-1.67103	3.41807
C	-11.52924	-0.8676	0.45868
H	-11.91083	0.15401	0.2955
H	-12.01646	-1.5131	-0.28922
H	-11.85704	-1.19245	1.45653
C	7.20283	1.34348	-0.59682
H	6.6246	1.17931	-1.52024
H	6.48827	1.71487	0.15504
H	7.93802	2.13745	-0.78968
C	6.97288	-3.49473	0.86151
H	6.20946	-3.30715	1.63325
H	6.43319	-3.8552	-0.0287
H	7.62555	-4.30538	1.21513
C	11.41901	-1.13337	0.59281
H	11.74856	-0.38694	1.33383
H	11.78157	-2.11823	0.92025
H	11.92237	-0.88413	-0.35546
H	-1.14816	-3.12486	-0.86125
H	1.30594	-2.95229	-0.82783

OBP2$^{\bullet}$

E(PBE0/def2-SVP) = –2230.17683516

Thermal correction to Gibbs Free Energy = 0.722438

Sum of electronic and thermal Free Energies = –2229.454975

C	2.55895	-4.41962	0.2427
C	2.08525	-5.73126	0.23057
C	0.75597	-5.99593	-0.05747
C	-0.15576	-4.94059	-0.33223
C	0.29454	-3.58218	-0.26904
C	1.682	-3.34246	-0.0065
C	-1.49436	-5.20838	-0.6955
C	-2.33966	-4.16651	-1.03034
C	-1.90932	-2.84062	-0.94453
C	-0.61416	-2.50471	-0.50747
C	2.13705	-2.00799	-0.06009
C	-0.14636	-1.12456	-0.33629
O	3.4734	-1.83801	0.07477
C	1.26614	-0.90973	-0.21726
C	-0.9971	0.01522	-0.27613
C	1.8105	0.40848	-0.21959
C	3.26416	0.54968	-0.1255
C	-2.47495	-0.09867	-0.15065
C	3.94792	1.77525	-0.16624
C	5.33061	1.83547	-0.06128
C	6.09174	0.66469	0.09052
C	5.42937	-0.56183	0.1308
C	4.04059	-0.60833	0.02361

C	-3.03855	-0.64296	1.00955
C	-4.4198	-0.717	1.15916
C	-5.2773	-0.25203	0.1533
C	-4.71012	0.29653	-1.0036
C	-3.32826	0.37815	-1.15154
C	0.94742	1.48666	-0.28339
C	-0.44661	1.31261	-0.26529
H	3.61104	-4.2116	0.43989
H	0.38815	-7.02437	-0.09268
H	-1.83453	-6.24602	-0.74083
H	-3.35949	-4.37151	-1.36587
H	-2.60373	-2.0603	-1.23997
H	3.38519	2.70282	-0.28347
H	5.83562	2.80353	-0.09559
H	5.98002	-1.49776	0.24568
H	-2.38388	-1.01179	1.80325
H	-4.8447	-1.14052	2.07314
H	-5.36321	0.66629	-1.79854
H	-2.90356	0.81077	-2.06034
H	2.77368	-6.55522	0.43489
C	7.57534	0.72791	0.20725
C	8.37075	0.73809	-0.95633
C	8.17603	0.77991	1.47796
C	9.76002	0.80044	-0.82479
C	9.57178	0.84281	1.56476
C	10.38217	0.85371	0.42757
H	10.37569	0.80653	-1.72981
H	10.03687	0.88556	2.55436
C	-6.75529	-0.33553	0.31253
C	-7.4297	-1.53	-0.01139
C	-7.47433	0.77885	0.78273
C	-8.81732	-1.5873	0.14254
C	-8.86336	0.67901	0.92354
C	-9.55437	-0.49405	0.61088
H	-9.33886	-2.51509	-0.11337
H	-9.42049	1.54699	1.2896
C	7.738	0.67892	-2.31671
H	7.12408	-0.22809	-2.43556
H	7.06521	1.53458	-2.48629
H	8.50015	0.68226	-3.10861
C	7.33961	0.77164	2.72492
H	6.62302	1.60829	2.7347
H	6.74265	-0.15085	2.80447
H	7.96823	0.84857	3.62316
C	11.8777	0.92052	0.53515
H	12.35038	0.04168	0.06718
H	12.27513	1.80894	0.01802
H	12.20546	0.96346	1.58359
C	-6.67278	-2.7218	-0.52421
H	-6.10689	-2.47578	-1.43714
H	-5.93454	-3.08011	0.21087
H	-7.3533	-3.55307	-0.75668
C	-6.76711	2.0571	1.13192
H	-5.97697	1.88791	1.88058
H	-6.27055	2.49873	0.25324
H	-7.4691	2.80051	1.5353
C	-11.04175	-0.59276	0.78729
H	-11.29629	-1.22843	1.65211
H	-11.49482	0.39475	0.95611
H	-11.52205	-1.04566	-0.0941
H	1.33953	2.50432	-0.27431
C	-1.2872	2.53719	-0.21737
C	-2.22859	2.76258	0.80216
C	-1.13292	3.53004	-1.19018
C	-2.98948	3.92031	0.83342
H	-2.36279	2.01842	1.58927
C	-1.89759	4.69763	-1.17791
H	-0.40884	3.38331	-1.99631
C	-2.83934	4.89835	-0.16246

H	-3.71701	4.09318	1.62974
H	-1.75457	5.43813	-1.96558
O	-3.62869	5.98303	-0.06022
C	-3.51594	6.99665	-1.02802
H	-3.75892	6.62677	-2.03915
H	-4.23942	7.77516	-0.75269
H	-2.50487	7.43909	-1.04166

OBP3•

E(PBE0/def2-SVP) = –2214.92800802

Thermal correction to Gibbs Free Energy = 0.683780

Sum of electronic and thermal Free Energies = –2214.244727

C	-2.38506	4.30036	0.42826
C	-1.88268	5.60081	0.46777
C	-0.55046	5.84879	0.17873
C	0.3354	4.78675	-0.14928
C	-0.14424	3.43711	-0.13976
C	-1.53434	3.2163	0.12493
C	1.67661	5.0405	-0.5131
C	2.4962	3.99583	-0.89909
C	2.03752	2.67705	-0.86549
C	0.73876	2.35158	-0.43163
C	-2.01941	1.89545	0.01761
C	0.24201	0.97638	-0.31385
O	-3.35768	1.74835	0.15742
C	-1.17392	0.78652	-0.19377
C	1.06831	-0.18255	-0.30344
C	-1.74742	-0.51814	-0.2499
C	-3.20385	-0.63057	-0.16273
C	2.54909	-0.10688	-0.18432
C	-3.91565	-1.83609	-0.26876
C	-5.29949	-1.86922	-0.16766
C	-6.03321	-0.69071	0.04686
C	-5.34266	0.5162	0.15232
C	-3.95327	0.53607	0.0456
C	3.13263	0.37924	0.99178
C	4.51615	0.41658	1.13315
C	5.3559	-0.02771	0.10315
C	4.76857	-0.51902	-1.06928
C	3.38413	-0.56401	-1.20948
C	-0.90829	-1.61172	-0.3586
C	0.48837	-1.46566	-0.33761
H	-3.43983	4.10703	0.6261
H	-0.1605	6.86962	0.18396
H	2.03918	6.0715	-0.5176
H	3.51747	4.1929	-1.23491
H	2.7121	1.89489	-1.19976
H	-3.3749	-2.76884	-0.43776
H	-5.82726	-2.8215	-0.2569
H	-5.87148	1.4572	0.31746
H	2.49195	0.73194	1.804
H	4.95692	0.79538	2.05914
H	5.40765	-0.87173	-1.88312
H	2.94355	-0.95084	-2.13133
H	-2.55096	6.42996	0.71364
C	-7.51818	-0.72532	0.15728
C	-8.31195	-0.50985	-0.98728
C	-8.12174	-0.97728	1.40248
C	-9.70272	-0.55134	-0.86266
C	-9.5188	-1.0093	1.48327
C	-10.32778	-0.79888	0.36462
H	-10.31721	-0.38602	-1.75329
H	-9.98633	-1.2047	2.45318
C	6.83658	0.01952	0.2522
C	7.53328	1.20951	-0.04091

C	7.53547	-1.1226	0.68347
C	8.92262	1.23334	0.10286
C	8.92763	-1.05583	0.81568
C	9.64057	0.11063	0.53026
H	9.46141	2.15799	-0.12751
H	9.46899	-1.94498	1.15345
C	-7.67596	-0.24365	-2.32116
H	-7.06817	0.675	-2.30216
H	-6.99682	-1.06042	-2.61313
H	-8.43588	-0.13259	-3.10738
C	-7.28675	-1.20571	2.6293
H	-6.6254	-2.07869	2.51086
H	-6.63159	-0.34451	2.83634
H	-7.91895	-1.37438	3.5124
C	-11.82485	-0.82837	0.46757
H	-12.2583	0.15874	0.23679
H	-12.26084	-1.54306	-0.24867
H	-12.15434	-1.11311	1.47701
C	6.79676	2.4325	-0.50786
H	6.21856	2.2279	-1.42326
H	6.07215	2.78128	0.24518
H	7.49226	3.25697	-0.7192
C	6.80452	-2.39556	1.0016
H	6.02483	-2.23272	1.76258
H	6.2916	-2.7999	0.11435
H	7.49435	-3.16514	1.37582
C	11.13365	0.16828	0.67329
H	11.43652	0.9549	1.3833
H	11.54197	-0.78751	1.03148
H	11.6173	0.40436	-0.28863
H	-1.32256	-2.62012	-0.38948
C	1.30484	-2.70876	-0.33169
C	2.21842	-2.98915	0.69558
C	1.14382	-3.65155	-1.35654
C	2.96281	-4.1645	0.69396
H	2.34914	-2.27875	1.51351
C	1.88578	-4.83086	-1.37586
H	0.43757	-3.45163	-2.1663
C	2.78929	-5.06623	-0.34889
H	3.67395	-4.38964	1.49151
H	1.77461	-5.56304	-2.1784
F	3.50897	-6.19337	-0.36006

Structure optimizations of adducts A1–A3

A1

E(PBE0/def2-SVP) = –2107.99443772

Thermal correction to Gibbs Free Energy = 0.629863

Sum of electronic and thermal Free Energies = –2107.366052

C	1.83305	3.15354	0.17507
C	-0.15246	4.57191	0.05659
C	-0.93833	3.41059	-0.35164
C	-0.33979	2.11028	-0.32432
C	1.06165	1.98519	-0.07271
C	-2.25458	3.52398	-0.82582
C	-2.9675	2.40608	-1.23417
C	-2.41489	1.13797	-1.11554
C	-1.11571	0.95161	-0.61811
C	1.65742	0.72646	-0.15826
C	-0.51445	-0.37543	-0.47302

O	2.98579	0.68394	-0.02958
C	0.90406	-0.46634	-0.34775
C	1.58637	-1.71457	-0.36589
C	3.0396	-1.70876	-0.24363
C	3.84891	-2.85684	-0.27625
C	5.22706	-2.76518	-0.15792
C	5.07153	-0.36987	0.04063
H	2.90793	3.05299	0.33223
H	-2.7252	4.49954	-0.92352
H	-3.97004	2.52331	-1.65039
H	-2.9988	0.28374	-1.44919
H	3.3893	-3.83884	-0.39802
H	5.83447	-3.6725	-0.18845
H	5.51686	0.61925	0.16425
C	3.689	-0.47892	-0.08071
C	5.85913	-1.51885	0.00124
C	-1.24854	-1.59081	-0.45441
C	-2.72236	-1.66651	-0.29079
C	-3.33354	-1.10757	0.84053
C	-3.52343	-2.3251	-1.23048
C	-4.71246	-1.17178	1.00367
H	-2.71944	-0.6059	1.59258
C	-4.90667	-2.38112	-1.06724
H	-3.06189	-2.77036	-2.11566
C	-5.52322	-1.79481	0.04416
H	-5.17452	-0.725	1.88793
H	-5.52116	-2.87742	-1.82303
C	7.34027	-1.42467	0.12357
C	8.12982	-1.29847	-1.03331
C	7.94209	-1.46431	1.39715
C	9.52016	-1.21237	-0.89616
C	9.33296	-1.37527	1.48861
C	10.14192	-1.24849	0.35377
H	10.13352	-1.11359	-1.79692
H	9.79878	-1.40624	2.47858
C	-7.00443	-1.81377	0.19828
C	-7.76264	-0.70822	-0.23889
C	-7.64202	-2.92447	0.77942
C	-9.15087	-0.73691	-0.08628
C	-9.03538	-2.91218	0.91491
C	-9.80851	-1.82988	0.48943
H	-9.73695	0.12215	-0.42796
H	-9.52889	-3.77658	1.36981
C	11.63399	-1.15664	0.4865
H	11.92597	-0.29782	1.11231
H	12.04674	-2.05746	0.96917
H	12.12024	-1.04405	-0.49296
C	7.49753	-1.2542	-2.39442
H	6.91535	-2.16694	-2.59867
H	6.79618	-0.40948	-2.4857
H	8.2583	-1.15193	-3.18095
C	7.10534	-1.59973	2.63636
H	6.40199	-0.75831	2.74174
H	6.4944	-2.51614	2.61192
H	7.73454	-1.6324	3.53687
C	-6.84527	-4.10568	1.25362
H	-6.30485	-4.58795	0.42346
H	-6.08253	-3.80688	1.99017
H	-7.49471	-4.86004	1.71975
C	-7.09265	0.48249	-0.86307
H	-6.39398	0.96697	-0.1623
H	-6.49876	0.19379	-1.74509
H	-7.83196	1.23201	-1.17862
C	-11.30152	-1.82802	0.64339
H	-11.63465	-0.99446	1.28305
H	-11.80291	-1.70234	-0.32993
H	-11.66223	-2.76423	1.09276
C	-0.67672	5.85884	0.29857
C	0.19455	6.93145	0.63362

C	-2.04423	6.23079	0.32466
N	0.89589	7.81857	0.90662
N	-3.14788	6.59744	0.37762
C	1.25293	4.3843	0.24338
H	1.8722	5.25191	0.47675
C	-0.5551	-2.80369	-0.49915
C	0.83329	-2.87751	-0.4876
H	1.31705	-3.85406	-0.52252
H	-1.13489	-3.72971	-0.50407

A2

E(PBE0/def2-SVP) = –2452.95675578

Thermal correction to Gibbs Free Energy = 0.730653

Sum of electronic and thermal Free Energies = –2452.226986

C	2.64099	3.73054	0.12582
C	0.82694	5.36012	0.02793
C	-0.09026	4.29159	-0.35767
C	0.35383	2.93114	-0.31579
C	1.73589	2.65435	-0.07947
C	-1.38649	4.55379	-0.82807
C	-2.22509	3.52079	-1.21762
C	-1.82438	2.19818	-1.07832
C	-0.55481	1.86383	-0.5819
C	2.18015	1.33318	-0.13988
C	-0.11128	0.47595	-0.39951
O	3.49688	1.13958	-0.02712
C	1.29042	0.23429	-0.28315
C	1.81946	-1.08399	-0.26195
C	3.26658	-1.24737	-0.17141
C	3.93516	-2.48294	-0.18745
C	5.31661	-2.55074	-0.09435
C	5.44643	-0.14942	0.03816
H	3.70063	3.50974	0.26229
H	-1.73848	5.57704	-0.93941
H	-3.20903	3.74583	-1.63406
H	-2.50851	1.41715	-1.39691
H	3.36233	-3.40713	-0.27607
H	5.81291	-3.52363	-0.10952
H	6.00691	0.7834	0.12548
C	4.05835	-0.09843	-0.05693
C	6.09313	-1.38367	0.02055
C	-0.98306	-0.64167	-0.32468
C	-2.46044	-0.50001	-0.22934
C	-3.0392	0.08121	0.90482
C	-3.29754	-0.98722	-1.23905
C	-4.42241	0.17648	1.02242
H	-2.39716	0.45902	1.70458
C	-4.68046	-0.88374	-1.12255
H	-2.85973	-1.44742	-2.12784
C	-5.26437	-0.3027	0.0102
H	-4.86059	0.62602	1.91736
H	-5.32138	-1.26279	-1.92285
C	7.57676	-1.46221	0.12256
C	8.3577	-1.49897	-1.0488
C	8.18996	-1.49658	1.38875
C	9.7486	-1.57076	-0.93058
C	9.58484	-1.56991	1.46145
C	10.3831	-1.60959	0.31507
H	10.35432	-1.59419	-1.84179
H	10.06011	-1.59536	2.44681
C	-6.74439	-0.20464	0.13769
C	-7.39924	1.0005	-0.18514
C	-7.48409	-1.31657	0.58239
C	-8.78927	1.07052	-0.05742
C	-8.87395	-1.20355	0.69763

C	-9.54689	-0.02065	0.38096
H	-9.29553	2.00778	-0.30902
H	-9.44659	-2.06765	1.04866
C	11.87647	-1.71914	0.41465
H	12.24514	-1.34861	1.38223
H	12.20061	-2.76972	0.32041
H	12.37673	-1.15469	-0.38657
C	7.71135	-1.45317	-2.40324
H	7.01787	-2.29672	-2.54837
H	7.1174	-0.53455	-2.53473
H	8.46456	-1.48975	-3.20272
C	7.36626	-1.4535	2.64336
H	6.78261	-0.52152	2.7112
H	6.63929	-2.28056	2.67664
H	8.00297	-1.52138	3.53655
C	-6.796	-2.60402	0.9372
H	-6.28643	-3.04305	0.06481
H	-6.0202	-2.44681	1.70324
H	-7.51329	-3.3434	1.32081
C	-6.62048	2.19161	-0.66589
H	-5.90908	2.54667	0.09673
H	-6.02282	1.9466	-1.5586
H	-7.28966	3.02564	-0.92009
C	-11.04143	0.07383	0.48226
H	-11.35711	1.04425	0.89487
H	-11.51065	-0.02047	-0.51189
H	-11.45435	-0.72272	1.11808
C	0.44702	6.69566	0.27885
C	1.43496	7.67282	0.57888
C	-0.87503	7.20025	0.35726
N	2.23477	8.48176	0.82275
N	-1.93556	7.67066	0.45429
C	2.20661	5.02107	0.18245
H	2.92467	5.81693	0.38685
C	-0.44896	-1.94916	-0.28259
C	0.93627	-2.15201	-0.3008
H	1.30999	-3.17579	-0.27152
C	-1.31729	-3.15121	-0.2083
C	-1.18436	-4.17444	-1.16101
C	-2.26949	-3.31795	0.80341
C	-1.99526	-5.2997	-1.12407
H	-0.4476	-4.07278	-1.9624
C	-3.08183	-4.44734	0.86122
H	-2.38382	-2.55115	1.57154
C	-2.96005	-5.44444	-0.11596
H	-1.90477	-6.0848	-1.87805
H	-3.80895	-4.53816	1.66874
O	-3.71269	-6.55655	-0.16099
C	-4.71902	-6.736	0.80593
H	-5.21484	-7.68617	0.56862
H	-4.30167	-6.7973	1.8256
H	-5.46704	-5.92496	0.77193

A3

E(PBE0/def2-SVP) = –2437.70696754

Thermal correction to Gibbs Free Energy = 0.694338

Sum of electronic and thermal Free Energies = –2437.013489

C	2.37625	3.68699	0.10489
C	0.49211	5.23389	-0.01826
C	-0.37558	4.12189	-0.39619
C	0.12822	2.78301	-0.3383
C	1.52053	2.56961	-0.09568
C	-1.68038	4.32086	-0.87335
C	-2.47174	3.24752	-1.25356
C	-2.01366	1.94577	-1.09836

C	-0.73158	1.67419	-0.5953
C	2.0233	1.26968	-0.14505
C	-0.22743	0.30994	-0.39918
O	3.34708	1.13465	-0.0303
C	1.18283	0.13073	-0.27975
C	1.76964	-1.16228	-0.24657
C	3.22222	-1.26163	-0.15288
C	3.94445	-2.46683	-0.15804
C	5.32749	-2.47261	-0.06379
C	5.35113	-0.0668	0.0489
H	3.44386	3.51474	0.249
H	-2.07723	5.32605	-0.99699
H	-3.46322	3.42463	-1.67515
H	-2.66102	1.13099	-1.4098
H	3.41382	-3.41667	-0.23984
H	5.86658	-3.42255	-0.07109
H	5.86983	0.89047	0.12913
C	3.96237	-0.07783	-0.04792
C	6.05155	-1.27143	0.04182
C	-1.05053	-0.84404	-0.31323
C	-2.53114	-0.76313	-0.20263
C	-3.11675	-0.18687	0.93078
C	-3.3619	-1.29396	-1.19532
C	-4.50078	-0.13363	1.06125
H	-2.47902	0.22404	1.71753
C	-4.74648	-1.23277	-1.06566
H	-2.91863	-1.75092	-2.08311
C	-5.33694	-0.6512	0.06322
H	-4.94449	0.31599	1.9534
H	-5.38329	-1.64323	-1.85364
C	7.53716	-1.2834	0.14417
C	8.31846	-1.27522	-1.0251
C	8.15114	-1.30585	1.41238
C	9.713	-1.28943	-0.90558
C	9.54581	-1.31991	1.48616
C	10.34679	-1.31165	0.33865
H	10.3202	-1.28216	-1.81586
H	10.02108	-1.33812	2.47191
C	-6.81786	-0.58566	0.20089
C	-7.5062	0.58299	-0.18329
C	-7.52398	-1.68863	0.71405
C	-8.89572	0.62509	-0.04619
C	-8.916	-1.60421	0.8365
C	-9.62118	-0.45818	0.46262
H	-9.42834	1.53301	-0.34661
H	-9.46357	-2.46232	1.23832
C	11.84313	-1.3261	0.4525
H	12.20809	-0.44889	1.01105
H	12.1924	-2.21836	0.99697
H	12.32338	-1.32223	-0.53633
C	7.6732	-1.24837	-2.38055
H	7.02002	-2.12215	-2.53398
H	7.03753	-0.35733	-2.5062
H	8.42884	-1.24338	-3.17855
C	7.32341	-1.31543	2.66493
H	6.69198	-0.41554	2.7377
H	6.64019	-2.17929	2.68984
H	7.96061	-1.35725	3.55936
C	-6.80101	-2.93828	1.12847
H	-6.28771	-3.41025	0.27542
H	-6.02291	-2.72392	1.87832
H	-7.49627	-3.67442	1.55603
C	-6.76124	1.76342	-0.73808
H	-6.0376	2.16464	-0.01062
H	-6.18143	1.48774	-1.63367
H	-7.45132	2.57339	-1.01326
C	-11.11405	-0.38084	0.59791
H	-11.41236	0.45487	1.25157
H	-11.59424	-0.2079	-0.37901

H	-11.5297	-1.30684	1.02021
C	0.05414	6.5542	0.21503
C	0.99706	7.57611	0.51202
C	-1.2886	7.00433	0.27504
N	1.75881	8.42158	0.75348
N	-2.36848	7.43161	0.35568
C	1.88478	4.95695	0.14773
H	2.56587	5.78549	0.34875
C	-0.45814	-2.12391	-0.26567
C	0.9338	-2.2682	-0.28037
H	1.35176	-3.27436	-0.2456
C	-1.27188	-3.36563	-0.1824
C	-1.10694	-4.36894	-1.1466
C	-2.18697	-3.57739	0.85917
C	-1.85073	-5.54567	-1.09358
H	-0.39816	-4.22056	-1.96511
C	-2.92984	-4.75095	0.9307
H	-2.3191	-2.81656	1.63018
C	-2.75526	-5.71556	-0.05448
H	-1.73941	-6.32675	-1.84849
H	-3.64224	-4.92575	1.73958
F	-3.47497	-6.8396	0.00372

Mechanistic Investigations

5a(S_0)

E(CAM-B3LYP/6-311+G**) = –1888.60078364

Thermal correction to Gibbs Free Energy = 0.627107

Sum of electronic and thermal Free Energies = –1887.973681

C	0.00681300	-0.23852200	0.48776500
C	0.09486400	-0.14028600	1.88328900
C	1.32258800	-0.02336000	2.47922700
C	2.50905500	-0.01877800	1.71104300
C	2.42376800	-0.17161000	0.30213300
C	1.14207800	-0.24930000	-0.29047700
C	3.77522500	0.16337500	2.31402400
C	4.89364800	0.21910200	1.53475400
C	4.80684800	0.03510600	0.14205600
C	3.60737900	-0.21122800	-0.49189800
C	1.00143100	-0.29092400	-1.76234300
C	3.49839200	-0.40413000	-1.96166800
O	-0.09035600	-0.46205700	-2.27298500
C	2.24309900	-0.19577600	-2.58460500
C	2.13806300	-0.07239400	-3.98434500
C	0.90004800	0.33957700	-4.70419400
C	0.31808100	1.58339700	-4.46840800
C	-0.78410800	1.99956300	-5.19690100
C	-0.75303700	-0.05109600	-6.42320500
C	3.27286400	-0.28824700	-4.75135900
C	4.46144400	-0.66997900	-4.15683900
H	-0.95622700	-0.29340200	-0.00377700
H	1.40295200	0.07946100	3.55581000
H	3.83608800	0.28196200	3.38985700
H	5.86496900	0.39777500	1.98001400
H	5.72036000	0.09676600	-0.42654000
H	0.73241200	2.23314800	-3.70591800
H	-1.22128000	2.97182900	-4.99739600
H	-1.16928000	-0.70194100	-7.18436100
H	-0.80779000	-0.13720900	2.48192600
H	3.21626000	-0.18128500	-5.82766400
H	5.30990800	-0.92602500	-4.78028800
C	0.35790700	-0.46564000	-5.70114500
H	0.79427100	-1.43765800	-5.90113700

C	-1.34297000	1.18649300	-6.18267700
C	4.60235200	-0.74591500	-2.77658400
C	5.90430800	-1.28621200	-2.28600800
C	7.10854500	-0.67132400	-2.62179800
C	5.95063300	-2.47428100	-1.55724100
C	8.32200000	-1.21701000	-2.22392100
H	7.09782600	0.25261600	-3.18915800
C	7.16324300	-3.02014300	-1.16677700
H	5.02730800	-2.97823500	-1.29613600
C	8.36976200	-2.39926700	-1.48975900
H	9.24634900	-0.71627700	-2.49020300
H	7.17515900	-3.94784400	-0.60537300
C	-2.54115000	1.63170300	-6.95780000
C	-3.82812400	1.32478000	-6.49520000
C	-2.38118800	2.35179600	-8.15003100
C	-4.93234600	1.74410700	-7.23300200
C	-3.51043200	2.75229900	-8.85848200
C	-4.79562000	2.46059800	-8.41579100
H	-5.92662900	1.50090400	-6.87143400
H	-3.38009000	3.30619500	-9.78310800
C	9.67616000	-2.99226200	-1.06981000
C	10.24745300	-2.63696800	0.15944500
C	10.32907900	-3.91000400	-1.90466000
C	11.46345700	-3.20373500	0.53180000
C	11.54307900	-4.45422700	-1.49571100
C	12.12907800	-4.11226800	-0.28239400
H	11.89973700	-2.92734700	1.48656200
H	12.04271200	-5.16711300	-2.14434600
C	-1.01121100	2.69219000	-8.67591500
H	-0.44327900	3.29075500	-7.95969800
H	-0.42210100	1.79312800	-8.87252900
H	-1.08376900	3.25790900	-9.60585300
C	-4.03298300	0.54690800	-5.22174900
H	-3.58374500	-0.44730300	-5.28151500
H	-3.57182200	1.04572800	-4.36629200
H	-5.09632100	0.42481800	-5.01056500
C	-6.00253500	2.93172800	-9.18155300
H	-6.28085900	3.94812600	-8.88626800
H	-5.81231900	2.94527600	-10.25664500
H	-6.86611600	2.28976900	-8.99806900
C	9.56793400	-1.65830300	1.08127600
H	9.43912400	-0.68273800	0.60591700
H	8.57281700	-2.00407400	1.37149400
H	10.15307000	-1.51448100	1.99056600
C	9.73738600	-4.31622300	-3.22904200
H	8.75236500	-4.77288600	-3.10632300
H	9.60670200	-3.45681800	-3.89109600
H	10.38158000	-5.03611400	-3.73547600
C	13.45856900	-4.68843600	0.12354900
H	13.56368800	-4.72174100	1.20952900
H	13.58787000	-5.70178500	-0.26148600
H	14.28208500	-4.08276100	-0.26709900

5a($S_{min\ 1}$)

E(CAM-B3LYP/6-311+G**) = –1888.57750170

Thermal correction to Gibbs Free Energy = 0.625098

Sum of electronic and thermal Free Energies = –1887.866322

C	-0.03532400	-0.10268600	0.10870500
C	-0.01992600	-0.16181800	1.50726600
C	1.16750600	-0.16054600	2.18990500
C	2.40447300	-0.12901500	1.49483300
C	2.40966900	-0.14159400	0.07231200
C	1.15658900	-0.08569000	-0.60787300
C	3.63247800	-0.05897300	2.18423000
C	4.80859700	0.02849100	1.48610200

C	4.81572000	-0.02319200	0.08851500
C	3.64283900	-0.16402200	-0.64115900
C	1.15641900	0.07488400	-2.03870000
C	3.61045900	-0.29233800	-2.10886500
O	0.02212500	0.27730900	-2.62231200
C	2.37885100	-0.02510100	-2.77598400
C	2.30886400	0.01113900	-4.19551000
C	1.07191600	0.41330800	-4.87045100
C	0.42100900	1.61644500	-4.51734300
C	-0.74702600	2.01116500	-5.16390100
C	-0.63973600	0.03830700	-6.53105500
C	3.42823700	-0.33725800	-4.93082400
C	4.59829800	-0.70559000	-4.28853300
H	-0.97404000	-0.05587600	-0.42621900
H	1.18218100	-0.17187400	3.27371800
H	3.62704000	-0.04753000	3.26835100
H	5.74965500	0.13032600	2.01393200
H	5.76416200	0.05193400	-0.41767900
H	0.87968500	2.27355800	-3.79194600
H	-1.22276300	2.94287800	-4.88247900
H	-1.06832600	-0.57831500	-7.31258900
H	-0.95810000	-0.18568200	2.04952700
H	3.39008000	-0.31315600	-6.01340100
H	5.45104300	-1.02489600	-4.87453700
C	0.51927100	-0.35742700	-5.90726900
H	0.99856800	-1.28665400	-6.19000200
C	-1.29839000	1.23185500	-6.16463700
C	4.70562100	-0.70383200	-2.89694100
C	5.98289200	-1.26198400	-2.36098800
C	7.20206900	-0.64709600	-2.63467100
C	5.99191900	-2.46163100	-1.65086700
C	8.39488100	-1.20279200	-2.18994200
H	7.21866200	0.28434900	-3.18941900
C	7.18405900	-3.01804300	-1.21491500
H	5.05532900	-2.96268600	-1.43576700
C	8.40561100	-2.39582200	-1.47209600
H	9.33188800	-0.70132100	-2.40613300
H	7.16777400	-3.95323800	-0.66613200
C	-2.55769400	1.63899000	-6.85472700
C	-3.79772800	1.35443500	-6.26704100
C	-2.49812400	2.29746200	-8.09078200
C	-4.96067900	1.73640300	-6.92995200
C	-3.68487600	2.66570300	-8.71709900
C	-4.92651900	2.39625500	-8.15255600
H	-5.91983000	1.50799400	-6.47620700
H	-3.63605500	3.17720000	-9.67325600
C	9.68920500	-2.99753300	-0.99747600
C	10.20415100	-2.65376200	0.25978100
C	10.37657500	-3.91239200	-1.80745900
C	11.39898100	-3.22895600	0.68419800
C	11.56798300	-4.46512900	-1.34655500
C	12.09813400	-4.13450000	-0.10478400
H	11.79132100	-2.96162000	1.66038100
H	12.09446800	-5.17558000	-1.97641000
C	-1.17766800	2.61508500	-8.74263500
H	-0.54204900	3.21747000	-8.08913800
H	-0.61722800	1.70800800	-8.98282000
H	-1.32787600	3.16920200	-9.66990200
C	-3.89113500	0.63974100	-4.94445100
H	-3.34633200	-0.30711400	-4.95713100
H	-3.46560300	1.23533100	-4.13319300
H	-4.93133200	0.42775900	-4.69422900
C	-6.19564600	2.83166200	-8.83328500
H	-6.46035200	3.85405800	-8.54666500
H	-6.09154300	2.81400500	-9.91982600
H	-7.03438600	2.18824000	-8.56158500
C	9.48644300	-1.67898000	1.15645200
H	9.38773200	-0.69807800	0.68499800
H	8.47563100	-2.01988900	1.39298100

H	10.02663000	-1.54801200	2.09506500
C	9.84515600	-4.30657700	-3.16074600
H	8.85629300	-4.76533800	-3.08613000
H	9.74252000	-3.44090200	-3.81952800
H	10.51221500	-5.02104200	-3.64493300
C	13.40506800	-4.71905600	0.35846700
H	13.45831300	-4.76239300	1.44788900
H	13.54934900	-5.72950600	-0.02893600
H	14.24824900	-4.11296000	0.01297400

5a($S_{react\ 1}$)

E(CAM-B3LYP/6-311+G**) = –1888.58824320

Thermal correction to Gibbs Free Energy = 0.626173

Sum of electronic and thermal Free Energies = –1887.860763

C	1.20290100	4.25187000	-1.01463200
C	0.52468900	5.45254500	-0.88992600
C	-0.70529300	5.50149600	-0.25507900
C	-1.29370900	4.32754800	0.26188200
C	-0.60881200	3.08072500	0.11108900
C	0.64842800	3.06319600	-0.50634200
C	-2.52598100	4.35097700	0.93643300
C	-3.06659800	3.18876300	1.48814800
C	-2.44463200	1.97574100	1.32199500
C	-1.23474100	1.86341000	0.56313200
C	1.42256900	1.82023200	-0.59693100
C	-0.59958800	0.61963900	0.29860700
O	2.53599900	1.82085700	-1.13375000
C	0.79883100	0.60884200	-0.05929400
C	-1.28719000	-0.64124600	0.43399800
C	1.52652700	-0.58727400	0.08794200
C	3.00826100	-0.68251000	0.02758400
C	-2.74782500	-0.77800200	0.32080800
C	3.81216200	0.09481100	0.86115800
C	5.18642800	-0.06143200	0.86438500
C	5.80817000	-0.98870500	0.02639900
C	5.00450500	-1.76607100	-0.80203800
C	-3.46347700	-0.06389200	-0.65027900
C	-4.82731800	-0.24875300	-0.80571500
C	-5.53054100	-1.13224100	0.00999200
C	-4.81952600	-1.84183000	0.98202700
C	-3.45440800	-1.67942400	1.12784000
C	0.84391800	-1.76791600	0.39623200
C	-0.53711800	-1.80312000	0.52083800
H	2.17170600	4.19493600	-1.49236500
H	-1.23372900	6.44177200	-0.14900900
H	-3.03574500	5.29950500	1.06449600
H	-3.97750400	3.24715100	2.07177700
H	-2.84957100	1.10560200	1.81364700
H	3.35454300	0.83029800	1.51168600
H	5.79248700	0.55189700	1.52200500
H	5.46343500	-2.49169000	-1.46451200
H	-2.93796400	0.61632200	-1.30832700
H	-5.35561800	0.30254400	-1.57513900
H	-5.34912000	-2.52911300	1.63205000
H	-2.93048100	-2.23360000	1.89747800
H	0.96196000	6.36205800	-1.28529700
C	7.29525900	-1.13745000	0.01836400
C	8.06626100	-0.40665500	-0.89700600
C	7.92158700	-2.00589400	0.92237100
C	9.45000000	-0.55736000	-0.88989600
C	9.30847700	-2.12862100	0.89667500
C	10.09135700	-1.41104800	0.00034600
H	10.04175600	0.00974900	-1.60183900
H	9.78788600	-2.80583900	1.59671800
C	-7.00345000	-1.32123800	-0.15058600

C	-7.89578600	-0.51120700	0.56479700
C	-7.49196800	-2.31231800	-1.01268300
C	-9.26442500	-0.70684500	0.40434500
C	-8.86799400	-2.47456200	-1.14514500
C	-9.77133400	-1.68101900	-0.44782900
H	-9.95213300	-0.07979000	0.96303400
H	-9.24253800	-3.24461400	-1.81217600
C	7.42190200	0.53087900	-1.88408800
H	6.76497600	-0.00281400	-2.57523300
H	6.80446600	1.28062600	-1.38455300
H	8.17881000	1.05107700	-2.47282800
C	7.12003800	-2.80896000	1.91343100
H	6.55781300	-2.16337500	2.59250900
H	6.39120200	-3.45128900	1.41331600
H	7.77245000	-3.44325400	2.51528500
C	11.59149800	-1.53146100	0.01100200
H	12.00787300	-1.41494300	-0.99158700
H	12.04105600	-0.75930500	0.64297700
H	11.91090700	-2.49991800	0.40050000
C	-7.39846900	0.55785000	1.50234700
H	-6.79208300	0.13548300	2.30761000
H	-6.77360000	1.28852400	0.98298000
H	-8.23371200	1.09209400	1.95676000
C	-6.55593400	-3.20033200	-1.78997200
H	-5.90344300	-2.61982800	-2.44665600
H	-5.90622300	-3.77772300	-1.12759900
H	-7.11492200	-3.90375100	-2.40819900
C	-11.25473400	-1.85250700	-0.63202500
H	-11.62283300	-1.21637400	-1.44280100
H	-11.50822500	-2.88366700	-0.88494200
H	-11.80280300	-1.57974600	0.27174900
H	1.41205100	-2.68378400	0.50381700
H	-1.03942400	-2.75719800	0.61804300
C	3.62333000	-1.62537600	-0.79243400
H	3.01984000	-2.23873400	-1.45187000

5a($S_{TS\ 1}$)

E(CAM-B3LYP/6-311+G**) = –1888.55260302

Thermal correction to Gibbs Free Energy = 0.628072

Sum of electronic and thermal Free Energies = –1887.862758

C	-1.56211200	3.14900500	0.53512000
C	-0.87565600	4.36907700	0.55479900
C	0.45183100	4.42443400	0.22168700
C	1.16149300	3.24578100	-0.12325700
C	0.49648100	1.98952400	-0.07778500
C	-0.89899000	1.96988100	0.21778400
C	2.50813500	3.29551900	-0.54068100
C	3.14933200	2.14882300	-0.92790700
C	2.50613600	0.90823400	-0.84745500
C	1.20721900	0.79045900	-0.37465400
C	-1.61553300	0.72574500	0.10564200
C	0.51838900	-0.50321500	-0.20684600
O	-2.90898500	0.76281800	0.25498200
C	-0.90793000	-0.49426900	-0.11850200
C	-1.63861600	-1.71996500	-0.11065300
C	-3.09032200	-1.68694100	-0.14570600
C	-3.73031400	-0.67875400	-0.92522900
C	-5.13596000	-0.55593900	-0.90964100
C	-5.25955800	-2.39356600	0.63043900
C	-0.94506200	-2.92053500	-0.05799800
C	0.43485600	-2.92565900	-0.02704800
H	-2.62092000	3.11293200	0.75123300
H	0.97889800	5.37146300	0.20294700
H	3.01249300	4.25451100	-0.57751300
H	4.16839300	2.19059100	-1.29439800

H	3.04944100	0.03383300	-1.16636800
H	-3.18326200	-0.22365300	-1.73895900
H	-5.60350900	0.19507300	-1.53442100
H	-5.86472900	-3.04464000	1.25029900
H	-1.40901100	5.27631500	0.81417100
H	-1.48973000	-3.85712500	-0.05473400
H	0.96498500	-3.86575500	0.06039200
C	-3.89286300	-2.54460900	0.61689900
H	-3.42731500	-3.29669700	1.24165600
C	-5.90476700	-1.38314400	-0.13004900
C	1.17806800	-1.74057800	-0.08477400
C	2.64991300	-1.91231300	0.09751900
C	3.40166800	-2.65083000	-0.81304300
C	3.28903900	-1.41346100	1.23174200
C	4.75931500	-2.86185700	-0.60950500
H	2.92402000	-3.05260700	-1.69968300
C	4.64212300	-1.63237200	1.43658800
H	2.72085100	-0.84562300	1.95919200
C	5.40205900	-2.35483900	0.51678500
H	5.32725800	-3.43070800	-1.33752900
H	5.11833500	-1.23656700	2.32686400
C	-7.39150400	-1.25191500	-0.08761300
C	-7.98251800	-0.27969100	0.73116700
C	-8.19327600	-2.10627700	-0.85676900
C	-9.37046500	-0.17939500	0.76351500
C	-9.57688400	-1.96876700	-0.79848800
C	-10.18538200	-1.01055300	0.00385700
H	-9.82575400	0.56918100	1.40416100
H	-10.19439500	-2.63017000	-1.39798600
C	6.86261600	-2.58383000	0.73832000
C	7.80101900	-1.67435600	0.23191500
C	7.29568800	-3.70551200	1.45911300
C	9.15640400	-1.90388900	0.45287100
C	8.65964300	-3.89905200	1.65823100
C	9.60700000	-3.01145600	1.16109200
H	9.87843200	-1.19402900	0.06153600
H	8.98903400	-4.76776500	2.21981600
C	-7.58467000	-3.15922100	-1.74569600
H	-6.88066800	-2.72453000	-2.45924300
H	-7.03255400	-3.90605200	-1.16925700
H	-8.35846300	-3.68059400	-2.31037700
C	-7.14673900	0.64507600	1.57706000
H	-6.45016300	0.09335400	2.21250600
H	-6.54419100	1.31904500	0.96348300
H	-7.78071400	1.25570700	2.22112300
C	-11.68209100	-0.85898100	0.02805800
H	-12.01427800	-0.15027800	-0.73673100
H	-12.18223000	-1.80929000	-0.16773200
H	-12.02908100	-0.48458000	0.99292800
C	7.36772400	-0.45585400	-0.54065600
H	6.82287800	-0.72890600	-1.44776400
H	6.70230900	0.17850300	0.04982800
H	8.23139800	0.14224000	-0.83441500
C	6.31146800	-4.69729700	2.02189600
H	5.62482600	-4.22528100	2.72875600
H	5.69758800	-5.14391300	1.23602500
H	6.83029600	-5.50284300	2.54324000
C	11.07803000	-3.25828600	1.36042500
H	11.64249000	-2.32396100	1.36168900
H	11.27154900	-3.77294500	2.30361400
H	11.48129700	-3.88367800	0.55796600

cyc(S_1)

E(CAM-B3LYP/6-311+G**) = –1888.51633905

Thermal correction to Gibbs Free Energy = 0.629584

Sum of electronic and thermal Free Energies = –1887.864445

C	-1.96105900	3.74075000	0.50585900
C	-1.45213600	5.03982600	0.56282700
C	-0.13489300	5.28233800	0.26692600
C	0.73542400	4.22277500	-0.09091000
C	0.24682000	2.88689000	-0.10873400
C	-1.13733700	2.66934700	0.16552900
C	2.07355300	4.47359500	-0.46041200
C	2.87476700	3.44148800	-0.86846100
C	2.40067400	2.12498400	-0.86701200
C	1.11547900	1.80697800	-0.44694400
C	-1.65638300	1.34631100	0.05858400
C	0.59545100	0.42519600	-0.41613500
O	-2.94328000	1.16326500	0.27135700
C	-0.80691500	0.24304400	-0.28959900
C	1.40248700	-0.73872400	-0.51004900
C	-1.39681300	-1.04198900	-0.46118800
C	-2.85792500	-1.14971300	-0.39952200
C	2.88700300	-0.77120600	-0.35647400
C	-3.59433500	0.04038000	-0.80157900
C	-5.02921800	0.02141300	-0.66316800
C	-5.67478800	-1.03314600	-0.07065000
C	-4.92094100	-2.15149900	0.34750400
C	3.48232200	-0.39010200	0.84564500
C	4.85357200	-0.48865800	1.02005400
C	5.67626600	-0.96949500	0.00131900
C	5.07869800	-1.35895000	-1.19449500
C	3.70376700	-1.26798300	-1.36936500
C	-0.58232400	-2.13219300	-0.67822600
C	0.79865900	-1.98106200	-0.66225000
H	-3.00365600	3.54963100	0.71813100
H	0.25982000	6.29145400	0.29135100
H	2.44330700	5.49245100	-0.44109100
H	3.89006800	3.63377100	-1.19485100
H	3.06632200	1.35281100	-1.21300500
H	-3.24646700	0.52160400	-1.71415900
H	-5.59061400	0.86781300	-1.03936800
H	-5.42940800	-2.98473800	0.81665600
H	2.86466100	-0.01104800	1.65154200
H	5.29485300	-0.18935400	1.96435200
H	5.69629200	-1.73911100	-2.00083000
H	3.26194700	-1.57235000	-2.31153700
H	-2.10817800	5.85929900	0.83206600
C	-7.15936700	-1.02478700	0.10780900
C	-7.72098200	-0.43245200	1.24728300
C	-7.98733200	-1.61607400	-0.85556200
C	-9.10432000	-0.44060300	1.40133300
C	-9.36631600	-1.60109700	-0.66453300
C	-9.94499300	-1.01639000	0.45570000
H	-9.53514700	0.01461600	2.28764800
H	-10.00405500	-2.06113200	-1.41287500
C	7.15568100	-1.06939200	0.19155600
C	7.98115000	0.01527100	-0.13761100
C	7.72007900	-2.24828600	0.69646400
C	9.35609200	-0.09898100	0.04409700
C	9.10058400	-2.32226600	0.86408400
C	9.93608400	-1.25862400	0.54618500
H	9.99098800	0.74252100	-0.21561400
H	9.53246000	-3.23895100	1.25320500
C	-6.85490900	0.20248800	2.30343400
H	-6.12518000	-0.50502700	2.70443500
H	-6.28671600	1.04603400	1.90436400
H	-7.46243600	0.56715700	3.13269100
C	-7.41193000	-2.26179300	-2.08895600
H	-6.82779000	-1.55077800	-2.67812600
H	-6.74324400	-3.08857800	-1.83666800
H	-8.20577400	-2.65462500	-2.72546400
C	-11.43956600	-0.98235800	0.62717200

H	-11.72047900	-1.01024700	1.68174000
H	-11.86035600	-0.06647100	0.20092000
H	-11.91784100	-1.82592800	0.12596200
C	7.40514600	1.29424800	-0.68692200
H	6.87792000	1.12453900	-1.62903700
H	6.68516500	1.73969200	0.00374600
H	8.19353800	2.02558400	-0.86976400
C	6.86114500	-3.43239500	1.05593400
H	6.11960300	-3.17502400	1.81597900
H	6.30917700	-3.80603500	0.19002400
H	7.47180900	-4.24881200	1.44376200
C	11.42239500	-1.34642900	0.76397700
H	11.70199000	-0.91298000	1.72912500
H	11.76542500	-2.38255400	0.75830700
H	11.97110300	-0.80318000	-0.00807500
H	-1.01729900	-3.11128300	-0.83889600
H	1.42996700	-2.85737400	-0.74307400
C	-3.53668300	-2.19813600	0.17723700
H	-2.98654900	-3.05472700	0.54904500

cyc(S_0)

E(CAM-B3LYP/6-311+G**) = –1888.54004647

Thermal correction to Gibbs Free Energy = 0.631175

Sum of electronic and thermal Free Energies = –1887.908923

C	-1.83562000	3.65639300	0.78882800
C	-1.27803600	4.92501500	1.01282700
C	0.02540200	5.17291800	0.67763000
C	0.84325100	4.15092800	0.13534000
C	0.31498100	2.84231800	-0.00460800
C	-1.05973800	2.62909700	0.29347900
C	2.16244300	4.41012200	-0.29963700
C	2.90045300	3.41295100	-0.87476300
C	2.39064100	2.10779400	-0.96975300
C	1.13500400	1.78356500	-0.49368700
C	-1.62286400	1.33926500	-0.04100100
C	0.60037900	0.40463500	-0.46479400
O	-2.95082600	1.23135600	0.12146000
C	-0.84884100	0.26296200	-0.39873800
C	1.36721600	-0.73249600	-0.42226900
C	-1.48531900	-1.04661500	-0.43578500
C	-2.85764300	-1.11126200	-0.45670100
C	2.85275900	-0.77753300	-0.29555500
C	-3.55755800	0.19607300	-0.68827800
C	-5.01681000	0.18459000	-0.38039600
C	-5.69910700	-0.95539900	-0.21520700
C	-4.99722200	-2.23537800	-0.23593700
C	3.48580700	-0.33102600	0.86365300
C	4.86203400	-0.42557300	1.00255500
C	5.65160200	-0.97221800	-0.00854300
C	5.01635100	-1.43232500	-1.15942700
C	3.63745900	-1.34318400	-1.29839100
C	-0.61489300	-2.18498700	-0.41259400
C	0.72368600	-2.02285900	-0.41387500
H	-2.88459000	3.48073000	0.98554900
H	0.44843300	6.16286600	0.80633500
H	2.56324900	5.41232300	-0.19862700
H	3.89514500	3.61779200	-1.25240300
H	3.01007600	1.34901900	-1.42206500
H	-3.38866600	0.52282200	-1.73268000
H	-5.51447600	1.14763400	-0.35356100
H	-5.57831800	-3.14175600	-0.11562800
H	2.89360500	0.09830800	1.66341900
H	5.33269500	-0.07165100	1.91325000
H	5.60766600	-1.86722500	-1.95780600
H	3.16675300	-1.70610500	-2.20558400

H	-1.89658600	5.71547100	1.42042500
C	-7.17505100	-0.95747900	0.02384900
C	-7.67305700	-0.90341800	1.33292200
C	-8.05988300	-1.04339300	-1.05871700
C	-9.05002500	-0.92501700	1.53464200
C	-9.43057600	-1.06283400	-0.81449800
C	-9.94634100	-1.00066400	0.47447300
H	-9.43107100	-0.88521600	2.55042300
H	-10.11213700	-1.13208600	-1.65661100
C	7.13576900	-1.06804300	0.14301300
C	7.95579600	-0.02026100	-0.29893000
C	7.71019100	-2.20626400	0.72469100
C	9.33530300	-0.12885800	-0.15003200
C	9.09468500	-2.27615100	0.85579700
C	9.92504900	-1.24746800	0.42761600
H	9.96566900	0.68431400	-0.49652100
H	9.53431400	-3.16140100	1.30460600
C	-6.74536100	-0.81832800	2.51646000
H	-6.00372500	-1.62054700	2.50656000
H	-6.19059100	0.12304000	2.52171300
H	-7.30417200	-0.88561100	3.45096800
C	-7.55246800	-1.11001300	-2.47550000
H	-7.05585700	-0.18039100	-2.76507500
H	-6.82296200	-1.91280200	-2.60626600
H	-8.37344100	-1.28281100	-3.17277600
C	-11.43170000	-0.99056500	0.71605600
H	-11.68649300	-1.48938000	1.65325600
H	-11.81035600	0.03433500	0.77815900
H	-11.97033800	-1.48997400	-0.09139100
C	7.36914000	1.21399000	-0.93287600
H	6.81503800	0.97255500	-1.84334800
H	6.67039500	1.71704300	-0.26010400
H	8.15472900	1.92365900	-1.19566600
C	6.85708100	-3.35139600	1.20420700
H	6.14853600	-3.03079300	1.97165900
H	6.26797000	-3.78160000	0.39065800
H	7.47628800	-4.14357500	1.62720800
C	11.41671900	-1.32754200	0.60913400
H	11.72216800	-0.85081300	1.54564900
H	11.75921000	-2.36338700	0.64229200
H	11.94465600	-0.82133800	-0.20149200
H	-1.03528300	-3.18189200	-0.41573000
H	1.36488700	-2.89644400	-0.39665900
C	-3.65093400	-2.29417200	-0.32227000
H	-3.15670100	-3.25533900	-0.25028100

Int1$^{\bullet+}$

E(CAM-B3LYP/6-311+G**) = –1888.36403270

Thermal correction to Gibbs Free Energy = 0.632883

Sum of electronic and thermal Free Energies = –1887.731690

C	-2.05696400	3.70826300	-0.66332300
C	-1.60469600	5.02018400	-0.73059900
C	-0.28457500	5.29587500	-0.45301900
C	0.62713500	4.27499000	-0.11627700
C	0.18634200	2.92667000	-0.07911800
C	-1.18463600	2.67067200	-0.33667000
C	1.96757000	4.58006100	0.20317900
C	2.82137200	3.57756200	0.57121000
C	2.38674300	2.24694300	0.58610800
C	1.09437900	1.87930600	0.23016200
C	-1.65555200	1.33079900	-0.23408000

C	0.61013900	0.48953300	0.25685000
O	-2.93431100	1.15984000	-0.45547700
C	-0.79315100	0.25648600	0.10750700
C	1.42827400	-0.64075300	0.47623700
C	-1.37980400	-1.01765900	0.37470700
C	-2.80513900	-1.16844100	0.22601300
C	2.91419200	-0.68093500	0.35823500
C	-3.44657700	-0.19610900	-0.69937300
C	-4.92971100	-0.14415200	-0.65196500
C	-5.65039900	-1.09292300	-0.01363800
C	-4.96716700	-2.12337200	0.71316500
C	3.70936700	-1.06147700	1.43668900
C	5.08596800	-1.17397100	1.29468200
C	5.70392800	-0.92615800	0.07123800
C	4.90106800	-0.55963600	-1.00924400
C	3.52777800	-0.43749900	-0.87030200
C	-0.54640900	-2.05226800	0.77267400
C	0.82125700	-1.86271900	0.77148800
H	-3.09639500	3.48303400	-0.85981900
H	0.07590100	6.31783700	-0.48655100
H	2.29813800	5.61139700	0.16967100
H	3.84309500	3.79986200	0.85098500
H	3.09701800	1.49920500	0.89434900
H	-3.12098800	-0.41141500	-1.73110100
H	-5.41033300	0.65236100	-1.20764900
H	-5.55307700	-2.87898700	1.22150900
H	3.25197900	-1.25952400	2.39975400
H	5.68916000	-1.46395400	2.14757600
H	5.35934000	-0.37578000	-1.97443500
H	2.92519900	-0.15667400	-1.72676100
H	-2.28945700	5.81737900	-0.98776500
C	-7.14497100	-1.07844000	-0.03179700
C	-7.84775400	-0.40114400	0.97364100
C	-7.83645800	-1.76012900	-1.04098600
C	-9.23851600	-0.41200400	0.94575800
C	-9.22815800	-1.74609800	-1.03101500
C	-9.94796000	-1.07553800	-0.04924800
H	-9.78169300	0.10949800	1.72731900
H	-9.76228700	-2.27774000	-1.81180300
C	7.18374100	-1.06445200	-0.08530900
C	8.02221200	0.02780000	0.16403200
C	7.73121300	-2.29366900	-0.48302600
C	9.39867300	-0.12527400	0.00956800
C	9.10926600	-2.40476400	-0.62493900
C	9.96203500	-1.33151000	-0.38469400
H	10.04534400	0.72395300	0.20505200
H	9.52860000	-3.35798900	-0.93160100
C	-7.12716000	0.33089400	2.07535500
H	-6.45733700	-0.33172500	2.62964800
H	-6.51619900	1.14756600	1.68267200
H	-7.83832800	0.75566200	2.78445200
C	-7.10408300	-2.50233200	-2.12804900
H	-6.52608200	-1.82216000	-2.75932300
H	-6.40205100	-3.23282000	-1.71772600
H	-7.80516200	-3.03617300	-2.77019800
C	-11.45147100	-1.04709100	-0.07426000
H	-11.86594700	-1.02003000	0.93518100
H	-11.81452300	-0.15934200	-0.60087700
H	-11.85784100	-1.92094400	-0.58619800
C	7.46433700	1.35850100	0.59700200
H	6.74865000	1.74956300	-0.13060900
H	6.94067200	1.28150000	1.55338600
H	8.26236200	2.09305600	0.71147000
C	6.85348400	-3.48719700	-0.75448900
H	6.26015300	-3.75700700	0.12269200
H	6.14924500	-3.29239700	-1.56726200
H	7.45583600	-4.35276600	-1.03251100
C	11.44893300	-1.48262100	-0.55666300
H	11.83354200	-2.31238200	0.04154100

H	11.70456100	-1.68935100	-1.59950400
H	11.97885300	-0.57763000	-0.25625900
H	-0.94956100	-3.03017300	0.99879000
H	1.46525800	-2.70958000	0.97711400
C	-3.58285500	-2.12824200	0.84047900
H	-3.12005700	-2.87139400	1.47818800

Int2

E(CAM-B3LYP/6-311+G**) = –2336.03144084

Thermal correction to Gibbs Free Energy = 0.670969

Sum of electronic and thermal Free Energies = –2335.360472

C	1.99238000	2.71445600	-0.23551900
C	1.43726300	3.91635700	-0.12138700
C	-0.03357000	4.04636800	0.18723100
C	-0.83330100	2.90041300	-0.43543000
C	-0.22814100	1.61082100	-0.40635700
C	1.19390400	1.50123200	-0.22274700
C	-2.10715100	3.01567100	-0.94457200
C	-2.81944800	1.88788100	-1.36247100
C	-2.29247000	0.63453600	-1.19454500
C	-0.99597900	0.45580400	-0.67598400
C	1.77198200	0.26720800	-0.24470500
C	-0.39363800	-0.86524800	-0.48926100
O	3.13044600	0.23012400	-0.16890900
C	1.01585000	-0.94333300	-0.34581000
C	-1.12583100	-2.08259700	-0.44234000
C	1.69615000	-2.18382900	-0.29907200
C	3.15901000	-2.17484100	-0.18029400
C	-2.60897800	-2.19223000	-0.31928200
C	3.81484200	-0.95102100	-0.12511200
C	5.19469500	-0.85021300	-0.02109200
C	5.97197600	-1.99712300	0.03354700
C	5.33119700	-3.23897100	-0.01899700
C	-3.35645700	-2.88082800	-1.27187300
C	-4.72772600	-3.04047700	-1.12068500
C	-5.39066400	-2.52782900	-0.00865000
C	-4.63686400	-1.85217100	0.95102600
C	-3.26919300	-1.68603200	0.79975600
C	0.95366600	-3.34428700	-0.36617500
C	-0.43254300	-3.28138900	-0.40936700
H	3.06070500	2.62152500	-0.37621800
H	-2.58766600	3.97343200	-1.08192900
H	-3.79963600	2.00948000	-1.80626100
H	-2.87363100	-0.21731400	-1.50535900
H	5.64537200	0.13407200	0.01827100
H	5.92219100	-4.14613700	0.02196600
H	-2.86339000	-3.28446800	-2.14911800
H	-5.29078200	-3.57290800	-1.87927900
H	-5.12930000	-1.45432200	1.83145900
H	-2.70605200	-1.15880700	1.56121400
H	2.05265400	4.80564600	-0.15615400
C	7.45907300	-1.90478500	0.14796500
C	8.24693000	-1.82844000	-1.00874800
C	8.06448700	-1.90275300	1.41150900
C	9.63048200	-1.75059300	-0.88002400
C	9.45171500	-1.82234300	1.49612600
C	10.25270400	-1.74327400	0.36320100
H	10.23769200	-1.69638900	-1.77813700
H	9.91697500	-1.82290800	2.47674000
C	-6.86585100	-2.70382500	0.15702100
C	-7.75155500	-1.76509000	-0.38962800
C	-7.36480000	-3.80585600	0.86501200
C	-9.12132700	-1.94563500	-0.21962800
C	-8.74146800	-3.95066700	1.01187600
C	-9.63721700	-3.03327800	0.47526400

H	-9.80304000	-1.21353300	-0.64103400
H	-9.12227500	-4.80487700	1.56288700
C	7.62277700	-1.83302300	-2.37966200
H	7.02661300	-2.73324400	-2.54748400
H	6.95449500	-0.97989500	-2.51986200
H	8.39011400	-1.78823700	-3.15347800
C	7.24331100	-1.98680300	2.67145700
H	6.54045700	-1.15374600	2.74924000
H	6.65171800	-2.90486200	2.70611900
H	7.88594100	-1.96767100	3.55252800
C	11.74829700	-1.62503300	0.47822200
H	12.25201100	-2.10965500	-0.36037700
H	12.05716900	-0.57524500	0.48267600
H	12.11394000	-2.07758500	1.40172400
C	-7.24623500	-0.56711900	-1.15055300
H	-6.58309000	0.04741300	-0.53671800
H	-6.67659800	-0.86410600	-2.03457300
H	-8.07621900	0.05880000	-1.48105600
C	-6.43859800	-4.82908400	1.46854700
H	-5.79956400	-5.28883400	0.71083100
H	-5.77494900	-4.38170800	2.21253600
H	-7.00556300	-5.62211300	1.95783100
C	-11.12258300	-3.22717600	0.61758500
H	-11.52117000	-3.81265200	-0.21665800
H	-11.36858000	-3.76046200	1.53776400
H	-11.65037600	-2.27167200	0.62737400
H	1.43426500	-4.31270600	-0.34238300
H	-1.00002100	-4.20364700	-0.37668700
C	3.95627800	-3.32211200	-0.12324300
H	3.49429300	-4.29986000	-0.16223500
C	-0.56219000	5.50556600	-0.07288200
C	-1.87567500	5.80737100	0.75980300
C	0.44863500	6.48747600	0.36343000
C	-0.81699600	5.74708800	-1.50220300
C	-1.64060400	5.65578400	2.19875600
C	-2.42686100	7.13408400	0.47023000
N	1.20975300	7.26055300	0.72867700
N	-1.01351300	5.93695100	-2.61381100
N	-1.47187900	5.51360500	3.32180200
N	-2.87433500	8.15891600	0.22739000
H	-2.63254400	5.07165800	0.48485800
H	-0.12802600	3.93354400	1.27625400
